# Phenotypic Variation and the Impact of Admixture in the *Oryza rufipogon* Species Complex (*ORSC*)

**DOI:** 10.3389/fpls.2022.787703

**Published:** 2022-06-13

**Authors:** Georgia C. Eizenga, HyunJung Kim, Janelle K. H. Jung, Anthony J. Greenberg, Jeremy D. Edwards, Maria Elizabeth B. Naredo, Maria Celeste N. Banaticla-Hilario, Sandra E. Harrington, Yuxin Shi, Jennifer A. Kimball, Lisa A. Harper, Kenneth L. McNally, Susan R. McCouch

**Affiliations:** ^1^Dale Bumpers National Rice Research Center, USDA-ARS, Stuttgart, AR, United States; ^2^Plant Breeding and Genetics Section, School of Integrative Plant Science, Cornell University, Ithaca, NY, United States; ^3^Bayesic Research LLC., Ithaca, NY, United States; ^4^International Rice Research Institute, Los Baños, Philippines

**Keywords:** rice, Bayesian Gaussian mixture models, *Oryza rufipogon*, *Oryza sativa*, *Oryza nivara*, genebank accessions, crop wild relatives, *Oryza rufipogon* species complex

## Abstract

Crop wild relatives represent valuable reservoirs of variation for breeding, but their populations are threatened in natural habitats, are sparsely represented in genebanks, and most are poorly characterized. The focus of this study is the *Oryza rufipogon* species complex (*ORSC*), wild progenitor of Asian rice (*Oryza sativa* L.). The *ORSC* comprises perennial, annual and intermediate forms which were historically designated as *O. rufipogon, O. nivara*, and *O. sativa* f. *spontanea* (or *Oryza* spp., an annual form of mixed *O. rufipogon/O. nivara* and *O. sativa* ancestry), respectively, based on non-standardized morphological, geographical, and/or ecologically-based species definitions and boundaries. Here, a collection of 240 diverse *ORSC* accessions, characterized by genotyping-by-sequencing (113,739 SNPs), was phenotyped for 44 traits associated with plant, panicle, and seed morphology in the screenhouse at the International Rice Research Institute, Philippines. These traits included heritable phenotypes often recorded as characterization data by genebanks. Over 100 of these *ORSC* accessions were also phenotyped in the greenhouse for 18 traits in Stuttgart, Arkansas, and 16 traits in Ithaca, New York, United States. We implemented a Bayesian Gaussian mixture model to infer accession groups from a subset of these phenotypic data and ascertained three phenotype-based group assignments. We used concordance between the genotypic subpopulations and these phenotype-based groups to identify a suite of phenotypic traits that could reliably differentiate the *ORSC* populations, whether measured in tropical or temperate regions. The traits provide insight into plant morphology, life history (perenniality versus annuality) and mating habit (self- versus cross-pollinated), and are largely consistent with genebank species designations. One phenotypic group contains predominantly *O. rufipogon* accessions characterized as perennial and largely out-crossing and one contains predominantly *O. nivara* accessions characterized as annual and largely inbreeding. From these groups, 42 “core” *O. rufipogon* and 25 “core” *O. nivara* accessions were identified for domestication studies. The third group, comprising 20% of our collection, has the most accessions identified as *Oryza* spp. (51.2%) and levels of *O. sativa* admixture accounting for more than 50% of the genome. This third group is potentially useful as a “pre-breeding” pool for breeders attempting to incorporate novel variation into elite breeding lines.

## Introduction

Wild relatives of domesticated crop species are some of the greatest sources of untapped genetic variation available to plant breeders as they confront the challenges of a changing climate. Yet populations of crop wild relatives are underrepresented in genebanks, threatened in their natural environments, and poorly characterized in both *ex situ* and *in situ* collections. The *Oryza rufipogon* species complex (*ORSC*), previously referred to as *O. rufipogon* Griff. or *O. perennis* Moench, is the wild progenitor of Asian rice (*Oryza sativa* L.) and is comprised of perennial, annual, and intermediate ecotypes (Sano et al., 1980; Oka, 1988). As summarized in Supplementary Table 1, the perennial ecotype exhibits vigorous vegetative growth, is largely out-crossing, and is found in areas which are continuously wet while the annual ecotype shows less vigorous vegetative growth, is primarily in-breeding, and is found in areas that are seasonally wet (Oka and Morishima, 1967; Morishima et al., 1984; Vaughan et al., 2008). In accordance with these differences in life history, the perennial form is capable of reproducing clonally via stolons, typically has low seed productivity, is late flowering and photoperiod sensitive, while the annual form lacks stolons, has high seed productivity, is frequently early-flowering and photoperiod insensitive (Morishima et al., 1984; Barbier, 1989a,b; Barbier et al., 1991; Morishima, 2001; Vaughan et al., 2003, 2008; Cai et al., 2004). An extensive survey of both annual and perennial ecotypes of the *ORSC* led Sharma and Shastry (1965) to treat the annual form as a separate species, designated *O. nivara*, as originally suggested by Chatterjee (1948), to distinguish it from the perennial *O. rufipogon*. A weedy, intermediate form is classified as *O. sativa* f. *spontanea* Roschev (Roschevicz, 1931; Oka, 1988) and sometimes erroneously designated *O. spontanea* (Oka, 1974; Sano et al., 1980; Vaughan, 1989; Hill, 2010). Weedy rice is considered an annual of mixed *O. rufipogon/O. nivara* and *O. sativa* ancestry (Morishima et al., 1961; Chang, 1976; Vaughan et al., 2001; Sharma, 2003).

To this day, wide differences in the nomenclature and trait-based definitions assigned to the wild ancestor of *O. sativa* persist in rice genebank databases and research labs around the world. According to the online database of the International Rice Germplasm Collection (IRGC)^1^ the list of available *ORSC* accessions includes 846 *O. rufipogon*, 1,455 *O. nivara*, and 1,134 accessions listed as either ‘*Oryza* spp.’ or ‘*Oryza* hybr.’. Many of the *Oryza* spp. or *Oryza* hybr. were previously designated as *O. spontanea (O. sativa* f. *spontanea)* or referred to as hybrids between *O. sativa* and *O. rufipogon* and/or *O. nivara*. The online wild strain database of the Japanese National Institute of Genetics, *Oryzabase* (Kurata and Yamazaki, 2006; Yamazaki et al., 2010), acknowledges confusion in the wild rice taxonomy and nomenclature, including a distinction between *O. nivara* and *O. rufipogon* based on ecology and life habit, but clearly states its use of the *sensu lato* classification of *O. rufipogon* as a single species with a continuous range of annual, perennial, and intermediate types as held by core collectors Morishima and colleagues (Morishima et al., 1961). There are 682 accessions in *Oryzabase* designated as ‘*O. rufipogon*’ with additional, but incomplete information on former species designations (i.e., ‘*O. perennis’, ‘O. perennis (O. nivara),’* or ‘*O. sativa f. spontanea*,’), and life habit designations, such as annual or perennial^2^.

While the species designations originally assigned to accessions in the IRGC have persisted largely unchanged through the decades, there has been a paradigm shift in biology from morphology-based to genetic identity-based species definitions. In the case of the *ORSC*, studies using molecular markers (isozymes, RFLPs, SSRs, SINEs and other indels, and SNPs) present conflicting and often contradictory results that collectively fail to support the existence of two, well-differentiated species. Global studies of genomic diversity in the *ORSC* document three to eight genetically distinct groups (Londo et al., 2006; Zheng and Ge, 2010; Huang et al., 2012; Banaticla-Hilario et al., 2013a,b; Liu et al., 2015; Kim et al., 2016) more closely associated with geography than with life-habit. Pronounced genetic differentiation has been reported between *O. rufipogon* and *O. nivara* only in studies involving local collections of germplasm from South Asia (Samal et al., 2018) and Southeast Asia (Kuroda et al., 2006; Banaticla-Hilario et al., 2013a), where both species occur sympatrically but remain differentiated due to differences in flowering time and ecological adaptation. Genetic differentiation has not been documented in China or Oceania where native populations of *O. nivara* are largely absent (Vaughan, 1994; Liu et al., 2015).

Several studies based on genome-wide SNPs have examined relationships among *ORSC* accessions and among *ORSC* and *O. sativa* subpopulations. A study by Huang et al. (2012) examined a panel of 446 *ORSC* accessions and 1,083 *O. sativa* cultivars that had been genotyped with ∼5M SNPs and identified three major groups of *ORSC* accessions using a neighbor-joining tree. These groups were significantly correlated with geographic distribution and were referred to as *OrI, OrII* and *OrIII*. Subsequently, *OrI* was divided into two sub-clades, where *OrIa* included *ORSC* accessions and rice belonging to the *O. sativa-indica* subpopulation and *OrIb* included *ORSC* and *O. sativa-aus* rices; *OrIII* was also divided into two sub-clades, where *OrIIIa* included *ORSC* accessions from Southern China as well as *O. sativa-japonica* rices, and *OrIIIb* included *ORSC* accessions that clustered independently of *O. sativa*.

The *ORSC* dataset generated by Huang et al. (2012) was subsequently re-analyzed by three independent research groups: Civán et al. (2015), Wang et al. (2017), and Choi and Purugganan (2018). In each case the authors reached very different conclusions about the subpopulation structure of the *ORSC* and relationships with the cultivated gene pools of *O. sativa.* These subsequent studies revealed evidence of substantial gene flow from domesticated groups into wild populations and suggested that introgression rather than phylogenetic relationship was a primary driver of the *ORSC* groups reported by Huang et al. (2012). Use of larger K values (population number) and local ancestry estimation techniques helped to resolve the heavily *O. sativa*-admixed wild groups as distinct from independent wild populations. The analysis by Wang et al. (2017) combined the sequence data of 435 *ORSC* accessions (Huang et al., 2012) with sequencing data from 203 *O. sativa* accessions included in the Rice Minicore collection (Wang et al., 2016). Subsequent STRUCTURE analysis (*K* = 9) ascertained six *ORSC* subpopulations: four unique *ORSC* subpopulations that were correlated with geographic distribution and two that clustered with *O. sativa* subpopulations. Of these, *Or-A* accessions had the broadest range with the highest proportion from Oceania, *Or-B* accessions were almost exclusively found in China, *Or-C* had the highest proportion from West India and Sri Lanka, and *Or-D* was mainly from the SE Asia, Bangladesh, and East India, *Or-E* accessions clustered with *O. sativa*-*aus* accessions, and *Or-F* clustered with *O. sativa-indica* (Wang et al., 2017). Of note, about 42% of these *ORSC* accessions were deemed to be substantially admixed, thus could not be assigned to a single ancestry group.

To further disentangle the population dynamics of the *ORSC*, a different collection of 286 accessions from the IRGC was genotyped using genotyping-by-sequencing (113,739 SNPs) and evaluated at 25 polymorphic sites in the chloroplast genome (Kim et al., 2016). Six wild subpopulation groups were identified; three were closely related to *O. sativa-indica, -aus*, and -*japonica*, respectively, while the other three subpopulations were genetically divergent, had unique chloroplast haplotypes, and were located at the geographical extremes of the species range.

Another study examined local differentiation between *O. rufipogon* and *O. nivara* using a collection of 52 pairs of sympatric *O. rufipogon* and *O. nivara* accessions that were phenotyped for 32 traits (Banaticla-Hilario et al., 2013a) and genotyped with 29 SSR markers (Banaticla-Hilario et al., 2013b). The genotypic neighbor-joining tree showed no clear-cut genetic separation of *O. nivara* and *O. rufipogon* accessions though, species separation was apparent at the local scale. Nonetheless, Bayesian clustering was able to differentiate the two species in sympatric population pairs across the entire range of their distribution (Banaticla-Hilario et al., 2013a). Furthermore, this study identified a cluster of *O. nivara* accessions from Nepal that diverged significantly from the other population groups (similar to the findings reported by Kim et al., 2016), as well as two or three other genetically and geographically distinct subpopulations of *O. nivara*, suggesting that reproductive barriers in this inbreeding group may tend to intensify under sympatric conditions, and that *O. nivara* may have originated more than once from its perennial ancestor. Local differences in flowering time or in floral and panicle structure associated with the mating system would impact adaptation and have been known to drive reproductive isolation in closely related species (Grillo et al., 2009; Liu et al., 2015). Statistical analysis of the phenotypic data showed 27 of the 32 traits were significantly different between *O. rufipogon* and *O. nivara* with spikelet width, anther length, culm length and photoperiod explaining 25.4 percent of the variation between the two species. These studies also noted that Southeast Asian accessions appeared to be more recently diverged and/or had more interspecific gene flow compared to those from South Asia.

To augment our understanding of diversity and population structure in the *ORSC* and to facilitate the selection of materials for use in plant breeding, we phenotyped the collection genotyped by Kim et al. (2016). Phenotyping was performed at three locations (Los Baños, Philippines, Arkansas, United States and New York, United States) for heritable traits, with emphasis on those commonly used to characterize rice accessions in genebanks (Bioversity International et al., 2007). Several of the traits were associated with differences in life habit and/or mating system and served to substantiate the distinction between *O. nivara* and *O. rufipogon* as distinct ecotypes within the *ORSC*. A significant proportion of *ORSC* accessions carried unexpected combinations of phenotypes and were associated with admixture from *O. sativa*.

To enable a more robust interpretation of the phenotypic variation observed, we also merged genotypic datasets for the 286 *ORSC* accessions analyzed by Kim et al. (2016) and the 446 *ORSC* accessions analyzed by Huang et al. (2012), Civán et al. (2015), Wang et al. (2017), and Choi and Purugganan (2018) and analyzed the extent of unique or shared variation in each of the studies. Based on these analyses, we identify a set of ‘core accessions’ representing *O. rufipogon* and *O. nivara* along with a large group of *ORSC* accessions that represent unique sources of naturally occurring, highly admixed pre-breeding material for use in plant breeding. This work adds value to the *ORSC* genetic resources currently conserved in the IRGC and enhances opportunities for expanded utilization in research and plant improvement.

## Materials and Methods

### Plant Materials and Phenotypic Data Collection

For this study, 240 *ORSC* accessions from the International Rice Germplasm Collection (IRGC) previously genotyped by Kim et al. (2016), were selected for phenotypic evaluation (Supplementary Table 2). This collection included 102 *ORSC* accessions from Southeast Asia, 79 accessions from South Asia, 55 accessions from East Asia and four accessions from Australasia. Of these, 222 accessions were evaluated in a screenhouse at the IRRI, Los Baños, Philippines and 130 were evaluated under greenhouse conditions in the United States, with 112 accessions phenotyped in more than one location. At IRRI two plants were grown of each accession with one plant per pot and each pot was considered a single replication. Seed of each accession was planted between June 20 and July 4, 2008, and the plants were phenotyped for the 44 morphological traits related to the vegetative, reproductive and harvest growth stages (Supplementary Table 3). Twenty-two of these 44 traits were measured five times on an individual plant to improve the accuracy of the measurement (Supplementary Table 4).

In the United States, 104 accessions were phenotyped at both Cornell University (CU) in Ithaca, New York and the Dale Bumpers National Rice Research Center (DB) near Stuttgart, Arkansas. Three plants were grown of each accession with one plant per pot and each pot was considered a single replication. At CU, 104 accessions were planted between August 31 and September 8, 2008, and each plant was phenotyped for 16 morphological traits. At DB, 129 *ORSC* accessions were grown in the greenhouse with three plants per accession, one plant per pot, each pot was considered one replication, and plants were phenotyped for 18 morphological traits. In Arkansas, each accession was phenotyped in two different years, thus a total of six plants were characterized for most accessions. The accessions were planted August 24 to September 6, 2007 (86 accessions); August 1 to September 12, 2008 (124 accessions) and August 12 to 13, 2009 (41 accessions). Supplementary Table 2 lists the location where each accession was grown.

Supplementary Table 3 summarizes the 57 phenotypic traits characterized across the three locations and indicates the location(s) where the trait data were collected. Additional details about the collection of phenotypic data at IRRI are included in Bioversity International et al. (2007). Also listed are the corresponding Planteome acronyms and the trait ontology or crop ontology terms (Cooper et al., 2018). At IRRI, for the 22 quantitative traits with five measurements per plant, the within-plant variances were very small, thus a mean was calculated for subsequent analyses. The replicated trait data collected for each accession by location was used to calculate a best linear unbiased estimator (BLUE) for each trait and accession by location as described in the next section. The BLUEs are listed by location in Supplementary Table 4.

### Data Quality

Using the replicated trait measurements taken at each location and a relationship matrix constructed from the GBS genotypes (Kim et al., 2016), we fit multi-trait Bayesian hierarchical models (Greenberg et al., 2011) to the data from each site separately. The model is:


   yi⋅∼tv(μj[i]⋅acc;Σe)e,dμj⋅acc∼Nd(μ+uj⋅Γ;Σs)  γj⋅∼Nd(0d;Σa)

Location parameters of the form ***y***_i_**.** are row-vectors of the corresponding parameter matrices that have data points as rows and traits as columns. The overall intercept μwas modeled with a high-variance (***I***$\sigma_{0}^{2}$ = 10^6^) Gaussian prior. The errors were modeled using a multivariate Student-*t* distribution with three degrees of freedom to dampen the effects of outliers (Greenberg et al., 2010; Greenberg et al., 2011). We computed the parameters using a C++ program based on an openly-available library^3^ that implements methods described by Greenberg et al. (2011).

The marker effects enter through the eigenvectors ***U*** of the relationship matrix, weighted by square roots of their eigenvalues. We estimated the relationship matrix from all non-singleton SNPs in each data set using the van Raden method (VanRaden, 2008). We used all the eigenvectors that correspond to non-zero eigenvalues. If this regression is performed using a Gaussian prior on the coefficients γ_*j*._, it is the Bayesian analog of the (matrix-variate) mixed effect model (Kang et al., 2010; Hoffman, 2013). The accession means $\mu_{j.}^{a\,c\,c}$are analogous to BLUEs. Modes of their posterior distributions were used in the subsequent analyses.

The covariance matrices Σ*^x^* were modeled using Wishart distributions with weakly-informative Wishart priors with two degrees of freedom for Σ*^e^* and Σ*^s^* and four degrees of freedom for Σ*^a^* (Gelman et al., 2004; Greenberg et al., 2011). This implies a uniform prior on narrow-sense (marker) heritability. Estimated model parameters can then be used to calculate marker heritability:


$$h_{M,p}^{2}=\frac{{\left(U\,\Gamma \right)}_{\cdot p}^{T}\,{\left(U\,\Gamma \right)}_{\cdot p}/\left(N_{a\,c\,c}-1\right)}{{\left(U\,\Gamma \right)}_{\cdot p}^{T}\,{\left(U\,\Gamma \right)}_{\cdot p}/\left(N_{a\,c\,c}-1\right)+\Sigma_{p,p}^{s}+\Sigma_{p,p}^{e}},$$

where *N*_acc_ is the number of accessions, and the rest of the notation is as described above. We used the variance of genome-estimated breeding values (GEBV).


$${\left(U\,\Gamma \right)}_{\cdot p}^{T}\,{\left(U\,\Gamma \right)}_{\cdot p}/\left(N_{a\,c\,c}-1\right)$$

rather than values from the marker covariance matrix $\Sigma_{p,p}^{a}$because the latter reflects the additive covariance only when the prior on the principal component regression (see above) is Gaussian. The $\Sigma_{p,p}^{s}$ values estimate the portion of total genetic variance not explained by genotyped markers. They include the non-additive effects and additive effects not tagged by SNPs. The sum of the GEBV and background variance, divided by total phenotypic variance, is broad-sense heritability (Lynch and Walsh, 1998).

### Mixture Model

Using estimates of phenotypic values for each accession, we fit a Gaussian mixture model (Robert, 1996) using a variational Bayes approach (McGrory and Titterington, 2007). The model is


$$\mu_{j,\cdot }^{a\,c\,c}∼\frac{1}{C}\,\sum_{m=1}^{N_{M}}N_{d}\,{\left(\mu_{m,\cdot };\Sigma_{A,m}\right)}^{z_{j\,m}}$$

    zjm∼Bernoulli(πm)    πm∼Dirichlet(α)   μm⋅∼NNM,d(0;λ0ΣA,m)ΣA,m∼W(Σ;0υ0)

where the notation is the same as for the hierarchical model above and **μ***_*m*_****_⋅_*** is a row vector of group means. Each group has a separate mean and covariance matrix. The prior population size **α** is set to a small value, shrinking groups with few members to zero. The number of groups must be set *a priori*.

We perform inference by running the model multiple times from different starting points and picking the best fit, as assessed using the deviance information criterion (DIC, McGrory and Titterington, 2007). Small DIC values indicate better fit. Our implementation of the model is available as the R package MuGaMix^4^. We use two methods to assess how many groups are statistically supported: the DIC and the number of groups with at least two accessions. We include the pipeline (implemented in R) that runs the mixture model, processes the results, and generates all the plots and summary tables presented in this manuscript in the Supplementary Material File 1.

### Merged Population Structure Analysis

For population structure analysis, the SNP datasets from Kim et al. (2016) and Huang et al. (2012) were merged into a single dataset. First, the Kim et al. (2016) SNPs from *ORSC* and *O. sativa* accessions were imputed using Beagle 5.0 (Browning et al., 2018). Next the intersection between the SNPs in the imputed Kim et al. (2016) and the (already imputed) SNPs in the Huang et al. (2012) was determined and a merged file was generated using BCFtools version 1.14 (Danecek et al., 2021). Population structure analysis was done using fastSTRUCTURE version 1.0 (Raj et al., 2014) with *K* values ranging from 2 to 9.

### RFMix Analysis

The local ancestry of the *ORSC* individuals was determined using RFMix (Maples et al., 2013). The training panel included ten individuals from each subpopulation (W1-W6) with the highest global ancestry as reported by Kim et al. (2016) and included individuals from each of the five *O. sativa* subpopulations, *indica* (IND) (10 individuals), *aus*, (AUS) (9 individuals), *temperate japonica* (TEJ) (9 individuals), *tropical japonica* (TRJ) (10 individuals), and *aromatic* (ARO) (6 individuals). Of the 60 *ORSC* individuals in the training panel, 49 were phenotyped in the current study. Prior to RFMix analysis, the genotypic data were imputed and phased using Beagle 5.0 (Browning et al., 2018).

## Results

### Evaluation of Phenotypic Data

Broad-sense and marker heritability distributions for each trait and location are presented in Supplementary Table 5. Heritabilities range from 0.98 to 0.33 and are generally moderate to high. Markers account for at least 33% of total genetic variance across all traits and locations, but it is the generally high *H*^2^ that gives confidence in within-site reproducibility of our phenotypic measurements.

Fourteen traits were measured at more than one site. We tested among-site reproducibility of these measurements by estimating correlations among phenotypes evaluated on the same accessions and generally see positive correlations (Supplementary Figure 2 and Supplementary Table 6), again suggesting that phenotypic measurements are reproducible across sites despite the diverse climatic conditions. One notable exception is panicle number (PNNB). Values of this trait are positively correlated between Cornell and Dale Bumpers (temperate and sub-tropical sites, respectively), but both are negatively correlated with IRRI measurements (tropical site), suggesting a genotype by environment interaction.

### Inferring Phenotypic Groups

Using estimates of 32 phenotypic values (binary traits were excluded) for each accession in the IRRI data set (n = 222), we fit a Gaussian mixture model (Robert, 1996) using a variational Bayes approach (McGrory and Titterington, 2007). To infer the underlying number of groups that is statistically supported and biologically meaningful, we ran analyses assuming two to 15 groups *a priori* and assessed model fit (Supplementary Figure 3) using the deviance information criterion and the number of non-empty groups. Both metrics lend statistical support for the division of our accessions into as many as 10 phenotypic groups. However, to make biologically relevant inferences we need a parsimonious classification that is not only statistically significant, but also robust to model uncertainty, reproducible, and interpretable given other biological information. The variational Bayes approach we used is fast but produces only point estimates of parameters. To test robustness of these estimates to initial conditions, we started the model fitting process from five independent sets of initial values. Model fit metrics were consistent across initial value sets (Supplementary Figure 3).

Moving beyond model fit estimates, we tested stability of group membership across model specifications and starting value sets. To anchor these inferences, we started by noting taxonomic classification of our accessions, as found in the IRRI-GRIN-Global database. Based on traditional classification methods, accessions in this database are listed as either *O. rufipogon*, *O. nivara*, or, if the taxonomic assignment is uncertain, as *Oryza* spp. or *Oryza* hybr. We combined the latter two categories into one, calling it *Oryza* spp. We tracked each accession, starting from the species designations, across phenotypic groups inferred given different *a priori* group numbers (*N*_*G*_) using a Sankey plot (Supplementary Figure 4; Kennedy and Sankey, 1898; implemented in the ggsankey R package)^5^. A line, which is like a ribbon in such plots, represents a single accession, while boxes arranged along the *y*-axis are groups. Groups derived from models assuming a different number of groups are shown along the *x*-axis. We can thus follow a given accession as its line connects to each group across models with different *N*_*G*_. If we set *N*_*G*_ = 3, the model infers one large group comprising most accessions. The second largest group is composed predominantly of accessions classified as *Oryza* spp. Increasing the *a priori N_*G*_* to four, we can assign most *O. rufipogon* and *O. nivara* accessions to separate groups. If we further increase *N*_*G*_, distinct groups corresponding to the traditional species designations are maintained, with an additional group that comprises most *Oryza* spp. accessions. However, additional small groups inferred with higher *a priori N*_*G*_ do not appear to maintain coherent membership across model runs (Supplementary Figure 4). Thus, while there is statistical support for finer classification, these groupings are not robust to model specification. We therefore focused our analyses on inferences assuming four *a priori* groups.

**FIGURE 1:**
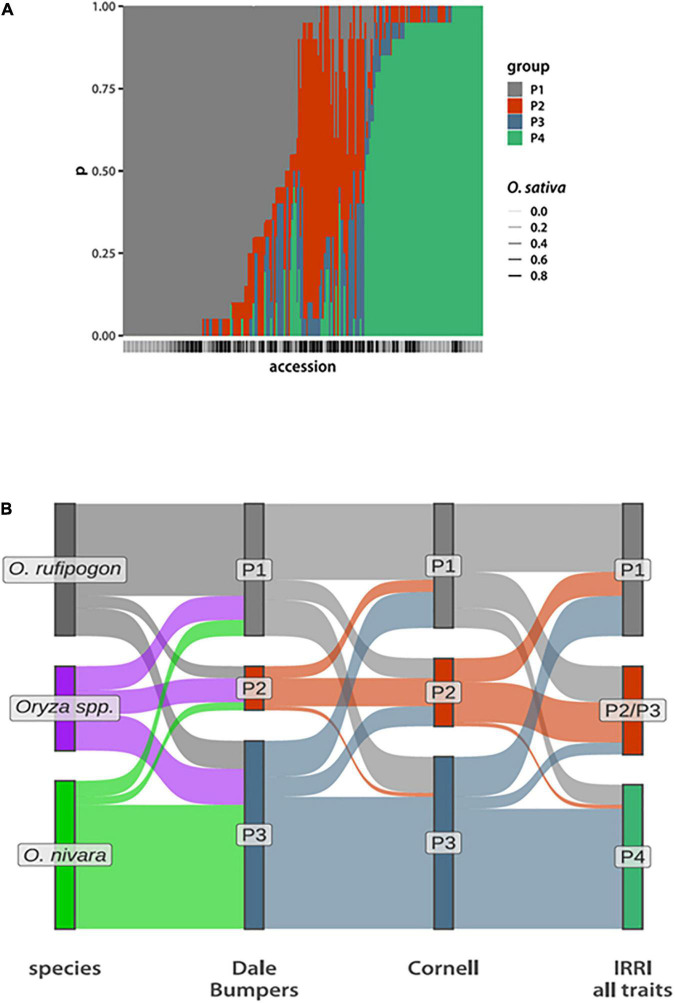
Phenotypic group composition. **(A)** Bar plot depicting posterior probabilities that a given accession (along the *x*-axis) belongs to each of the four phenotypic groups. The bars are stacked, with the total probability summing to 1. The bars below the x-axis indicate the *O. sativa* genome fraction, with the darker bars having a higher *O. sativa* fraction. (The actual values are in Supplementary Table 2) **(B)** Sankey plots comparing compositions of phenotypic groups inferred from data collected at Dale Bumpers and Cornell assuming *N_*G*_* = 3 compared to species designations and the four groups inferred from data collected at IRRI. Each accession is a single “ribbon” running from left to right and colored according to the grouping to the left.

Given that models with *N*_*G*_ = 4 appear to be the most parsimonious, while still capturing biologically meaningful stratification of the *ORSC*, we next tested these models for robustness. We started model fitting from 20 sets of initial conditions and averaged posterior probabilities that a given accession belongs to a particular group across runs. We see a set of accessions that reproducibly belong to the P1 or P4 groups (Figure 1A), corresponding to *O. rufipogon* and *O. nivara*, respectively. The P3 group has the least reproducible membership. This is because for some initial conditions, a small number of accessions are assigned to it, while for others these lines are placed in P2 (see Supplementary Material 1 for a detailed analysis of these shifts). This may be due to the approximate nature of the variational Bayes inference. Therefore, we added the posterior probabilities of P2 and P3 assignment in subsequent analyses and called the resulting group P2/P3.

The resulting P1 phenotype group contains 106 accessions, of which 82 (78%) are classified as perennial *O. rufipogon* species. In contrast, P4 has 73 accessions with 43 (59%) classified as *O. nivara.* About half of the P2/P3 group are designated as *Oryza* spp. in the IRGC, with the remaining accessions about equally divided between *O. nivara* and *O. rufipogon* (Supplementary Figure 5 and Supplementary Table 2).

To further test the reproducibility of phenotypic group inference, we used data collected at DB and CU. These data sets include different, but overlapping, phenotype sets that were measured on fewer than half the accessions represented in the IRRI data (Supplementary Tables 2, 3). Despite this, we can still identify a similar set of three groups (Figure 1B). This time, setting *N*_*G*_ = 3 *a priori* is sufficient to separate *O. rufipogon* and *O. nivara* from each other and from a group that largely corresponds to the *Oryza* spp. designation from IRRI-GRIN-Global. Taking these observations together with the above statistical robustness analyses, we see that a core set of accessions can be reproducibly assigned to a phenotypic group, while inference for others is less certain.

### Subsets of Traits Defining Phenotypic Groups

**FIGURE 2:**
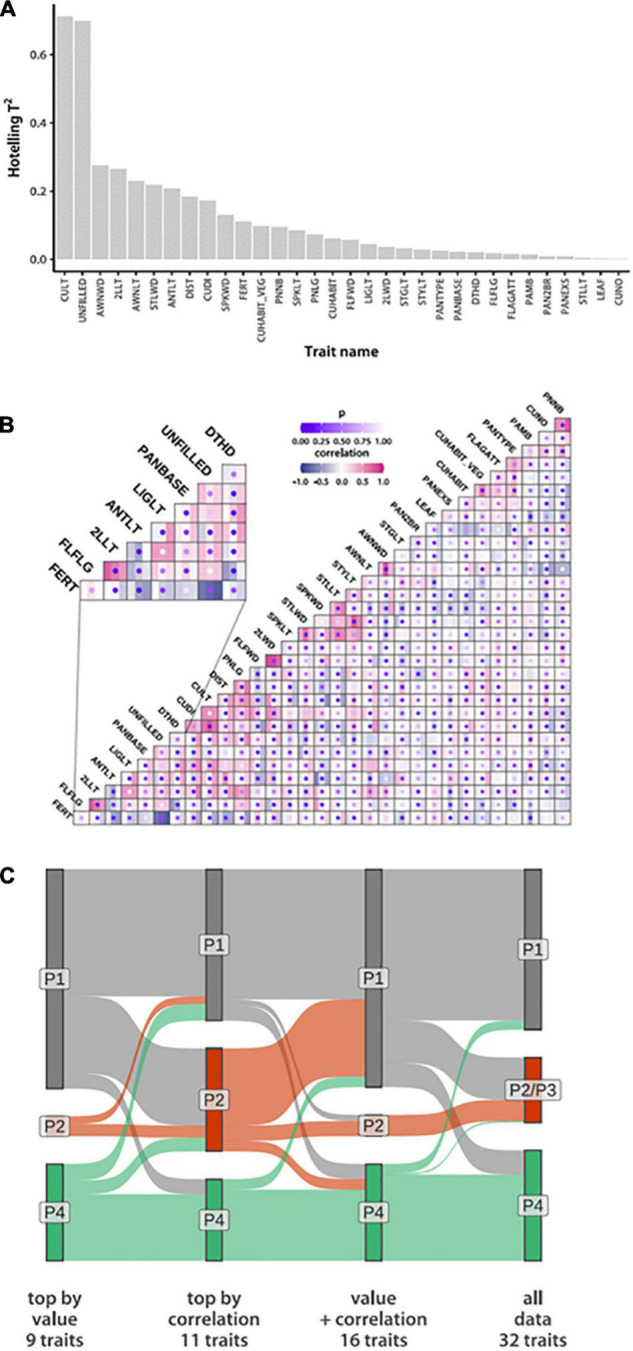
Phenotypic identifiers of group divergence based on the IRRI data. **(A)** Traits sorted by the strength of the association of their values to *N_*G*_* = 4 group assignments based on Hotelling *T*^2^ values **(B)** Among-trait correlation estimates within each phenotypic group. Squares mark trait pairs, with the three bars within each square colored according to correlation magnitude in the three phenotypic groups. Dots represent probabilities of observing the correlation differences by chance (lower probabilities reflect high confidence of correlation difference). The inset shows a segment of the plot in more detail. (Trait acronyms and actual values are in Supplementary Table 7) **(C)** Sankey plot comparing accession membership across groups inferred from trait subsets listed in Table 1.

Having established a robust phenotype-driven grouping of *ORSC* accessions, we next wanted to define a minimal combination of phenotypic traits that is sufficient to reproduce these divisions. Our mixture model allows both phenotype means and covariance to vary among groups. We started by ranking traits according to how well their values correlate with our groups (Figure 2A). We used a multivariate regression with group identity probabilities as the response variable and trait values as predictors. Since the last group membership is fully determined by the other three, only P1 and the combined P2 and P3 membership probabilities were used. We estimated regression coefficients and their covariance and used them to calculate a Hotelling *T*^2^ statistic (Hotelling, 1931) of each trait-group association. This is a multivariate version of the square of Student’s *t* statistic widely used to determine statistical significance of individual regression coefficients. However, in this case we were not interested in the significance of associations. Instead, we ranked traits by their *T*^2^ values (Figure 2A). Two traits, culm length (CULT) and number of empty spikelets per panicle (UNFILLED), stand out for their association with phenotypic groups. Their Hotelling *T*^2^ statistics (0.71 for CULT and 0.70 for UNFILLED) are over twice the value of the next seven highest trait-group associations for which *T*^2^ ranges from 0.17 to 0.28 (Table 1 and Supplementary Table 7). Accessions belonging to the P1 group are on average taller, with P4 the shortest and P2/P3 intermediate. P2/P3 accessions typically have the most empty spikelets per panicle, while P4 accessions have the least (Supplementary Figure 6).

**TABLE 1:** Phenotypic traits identified by trait value (9 traits), correlation switching (11 traits) and the union of both sets of traits (16 traits) based on the analysis of the phenotypic trait data collected at IRRI.

Descriptive trait name	Trait acronym (IRRI)	Top traits by value (9)	Top traits by correlation (11)	Union of value & correlation (16)
Anther length	ANTLT	X	X	X
Awn length	AWNLT	X		X
Awn width	AWNWD	X		X
Culm diameter	CUDI	X	X	X
Culm length	CULT	X	X	X
Days to 50% heading	DTHD		X	X
Distance of nearest spikelet to the panicle base	DIST	X		X
Flag leaf lamina width	FLFWD		X	X
Ligule length	LIGLT		X	X
No. empty spikelets per panicle	UNFILLED	X	X	X
Panicle fertility	FERT		X	X
Panicle length	PNLG		X	X
Penultimate (2nd) leaf length	2LLT	X		X
Spikelet length	SPKLT		X	X
Spikelet width	SPKWD		X	X
Sterile lemma width	STLWD	X		X

*These were used to construct the groups shown in the Sankey plot (Figure 2C).*

To find pairs of traits that change correlations across groups, we first estimated within-group associations and then used a permutation test (randomly assigning accessions to groups) to assess statistical evidence for across-group variation (Figure 2B). The most significant correlation changes occur between the number of empty spikelets (UNFILLED) and either panicle length (PNLG) or culm diameter (CUDI). These correlations are positive (0.54 and 0.48, respectively) only in P4 (Supplementary Table 7).

To test if subsets of traits can reproduce our phenotypic groups, we took 11 traits that belong to pairs that change correlations with probability at least 0.001 (Table 1). This set also includes the top two traits (CULT and UNFILLED) whose values correlate best with group assignments. To expand the trait subset, we added seven phenotypes with *T*^2^ > 0.17. Hotelling values showed a marked drop below this cut-off. These two sets have four traits in common [CULT, UNFILLED, CUDI, and anther length (ANTLT)]. We also used the union of the two sets, containing 16 traits, or half the number in the full data set. Although covariance matrices include both correlations and variances, all traits with appreciable among-group variance changes are already accounted for by using correlation and mean differences (Supplementary Figures 6, 7).

We used three data subsets (nine traits associated with phenotypic groups by value only, 11 that switch correlations, and 16 that were associated either by value or correlation) to re-fit our mixture model, setting the *a priori* number of groups at three. We ran our analyses 20 times and averaged posterior probabilities of accessions belonging to each group. Using all three data sets, we consistently see three groups that separate *O. rufipogon* from *O. nivara*. The nine- and 16-trait data sets assign most P2/P3 accessions to P1 (Figure 2C). The 11 traits most likely to change correlations across groups appear to be sufficient to recapitulate the grouping inferred from the whole data set. The two traits, CULT and UNFILLED, most closely aligned with groups by value and are included in this collection.

Our approach identified a minimally sufficient set of traits that captures most of the phenotypic group structure in the IRRI data. This does not mean that other sets of traits could not achieve similar results but an exhaustive search for all possible combinations that could achieve the same result was not undertaken considering time and the computational expense. Instead, we pursued two approaches using the Cornell and Dale Bumpers data sets to find alternative sets of traits that could be used to re-infer the three major phenotypic groups identified using the full data set. First, we used the process outlined above to identify traits associated with the phenotypic groups that were inferred from IRRI data by value or that change correlations with other traits. Supplementary Figures 8, 9 visualize the Dale Bumpers data analyses and Supplementary Figures 10, 11 visualize the Cornell data analyses with the actual values in Supplementary Table 7. This approach did not identify additional trait subsets that could reliably identify phenotypic groups (Supplementary Figures 12A,C). Second, we identified traits evaluated at Dale Bumpers and Cornell that are the same or similar to the 11 phenotypes (Supplementary Table 3) that defined the grouping patterns in the IRRI data. Re-running our mixture model with these analogous traits reproduced the original groups, albeit at lower resolution (Supplementary Figures 12B,D). Using fewer than 11 traits thus appears to significantly degrade group assignment precision and reproducibility.

### Relationships Between Phenotypic Groups and Genetic Subpopulations

**FIGURE 3:**
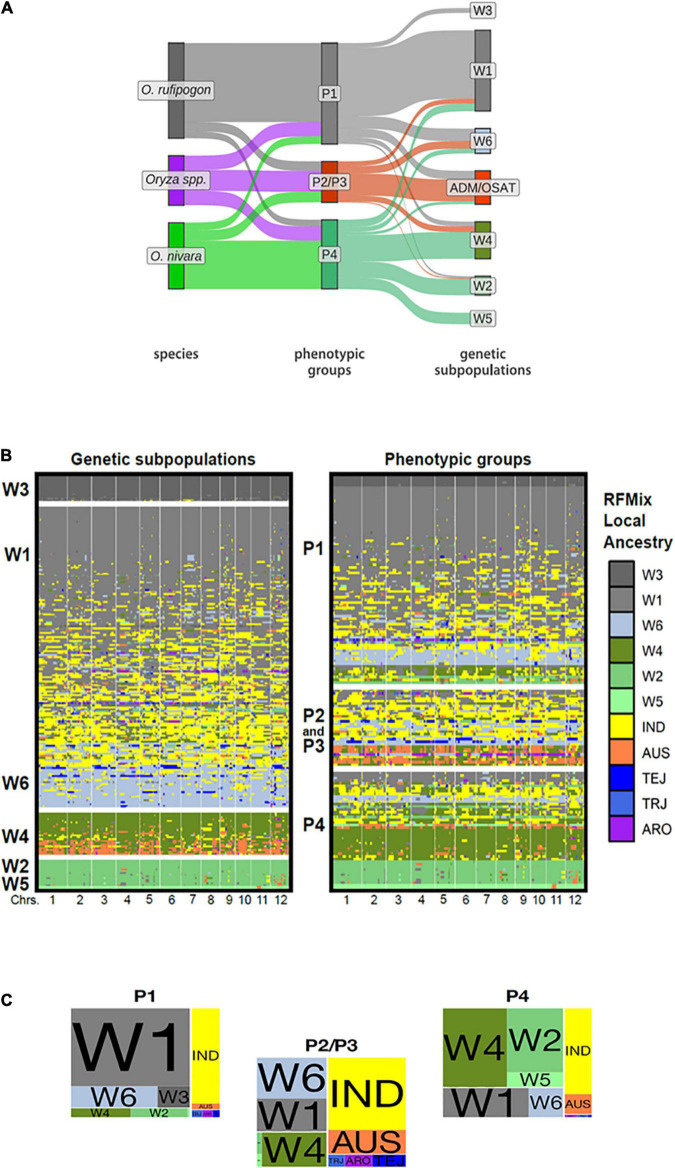
Genotypic composition, local ancestry and phenotypic groups of *ORSC* accessions. **(A)** Sankey plot depicting correspondence between traditional species designations, our phenotypic groups, and Kim et al. (2016) genetic subpopulations using 32 phenotypic traits. (ADM/OSAT are accessions with a high proportion of admixture with *O. sativa*) **(B)** Graphical genotypes display the local ancestry of each *ORSC* individual as determined by RFMix trained on six *ORSC* subpopulations (W1-W6) and five *O. sativa* subpopulations: *indica* (IND), *aus*, (AUS), *temperate japonica* (TEJ), *tropical japonica* (TRJ), and *aromatic* (ARO). The accessions are arranged according to global genotypic ancestry (left) and phenotypic group assignment (right). Each individual’s genotype is represented by two adjoining rows corresponding to phased haplotypes with colors indicating RFMix population assignments of chromosome segments **(C)** Proportion of the *ORSC* subpopulations (left) and *O. sativa* subpopulations (right) within each of the three phenotypic classes.

While our phenotypic groups largely correspond to IRRI-GRIN-Global species designations, we wanted to know how the groups related to previously characterized genotypic subpopulations. The accessions used in this study were genotyped by Kim et al. (2016) who reported six *ORSC* subpopulations (W1-W6). Based on RFMix (Maples et al., 2013) analysis, we identified introgressions among *ORSC* subpopulations and between the *ORSC* and five *O. sativa* subpopulations (IND, AUS, TRJ, TEJ, ARO). We partitioned accessions with more than half of their genome introgressed from *O. sativa* into a separate “admixture with *O. sativa*” group (ADM/OSAT). By means of a Sankey plot, we related the species designation, phenotypic group and genetic subpopulation for the *ORSC* accessions with complete information (Figure 3A and Supplementary Table 2). Across the *ORSC* subpopulations, the majority of the W1, all W3, and about half of the W6 accessions (as per Kim et al., 2016) clustered in the P1 group, taxonomically designated as *O. rufipogon*. Conversely, all of the W5 accessions and most of the W2 clustered in the P4 group, taxonomically designated as *O. nivara*. Finally, accessions with significant *O. sativa* introgression fell almost exclusively into the P2/P3 group and are classified as *Oryza* spp. The P2/P3 group identified using the reduced set of 11 traits also includes most of the W6 subpopulation (Supplementary Figure 13). Eight accessions taxonomically classified as *O. rufipogon* in the IRGC were genetically designated as W2 or W4 by Kim et al. (2016) and clustered with the P4 group in this study. These accessions had less than 20% of their genome from *O. sativa* and were re-classified as *O. nivara* in our analyses (including in the results reported above) and are identified in Supplementary Table 2.

Surveying the individual introgressions across the genome, there were no obvious patterns identifying particular genomic regions underlying the phenotypic group differentiation (Figure 3B). This result was not surprising, given that these groups are distinguished by multiple polygenic traits. We do see that those accessions from the P2/P3 group were more likely to harbor *O. sativa* introgressions (Figures 3B,C) and the median portion of the genome coming from *O. sativa* is also larger in P2/P3 than in P1 or P4 accessions (Supplementary Figures 14B, 15). However, P1 and to a lesser extent P4 harbor a few accessions with significant contributions from *O. sativa* or *ORSC* subpopulations typically associated with a different phenotypic group, suggesting that similar phenotypic syndromes can be achieved through divergent genotypes.

Most *O. sativa* introgressions come from the *indica* subpopulation (Figure 3 and Supplementary Figure 14B). Only accessions belonging to the P2/P3 group show appreciable contributions from *O. sativa japonica*. Introgressions from *aus* are relatively more prevalent in accessions from both P2/P3 and P4.

### Genomic Analysis of Merged *ORSC* Datasets and Geographic Distribution

**FIGURE 4:**
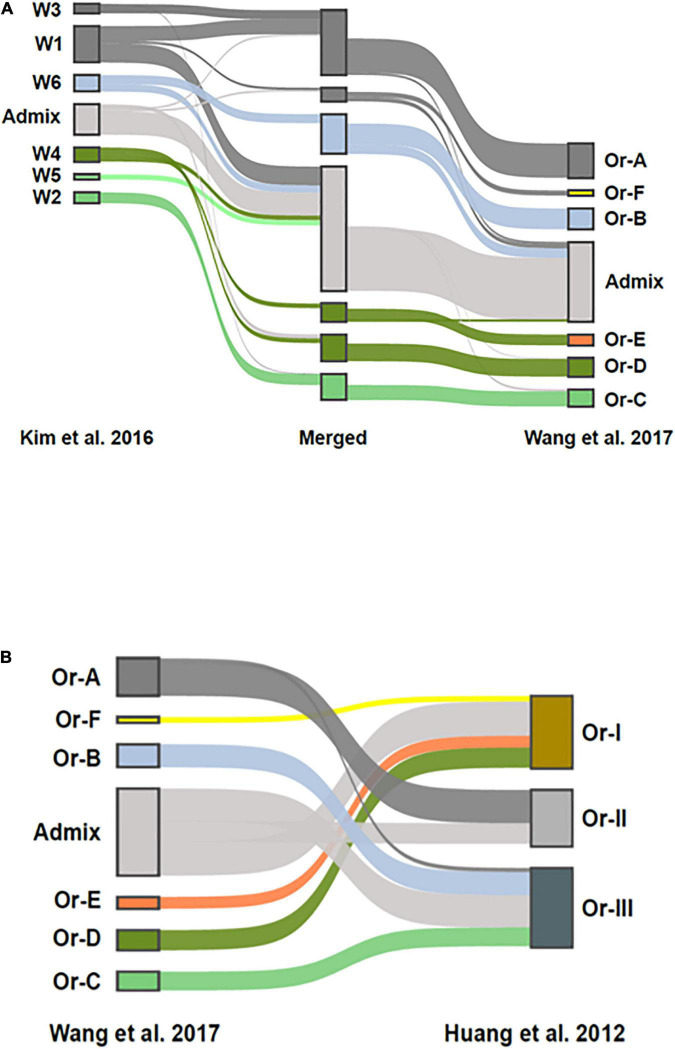
Comparison of *ORSC* genotypic subpopulations reported by Kim et al. (2016) based on 286 *ORSC* accessions to those reported by Wang et al. (2017) and Huang et al. (2012) based on 446 *ORSC* accessions. **(A)** Population structure assignments in a merged SNP dataset consisting of accessions from two *ORSC* collections. On the left side are the accessions from Kim et al. (2016), on the right are accessions from the analysis by Wang et al. (2017). In the center is a merged set with group assignments at *K* = 7. Ribbons connect the group assignments of the merged accessions with their group assignments in the respective *ORSC* collections **(B)** Comparison of the three subpopulations originally identified by Huang et al. (2012) to the six subpopulations and admixed group identified by Wang et al. (2017) when reanalyzing the same genotypic data. The inclusion of *aus*-like (*Or-E*) and *indica*-like (*Or-F*) accessions in *Or-I* and *japonica*-like (*Or-B*) accessions in *Or-III* as reported by Huang et al. (2012) is confirmed.

To compare the population structure of the collection of *ORSC* analyzed by Kim et al. (2016) (*n* = 286) with that generated by Huang et al. (2012) and subsequently analyzed by Wang et al. (2017) (*n* = 435), a merged SNP dataset was constructed and analyzed using fastSTRUCTURE (Raj et al., 2014). The combined dataset contained 55,213 SNPs shared between them. Structure analysis of the merged data resulted in six subpopulations that match nearly perfectly with the six subpopulations reported by Wang et al. (2017), (Figure 4A). The six subpopulations, designated W1 to W6 by Kim et al. (2016) have a clear connection to those designated *Or-A* to *Or-F* by Wang et al. (2017) as illustrated by shared merged groups. The main differences are due to the thresholds used to classify accessions designated as *admixed*, and the different size and composition of the two collections. Where Wang et al. (2017) used a threshold of > 80% ancestry to classify accessions as belonging to one of the wild groups, Kim et al. (2016) used a threshold of >60%. As a result, roughly half of the accessions designated as W1 by Kim et al. (2016) were identified as *admixed* in the combined analysis. It is also noteworthy that Kim et al. (2016) had a smaller proportion of wild accessions from China (W6), and a larger proportion from Papua, New Guinea (W3) and from Nepal (W5), which impacted the subpopulation structure that emerged in the analysis. Nonetheless, there is a clear correspondence between genetic groups in the two studies, with W1 and W3 (predominantly *O. rufipogon* as described by Kim et al., 2016) corresponding to *Or-A* (Wang et al., 2017), W6 (Chinese *O. rufipogon*) to *Or-B*, and W2 (predominantly *O. nivara*) to *Or-C*. *W4* (*aus*-like wild ancestor as described by Kim et al., 2016) is split between *Or-D* (wild) and *Or-E* (feral), and interestingly, the accessions clustered as *Or-E* by Wang et al. (2017) were recognized as a separate group by Kim et al. (2016) when a higher *K* value (*K* = 8) was used in the analysis.

The groups identified by Wang et al. (2017) also show reasonable correspondence to the original three groups proposed by Huang et al. (2012), (Figure 4B), though Huang et al. (2012) did not explicitly call out the large proportion of *ORSC* accessions that are highly admixed with *O. sativa* as both Wang et al. (2017) and Kim et al. (2016) did. Given the different compositions of the two germplasm collections, this merged genomic analysis allows us to interpret our findings about the three phenotypic groups in a larger context.

There is support for geographic subpopulation structure whereby accessions collected from the Malay Archipelago (Malaysia, Indonesia, Philippines, Papua New Guinea), which represents the southeastern extreme of the geographic distribution of the *ORSC*, are phenotypically classified as P1 in this study; they are also predominantly classified as *O. rufipogon* in the IRGC (Supplementary Figure 16). Accessions from India, Nepal, and Sri Lanka, representing the northwestern extreme of the geographic distribution, are predominantly classified as P4 in this study and *O. nivara* in the IRGC. Accessions collected from China were genetically classified as *O. rufipogon* with substantial admixture from *O. sativa* (Kim et al., 2016), but they were phenotypically more similar to annual *O. nivara* in this study. Accessions collected across the coastal regions of South and Southeast Asia are recognized phenotypically as P2/P3 and are typically the most highly admixed with *O. sativa*. A great diversity of phenotypic variation is found in mainland Southeast Asia where *ORSC* accessions manifesting both P1 and P4 phenotypic syndromes co-exist, often sympatrically or in contiguous environments. These geographical distributions of *O. rufipogon* and *O. nivara* are clearly shown in Supplementary Figure 16B where only the locations of the “core” accessions, those for which the species, phenotypic group and genotypic subpopulation agreed, are shown. The fact that cultivated forms of rice, predominantly representing the *indica* subpopulation, overlap with wild rice habitats in many parts of Asia creates endless possibilities for cross fertilization and the emergence of novel phenotypes. In addition to their inherent value and interest, these naturally occurring interspecific populations represent valuable, ecologically viable pools of variation for plant breeders interested in identifying novel forms of climate resilience for crop improvement.

## Discussion

### Trait Significance

The traits measured in this study emphasize highly heritable, easily measured morphological and physiological traits that can be readily observed in populations of crop wild relatives grown under controlled conditions. Many of the same traits are evaluated by genebanks and widely used for identification, tracking and management of *ex situ* germplasm collections. Of the traits measured here, four were significantly associated with phenotypic groups and with life history; these included days to heading, plant height, percent filled spikelets per panicle (seeds/panicle), and spikelet/seed size (related to grain weight). These traits provided information that was valuable for differentiating annual and perennial life habits and were similar to traits evaluated by Grillo et al. (2009) in two *O. rufipogon*/*O. nivara* mapping populations. We also measured several traits that were potentially informative about mating habit, including anther length, style length and stigma length, but only anther length discriminated the groups based on both Hotelling *T*^2^ value and correlation estimates (Table 1). These traits related to life history and mating habit support our understanding of the biological significance of our phenotypic groups.

### Traits That Differentiate Phenotypic Groups

The single most informative trait for distinguishing *ORSC* phenotypic groups based on the Hotelling *T*^2^ values across all three datasets was seed width (HULGRWD) (*T*^2^ value = 1.15) measured at Dale Bumpers, which served as a proxy for spikelet width (SPKWD) measured at IRRI, and seed volume, (*T*^2^ value = 1.70) calculated from seed width and length. Spikelet width and seed width were highly correlated (*r* = 0.62) and both width and volume were informative for distinguishing groups. Seed traits were not measured at Cornell and therefore could not be used for further confirmation.

Comparing this analysis to the principle component analysis (PCA) of phenotypic data collected at IRRI on a different set of 116 *ORSC* accessions, Banaticla-Hilario et al. (2013a) also reported SPKWD as one of the most important characters separating *O. nivara* and *O. rufipogon* accessions. The other important traits included anther length (ANTLT), days to heading (DTHD) and culm length (CULT). Of note, spikelet fertility was not included in the PCA because it was highly correlated with anther length and spikelet width, leading to the conclusion that anther length and spikelet width serve as a proxy for fertility when plants are grown under controlled screenhouse conditions. Our data on DTHD collected at IRRI differentiated P1 from the other groups. The trend is the same in the Cornell and the Dale Bumpers data, but with more overlap between groups. This may be a consequence of evaluating DTHD during the short days of fall at higher latitudes. Across our three environments, unfilled grain number (UNFILGRNB) in the IRRI and Dale Bumpers data and seed weight (Seed_S2) collected from bagged and unbagged panicles at Cornell provided useful proxies for spikelet fertility. Lastly, culm length (CULT) which was measured from the soil surface to the panicle base at IRRI and plant height (PTHT) measured from the soil surface to the tip (end) of the panicle at Dale Bumpers and Cornell were prominent differentiators across all three studies. This comparison confirms that many traits distinguishing the phenotypic groups can be identified across different environments and panels of *ORSC* accessions. These observations are confirmed by earlier studies summarized in Supplementary Table 1 in which *O. nivara* was characterized by having wider seeds, higher seed production (fertility), shorter culm length (plant height), and earlier flowering (due to photoperiod insensitivity) compared to *O. rufipogon* which was characterized by relatively narrow seeds, low seed production, longer culm length (taller plant) and later flowering (due to photoperiod sensitivity) (Morishima et al., 1961; Oka and Morishima, 1967; Barbier, 1989a).

Considering the high information content of SPKWD (spikelet/seed width) as a distinguishing feature of the phenotypic groups identified in this study and its utility for differentiating the *O. rufipogon* and *O. nivara* species, we recommend including seed width and length in the suite of traits collected on *ORSC* accessions in genebanks around the world. With the availability of scanners and appropriate software, this would be an inexpensive investment that would help improve the classification of *ORSC* accessions, making collections of these crop wild relatives more valuable to users.

### “Core” *Oryza rufipogon* and *Oryza nivara* Accessions and Admixture With *Oryza sativa*

Of the *ORSC* accessions phenotyped in this study, approximately 41% (∼98 accessions, including the 49 extracted for use as training sets) had less than 5% introgression from *O. sativa* and can be considered non-admixed and wild. Most of these (*n* = 91) clustered in either P1 (*n* = 48) where most were classified as *O. rufipogon* or in P4 (*n* = 43) where most were classified as *O. nivara*, while a few (*n* = 7) clustered in P2/P3.

Based on analyses of both phenotypic and genotypic data using different model parameters, we identified 42 “core” accessions that are consistently classified as perennial *O. rufipogon* and 25 “core” accessions consistently classified as annual *O. nivara*. These “core” accessions all have <20% admixture from *O. sativa* and intersect with the group carrying <5% *O. sativa* introgression. This collection of 67 accessions provides a useful subset of *ORSC* materials for studying the genetic basis of the phenotypic divergence that distinguishes the two ecotypes of wild rice in Asia.

*ORSC* accessions that are highly admixed with *O. sativa* are also of interest. In this study, 25% of accessions were classified as admixed because they had >40% of the genome introgressed from *O. sativa.* In the previous study by Wang et al. (2017) using a different panel of *ORSC* accessions and a different threshold for determining admixture, 42% of wild rice samples were reported to be substantially admixed with >20% of the genome showing introgression from *O. sativa.* In both studies, *indica* and *aus* introgressions accounted for the largest proportion, and *japonica* introgressions were rare. Admixed accessions found in P1 and P4 in this study had an average of 19.8% and 19.0% of their genomes comprised of introgressions from *O. sativa*, respectively, while levels of admixture in P2/P3 averaged 52.6%; indeed, two P2/P3 accessions carried ∼85% *O. sativa* DNA (Figure 3C and Supplementary Figure 15). Nonetheless, the genome-wide level of admixture alone, is not a good predictor of phenotype, given that 13 accessions classified phenotypically into P1 carry > 40% *O. sativa* DNA, and 9 accessions classified phenotypically as P4 carry > 40% *O. sativa* DNA. We therefore infer that it is the particular distribution of *O. sativa* introgressions across the genome and the subpopulation ancestry of each introgression, in combination with the *ORSC* genetic background that determines the phenotypic outcome. Our evaluation of this *ORSC* collection provides the foundation for future research to examine the genotype-phenotype relationships for specific traits of interest.

### SINE-Codes and Chloroplast Markers as Predictors of Genotypic, Species and Phenotypic Groups

In rice, SINE codes have been associated with life history (annual, intermediate or perennial growth habit) and found useful for distinguishing *O. nivara* from *O. rufipogon* (Motohashi et al., 1997; Cheng et al., 2003; Chen et al., 2004; Xu et al., 2007). Kim (2016) developed a SINE code and used it to classify most of the *ORSC* accessions used in this study (Supplementary Table 2). Using the SINE code, 74.5% of the accessions classified as P1 were identified as perennial (compared to 72.5% of accessions classified as *O. rufipogon*), and 62.5% of accessions classified as P4 were identified as annual or intermediate (compared to 61.7% classified as *O. nivara*). In P2/P3, 47.4% of accessions were classified as perennial and 52.6% as annual or intermediate using the SINE code (compared to 23.3% classified as *O. rufipogon*, 25.6% as *O. nivara* in P2/P3 with the remainder identified as *Oryza* spp.). Thus, the SINE-based classifications approximated the species designations in these groups, though they were not entirely concordant.

Kim et al. (2016) further analyzed 286 *ORSC* accessions for variation at 25 polymorphic sites in the chloroplast genome and identified unique chloroplast haplotypes associated with three wild subpopulation groups located at the geographical extremes of the species range. These may be of interest for investigating refuge populations of *ORSC* and elucidating phylogenetic relationships among wild populations of *Oryza* but chloroplast haplotype analysis alone was not robust enough to be useful as a predictor of genetic subpopulation, life habit or species in the *ORSC*. Nevertheless, the chloroplast data provided clear evidence of gene flow between annual and perennial populations, as well as between *ORSC* and *O. sativa* populations, and confirmed that it occurs through both pollen dissemination and seed dispersal (likely facilitated by human migration). Taken together, these data suggest that the population structure of the *ORSC* is the result of complex evolutionary pathways that intersect and loop back upon each other due to highly permeable ‘species’ boundaries and, as documented in this study, highly plastic phenotypic variation. Information about SINE-code and chloroplast haplotype variation (as determined by Kim et al., 2016) corresponding to each accession is summarized in Supplementary Table 2. These features, along with geographical and ecological information about where each accession was collected, disease and insect resistance, grain quality and additional use-data provided by the IRGC database add value to these genetic resources and enhance our understanding of where they come from and how they might be used in the future.

### Emergent Phenotypes

The P2/P3 group is characterized by 43 accessions with trait combinations that form a coherent group, despite the fact that they do not match either of the *a priori* taxonomic species descriptions. This group is comprised of 29 (67%) admixed *ORSC* accessions with > 40% of the genome derived from *O. sativa* but it also contains accessions classified as annual *O. nivara* or perennial *O. rufipogon*. The fact that the P2/P3 accessions were collected in diverse ecological and geographic regions, especially from coastal regions of China, Southeast and South Asia (Supplementary Figure 16), and yet share a common suite of emergent phenotypes suggests that particular combinations of traits may evolve repeatedly and independently when annual, perennial and intermediate forms of common wild rice come together as sympatric swarms in unsupervised settings. The patterns of admixture observed in this group suggest that diverse *ORSC* populations have hybridized among themselves and with local populations of *O. sativa*, giving rise to forms of trait variation not observed in the parental populations. In rice, several lines of evidence suggest that annual and intermediate forms have emerged repeatedly from genetically and geographically diverse populations of perennial ancestors in response to changing patterns of temperature, rainfall and CO_2_ (Grillo et al., 2009; Banaticla-Hilario et al., 2013a,b; Liu et al., 2015). The *ORSC* populations examined here provide opportunities to deepen our understanding of incipient speciation and to identify mechanisms by which annual life forms may evolve from perennial ancestors in response to changes in the environment. They also provide material for examining what kinds of selection pressure(s) are associated with a shift toward the annual, early-flowering, seed-bearing habit or conversely, serve to maintain the perennial, stoloniferous, late-flowering and vegetatively vigorous habit. During times of climate change, a strategy of balancing selection to maintain components of annual, intermediate and perennial life forms may be favored by evolution to allow for the emergence of new resilience mechanisms. We have a lot to learn from the existence of these dynamic *ORSC* populations. They have survived many waves of climate change in the past and are likely to hold secrets about survival strategies for the future.

## Data Availability Statement

The original contributions presented in this study are included in the article/Supplementary Material, further inquiries can be directed to the corresponding author/s.

## Author Contributions

KM, MEN, MB-H, GCE, HJK, JJ, SH, JK, and LH phenotyped the plants. YS managed the data. AG, JE, GCE, HJK, JJ, and SRM analyzed the data. GCE, AG, JE, and SRM wrote the manuscript. SRM conceptualized the project. All authors contributed to the article and approved the submitted version.

## Conflict of Interest

AG is employed by Bayesic Research LLC. The remaining authors declare that the research was conducted in the absence of any commercial or financial relationships that could be construed as a potential conflict of interest.

## Publisher’s Note

All claims expressed in this article are solely those of the authors and do not necessarily represent those of their affiliated organizations, or those of the publisher, the editors and the reviewers. Any product that may be evaluated in this article, or claim that may be made by its manufacturer, is not guaranteed or endorsed by the publisher.

## Footnotes

1
https://gringlobal.irri.org/gringlobal/search

2
https://shigen.nig.ac.jp/rice/oryzabase/strain/wildCore/list

3
https://github.com/tonymugen/MuGen

4
https://github.com/tonymugen/MuGaMix

5
https://github.com/davidsjoberg/ggsankey
