# Supplementary material for: Phenotypic Variation and the Impact of Admixture in the Oryza rufipogon Species Complex (ORSC)
Source: Front Plant Sci. 2022 Jun 13;13:787703. doi: 10.3389/fpls.2022.787703 (PMC9235872; doi:10.3389/fpls.2022.787703)
Supplement: Supplementary file 1 [file Table_1.pdf]

Supplementary Table 1: Summary of *O. rufipogon, O. nivara* and *O. sativa* habitat preferences, geographic range, and morphological traits. [Edited from Jung (2016) and Kim (2016)]

Trait	*Oryza* species		
	O. rufipogon	***O. nivara***	***O. sativa***
Life habit[1]	Perennial	Annual	Annual
Habitat[2]	Deepwater/aquatic, swamps/marshes, rice fields, moderately deep water, prefers clay/loam soil and black soil	Seasonally dry, swamps, pond/stream/field banks	Dry and wetland fields, deep water up to 4m, floating >4m
Geographic range[2]	Tropical Asia to Australia[2]	Drier regions of S/SE Asia	Worldwide
Photoperiod response	Sensitive	Usually insensitive	Sensitive
Plant type[2]	Decumbent or floating, tufted and spreading/scrambling	Semi-erect to decumbent	
Lateral meristem formation/nodal tillering	Present		Absent
Horizontal stems	Present	Absent	Absent
Regeneration ability of stem segments[6,7]	Mod-high; mod-high (1.51-2.50 ave. in 0-3 scale)	Mostly low; low (0.07-0.33 ave. in 0-3 scale)	Low-moderatelyhigh
Plant height	Tall, ~150cm ave.[5]; culm length: 234-293cm[7]	Short to intermediate(usu. <2m)[2]; short (ave. 84 cm)[5]; Culm length: 127-151.7cm[7]	
Internodes	Long		
Ligule	2.07 cm ave. length[5]	1.19 cm ave. length[5]	1.71 cm ave. length[5]
Characteristics at end of growing season	Dried productive tillers, green tillers present that will flower next season	All tillers are productive, all dried	
Stolons	Sometimes	None	None
Roots	Perennial root stock, adventitious roots		

**Trait**	***Oryza* species**		
	O. rufipogon	***O. nivara***	***O. sativa***
Days to heading[7]	Longer (137-146 d ave.)	Shorter (112-145 d ave.)	
Panicle number[7]	Lower (3.3-8.5 ave.)	Higher (10.4-14.5 ave.)	
Panicle length[5]	Ave. 21.3cm	Ave. 13.3cm	Ave. 21.8cm
Panicle exsertion	Well exserted	Inserted or not well exserted/ partially exserted	
Panicle shape	Spreading, open	Semi-open	Erect, compact
Panicle branching[5]	Ave. 7.2 primary branches/panicle	Few secondary and tertiary branches; ave.5.06 primary branches/panicle	Secondary branching; ave. 10.2 primary branches/panicle
Spikelet dimensions	Usually 8-9 mm L[2]; ave. 8.13 mm L, 2.27mm W[5]	Large - 6-8.4mm L, 1.9-3.0 mm W, 1.2-2.0 mm thick, ave.[2]; 8.14L, 2.56 W ave.[5]	Usually 4-8.5mm L,2-4 mm W[2] ; ave. 8.03mm L,3.05mm W[5]
Spikelets/panicle	Ave. 63.45	Ave. 39.35	Ave. 113.95
Spikelet fertility	May be low	High	
Time between spikelet opening and pollenemission[6]	Longer: ~2-6 min	Short: ~1-2 min	Short: immediately-30 sec
Awns	Long - 5-11cm [2]; ave. 5.87[5]	Long/strong (4-10cm)[2]; 6.91 cm ave. [5]	Short-none[2]; ave. 0.72cm[5]
Anthers	>3mm L to >7; indehiscent,pendant; 4.88 mm ave. L[5]; 4.79-5.07 cm ave.[7]	<2.5mm; immd. dehiscent, upright; 2.82 mm ave. L[5]; 2.10-2.21 cm ave.[7]	Usually <2.1 mm L[2]; 2.51 mm ave.[5]
Embryo size	Usually 1-1.5 mm long	Usually 1-1.5 mm long	Usually <2.1 mm long
Synchronicity of seed maturation	Asynchronous	Asynchronous	Synchronous
Shattering[2]	Highly shattering	Highly shattering	Non-shattering
Seed production	Low	High	High
Seed dormancy	Mod-mod high [6]	Mod-high[6], strong[2]	Low[6]

[1] Vaughan DA, Morishima H, Kadowaki K (2003). Diversity in the *Oryza* genus. Current Opinion in Plant Biology 6 139–146.

[2] Vaughan DA (1994) The Wild Relatives of Rice: A Genetic Resources Handbook, IRRI, Philippines

[3] Grillo MA, Li C, Fowlkes AM, Briggeman TM, Zhou A, Schemske DW, Sang T (2009) Genetic architecture for the adaptive origin of annual wild rice, *O. nivara.* Evolution 63:870-883.

(Individuals in studychosen based on characteristics displayed under greenhouse growing conditions.)

[4] Li C, Zhou A, Sang T (2005) Genetic analysis of rice domestication syndrome with the wild annual species, *O. nivara*. New Phytologist 170:185-193.

[5] Morishima H, Oka HI, Chang WT (1961) Directions of differentiation in populations of wild rice, *Oryza perennis* and *O. sativa f. spontanea.* Evolution 15: 326-339.

O. perennis traits entered here as *O. rufipogon* and *O. sativa f. spontanea* as *O. nivara*

[6] Oka HI, Morishima H (1967) Variations in the breeding system of a wild rice *O. perennis.* Evolution 21:249-258.

O. perennis (Asian race); *perennis* type entered as *O. rufipogon* and *O. perennis* Asian race, *spontanea* type entered as *O. nivara*

[7] Barbier P (1989a) Genetic variation and ecotypic differentiation in the wild rice species *Oryza rufipogon*. I. Population differentiation in life-history traits and isozymic loci. Japanese Journal Genetics 64:259-271.
