# Supplementary material for: Phenotypic Variation and the Impact of Admixture in the Oryza rufipogon Species Complex (ORSC)
Source: Front Plant Sci. 2022 Jun 13;13:787703. doi: 10.3389/fpls.2022.787703 (PMC9235872; doi:10.3389/fpls.2022.787703)
Supplement: Supplementary file 8 [file Data_Sheet_1.ZIP › mixtureModel.pdf]

The data analysis pipeline for the mixture model

Anthony J. Greenberg

April 11, 2022

```
R version 4.1.2 (2021-11-01)
Platform: x86_64-pc-linux-gnu (64-bit)
Running under: Arch Linux

Matrix products: default
BLAS/LAPACK: /opt/intel/mkl/lib/intel64/libmkl_gf_lp64.so

locale:
 [1] LC_CTYPE=en_US.UTF-8       LC_NUMERIC=C               LC_TIME=en_US.UTF-8
 [4] LC_COLLATE=en_US.UTF-8     LC_MONETARY=en_US.UTF-8    LC_MESSAGES=en_US.UTF-8
 [7] LC_PAPER=en_US.UTF-8       LC_NAME=C                  LC_ADDRESS=C
[10] LC_TELEPHONE=C             LC_MEASUREMENT=en_US.UTF-8 LC_IDENTIFICATION=C

attached base packages:
[1] stats     graphics  grDevices utils     datasets  methods   base

other attached packages:
 [1] ggspatial_1.1.5         rnaturalearthdata_0.1.0 rnaturalearth_0.1.0
 [4] sf_1.0-5                dplyr_1.0.7             ggsankey_0.0.99999
 [7] MuGaMix_1.0             showtext_0.9-4          showtextdb_3.0
[10] sysfonts_0.8.5          ggplot2_3.3.5           data.table_1.14.2

loaded via a namespace (and not attached):
 [1] Rcpp_1.0.7         pillar_1.6.4       compiler_4.1.2     class_7.3-19
 [5] tools_4.1.2        lattice_0.20-45    lifecycle_1.0.1    tibble_3.1.6
 [9] gtable_0.3.0       pkgconfig_2.0.3    rlang_0.4.12       DBI_1.1.1
[13] e1071_1.7-9        withr_2.4.2        generics_0.1.1     vctrs_0.3.8
[17] classInt_0.4-3     grid_4.1.2         tidyselect_1.1.1   glue_1.5.0
[21] R6_2.5.1           fansi_0.5.0        sp_1.4-6           purrr_0.3.4
[25] magrittr_2.0.1     scales_1.1.1       ellipsis_0.3.2     units_0.7-2
[29] colorspace_2.0-2   utf8_1.2.2         KernSmooth_2.23-20 proxy_0.4-26
[33] munsell_0.5.0      crayon_1.4.2
```

1 All traits, IRRI data

This document contains the full data analysis pipeline we used to run multi-trait mixture models and analyze the results.

Read the data and establish constants.

```
> phenoAll   <- fread("./LNmodeIRRI.tsv")
> trtNames   <- fread("./traitNames.tsv")
> trtNames   <- trtNames[!is.na(IRRI) & (binary == 0),
+                  c("binary", "Planteome", "IRRI" )]
> trtNamesNB <- trtNames[IRRI %in% names(phenoAll)[-1], IRRI]
> trtNamesNB
```

```
 [1] "DTHD"      "CUNO"      "FLFLG"     "PNLG"      "PNNB"        "CULT"
 [7] "FLFWD"     "PANBASE"   "UNFILLED"  "2LLT"      "2LWD"        "ANTLT"
[13] "AWNLT"     "AWNWD"     "CUDI"      "CUHABIT"   "CUHABIT_VEG" "DIST"
[19] "FERT"      "FLAGATT"   "LEAF"      "LIGLT"     "PAMB"        "PAN2BR"
[25] "PANEXS"    "PANTYPE"   "SPKLT"     "SPKWD"     "STGLT"       "STLLT"
[31] "STLWD"     "STYLT"
```

```
> d          <- length(trtNamesNB)
> colSubset  <- c("NSFTV_ID", trtNamesNB)
> phenoAll   <- phenoAll[, ..colSubset]
> N          <- phenoAll[, .N]
> d
```

```
[1] 32
```

```
> N
```

```
[1] 222
```

Mahalanobis distance function to rank traits. The output of this function is used to calculate Hotelling's T, a multivariate version of Student's t statistic.

```
> mhl <- function(vec, S){
+     vec%*%S%*%matrix(vec, nrow = length(vec), ncol = 1)
+ }
```

Extracting the number of groups with greater than specified effective number of accessions.

```
> eachNZgrp <- function(vbObj, accNum){
+     sum(vbObj$effNm >= accNum)
+ }
> getNZgrp <- function(vbList, minEffNum){
+     sapply(vbList, eachNZgrp, minEffNum)
+ }
```

Set colors for groups and populations.

```
> wPopColors  <- c("W1" = "grey60", "W1orI" = "grey65",
+                  "W2" = "palegreen3", "W3" = "grey90",
+                  "W4" = "olivedrab4", "W5" = "palegreen", "W6" = "grey30",
```

```
+                  "W7" = "grey45", "W8" = "palegreen2")
> sppColors   <- c("O. rufipogon" = "grey40", "O. nivara" = "green3",
+                  "Oryza spp." = "purple")
> pGrp8Colors <- c("P1" = "grey50", "P2" = "orangered3", "P4" = "mediumseagreen",
+                  "P3" = "skyblue4", "P5" = "thistle2", "P6" = "lightcoral",
+                  "P7" = "tan", "P8" = "yellow3", "P2/P3" = "orangered3",
+                  "O. rufipogon" = "grey40", "O. nivara" = "green3",
+                  "Oryza spp." = "purple")
> sativaCols  <- c("ARO" = "purple", "AUS" = "sienna1", "IND" = "yellow",
+                      "TRJ" = "royalblue", "TEJ" = "blue")
> pPop3Colors <- c("P1" = "grey50", "P2" = "skyblue4", "P3" = "orangered3")
```

Now run the model at $P = 4$ with the three subsets of traits (Mahalanobis only, correlation variation only, and the union of the two).

```
> alpha0  <- 1e-3
> nVBreps <- 200
> nReps   <- 5
> Ysc     <- phenoAll[, lapply(.SD, scale), .SDcols = trtNamesNB]
> names(Ysc) <- trtNamesNB
> if (file.exists("ordIRRI.Rdata")) {
+     load(file = "ordIRRI.Rdata")
+ } else {
+     ordIRRI <- vector("list", 14)
+     for (iGrp in 2:15) {
+         ordIRRI[[iGrp - 1]] <- replicate(nReps,
+                           MuGaMix::quickFitModel(Ysc, trtNamesNB, iGrp, alpha0, nVBreps),
+                           simplify = FALSE)
+     }
+     save(ordIRRI, file = "ordIRRI.Rdata")
+ }
```

Plot number of estimated groups.

```
> gt2GrpDT <- data.table(fullGrp = c(sapply(ordIRRI, getNZgrp, 2)),
+                           nGrps = rep(2:15, each = nReps))
> pdfFlNam <- "gt2GrpIRRI.pdf"
> showtext_auto()
> ggplot(data = gt2GrpDT, aes(x = nGrps, y = fullGrp)) +
+     geom_point() +
+     theme_classic(base_size = 18, base_family = "myriad") +
+     xlab("Number of groups") + ylab("Number with > 2 lines")
> ggsave(pdfFlNam, width = 5, height = 5, units = "in",
+             device = "pdf", useDingbats = FALSE)
> cat("\\includegraphics{", pdfFlNam, "}\n\n", sep = "")
```

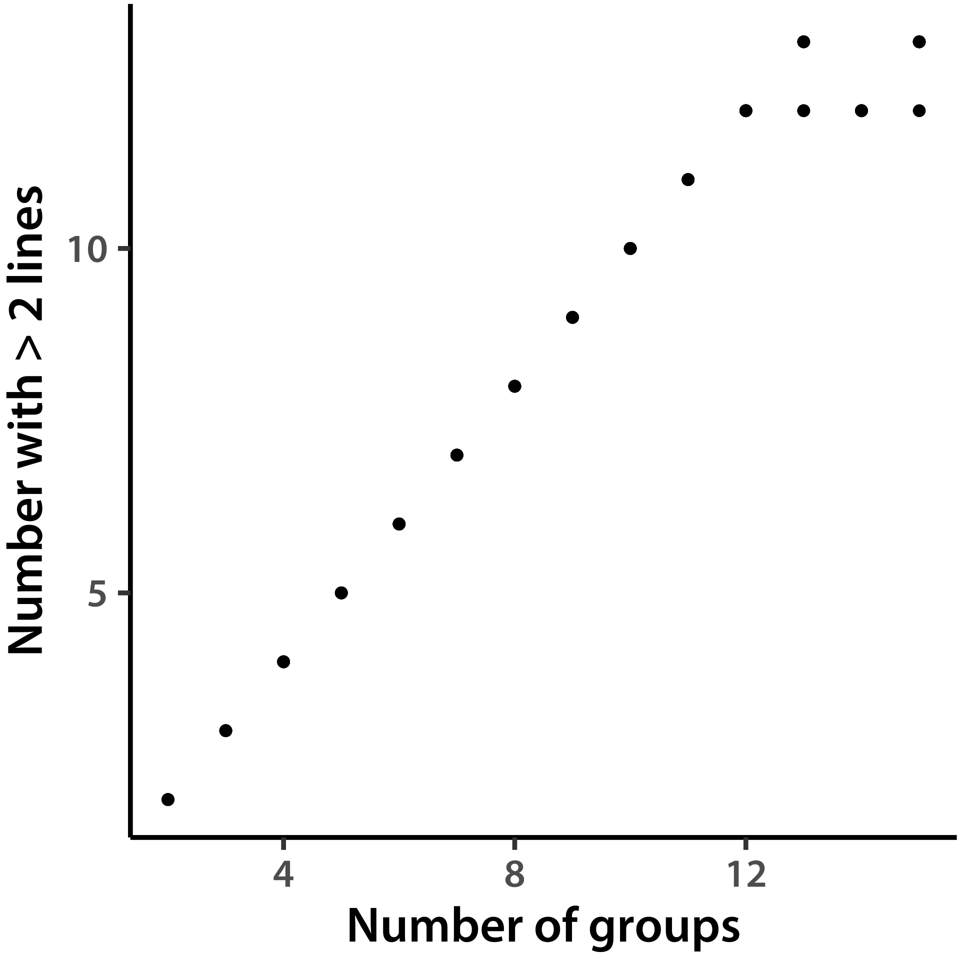

Extract DIC values.

```
> eachDIC <- function(vbObj){
+     vbObj$DIC
+ }
> getDIC <- function(vbList){
+     sapply(vbList, eachDIC)
+ }
> dicVec <- c(sapply(ordIRRI, getDIC))
> dicDT  <- data.table(DIC = dicVec, nGrps = rep(2:15, each = nReps))
```

Plot the DIC distributions.

```
> pdfFlNam <- "dicIRRI.pdf"
> showtext_auto()
> ggplot(data = dicDT, aes(x = nGrps, y = DIC)) +
+     geom_point() +
+     geom_smooth() +
```

```
+     theme_classic(base_size=18, base_family="myriad") +
+     xlab("Number of groups")
> ggsave(pdfFlNam, width=6, height=5, units="in", device="pdf", useDingbats=FALSE)
> cat("\\includegraphics{", pdfFlNam, "}\n\n", sep="")
```

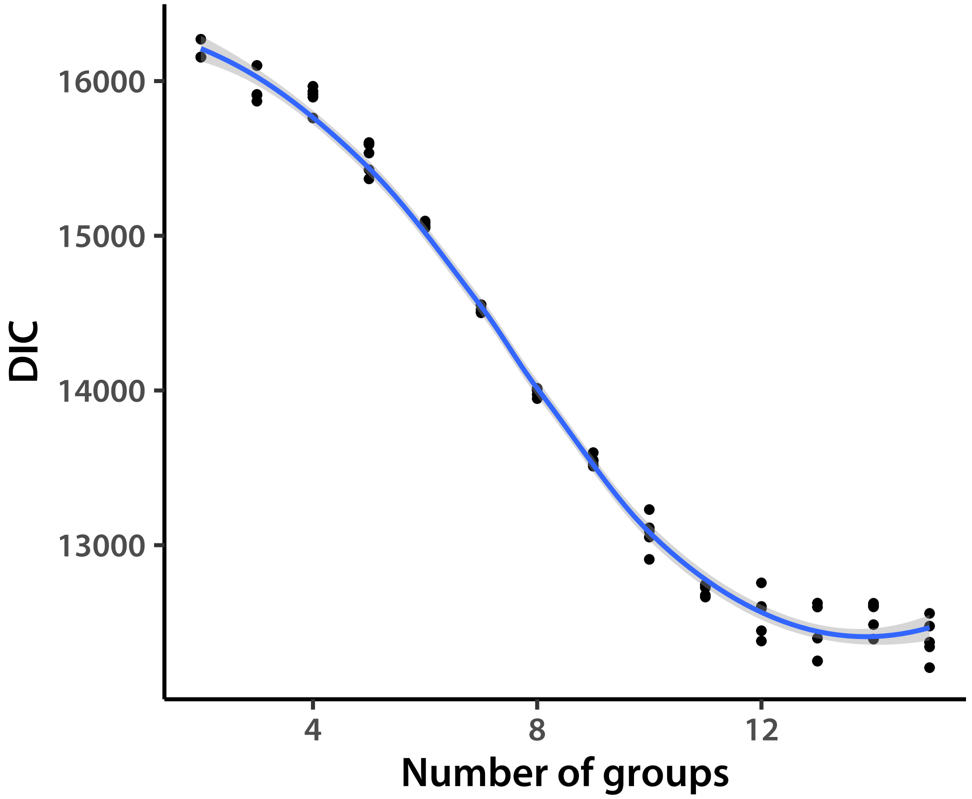

Average p-values from the replicate runs.

```
> jacDist <- function(vec1, vec2, ctVal){
+     v1 <- vec1 >= ctVal
+     v2 <- vec2 >= ctVal
+     res <- sum(v1 & v2)/sum(v1 | v2)
+     if (is.nan(res)) {
+         return(0.0)
+     } else {
+         return(res)
+     }
+ }
> bestCorCol <- function(mat2, vec, ctVal){
+     apply(mat2, 2, jacDist, vec, ctVal)
+ }
> bestColLst <- function(ind, pList, indList, vec, ctVal){
+     bestCorCol(pList[[ind]]$p[, indList[[ind]]], vec, ctVal)
```

```
+ }
> addCol <- function(repInd, grpInd, Ngrp){
+     pMat[, grpInd] <<- (pMat[, grpInd] +
+             ordIRRIp4[[repInd + 1]]$p[,
+                                 indList[[repInd]][bestInd[repInd]]])
+     indList[[repInd]] <<- indList[[repInd]][-bestInd[repInd]]
+     return(NULL)
+ }
> normalizeP <- function(pVec){
+     pVec/sum(pVec)
+ }
> renameGroups <- function(vec){
+     c("P1", "P2", "P4")[which.max(vec)]
+ }
> renameGroups23 <- function(vec){
+     c("P1", "P2/P3", "P4")[which.max(vec)]
+ }
```

Re-run the $N_G = 4$ model and average among runs.

```
> nReps <- 15
> Ngrp  <- 4
> if (file.exists("ordIRRIP4.Rdata")) {
+     load(file = "ordIRRIP4.Rdata")
+ } else {
+     ordIRRIp4 <- replicate(nReps,
+                     MuGaMix::quickFitModel(Ysc, trtNamesNB, Ngrp, alpha0, nVBreps),
+                     simplify = FALSE)
+     save(ordIRRIp4, file = "ordIRRIP4.Rdata")
+ }
```

Plot raw grouping relationships.

```
> rawGrp <- data.table(run1 = paste0("P", apply(ordIRRIp4[[1]]$p,
+                                       1, which.max)),
+                     run2 = paste0("P", apply(ordIRRIp4[[2]]$p,
+                                       1, which.max)),
+                     run3 = paste0("P", apply(ordIRRIp4[[3]]$p,
+                                       1, which.max)),
+                     run4 = paste0("P", apply(ordIRRIp4[[4]]$p,
+                                       1, which.max)),
+                     run5 = paste0("P", apply(ordIRRIp4[[5]]$p,
+                                       1, which.max)))
> pGrpSan  <- as.data.table(ggsankey::make_long(rawGrp, run1, run2,
+                                       run3, run4, run5))
```

Plot.

```
> pdfFlNam <- "sankeyPGrp4IRRIraw.pdf"
> showtext_auto()
> ggplot(data = pGrpSan, aes(x = x, next_x = next_x, node = node, next_node = next_node,
+                            fill = node, label = node)) +
+     geom_sankey(flow.alpha = 0.6, node.color = "grey30") +
+     geom_sankey_label(size = 5, color = "grey20", fill = "grey90", alpha = 0.8) +
+     scale_fill_manual(values = pGrp8Colors) +
+     theme_sankey(base_size = 20, base_family = "myriad") +
+     theme(strip.background = element_rect(fill = "grey95", linetype = "blank"),
+           legend.position = "none")
> ggsave(pdfFlNam, width = 8, height = 6, units = "in",
+           device = "pdf", useDingbats = FALSE)
> cat("\\includegraphics[width=0.9\\textwidth]{", pdfFlNam, "}\n\n", sep = "")
```

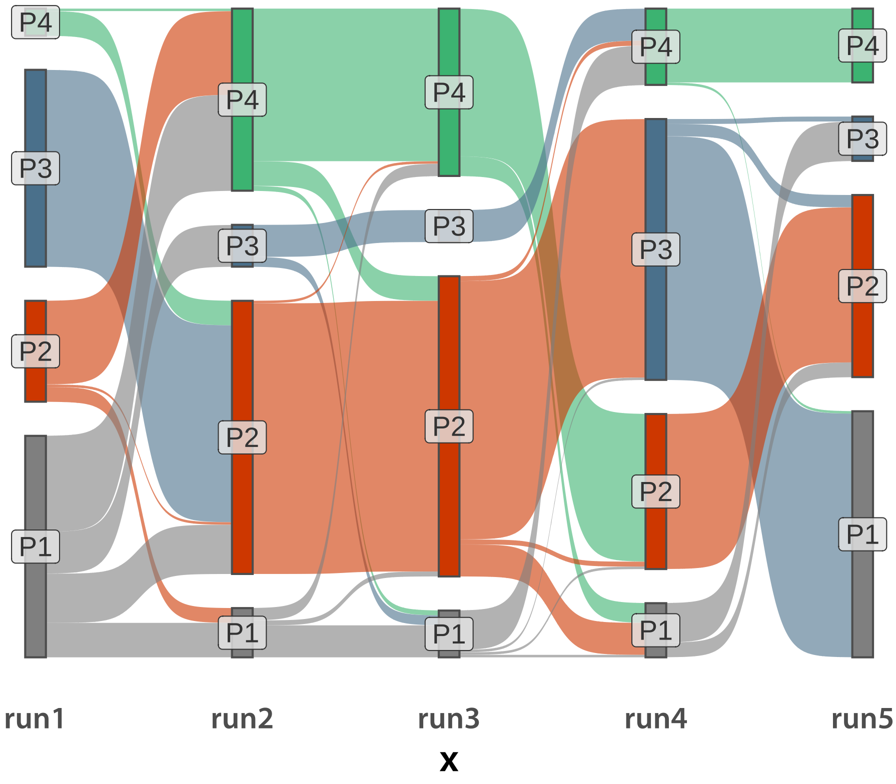

Add the $N_G = 4$ runs together and re-arrange group labels as close as possible to the old ones.

```
> newGrp    <- fread("./IRRIgroups.tsv")
> rfmix     <- fread("./Q_val_sum_RFMIX.tsv")
> ordIRRIp4 <- c(ordIRRI[[3]], ordIRRIp4)
> pMat      <- ordIRRIp4[[1]]$p[, c(1, 2, 4, 3)]
> nReps     <- 20
> indList   <- lapply(2:nReps, function(i){1:Ngrp})
```

```
> # P1
> simList   <- lapply(1:(nReps - 1), bestColLst,
+              ordIRRIp4[-1], indList, pMat[, 1], 0.9)
> bestInd   <- unlist(lapply(simList, which.max))
> trash     <- sapply(1:(nReps - 1), addCol, 1, Ngrp)
> # P4
> simList   <- lapply(1:(nReps - 1), bestColLst,
+              ordIRRIp4[-1], indList, pMat[, 4], 0.9)
> bestInd   <- unlist(lapply(simList, which.max))
> trash     <- sapply(1:(nReps - 1), addCol, 4, Ngrp)
> # P2
> simList   <- lapply(1:(nReps - 1), bestColLst,
+              ordIRRIp4[-1], indList, pMat[, 2], 0.9)
> bestInd  <- unlist(lapply(simList, which.max))
> trash    <- sapply(1:(nReps - 1), addCol, 2, Ngrp)
> # P3
> bestInd  <- rep(1, nReps - 1)
> trash    <- sapply(1:(nReps - 1), addCol, 3, Ngrp)
> pMat     <- t(apply(pMat, 1, normalizeP))
> pMat3    <- cbind(pMat[, 1], rowSums(pMat[, 2:3]), pMat[, 4])
> fwrite(pMat3, file = "ordIRRIP4.tsv", sep = "\t",
+              col.names = FALSE)
> p4Vec    <- apply(pMat3, 1, renameGroups)
> p23Vec   <- apply(pMat3, 1, renameGroups23)
> newGrp   <- newGrp[, newGrp4 := p4Vec]
> newGrp   <- newGrp[, newGrp23 := p23Vec]
> newGrp   <- newGrp[, grp4p :=  apply(pMat3, 1, max)]
```

I next plot the probability of belonging to a group for each accession (similar to a STRUCTURE plot).

```
> pvDT      <- as.data.table(round(pMat, 3))
> pMatIRRI4 <- round(pMat, 3)
> setnames(pvDT, paste0("P", 1:Ngrp))
> pvDT <- pvDT[, accession := newGrp[, NSFTV_ID]]
```

I set up two order variables, one reflecting phenotypic group IDs and probabilities, and another reflecting *O. sativa* introgression within phenotypic groups.

```
> pvDT <- pvDT[, orderVar1 := apply(round(pMat, 3), 1, which.max)]
> pvDT <- pvDT[, maxP := round(apply(pMat, 1, max), 3)]
> pvDT <- pvDT[, orderVar1 := ifelse((orderVar1 == 1) & (maxP >= 0.35),
+                          orderVar1 + 1 - maxP, orderVar1)]
> pvDT <- pvDT[, orderVar1 := ifelse((orderVar1 == 4) & (maxP >= 0.35),
+                          orderVar1 + maxP, orderVar1)]
> pvDT <- pvDT[, orderVar1 := ifelse((orderVar1 == 2) & (maxP >= 0.5),
+                          orderVar1 + 1 - maxP, orderVar1)]
> pvDT
```

```
       P1   P2   P3   P4 accession orderVar1 maxP
  1: 0.00 0.05 0.00 0.95    NID401      4.95 0.95
  2: 0.05 0.90 0.05 0.00    NID402      2.10 0.90
  3: 1.00 0.00 0.00 0.00    NID403      1.00 1.00
  4: 0.20 0.60 0.05 0.15    NID404      2.40 0.60
  5: 0.00 0.00 0.00 1.00    NID405      5.00 1.00
 ---
218: 1.00 0.00 0.00 0.00    NID755      1.00 1.00
219: 0.00 0.05 0.00 0.95    NID757      4.95 0.95
220: 1.00 0.00 0.00 0.00    NID759      1.00 1.00
221: 0.00 0.00 0.00 1.00    NID760      5.00 1.00
222: 0.00 0.05 0.00 0.95    NID762      4.95 0.95
```

```
> oSat <- rfmix[NSFTV_ID %in% pvDT[, accession], .(NSFTV_ID, O_sativa)]
```

Accessions that were used for training and therefore absent from `rfmix` results.

```
> oSat <- rbind(oSat,
+         data.table(
+         NSFTV_ID = newGrp[!(NSFTV_ID %in% oSat[, NSFTV_ID]), NSFTV_ID],
+         O_sativa = 0))
> oSat <- oSat[, NSFTV_ID := factor(NSFTV_ID, levels = unique(pvDT[, accession]))]
> names(oSat) <- c("accession", "O_sativa")
> oSat
```

```
     accession O_sativa
  1:    NID405     0.19
  2:    NID413     0.02
  3:    NID446     0.02
  4:    NID449     0.01
  5:    NID450     0.01
 ---
218:    NID720     0.00
219:    NID725     0.00
220:    NID737     0.00
221:    NID738     0.00
222:    NID743     0.00
```

```
> pvDT <- pvDT[oSat, on = "accession"]
> pvDT <- pvDT[, orderVar2 := ifelse(orderVar1 >= 4, 1 - O_sativa, O_sativa)]
> pvDT <- setorderv(pvDT, c("orderVar1", "orderVar2"), c(1, 1))
> pvDT <- melt(pvDT, measure = paste0("P", 1:4),
+             variable.name = "group", value.name = "p")
> pvDT <- pvDT[, accession := factor(accession, levels = unique(accession))]
```

Plot.

```
> pdfFlNam <- "structIRRIP4.pdf"
> showtext_auto()
> ggplot(data = pvDT, aes(x = accession, y = p, fill = group)) +
+     geom_col() +
+     scale_fill_manual(values = pGrp8Colors[c(1, 2, 4, 3)]) +
+     geom_rug(aes(x = accession, alpha = O_sativa), sides = "b") +
+     theme_classic(base_size = 18, base_family = "myriad") +
+     guides(alpha = guide_legend(expression(italic("O. sativa")))) +
+     theme(axis.line = element_blank(), axis.text.x = element_blank(),
+         axis.ticks.x  = element_blank())
> ggsave(pdfFlNam, width = 8, height = 6, units = "in",
+             device = "pdf", useDingbats = FALSE)
> cat("\\includegraphics[width = 0.95\\textwidth]{", pdfFlNam, "}\n\n", sep = "")
```

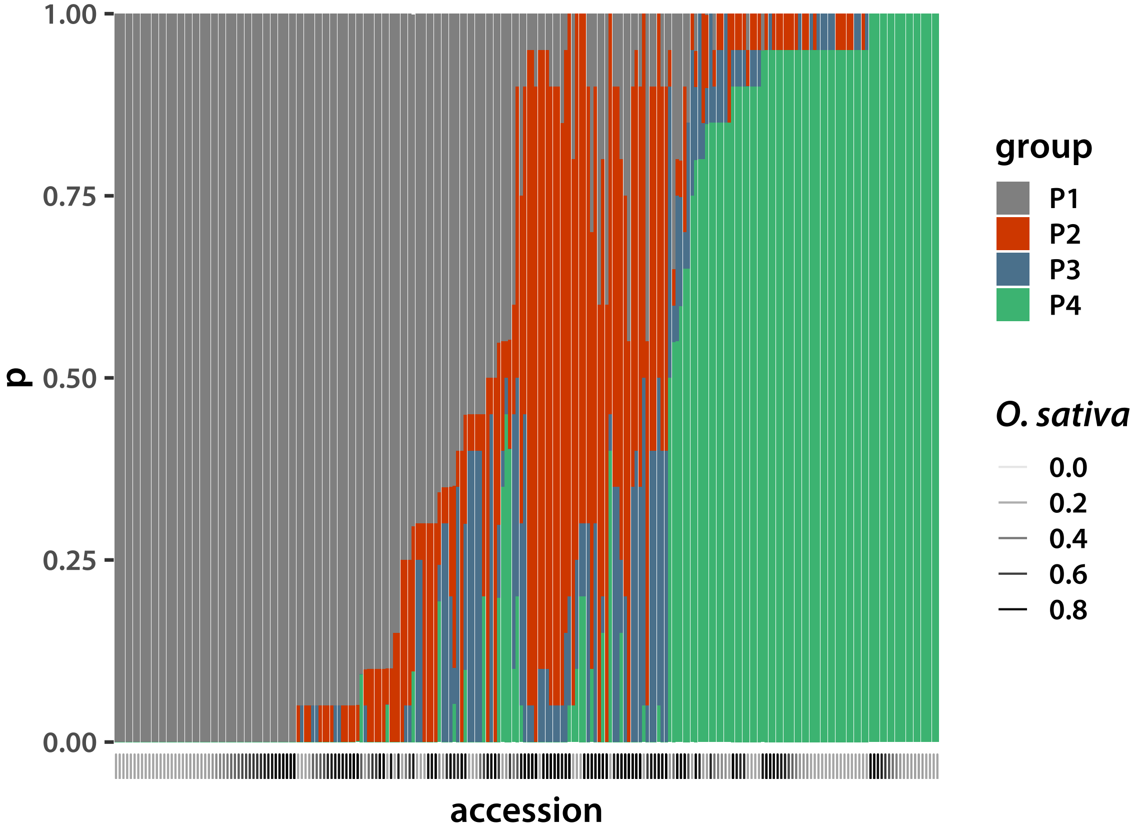

Now average the $N_G = 3$ runs the same as $N_G = 4$.

```
> nReps <- 15
> Ngrp  <- 3
> if (file.exists("ordIRRIP3.Rdata")) {
+     load(file = "ordIRRIP3.Rdata")
```

```
+ } else {
+     ordIRRIp3 <- replicate(nReps,
+                     MuGaMix::quickFitModel(Ysc, trtNamesNB, Ngrp, alpha0, nVBreps),
+                     simplify = FALSE)
+     save(ordIRRIp3, file = "ordIRRIP3.Rdata")
+ }
> addCol <- function(repInd, grpInd, Ngrp){
+     pMat[, grpInd] <<- (pMat[, grpInd] +
+             ordIRRIp3[[repInd + 1]]$p[,
+                                  indList[[repInd]][bestInd[repInd]]])
+     indList[[repInd]] <<- indList[[repInd]][-bestInd[repInd]]
+     return(NULL)
+ }
> nReps     <- 20
> pMat      <- ordIRRI[[2]][[2]]$p[, c(2, 1, 3)]
> ordIRRIp3 <- c(ordIRRI[[2]], ordIRRIp3)
> indList   <- lapply(2:nReps, function(i){1:Ngrp})
> # P1
> simList   <- lapply(1:(nReps - 1), bestColLst,
+             ordIRRIp3[-2], indList, pMat[, 1], 0.9)
> bestInd   <- unlist(lapply(simList, which.max))
> trash     <- sapply(1:(nReps - 1), addCol, 1, Ngrp)
> colSums(pMat)

[1] 2983.36232   12.00000   95.03062


> # P2
> simList <- lapply(1:(nReps - 1), bestColLst,
+           ordIRRIp3[-2], indList, pMat[, 3], 0.9)
> bestInd <- unlist(lapply(simList, which.max))
> trash   <- sapply(1:(nReps - 1), addCol, 2, Ngrp)
> colSums(pMat)

[1] 2983.36232  990.63831   95.03062


> # P3
> bestInd  <- rep(1, nReps - 1)
> trash    <- sapply(1:(nReps - 1), addCol, 3, Ngrp)
> pMat     <- t(apply(pMat, 1, normalizeP))
> fwrite(pMat, file = "ordIRRIP3.tsv", sep = "\t",
+             col.names = FALSE)
> newGrp   <- newGrp[, newGrp3 := paste0("P", apply(pMat, 1, which.max))]
> newGrp   <- newGrp[, grp3p := apply(pMat, 1, max)]
> p3Grp    <- newGrp[, .(newGrp3)]
```

```
> p3Grp    <- p3Grp[, newGrp3s1 := paste0("P", apply(ordIRRIp3[[1]]$p, 1, which.max))]
> p3Grp    <- p3Grp[, newGrp3s2 := paste0("P",
+                              apply(ordIRRIp3[[2]]$p[, c(2, 1, 3)], 1, which.max))]
> p3Grp    <- p3Grp[, newGrp3s3 := paste0("P",
+                              apply(ordIRRIp3[[3]]$p[, c(3, 1, 2)], 1, which.max))]
```

Add $N_G = 6$ and 8.

```
> lapply(ordIRRI[[5]], function(lst){lst$DIC})

[[1]]
[1] 15095.33

[[2]]
[1] 15058.01

[[3]]
[1] 15085.32

[[4]]
[1] 15050.4

[[5]]
[1] 15069.35

> lapply(ordIRRI[[7]], function(lst){lst$DIC})

[[1]]
[1] 14013.75

[[2]]
[1] 13973.85

[[3]]
[1] 13947.35

[[4]]
[1] 14004.1

[[5]]
[1] 14000.3

> newGrp <- newGrp[, newGrp6 := factor(paste0("P",
+                                  apply(ordIRRI[[5]][[4]]$p[, c(1, 3, 6, 4, 2, 5)],
+                                          1, which.max)), levels = paste0("P", 6:1))]
> newGrp <- newGrp[, newGrp8 := factor(paste0("P",
+                                  apply(ordIRRI[[7]][[3]]$p[, c(5, 1, 7, 3, 6, 8, 4, 2)],
+                                          1, which.max)), levels = paste0("P", 8:1))]
> newGrp  <- newGrp[, newGrp3 := factor(newGrp3, levels = paste0("P", 3:1))]
> newGrp  <- newGrp[, newGrp4 := factor(newGrp4, levels = paste0("P", 4:1))]
```

We received updated IRRI gene bank data listing species designations. Read the new designations from a file and relate to the accession information we already have.

```
> grinSsp <- fread("./GRINsppNames.tsv")
> newGrp  <- newGrp[grinSsp[, .(NSFTV_ID, new_Oryza_spp)], on = "NSFTV_ID", nomatch = 0]
> newGrp  <- newGrp[, sspFixed := factor(sspFixed,
+                                  levels = c("nivara", "spontanea", "rufipogon"))]
> levels(newGrp$sspFixed) <- c("O. nivara", "Oryza spp.", "O. rufipogon")
```

Compare the old and the new GRIN species designations.

```
> sppSan <- as.data.table(ggsankey::make_long(newGrp, sspFixed, new_Oryza_spp))
> sppSan <- sppSan[, node := factor(node,
+                          levels = c("O. nivara", "Oryza spp.", "O. rufipogon"))]
> sppSan <- sppSan[, next_node := factor(next_node,
+                          levels = c("O. nivara", "Oryza spp.", "O. rufipogon"))]
```

Plot.

```
> pdfFlNam <- "sankeyGRINsppCompare.pdf"
> showtext_auto()
> ggplot(data = sppSan, aes(x = x, next_x = next_x, node = node, next_node = next_node,
+                              fill = node, label = node)) +
+     geom_sankey(flow.alpha = 0.6, node.color = "grey30") +
+     geom_sankey_label(size = 5, color = "grey20", fill = "grey90", alpha = 0.8) +
+     scale_fill_manual(values = sppColors) +
+     scale_x_discrete(labels = c("old species", "GRIN species")) +
+     theme_sankey(base_size = 20, base_family = "myriad") +
+     theme(strip.background = element_rect(fill = "grey95", linetype = "blank"),
+             legend.position = "none") +
+     labs(x = "designation")
> ggsave(pdfFlNam, width = 8, height = 6, units = "in",
+             device = "pdf", useDingbats = FALSE)
> cat("\\includegraphics[width=0.9\\textwidth]{", pdfFlNam, "}\n\n", sep = "")
```

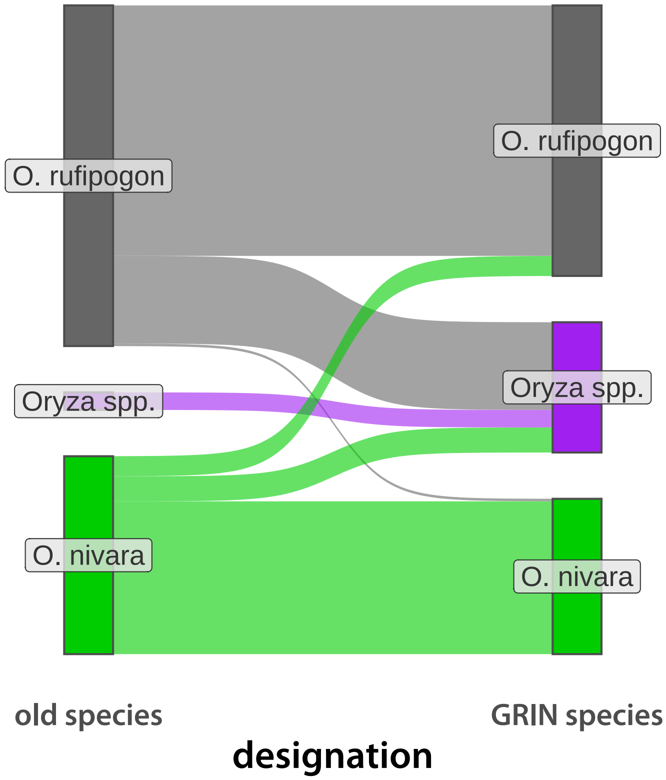


```
> newGrp[(sspFixed == "O. nivara") & (new_Oryza_spp == "O. rufipogon"), ]

   NSFTV_ID genPop probability   Species genPopOr   sspFixed genPopK8 newGrp4 newGrp23
1:   NID463     W2      0.8833 rufipogon       W2 O. nivara       W2      P4        P4
2:   NID482     W2      1.0000 rufipogon       W2 O. nivara       W2      P4        P4
3:   NID491     W2      1.0000 rufipogon       W2 O. nivara       W2      P4        P4
4:   NID492     W2      1.0000 rufipogon       W2 O. nivara       W2      P4        P4
5:   NID556     W4      1.0000 rufipogon       W4 O. nivara       W8      P4        P4
6:   NID558     W4      1.0000 rufipogon       W4 O. nivara       W4      P4        P4
7:   NID666     W2      1.0000 rufipogon       W2 O. nivara       W2      P4        P4
8:   NID735     W4      0.9668 rufipogon       W4 O. nivara       W8      P4        P4
       grp4p newGrp3     grp3p newGrp6 newGrp8 new_Oryza_spp
1: 0.9500000      P1 0.6000000      P4      P4  O. rufipogon
2: 0.8499068      P1 0.5004958      P4      P4  O. rufipogon
3: 0.8500000      P1 0.5500001      P4      P6  O. rufipogon
4: 0.8499998      P1 0.5500010      P4      P5  O. rufipogon
5: 0.9500000      P1 0.5500000      P4      P4  O. rufipogon
6: 1.0000000      P1 0.5500000      P4      P4  O. rufipogon
7: 0.9500000      P1 0.5500000      P4      P4  O. rufipogon
8: 0.9500000      P2 0.4997681      P4      P4  O. rufipogon
```

```
> newGrp[(sspFixed == "O. rufipogon") & (new_Oryza_spp == "O. nivara"), ]
```

```
   NSFTV_ID genPop probability   Species genPopOr     sspFixed genPopK8 newGrp4 newGrp23
1:   NID715     W4      0.5611 rufipogon       W4 O. rufipogon       W8      P4       P4
       grp4p newGrp3     grp3p newGrp6 newGrp8 new_Oryza_spp
1: 0.7992102      P1 0.7501212      P3      P5     O. nivara
```

There are eight accessions that are clearly misidentified in GRIN as *O. rufipogon* when both Kim *et al.* genotypic and our phenotypic groups confidently assign them to *O. nivara*. On the other hand, the single GRIN *O. nivara* that is listed as *O. rufipogon* in our data set is correct in GRIN. Fix the designations accordingly.

```
> newGrp <- newGrp[, GRIN_spp :=
+         ifelse((sspFixed == "O. nivara") & (new_Oryza_spp == "O. rufipogon"),
+                "O. nivara", new_Oryza_spp)]
> newGrp[(sspFixed == "O. nivara") & (new_Oryza_spp == "O. rufipogon"), ]
```

```
   NSFTV_ID genPop probability   Species genPopOr  sspFixed genPopK8 newGrp4 newGrp23
1:   NID463     W2      0.8833 rufipogon       W2 O. nivara       W2      P4       P4
2:   NID482     W2      1.0000 rufipogon       W2 O. nivara       W2      P4       P4
3:   NID491     W2      1.0000 rufipogon       W2 O. nivara       W2      P4       P4
4:   NID492     W2      1.0000 rufipogon       W2 O. nivara       W2      P4       P4
5:   NID556     W4      1.0000 rufipogon       W4 O. nivara       W8      P4       P4
6:   NID558     W4      1.0000 rufipogon       W4 O. nivara       W4      P4       P4
7:   NID666     W2      1.0000 rufipogon       W2 O. nivara       W2      P4       P4
8:   NID735     W4      0.9668 rufipogon       W4 O. nivara       W8      P4       P4
       grp4p newGrp3     grp3p newGrp6 newGrp8 new_Oryza_spp  GRIN_spp
1: 0.9500000      P1 0.6000000      P4      P4  O. rufipogon O. nivara
2: 0.8499068      P1 0.5004958      P4      P4  O. rufipogon O. nivara
3: 0.8500000      P1 0.5500001      P4      P6  O. rufipogon O. nivara
4: 0.8499998      P1 0.5500010      P4      P5  O. rufipogon O. nivara
5: 0.9500000      P1 0.5500000      P4      P4  O. rufipogon O. nivara
6: 1.0000000      P1 0.5500000      P4      P4  O. rufipogon O. nivara
7: 0.9500000      P1 0.5500000      P4      P4  O. rufipogon O. nivara
8: 0.9500000      P2 0.4997681      P4      P4  O. rufipogon O. nivara
```

Now look at the correspondence among phenotypic groups and species.

```
> pGrpSan <- as.data.table(ggsankey::make_long(newGrp, GRIN_spp, newGrp3, newGrp4,
+                                               newGrp6, newGrp8))
> pGrpSan <- pGrpSan[, node :=
+                ifelse((node == "P2") & (x == "newGrp4"), "P2/P3", node) ]
> pGrpSan <- pGrpSan[, next_node :=
+                ifelse((next_node == "P2") & (next_x == "newGrp4"), "P2/P3", next_node) ]
> pInds   <- c(paste0("P", c(4, 2)), "P2/P3", paste0("P", c(8, 7, 6, 5, 3, 1)))
> pGrpSan <- pGrpSan[, node := factor(node,
+                     levels = c("O. nivara", "Oryza spp.", "O. rufipogon", pInds))]
> pGrpSan <- pGrpSan[, next_node := factor(next_node,
+                     levels = c("O. nivara", "Oryza spp.", "O. rufipogon", pInds))]
> pGrpSan
```

```
             x         node  next_x next_node
   1: GRIN_spp O. rufipogon newGrp3        P1
   2:  newGrp3           P1 newGrp4        P4
   3:  newGrp4           P4 newGrp6        P4
   4:  newGrp6           P4 newGrp8        P3
   5:  newGrp8           P3    <NA>      <NA>
  ---
1106: GRIN_spp    O. nivara newGrp3        P1
1107:  newGrp3           P1 newGrp4        P4
1108:  newGrp4           P4 newGrp6        P4
1109:  newGrp6           P4 newGrp8        P4
1110:  newGrp8           P4    <NA>      <NA>
```

Plot.

```
> pdfFlNam <- "sankeyPGrpIRRI.pdf"
> showtext_auto()
> ggplot(data = pGrpSan, aes(x = x, next_x = next_x, node = node, next_node = next_node,
+                              fill = node, label = node)) +
+     geom_sankey(flow.alpha = 0.6, node.color = "grey30") +
+     geom_sankey_label(size = 5, color = "grey20", fill = "grey90", alpha = 0.8,
+             label = c(expression(italic("O. nivara")),
+                       expression(italic("Oryza spp.")),
+                       expression(italic("O. rufipogon")),
+                       "P2", "P3", "P1", "P4", "P2/P3", "P1",
+                       "P4", "P2", "P6", "P5", "P3", "P1",
+                       "P4", "P2", "P8", "P7", "P6", "P5", "P3", "P1")) +
+     scale_fill_manual(values = pGrp8Colors) +
+     scale_x_discrete(labels = c("species", "3", "4", "6", "8")) +
+     theme_sankey(base_size = 20, base_family = "myriad") +
+     theme(strip.background = element_rect(fill = "grey95", linetype = "blank"),
+             legend.position = "none") +
+     labs(x = expression(N[G]))
> ggsave(pdfFlNam, width = 8, height = 6, units = "in",
+             device = "pdf", useDingbats = FALSE)
> cat("\\includegraphics[width=0.9\\textwidth]{", pdfFlNam, "}\n\n", sep = "")
```

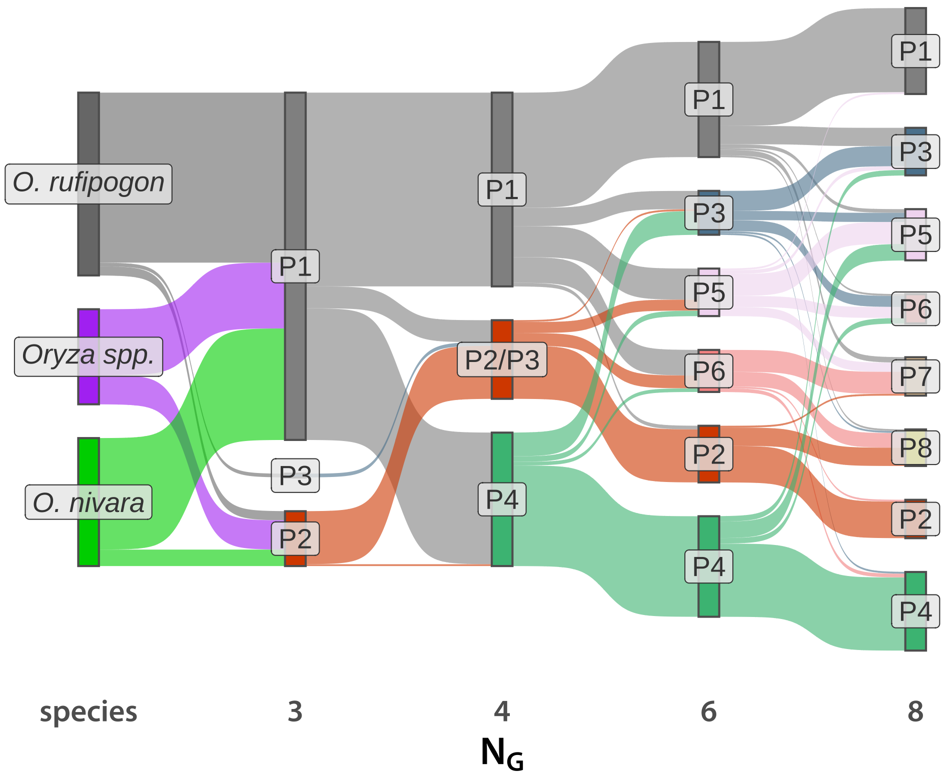

Plot individual $N_G = 3$ results.

```
> pdfFlNam <- "sankeyPGrp3IRRI.pdf"
> pGrpSan  <- as.data.table(ggsankey::make_long(p3Grp, newGrp3s1,
+                                      newGrp3s2, newGrp3s3, newGrp3))
> showtext_auto()
> ggplot(data = pGrpSan, aes(x = x, next_x = next_x, node = node, next_node = next_node,
+                              fill = node, label = node)) +
+     geom_sankey(flow.alpha = 0.6, node.color = "grey30") +
+     geom_sankey_label(size = 5, color = "grey20", fill = "grey90", alpha = 0.8) +
+     scale_fill_manual(values = pGrp8Colors) +
+     theme_sankey(base_size = 20, base_family = "myriad") +
+     theme(strip.background = element_rect(fill = "grey95", linetype = "blank"),
+             legend.position = "none")
> ggsave(pdfFlNam, width = 8, height = 6, units = "in",
+             device = "pdf", useDingbats = FALSE)
> cat("\\includegraphics[width=0.9\\textwidth]{", pdfFlNam, "}\n\n", sep = "")
```

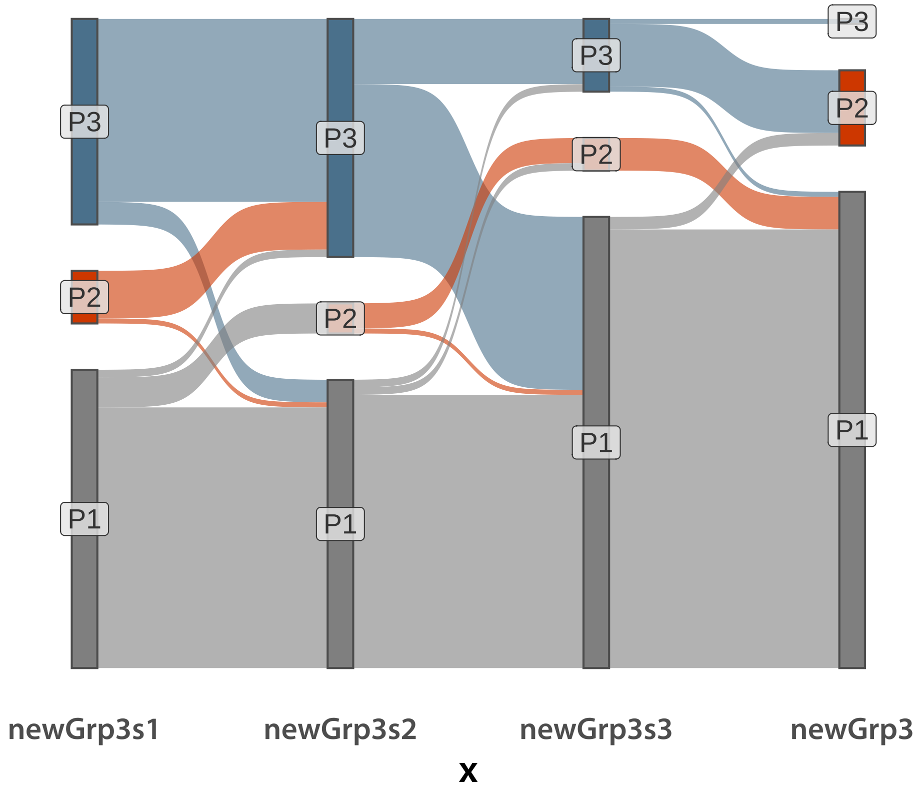

Make the supplemental table.

```
> oldTable <- fread("./germplasmSuppTable.tsv")
> oldTable <- merge(oldTable, newGrp[, .(NSFTV_ID, newGrp23, grp4p, GRIN_spp)],
+         by.x = "NSFTV_ID", by.y = "NSFTV_ID", all.x = TRUE, all.y = TRUE)
> oldTable <- oldTable[, grp4p := round(grp4p, 3)]
> oldTable <- merge(oldTable, rfmix[, .(NSFTV_ID, O_sativa)],
+         by.x = "NSFTV_ID", by.y = "NSFTV_ID", all.x = TRUE, all.y = FALSE)
> fwrite(oldTable, file = "germplasmSuppTableNew.tsv", quote = FALSE, na = "NA",
+         sep = "\t")
```

Identify accessions with name changes.

```
> oldTable[Species != GRIN_spp & GRIN_spp != "Oryza spp.", ]

   NSFTV_ID    IRGC_ID      Species     Country    Latitude    Longitude
1:   NID463 IRGC104599 O. rufipogon   Sri Lanka    7°40'''N    80°10'''E
2:   NID482 IRGC105349 O. rufipogon       India   10°30'''N     76°6'''E
3:   NID491 IRGC105568 O. rufipogon Philippines  7°52'48''N  125°0'21''E
4:   NID492 IRGC105569 O. rufipogon    Cambodia 12°46'17''N 102°38'44''E
5:   NID556 IRGC105494 O. rufipogon     Myanmar  18°49'0''N   95°13'0''E
6:   NID558 IRGC105616 O. rufipogon       China 23°44'56''N  108°18'6''E
7:   NID666 IRGC100211 O. rufipogon       India 10°28'19''N  76°25'28''E
```

```
8:   NID715 IRGC104497 O. rufipogon    Thailand  13°2'22''N 101°29'31''E
9:   NID735 IRGC105493 O. rufipogon     Myanmar   20°3'6''N   95°8'19''E
   Kim2016_population Huang2012_population IRRI_phenotype CU_phenotype DB_phenotype
1:                 W2                 <NA>           IRRI           CU           DB
2:                 W2                 <NA>           IRRI           CU           DB
3:                 W2               Or-III           IRRI         <NA>         <NA>
4:                 W2                 Or-I           IRRI           CU           DB
5:                 W4                 Or-I           IRRI         <NA>           DB
6:                 W4                 <NA>           IRRI         <NA>           DB
7:                 W2                 <NA>           IRRI           CU           DB
8:    Admix of W4/ W1                 <NA>           IRRI           CU           DB
9:                 W4                 Or-I           IRRI         <NA>         <NA>
   newGrp23 grp4p  GRIN_spp O_sativa
1:       P4 0.950 O. nivara     0.00
2:       P4 0.850 O. nivara       NA
3:       P4 0.850 O. nivara     0.05
4:       P4 0.850 O. nivara     0.04
5:       P4 0.950 O. nivara     0.10
6:       P4 1.000 O. nivara     0.13
7:       P4 0.950 O. nivara       NA
8:       P4 0.799 O. nivara     0.42
9:       P4 0.950 O. nivara     0.02
```

```
> fwrite(oldTable[Species != GRIN_spp & GRIN_spp != "Oryza spp.", ],
+     file = "nameChanges.tsv", quote = FALSE, na = "NA", sep = "\t")
> pureRufi <- rbind(oldTable[GRIN_spp == "O. rufipogon" &
+         Kim2016_population %in% c("W3", "W1", "W6") &
+         newGrp23 == "P1" & grp4p > 0.8 &
+         O_sativa < 0.2, ],
+     oldTable[GRIN_spp == "O. rufipogon" &
+         Kim2016_population %in% c("W3", "W1", "W6") &
+         newGrp23 == "P1" & grp4p > 0.8 &
+         is.na(O_sativa), ])
> fwrite(pureRufi, file = "pureRufi.tsv",
+         sep = "\t", quote = FALSE, na = "NA")
> pureNivara <- rbind(oldTable[GRIN_spp == "O. nivara" &
+         Kim2016_population %in% c("W2", "W4", "W5") &
+         newGrp23 == "P4" & grp4p > 0.8 &
+         O_sativa < 0.2, ],
+     oldTable[GRIN_spp == "O. nivara" &
+         Kim2016_population %in% c("W2", "W4", "W5") &
+         newGrp23 == "P4" & grp4p > 0.8 &
+         is.na(O_sativa), ])
> fwrite(pureNivara, file = "pureNivara.tsv",
+         sep = "\t", quote = FALSE, na = "NA")
```

Export the p-value matrices.

2 Relationship to genetic groups

Build the genetic population plot.

```
> popGrpColors <- c("P1" = "grey50", "P2/P3" = "orangered3",
+                    "P2" = "orangered3",
+                    "P3" = "skyblue4", "P4" = "mediumseagreen",
+                    "W1" = "grey50", "W2" = "palegreen3", "W3" = "grey40",
+                    "W4" = "olivedrab4", "W5" = "palegreen",
+                    "W6" = "lightsteelblue",
+                    "O. rufipogon" = "grey40", "O. nivara" = "green3",
+                    "Oryza spp." = "purple", "ADM/OSAT" = "orangered2")
> pGrpSan      <- as.data.table(ggsankey::make_long(newGrp, GRIN_spp, newGrp23, genPop))
> sankeyLevels <- c("O. nivara", "Oryza spp.", "O. rufipogon", c("P4", "P2/P3", "P1"),
+                    paste0("W", c(5, 2, 4, 6, 1, 3)))
> pGrpSan      <- pGrpSan[, node := factor(node, levels = sankeyLevels)]
> pGrpSan      <- pGrpSan[, next_node := factor(next_node, levels = sankeyLevels)]
```

Plot.

```
> pdfFlNam <- "sankeyPGrpPopsIRRI.pdf"
> showtext_auto()
> ggplot(data = pGrpSan, aes(x = x, next_x = next_x, node = node, next_node = next_node,
+                            fill = node, label = node)) +
+     geom_sankey(flow.alpha = 0.6, node.color = "grey30") +
+     geom_sankey_label(size = 5, color = "grey20", fill = "grey90", alpha = 0.8) +
+     scale_fill_manual(values = popGrpColors) +
+     scale_x_discrete(labels = c("species", "phenotypic\ngroups",
+                         "genetic\nsubpopulations")) +
+     theme_sankey(base_size = 20, base_family = "myriad") +
+     theme(strip.background = element_rect(fill = "grey95", linetype = "blank"),
+            legend.position = "none") + labs(x = NULL)
> ggsave(pdfFlNam, width = 8, height = 6, units = "in",
+            device = "pdf", useDingbats = FALSE)
> cat("\\includegraphics[width=0.9\\textwidth]{", pdfFlNam, "}\n\n", sep = "")
```

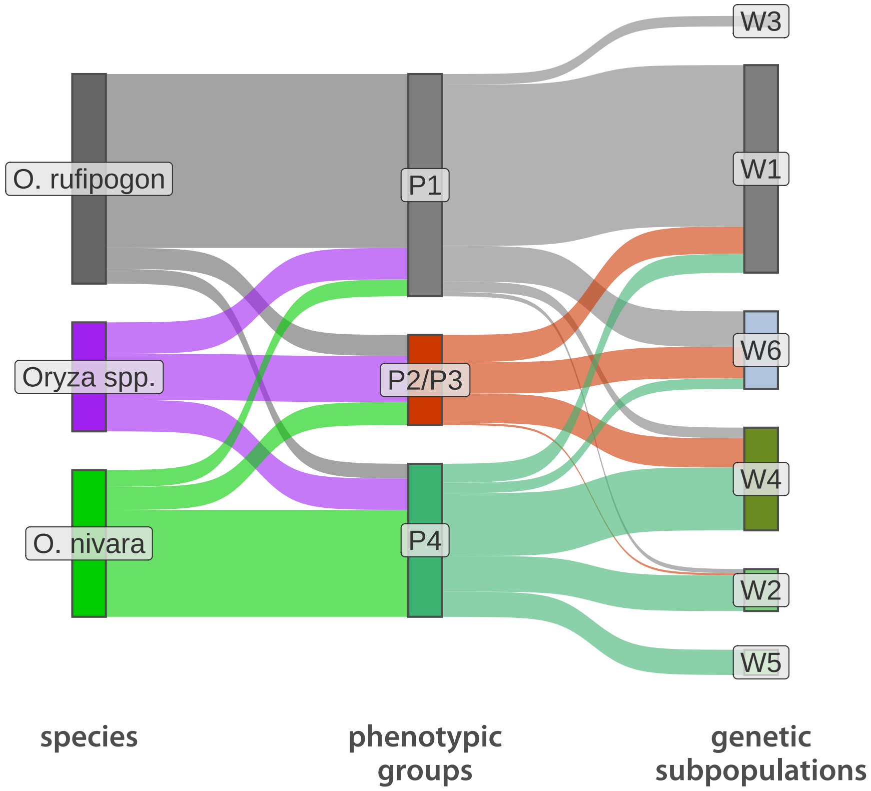

Fraction of accessions that are in the wrong P group.

```
> newGrp[(newGrp23 == "P1") &
+     (sspFixed == "O. nivara") &
+     (genPopK8 %in% c("W4", "W2")), .N]

[1] 1
```

```
> newGrp[(newGrp23 == "P4") &
+     (sspFixed == "O. rufipogon") &
+     (genPopK8 %in% c("W1", "W6")), .N]

[1] 9
```

```
> newGrp[(newGrp23 == "P1") &
+     (sspFixed == "O. nivara") &
+     (genPopK8 %in% c("W4", "W2")), .N]/newGrp[newGrp23 == "P1", .N]

[1] 0.009433962
```

```
> newGrp[(newGrp23 == "P4") &
+     (sspFixed == "O. rufipogon") &
+     (genPopK8 %in% c("W1", "W6")), .N]/newGrp[newGrp23 == "P4", .N]
```

```
[1] 0.1232877
```

```
> newGrp[, sum(newGrp23 == "P2/P3")]/newGrp[, .N]
```

```
[1] 0.1936937
```

Re-make the RFMIX genome fraction boxplots.

```
> sativaGrp  <- c("ARO", "AUS", "IND", "TEJ", "TRJ", "O_sativa")
> sativaCols <- c("ARO" = "purple", "AUS" = "sienna1", "IND" = "yellow",
+                 "TRJ" = "royalblue", "TEJ" = "blue")
> rufiGrp    <- paste0("W", 1:6)
> rfmix      <- rfmix[, sativaInd := ifelse(O_sativa >= 0.25, "O. sativa", "ORSC")]
> colSubset  <- c("NSFTV_ID", "newGrp23")
> rfmixPG    <- newGrp[, ..colSubset][rfmix, on = "NSFTV_ID", nomatch = 0]
> rfmixPG    <- melt(rfmixPG, id.vars = colSubset,
+                 measure.vars = c(sativaGrp, rufiGrp),
+                 variable.name = "population",
+                 value.name = "fraction")
> rfmixPG    <- rfmixPG[, newGrp23 := factor(newGrp23, levels = c("P1", "P2/P3", "P4"))]
> rfmixPG
```

```
      NSFTV_ID newGrp23 population fraction
   1:   NID405       P4        ARO     0.00
   2:   NID413       P4        ARO     0.00
   3:   NID446       P4        ARO     0.00
   4:   NID449       P4        ARO     0.00
   5:   NID450       P4        ARO     0.00
  ---
2108:   NID731       P1         W6     0.03
2109:   NID734       P1         W6     0.00
2110:   NID752    P2/P3         W6     0.00
2111:   NID753       P1         W6     0.03
2112:   NID759       P1         W6     0.02
```

First, I plot the fraction of *O. rufipogon* populations per $N_G = 4$ phenotypic groups.

```
> pdfFlNam <- "rfmixRufiGrp4.pdf"
> showtext_auto()
> ggplot(data = rfmixPG[population %in% rufiGrp, ],
+         aes(x = newGrp23, y = fraction, fill = population)) +
+     geom_boxplot() +
+     scale_fill_manual(values = popGrpColors[6:11]) +
+     theme_classic(base_size = 14, base_family = "myriad") +
```

```
+     theme(legend.position = "top") +
+     xlab("phenotypic group") + ylab("genome fraction") +
+     labs(fill = "subpopulation")
> ggsave(pdfFlNam, width = 5, height = 5, units = "in",
+             device = "pdf", useDingbats = FALSE)
> cat("\\includegraphics[width = 0.95\\textwidth]{", pdfFlNam, "}\n\n", sep = "")
```

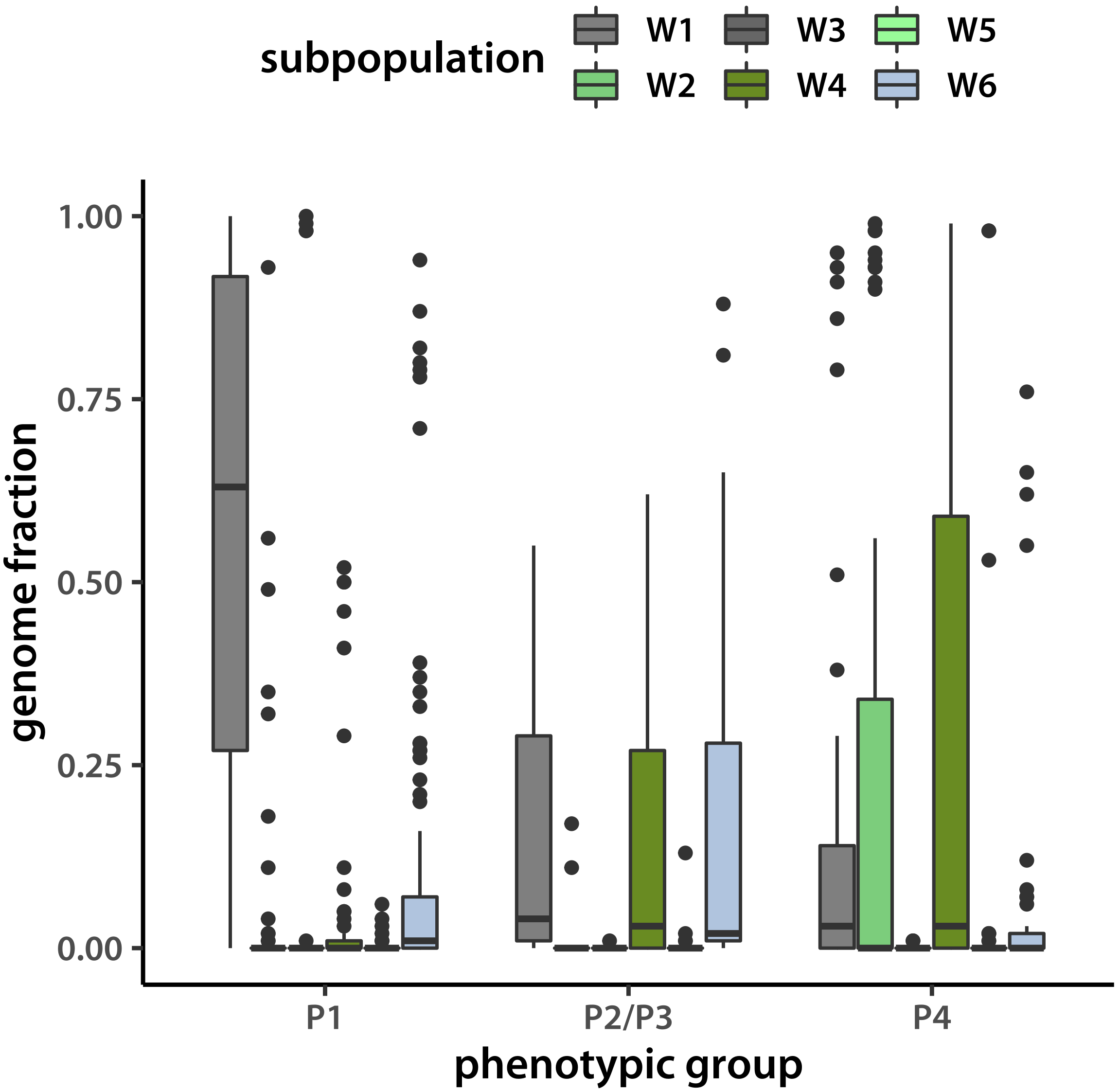

Now do the same with the *O. sativa* introgressions.

```
> pdfFlNam <- "rfmixSativaGrp4.pdf"
> showtext_auto()
> ggplot(data = rfmixPG[population %in% sativaGrp[-6], ],
```

```
+         aes(x = newGrp23, y = fraction, fill = population)) +
+     geom_boxplot() +
+     scale_fill_manual(values = sativaCols) +
+     theme_classic(base_size = 14, base_family = "myriad") +
+     theme(legend.position = "top") +
+     guides(fill = guide_legend(ncol = 3)) +
+     xlab("phenotypic group") + ylab("genome fraction") +
+     labs(fill = "subpopulation")
> ggsave(pdfFlNam, width = 5, height = 5, units = "in",
+             device = "pdf", useDingbats = FALSE)
> cat("\\includegraphics[width = 0.95\\textwidth]{", pdfFlNam, "}\n\n", sep = "")
```

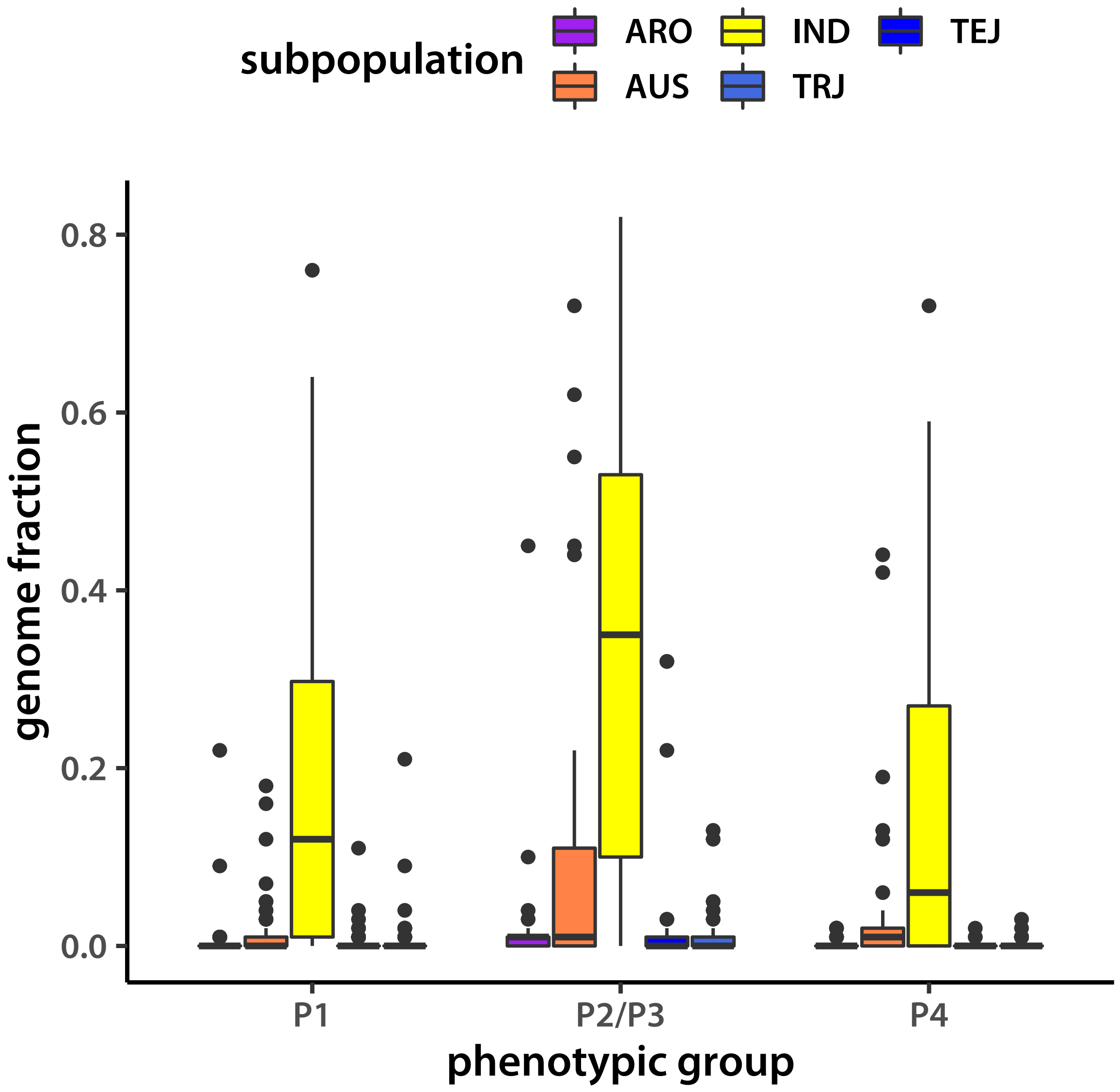

I next plot an binned cumulative distribution of genome fractions covered by *O. sativa* introgressions.

```
> rfmixSat <- rfmixPG[population == sativaGrp[6], ]
> cutOffs <- seq(from = 0.05, to = 1.0, by = 0.05)
> cumCount <- function(ct, vec){
+     sum(vec <= ct)
+ }
> rfmixSat <- rfmixSat[, sapply(cutOffs, cumCount, fraction), by = newGrp23 ]
> rfmixSat <- rfmixSat[, cumFrac := V1/(rfmixPG[population == sativaGrp[6], .N])]
> rfmixSat <- rfmixSat[, fracSat := factor(rep(cutOffs, times = 3), levels = cutOffs)]
> rfmixSat
```

```
    newGrp23 V1     cumFrac fracSat
 1:       P4 19 0.107954545    0.05
 2:       P4 24 0.136363636     0.1
 3:       P4 27 0.153409091    0.15
 4:       P4 28 0.159090909     0.2
 5:       P4 32 0.181818182    0.25
 6:       P4 35 0.198863636     0.3
 7:       P4 38 0.215909091    0.35
 8:       P4 40 0.227272727     0.4
 9:       P4 44 0.250000000    0.45
10:       P4 46 0.261363636     0.5
11:       P4 47 0.267045455    0.55
12:       P4 48 0.272727273     0.6
13:       P4 48 0.272727273    0.65
14:       P4 48 0.272727273     0.7
15:       P4 49 0.278409091    0.75
16:       P4 49 0.278409091     0.8
17:       P4 49 0.278409091    0.85
18:       P4 49 0.278409091     0.9
19:       P4 49 0.278409091    0.95
20:       P4 49 0.278409091       1
21:    P2/P3  0 0.000000000    0.05
22:    P2/P3  1 0.005681818     0.1
23:    P2/P3  2 0.011363636    0.15
24:    P2/P3  3 0.017045455     0.2
25:    P2/P3  3 0.017045455    0.25
26:    P2/P3  4 0.022727273     0.3
27:    P2/P3  7 0.039772727    0.35
28:    P2/P3  8 0.045454545     0.4
29:    P2/P3 11 0.062500000    0.45
30:    P2/P3 14 0.079545455     0.5
31:    P2/P3 19 0.107954545    0.55
32:    P2/P3 23 0.130681818     0.6
33:    P2/P3 27 0.153409091    0.65
34:    P2/P3 31 0.176136364     0.7
35:    P2/P3 35 0.198863636    0.75
36:    P2/P3 35 0.198863636     0.8
```

```
37:    P2/P3 37 0.210227273    0.85
38:    P2/P3 37 0.210227273     0.9
39:    P2/P3 37 0.210227273    0.95
40:    P2/P3 37 0.210227273       1
41:       P1 32 0.181818182    0.05
42:       P1 40 0.227272727     0.1
43:       P1 45 0.255681818    0.15
44:       P1 55 0.312500000     0.2
45:       P1 58 0.329545455    0.25
46:       P1 65 0.369318182     0.3
47:       P1 71 0.403409091    0.35
48:       P1 76 0.431818182     0.4
49:       P1 80 0.454545455    0.45
50:       P1 81 0.460227273     0.5
51:       P1 84 0.477272727    0.55
52:       P1 84 0.477272727     0.6
53:       P1 87 0.494318182    0.65
54:       P1 88 0.500000000     0.7
55:       P1 89 0.505681818    0.75
56:       P1 89 0.505681818     0.8
57:       P1 90 0.511363636    0.85
58:       P1 90 0.511363636     0.9
59:       P1 90 0.511363636    0.95
60:       P1 90 0.511363636       1
    newGrp23 V1     cumFrac fracSat
```

```
> rfmixSat <- rfmixSat[!(fracSat %in% c("0.9", "0.95", "1")), ]

> pdfFlNam <- "cumOsatFrac.pdf"
> showtext_auto()
> ggplot(data = rfmixSat, aes(x = fracSat, y = cumFrac, fill = newGrp23)) +
+     geom_col() +
+     scale_fill_manual(values = pGrp8Colors[c(1, 3, 9)]) +
+     theme_classic(base_size = 18, base_family = "myriad") +
+     labs(fill = "phenotypic\ngroups") +
+     theme(strip.background = element_rect(fill = "grey95", linetype = "blank"),
+             axis.text.x = element_text(angle = 45, hjust = 1, vjust = 1)) +
+     xlab("cumulative O. sativa fraction") + ylab("fraction of all accessions")
> ggsave(pdfFlNam, width = 8, height = 6, units = "in",
+             device = "pdf", useDingbats = FALSE)
> cat("\\includegraphics{", pdfFlNam, "}\n\n", sep = "")
```

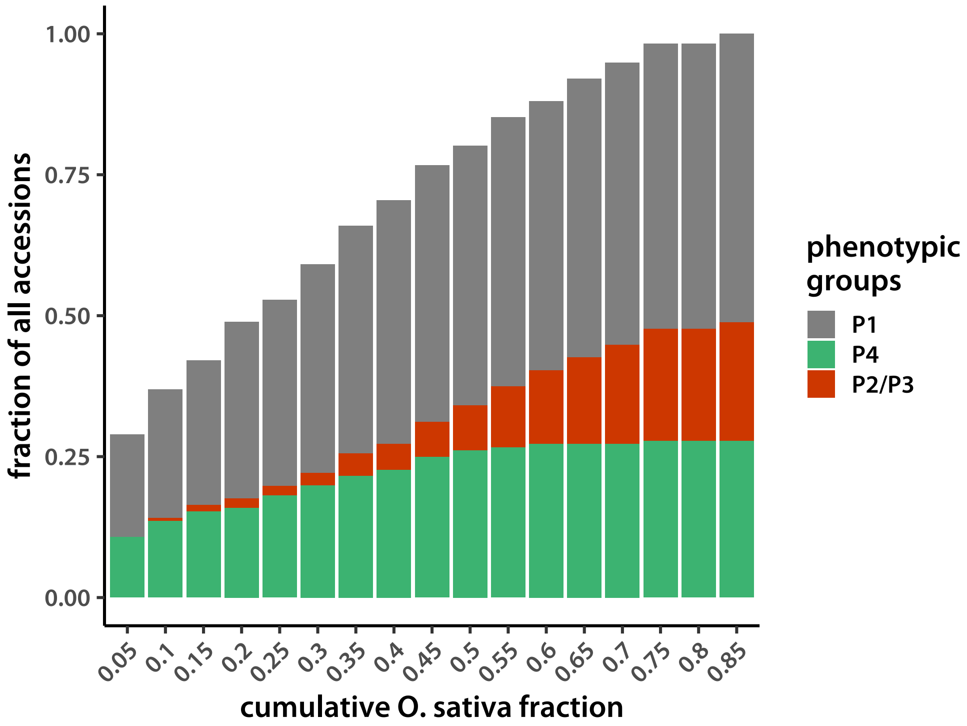

3 Georgraphical distributions

I next plot the penotypic groups on a map.

```
> coorData <- fread("./huangKimMergeJeremy.tsv")
> coorData <- coorData[Study == "Kim", ]
> keepData <- c("ID", "Country", "Latitude", "Longitude")
> coorData <- coorData[, ..keepData]
> coorGrp  <- newGrp[coorData, on = "NSFTV_ID == ID", nomatch = 0]
> coorGrp  <- coorGrp[!is.na(Longitude) & !is.na(Latitude), ]
> coorGrp  <- coorGrp[, newGrp23 := factor(newGrp23, levels = c("P1", "P2/P3", "P4"))]
> world    <- ne_countries(scale = "medium", returnclass = "sf")
> lonRange <- range(coorGrp[, Longitude])
> latRange <- range(coorGrp[, Latitude])
```

Plot.

```
> pdfFlNam <- "mapIRRIp4.pdf"
> showtext_auto()
> ggplot(data = world) +
+     geom_sf(fill = "ivory") +
+     coord_sf(xlim = lonRange, ylim = latRange) +
+     annotation_scale(location = "bl", width_hint = 0.4) +
+     geom_jitter(data = coorGrp, aes(x = Longitude, y = Latitude,
```

```
+                 color = newGrp23, alpha = grp4p), size = 2.3, width = 0.9) +
+     scale_color_manual(values = pGrp8Colors[c(1, 3, 9)]) +
+     theme_classic(base_size = 18, base_family = "myriad") +
+     labs(color = "phenotypic\ngroup") +
+     guides(alpha = "none") +
+     theme(panel.background = element_rect(fill = "aliceblue"),
+           legend.position = "top") +
+     ylab("") + xlab("")
> ggsave(pdfFlNam, width = 7, height = 8, units = "in",
+             device = "pdf", useDingbats = FALSE)
> cat("\\includegraphics[width = 0.95\\textwidth]{", pdfFlNam, "}\n\n", sep = "")
```

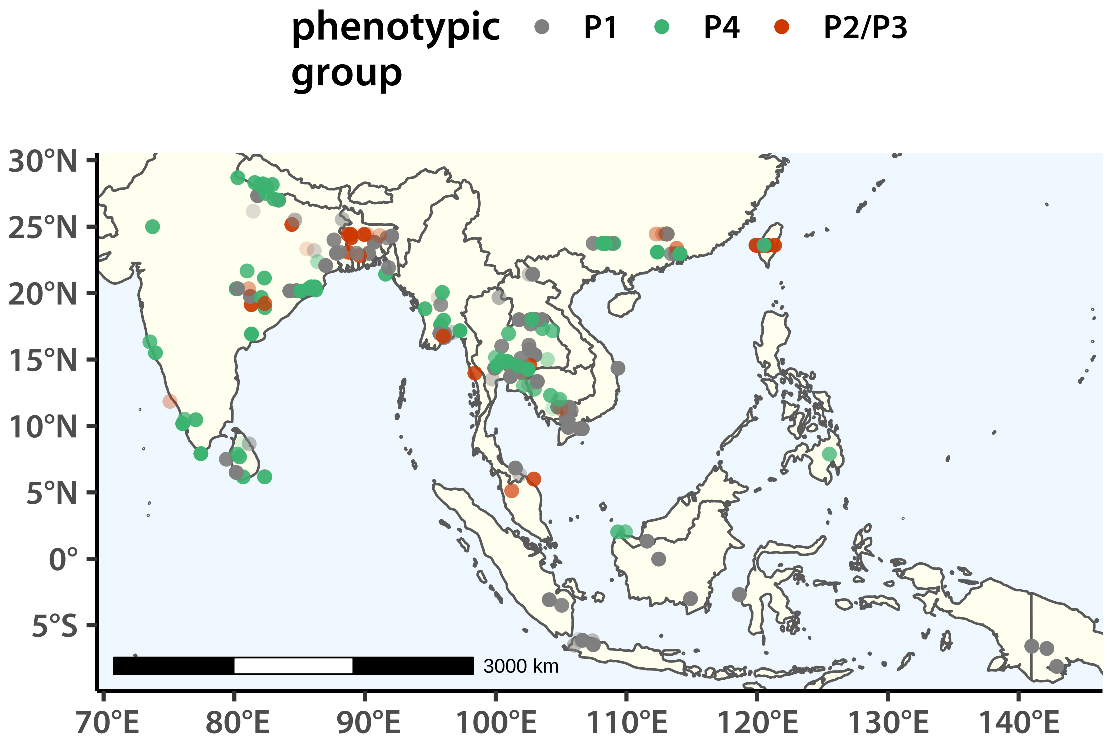

Plot the pure species.

```
> newGrpPure <- newGrp[NSFTV_ID %in% c(pureRufi$NSFTV_ID, pureNivara$NSFTV_ID), ]
> coorGrp    <- newGrpPure[coorData, on = "NSFTV_ID == ID", nomatch = 0]
> coorGrp    <- coorGrp[!is.na(Longitude) & !is.na(Latitude), ]
```

Plot.

```
> pdfFlNam <- "mapPureSpp.pdf"
> showtext_auto()
> ggplot(data = world) +
+     geom_sf(fill = "ivory") +
+     coord_sf(xlim = lonRange, ylim = latRange) +
+     annotation_scale(location = "bl", width_hint = 0.4) +
+     geom_jitter(data = coorGrp, aes(x = Longitude, y = Latitude,
+                     color = GRIN_spp), size = 2.3, width = 0.8) +
+     scale_color_manual(values = sppColors[1:2],
+         labels = c(expression(italic("O. rufipogon")), expression(italic("O. nivara")))) +
+     theme_classic(base_size = 18, base_family = "myriad") +
+     labs(color = "species") +
+     guides(alpha = "none") +
+     theme(panel.background = element_rect(fill = "aliceblue"),
+           legend.position = "top") +
+     ylab("") + xlab("")
> ggsave(pdfFlNam, width = 7, height = 8, units = "in",
+             device = "pdf", useDingbats = FALSE)
> cat("\\includegraphics[width = 0.95\\textwidth]{", pdfFlNam, "}\n\n", sep = "")
```

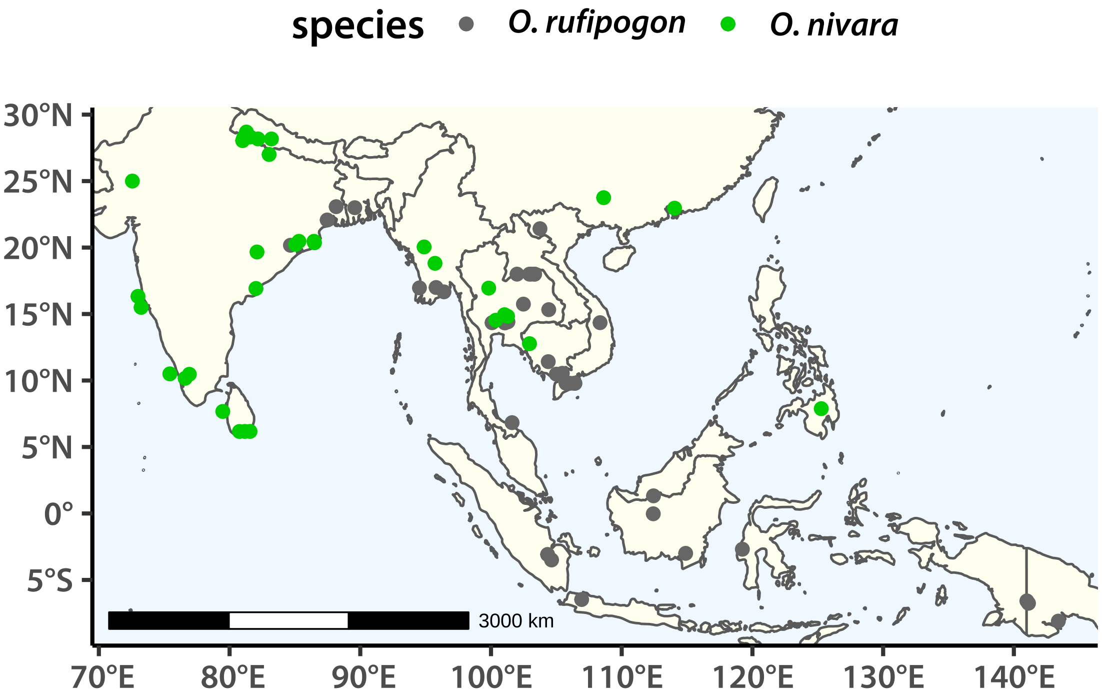

4 Trait subsets

Calculate group discrimination scores for all traits.

```
> Ysc      <- matrix(unlist(Ysc), nrow = N)
> Psc      <- pMatIRRI4[, 1:2]
> Psc[, 2] <- Psc[, 2] + pMatIRRI4[, 3]
```

```
> Psc      <- scale(Psc, scale = FALSE)
> betaEst  <- solve(crossprod(Ysc), crossprod(Ysc, Psc))
> Rsd      <- Psc - Ysc%*%betaEst
> Sest     <- crossprod(Rsd)
> Sest     <- chol2inv(chol(Sest))
> mhlDist  <- apply(betaEst, 1, mhl, Sest)
> XtX      <- colSums(Ysc*Ysc)
> mhlDist  <- mhlDist*XtX
> mhlDT    <- data.table(mhlD = mhlDist, IRRI = trtNamesNB)
> mhlDT    <- mhlDT[trtNames, on = "IRRI", nomatch = 0]
> mhlDT    <- setorder(mhlDT, -mhlD)
> mhlDT    <- mhlDT[, IRRI := factor(IRRI, levels = unique(IRRI))]
> mhlDT
```

```
            mhlD        IRRI binary    Planteome
 1: 7.119060e-01        CULT      0         PTHT
 2: 6.994920e-01    UNFILLED      0    UNFILGRNB
 3: 2.754994e-01       AWNWD      0        AWNWD
 4: 2.647407e-01        2LLT      0      PENLFLG
 5: 2.291304e-01       AWNLT      0        AWNLG
 6: 2.174085e-01       STLWD      0      STLEMWD
 7: 2.070679e-01       ANTLT      0        ANTLT
 8: 1.838106e-01        DIST      0 PanBasBrDist
 9: 1.715226e-01        CUDI      0         CUDI
10: 1.301208e-01       SPKWD      0        SPKWD
11: 1.099093e-01        FERT      0       SPKFRT
12: 9.692020e-02 CUHABIT_VEG      0  CULMHAB_VEG
13: 9.434603e-02        PNNB      0         PNNB
14: 8.401237e-02       SPKLT      0        SPKLG
15: 7.225529e-02        PNLG      0         PNLG
16: 6.101713e-02     CUHABIT      0      CULMHAB
17: 5.683213e-02       FLFWD      0       FLFLWD
18: 4.301428e-02       LIGLT      0        LIGLG
19: 3.619160e-02        2LWD      0     PENLFLWD
20: 3.159207e-02       STGLT      0       STIGLG
21: 2.792544e-02       STYLT      0       STYLLG
22: 2.583463e-02     PANTYPE      0       PANTYP
23: 2.149031e-02     PANBASE      0        PBRNB
24: 2.037659e-02        DTHD      0         DTHD
25: 1.776284e-02       FLFLG      0        FLFLG
26: 1.546352e-02     FLAGATT      0         LFAG
27: 1.300272e-02        PAMB      0     PanAxAtt
28: 8.927354e-03      PAN2BR      0     Pan2ndBr
29: 8.855837e-03      PANEXS      0       PANEXS
30: 2.520883e-03       STLLT      0      STLEMLG
31: 1.249988e-03        LEAF      0         LSEN
32: 4.167162e-05        CUNO      0         CUNO
            mhlD        IRRI binary    Planteome
```

```
> fwrite(mhlDT, file = "./MahalanobisIRRI.tsv", sep = "\t", quote = FALSE)
```

Plot the sorted distances.

```
> pdfFlNam <- "traitMhlIRRI.pdf"
> showtext_auto()
> ggplot(data = mhlDT, aes(x = IRRI, y = mhlD)) +
+     geom_col(fill = "grey80") +
+     theme_classic(base_size = 18, base_family="myriad") +
+     theme(axis.text.x = element_text(angle = 90, hjust = 1, vjust = 0.5, size = 12)) +
+     ylab(expression("Hotelling T"^2)) + xlab("Trait name")
> ggsave(pdfFlNam, width = 10, height = 6, units = "in",
+             device = "pdf", useDingbats = FALSE)
> cat("\\includegraphics{", pdfFlNam, "}\n\n", sep = "")
```

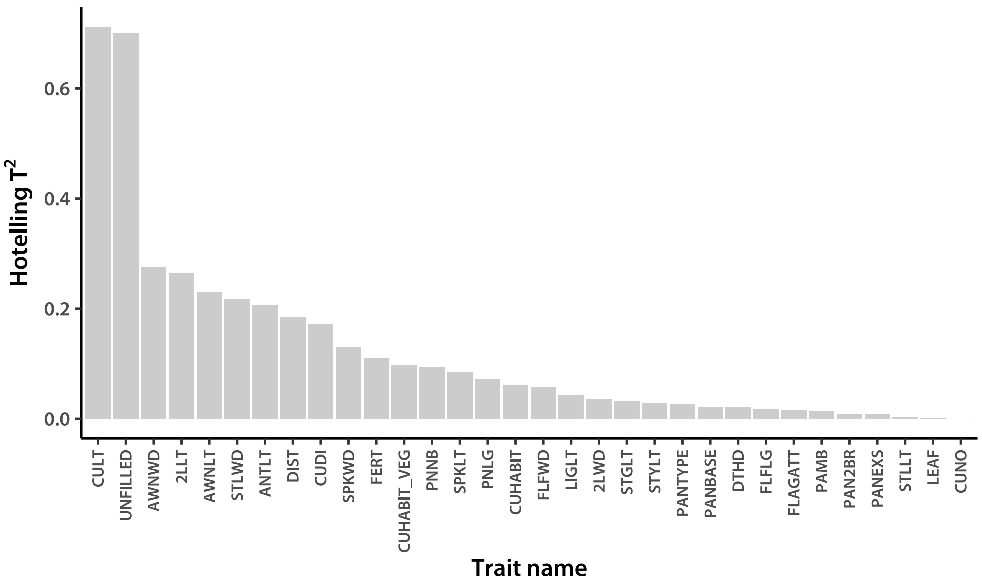

Build trait value boxplots.

```
> trtNID  <- c("NSFTV_ID", mhlDT[, as.character(IRRI)])
> traitGP <- newGrp[, ..colSubset][phenoAll[, ..trtNID], on = "NSFTV_ID"]
> traitLG <- melt(traitGP, id.vars = c("NSFTV_ID", "newGrp23"),
+             variable.name = "trait",
+             measure.vars = trtNID[-1], value.name = "value")
> traitLG <- traitLG[, newGrp23 := factor(newGrp23, levels = c("P1", "P2/P3", "P4"))]
> traitLG <- traitLG[, trait := factor(trait, levels = mhlDT[, as.character(IRRI)])]

> pdfFlNam <- "traitsIRRIbxpP4.pdf"
> showtext_auto()
> ggplot(data = traitLG, aes(x = newGrp23, y = value, fill = newGrp23)) +
+     geom_boxplot(show.legend = FALSE) +
```

```
+     scale_fill_manual(values = popGrpColors) +
+     facet_wrap(~trait, ncol = 5, scales = "free_y") +
+     theme_classic(base_size = 12, base_family = "myriad") +
+     theme(strip.background = element_rect(fill = "grey95", linetype = "blank"),
+             strip.text = element_text(size = 10, margin = margin(c(1, 0, 1, 0), "pt"))) +
+     xlab("phenotypic group")
> ggsave(pdfFlNam, width = 8, height = 10, units = "in",
+             device = "pdf", useDingbats = FALSE)
> cat("\\includegraphics[width = 0.95\\textwidth]{", pdfFlNam, "}\n\n", sep = "")
```

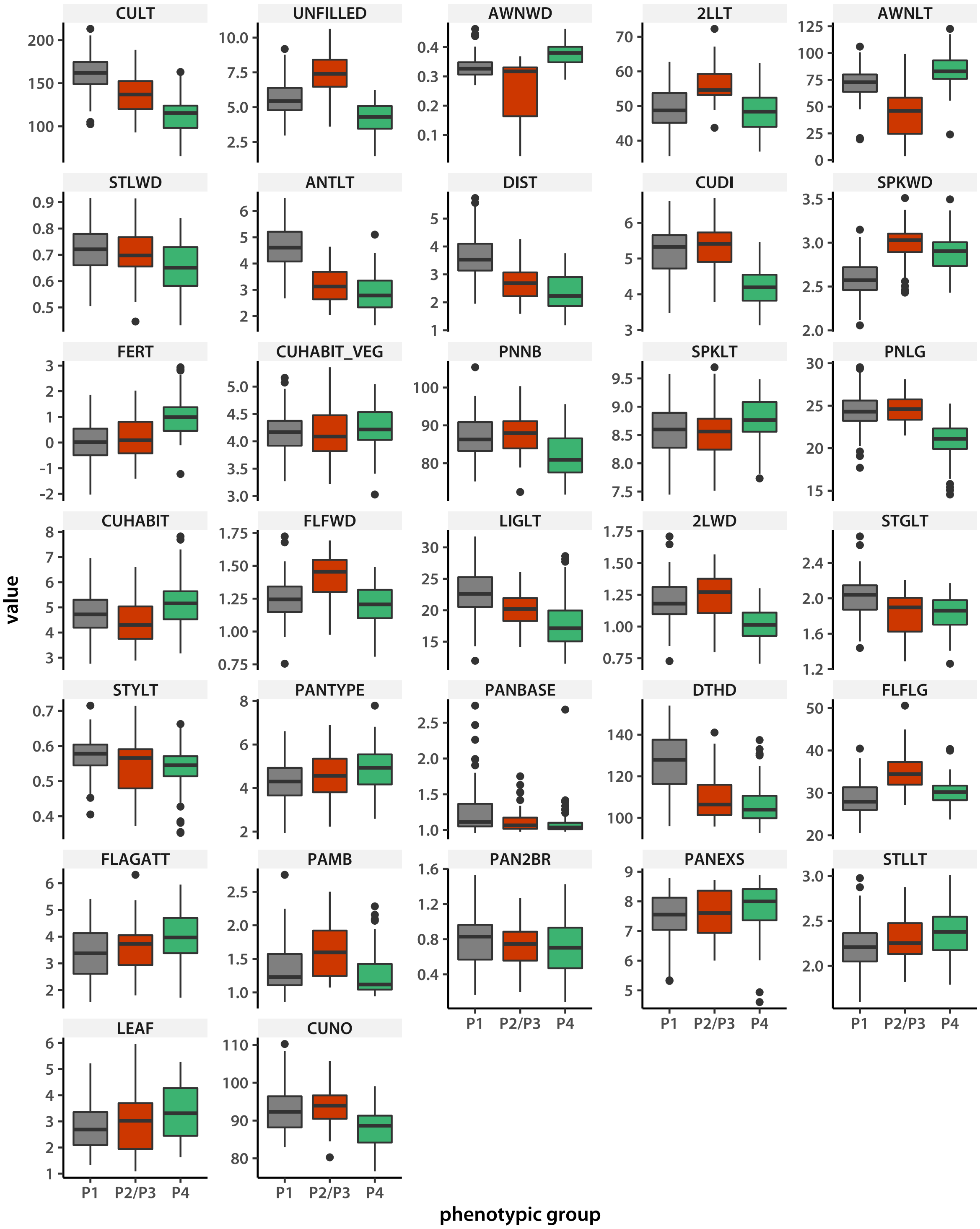

Subset of traits for the main text.

```
> pdfFlNam <- "traitsIRRIbxpP4sixTr.pdf"
> trtSubs <- c("CULT", "DTHD", "SPKWD", "ANTLT", "FERT", "PNLG")
```

```
> showtext_auto()
> ggplot(data = traitLG[trait %in% trtSubs],
+         aes(x = newGrp23, y = value, fill = newGrp23)) +
+     geom_boxplot(show.legend = FALSE) +
+     scale_fill_manual(values = popGrpColors) +
+     facet_wrap(~trait, ncol = 3, scales = "free_y") +
+     theme_classic(base_size = 14, base_family = "myriad") +
+     theme(strip.background = element_rect(fill = "grey95", linetype = "blank"),
+             strip.text = element_text(size = 12, margin = margin(c(1, 0, 1, 0), "pt"))) +
+     xlab("phenotypic group")
> ggsave(pdfFlNam, width = 8, height = 6, units = "in",
+             device = "pdf", useDingbats = FALSE)
> cat("\\includegraphics[width = 0.95\\textwidth]{", pdfFlNam, "}\n\n", sep = "")
```

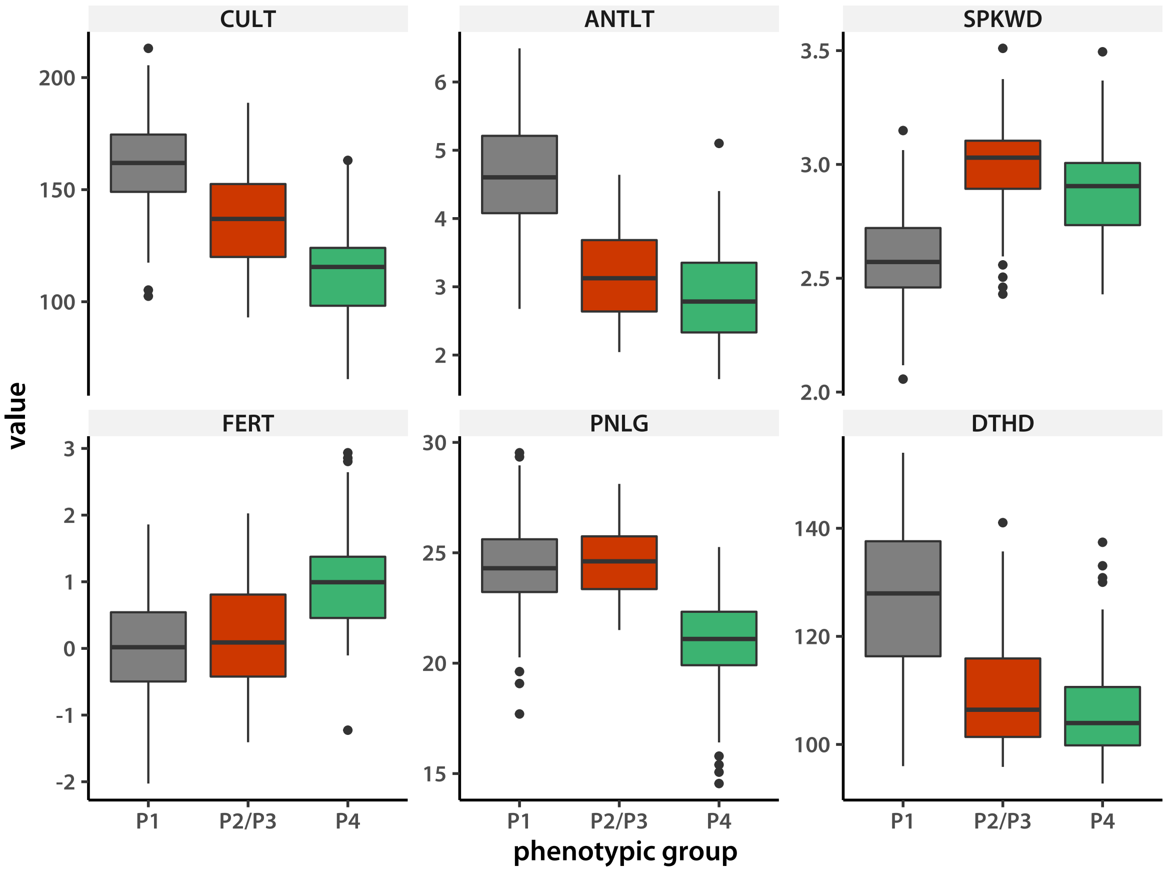

Trait coefficients of variation next.

```
> traitVar <- traitLG[, .(traitCV = sd(value)), by = .(newGrp23, trait)]
> traitVar <- traitVar[, traitCV :=
+             traitCV/rep(traitLG[, mean(value), by = trait]$V1, each = 3)]
> traitVar <- traitVar[, cvCV := sd(traitCV)/mean(traitCV), by = trait]
> traitVar <- traitVar[, trait := factor(trait, levels = mhlDT[, as.character(IRRI)])]
```

Plot.

```
> pdfFlNam <- "traitSdIRRIbpP4.pdf"
> showtext_auto()
> ggplot(data = traitVar,
+         aes(x = newGrp23, y = traitCV, fill = newGrp23)) +
+     geom_col(show.legend = FALSE) +
+     scale_fill_manual(values = popGrpColors) +
+     facet_wrap(~trait, ncol = 5, scales = "free_y") +
+     theme_classic(base_size = 14, base_family = "myriad") +
+     theme(strip.background = element_rect(fill = "grey95", linetype = "blank")) +
+     ylab("coefficient of variation") + xlab("phenotypic group")
> ggsave(pdfFlNam, width = 8, height = 10, units = "in",
+             device = "pdf", useDingbats = FALSE)
> cat("\\includegraphics[width = 0.95\\textwidth]{", pdfFlNam, "}\n\n", sep = "")
```

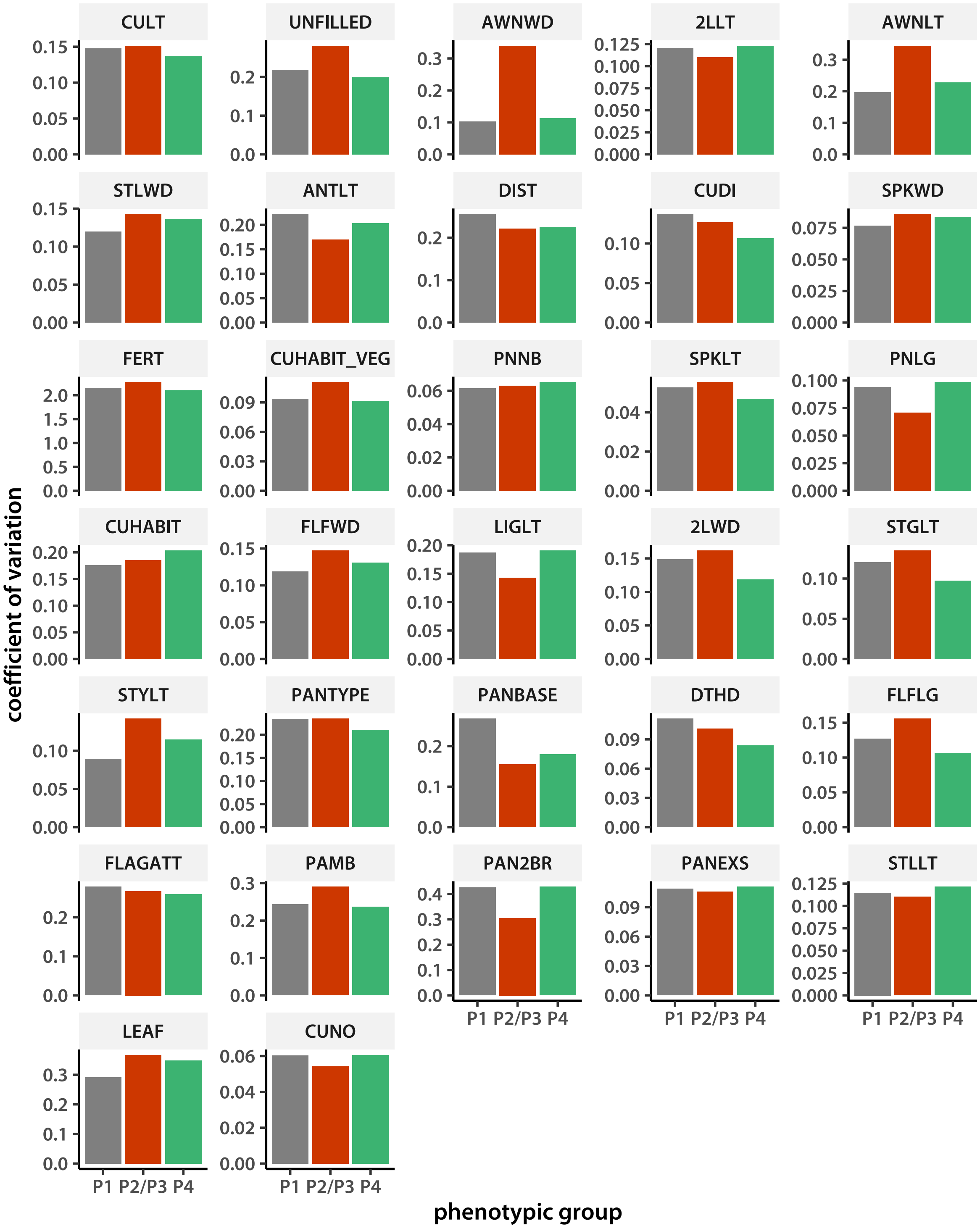

Finally, I compare trait correlations across groups.

```
> allTraits <- c("FERT", "FLFLG", "2LLT", "ANTLT", "LIGLT", "PANBASE",
+                   "UNFILLED", "DTHD", "CUDI", "CULT", "DIST", "PNLG", "FLFWD", "2LWD",
```

```
+                  "SPKLT", "STLWD", "SPKWD", "STLLT", "STYLT", "AWNLT", "AWNWD",
+                  "STGLT", "PAN2BR", "LEAF", "PANEXS", "CUHABIT",
+                  "CUHABIT_VEG", "FLAGATT", "PANTYPE", "PAMB", "CUNO", "PNNB")
> d          <- length(allTraits)
> Ngrp       <- 3
> corList    <- list(P1 = NULL, "P2/P3" = NULL, P4 = NULL)
> grpCor     <- cor(matrix(unlist(traitGP[newGrp23 == "P1", ..allTraits]),
+                          ncol = d))
> colnames(grpCor) <- allTraits
> rownames(grpCor) <- allTraits
> corList$P1       <- grpCor
> grpCor[row(grpCor) >= col(grpCor)] <- NA
> corDT <- data.table(correlation = array(grpCor),
+                      x     = paste(rep(allTraits, each = d), "P1", sep = "."),
+                      y     = rep(allTraits, times = d),
+                      group = rep("P1", times = d^2))
> corDT <- corDT[!is.na(correlation), ]
> for (grp in c("P2/P3", "P4")){
+     grpCor <- cor(matrix(unlist(traitGP[newGrp23 == grp, ..allTraits]),
+                          ncol = d))
+     colnames(grpCor) <- allTraits
+     rownames(grpCor) <- allTraits
+     corList[[grp]]   <- grpCor
+     if (grp == "P2/P3"){
+         grpCor[row(grpCor) > col(grpCor)] <- NA
+         diag(grpCor) <- 0.0
+     } else {
+         grpCor[row(grpCor) >= col(grpCor)] <- NA
+     }
+     corDT <- rbind(corDT, data.table(correlation = array(grpCor),
+                          x     = paste(rep(allTraits, each = d), grp, sep = "."),
+                          y     = rep(allTraits, times = d),
+                          group = rep(grp, times = d^2)))
+     corDT <- corDT[!is.na(correlation), ]
+     corDT[paste(y, "P1", sep = ".") == x, group := NA]
+ }
> corDT <- corDT[, x := factor(x, levels = paste(rep(allTraits, each = Ngrp),
+                                    rep(c("P1", "P2/P3", "P4"), times = d), sep = "."))]
> corDT <- corDT[, y := factor(y, levels = allTraits)]
```

Now develop a permutation test to see if such differences are expected by chance. I will randomly re-assign accessions to phenotypic groups, re-estimate among-trait correlations with each group, and report among-group ranges for each trait pair.

```
> lowTrCor <- function(mat){
+     cmat <- cor(mat)
```

```
+     return(cmat[row(cmat) < col(cmat)])
+ }
> oneSample <- function(){
+     tmpDT <- traitGP[, .(corPer = lowTrCor(.SD)),
+                 by = sample(newGrp23), .SDcols = allTraits]
+     tmpDT <- tmpDT[, traitPair := ..traitPair]
+     tmpDT <- tmpDT[, .(corSpanPer = diff(range(corPer))), by = traitPair]
+     return(tmpDT[, corSpanPer])
+ }
> getPval <- function(vec){
+     return( (sum(vec[1] <= vec[-1]) + 1)/length(vec) )
+ }
> traitGP   <- traitGP[, newGrp23 := factor(newGrp23, levels = c("P1", "P2/P3", "P4"))]
> if (file.exists("corPvalIRRI.tsv")) {
+     pValDT <- fread("corPvalIRRI.tsv")
+ } else {
+     traitPair  <- corDT[sub("\\.P.+", "", x) != y, ]
+     traitPair  <- traitPair[, traitPair := paste(sub("\\.P.+", "", x), y, sep = ".")]
+     traitPair  <- traitPair[, traitPair]
+     ltCorDT    <- traitGP[, .(corTrue = lowTrCor(.SD)),
+                    by = newGrp23, .SDcols = allTraits]
+     ltCorDT    <- ltCorDT[, traitPair := ..traitPair]
+     ltCorDT    <- ltCorDT[, .(corSpan = diff(range(corTrue))), by = traitPair]
+     spanPerMat <- replicate(9999, oneSample())
+     spanPerMat <- cbind(ltCorDT[, corSpan], spanPerMat)
+     traitPmat  <- matrix(unlist(strsplit(ltCorDT[, traitPair], "\\.")),
+                          ncol = 2, byrow = TRUE)
+     pValDT     <- data.table(x = factor(traitPmat[, 1], levels = allTraits),
+                          y = factor(traitPmat[, 2], levels = allTraits),
+                          p = apply(spanPerMat, 1, getPval))
+     pValDT <- pValDT[, trtPair := paste(x, y, sep = ".")]
+     corDT  <- corDT[, trtPair := paste(sub("\\.P.+", "", x), y, sep = ".")]
+     pValDT <- pValDT[corDT, on = "trtPair"]
+     pValDT <- pValDT[is.na(p), p := 1.0]
+     fwrite(pValDT, file = "corPvalIRRI.tsv", quote = FALSE, sep = "\t")
+ }
> pValDT <- pValDT[, i.x := factor(i.x, levels = paste(rep(allTraits, each = Ngrp),
+                                 rep(c("P1", "P2/P3", "P4"), times = d), sep = "."))]
> pValDT <- pValDT[, i.y := factor(i.y, levels = allTraits)]
```

Plot.

```
> pdfFlNam <- "traitCorIRRIp4.pdf"
> showtext_auto()
> ggplot(data = corDT, aes(x = x, y = y, fill = correlation)) +
+     geom_tile() +
```

```
+     scale_fill_gradient2(low = "royalblue4", high = "mediumvioletred",
+                 mid = "white", midpoint = 0, limit = c(-1, 1)) +
+     geom_point(data = pValDT[grep("P2/P3", i.x), ],
+                 aes(x = as.integer(i.x) - 2.0, y = i.y, color = p), size = 2) +
+     scale_color_gradient2(low = "#27015d", high = "#fbf9fd",
+                         mid = "#6801f9", midpoint = 0.15, limit = c(0, 1)) +
+     scale_x_discrete(expand = c(0.04, 0.0)) +
+     scale_y_discrete(expand = c(0.05, 0.0)) +
+     geom_segment(data = corDT[grep("P4", x), ],
+                 aes(x = as.integer(x) - 4.5, y = 0.5,
+                     xend = as.integer(x) - 4.5, yend = as.integer(y) + 0.5),
+                 color = "grey30", size = 0.75) +
+     geom_segment(data = corDT[grep("P1", x), ],
+                 aes(x = as.integer(x) - 2.5, y = as.integer(y) + 0.5,
+                     xend = max(as.integer(x)) + 0.5, yend = as.integer(y) + 0.5),
+                 color = "grey30", size = 0.75) +
+     geom_segment(x = 1.5, y = 0.5, xend = d*Ngrp - 1.5, yend = 0.5,
+                 color = "grey30", size = 0.75) +
+     geom_segment(x = d*Ngrp - 1.5, y = 0.5, xend = d*Ngrp - 1.5,
+                     yend = d - 0.5, color = "grey30", size = 0.75) +
+     theme_minimal(base_size = 18, base_family = "myriad") +
+     theme(axis.title = element_blank(),
+             axis.ticks = element_blank(),
+             axis.text = element_blank(),
+             panel.grid.major = element_blank(),
+             legend.position = c(0.32, 0.67),
+             legend.direction = "horizontal",
+             legend.justification = c(1, 0)) +
+     geom_text(data = corDT[x == paste(y, "P2/P3", sep = "."), ],
+             aes(x = x, y = y, label = y),
+             hjust = 1.0, angle = -45, fontface = "bold", size = 5) +
+     guides(fill = guide_colorbar(barwidth = 9, barheight = 1,
+                     title.position = "top", title.hjust = 0.5),
+             color = guide_colorbar(barwidth = 9, barheight = 1,
+                     title.position = "top", title.hjust = 0.5))
> ggsave(pdfFlNam, width = 12, height = 10, units = "in",
+             device = "pdf", useDingbats = FALSE)
> cat("\\includegraphics[width = 0.95\\textwidth]{", pdfFlNam, "}\n\n", sep = "")
```

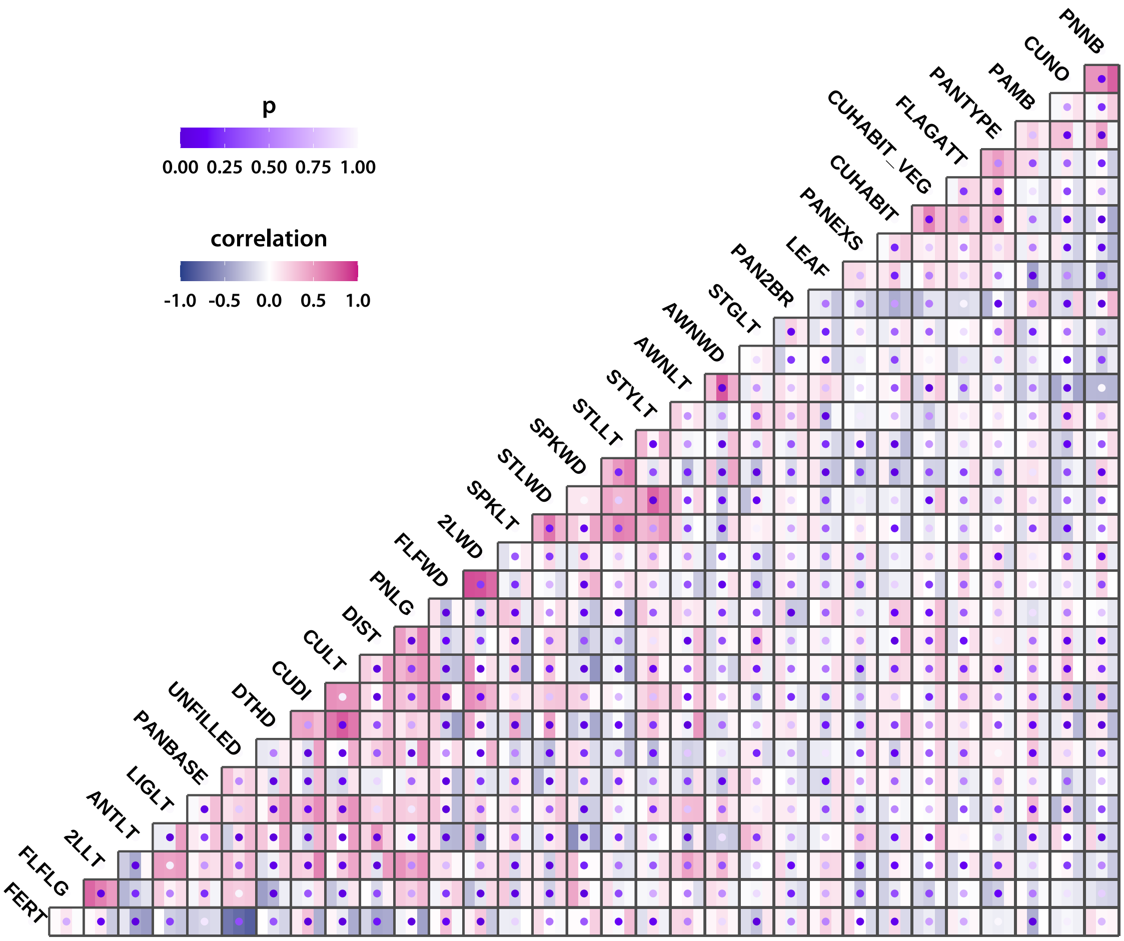

Identify trait pairs with the biggest among-group correlation variation.

```
> corPvalsP1 <- setorder(pValDT[group == "P1", ], p)
> corPvalsP1[p <= 0.001, ]

        x        y     p        trtPair correlation      i.x      i.y group
 1:  CUDI UNFILLED 1e-04  CUDI.UNFILLED  0.05404256  CUDI.P1 UNFILLED    P1
 2:  PNLG UNFILLED 1e-04  PNLG.UNFILLED  0.29123913  PNLG.P1 UNFILLED    P1
 3: SPKLT     DTHD 2e-04     SPKLT.DTHD -0.13359488 SPKLT.P1     DTHD    P1
 4:  CULT    ANTLT 3e-04     CULT.ANTLT  0.27552526  CULT.P1    ANTLT    P1
 5: FLFWD    LIGLT 3e-04    FLFWD.LIGLT  0.28709938 FLFWD.P1    LIGLT    P1
 6: FLFWD     DTHD 3e-04     FLFWD.DTHD  0.23671012 FLFWD.P1     DTHD    P1
 7:  CUDI     FERT 6e-04      CUDI.FERT  0.10307865  CUDI.P1     FERT    P1
 8: FLFWD     CULT 8e-04     FLFWD.CULT  0.33117337 FLFWD.P1     CULT    P1
 9: LIGLT    ANTLT 9e-04    LIGLT.ANTLT -0.08757841 LIGLT.P1    ANTLT    P1
10: SPKWD    ANTLT 9e-04    SPKWD.ANTLT -0.55443839 SPKWD.P1    ANTLT    P1

> corTraits  <- unique(unlist(corPvalsP1[p <= 0.001, .(x, y)]))
> mhlTraits  <- as.character(mhlDT[1:9, IRRI])
> sum(mhlTraits %in% corTraits)
```

```
[1] 4
```

```
> irriP4traits <- unique(c(corTraits, mhlTraits))
> length(mhlTraits)
```

```
[1] 9
```

```
> mhlTraits
```

```
[1] "CULT"     "UNFILLED" "AWNWD"    "2LLT"     "AWNLT"    "STLWD"    "ANTLT"
[8] "DIST"     "CUDI"
```

```
> length(corTraits)
```

```
[1] 11
```

```
> corTraits
```

```
 [1] "CUDI"     "PNLG"     "SPKLT"    "CULT"     "FLFWD"    "LIGLT"    "SPKWD"
 [8] "UNFILLED" "DTHD"     "ANTLT"    "FERT"
```

```
> length(irriP4traits)
```

```
[1] 16
```

```
> length(trtNamesNB)
```

```
[1] 32
```

```
> irriP4traits
```

```
 [1] "CUDI"     "PNLG"     "SPKLT"    "CULT"     "FLFWD"    "LIGLT"    "SPKWD"
 [8] "UNFILLED" "DTHD"     "ANTLT"    "FERT"     "AWNWD"    "2LLT"     "AWNLT"
[15] "STLWD"    "DIST"
```

```
> traitMat <- data.table(trait_acronym = sort(irriP4traits),
+                 top_by_value = ifelse(sort(irriP4traits) %in% mhlTraits, "X", ""),
+                 top_by_correlation =
+                     ifelse(sort(irriP4traits) %in% corTraits, "X", ""),
+                 union = rep("X", times = length(irriP4traits)))
> fwrite(traitMat, file = "traitSubsetsIRRI.tsv", sep = "\t", quote = FALSE)
> mhlDT <- mhlDT[, mhlD := round(mhlD, 4)]
> fwrite(mhlDT, file = "MahalanobisIRRI.tsv", sep = "\t", quote = FALSE)
```

Re-run mixture models with trait subsets, starting with $N_G = 3$, averaging among 20 runs.

```
> nReps <- 20
> Ngrp  <- 3
> Ysc   <- phenoAll[, lapply(.SD, scale), .SDcols = mhlTraits]
> names(Ysc) <- mhlTraits
> if (file.exists("ordIRRImhlP3.Rdata")) {
+     load(file = "ordIRRImhlP3.Rdata")
+ } else {
+     ordIRRImhl <- replicate(nReps,
+                    MuGaMix::quickFitModel(Ysc, mhlTraits, Ngrp, alpha0, nVBreps),
+                    simplify = FALSE)
+     save(ordIRRImhl, file = "ordIRRImhlP3.Rdata")
+ }
> addCol <- function(repInd, grpInd, Ngrp, pList){
+     pMat[, grpInd] <<- (pMat[, grpInd] +
+             pList[[repInd + 1]]$p[,
+                                indList[[repInd]][bestInd[repInd]]])
+     indList[[repInd]] <<- indList[[repInd]][-bestInd[repInd]]
+     return(NULL)
+ }
> indList <- lapply(2:nReps, function(i){1:Ngrp})
```

Anchor the first p-value matrix to the whole-data set groups.

```
> pMat <- ordIRRImhl[[1]]$p
> sum(which(pMat[, 1] > 0.9) %in% newGrp[, which(newGrp23 == "P1")])

[1] 2

> sum(which(pMat[, 2] > 0.9) %in% newGrp[, which(newGrp23 == "P1")])

[1] 4

> sum(which(pMat[, 3] > 0.9) %in% newGrp[, which(newGrp23 == "P1")])

[1] 93

> sum(which(pMat[, 2] > 0.9) %in% newGrp[, which(newGrp23 == "P4")])

[1] 55

> pMat <- pMat[, c(3, 1, 2)]
```

Average posterior probabilities across replicates.

```
> # P1
> simList <- lapply(1:(nReps - 1), bestColLst,
+           ordIRRImhl[-1], indList, pMat3[, 1], 0.9)
> bestInd <- unlist(lapply(simList, which.max))
> trash   <- sapply(1:(nReps - 1), addCol, 1,
+               Ngrp, ordIRRImhl)
> # P4
> simList <- lapply(1:(nReps - 1), bestColLst,
+           ordIRRImhl[-1], indList, pMat3[, 3], 0.9)
> bestInd <- unlist(lapply(simList, which.max))
> trash   <- sapply(1:(nReps - 1), addCol, 3,
+               Ngrp, ordIRRImhl)
> # P2/P3
> bestInd <- rep(1, nReps - 1)
> trash   <- sapply(1:(nReps - 1), addCol, 2,
+                Ngrp, ordIRRImhl)
> pMat    <- t(apply(pMat, 1, normalizeP))
> newGrp  <- newGrp[, newGrp3mhl :=
+     sapply(apply(pMat, 1, which.max), function(i){c("P1", "P2", "P4")[i]})]
```

Next, the correlation-switching traits only.

```
> Ysc   <- phenoAll[, lapply(.SD, scale), .SDcols = corTraits]
> names(Ysc) <- corTraits
> if (file.exists("ordIRRIcorP3.Rdata")) {
+     load(file = "ordIRRIcorP3.Rdata")
+ } else {
+     ordIRRIcor <- replicate(nReps,
+                     MuGaMix::quickFitModel(Ysc, corTraits, Ngrp, alpha0, nVBreps),
+                     simplify = FALSE)
+     save(ordIRRIcor, file = "ordIRRIcorP3.Rdata")
+ }
> indList <- lapply(2:nReps, function(i){1:Ngrp})
> pMat    <- ordIRRIcor[[1]]$p
> sum(which(pMat[, 1] > 0.9) %in% newGrp[, which(newGrp23 == "P1")])

[1] 1

> sum(which(pMat[, 2] > 0.9) %in% newGrp[, which(newGrp23 == "P1")])

[1] 76

> sum(which(pMat[, 3] > 0.9) %in% newGrp[, which(newGrp23 == "P1")])

[1] 20
```

```
> sum(which(pMat[, 2] > 0.9) %in% newGrp[, which(newGrp23 == "P4")])
```

```
[1] 9
```

```
> sum(which(pMat[, 1] > 0.9) %in% newGrp[, which(newGrp23 == "P4")])
```

```
[1] 41
```

```
> pMat <- pMat[, c(2, 3, 1)]
> # P1
> simList <- lapply(1:(nReps - 1), bestColLst,
+            ordIRRIcor[-1], indList, pMat3[, 1], 0.9)
> bestInd <- unlist(lapply(simList, which.max))
> trash   <- sapply(1:(nReps - 1), addCol, 1,
+                Ngrp, ordIRRIcor)
> # P4
> simList <- lapply(1:(nReps - 1), bestColLst,
+            ordIRRIcor[-1], indList, pMat3[, 3], 0.9)
> bestInd <- unlist(lapply(simList, which.max))
> trash   <- sapply(1:(nReps - 1), addCol, 3,
+                Ngrp, ordIRRIcor)
> # P2/P3
> bestInd <- rep(1, nReps - 1)
> trash   <- sapply(1:(nReps - 1), addCol, 2,
+                 Ngrp, ordIRRIcor)
> pMat    <- t(apply(pMat, 1, normalizeP))
> newGrp  <- newGrp[, newGrp3cor :=
+     sapply(apply(pMat, 1, which.max), function(i){c("P1", "P2", "P4")[i]})]
```

Finally, the union of the two groups.

```
> Ysc   <- phenoAll[, lapply(.SD, scale), .SDcols = irriP4traits]
> names(Ysc) <- irriP4traits
> if (file.exists("ordIRRImcP3.Rdata")) {
+     load(file = "ordIRRImcP3.Rdata")
+ } else {
+     ordIRRImc <- replicate(nReps,
+                    MuGaMix::quickFitModel(Ysc, irriP4traits, Ngrp, alpha0, nVBreps),
+                    simplify = FALSE)
+     save(ordIRRImc, file = "ordIRRImcP3.Rdata")
+ }
> indList <- lapply(2:nReps, function(i){1:Ngrp})
> pMat    <- ordIRRImc[[1]]$p
> sum(which(pMat[, 1] > 0.9) %in% newGrp[, which(newGrp23 == "P1")])
```

```
[1] 0
```

```
> sum(which(pMat[, 2] > 0.9) %in% newGrp[, which(newGrp23 == "P1")])

[1] 6


> sum(which(pMat[, 3] > 0.9) %in% newGrp[, which(newGrp23 == "P1")])

[1] 99


> sum(which(pMat[, 2] > 0.9) %in% newGrp[, which(newGrp23 == "P4")])

[1] 56


> sum(which(pMat[, 1] > 0.9) %in% newGrp[, which(newGrp23 == "P4")])

[1] 0


> pMat <- pMat[, c(3, 1, 2)]
> # P1
> simList <- lapply(1:(nReps - 1), bestColLst,
+           ordIRRImc[-1], indList, pMat3[, 1], 0.9)
> bestInd <- unlist(lapply(simList, which.max))
> trash   <- sapply(1:(nReps - 1), addCol, 1,
+               Ngrp, ordIRRImc)
> # P4
> simList <- lapply(1:(nReps - 1), bestColLst,
+           ordIRRImc[-1], indList, pMat3[, 3], 0.9)
> bestInd <- unlist(lapply(simList, which.max))
> trash   <- sapply(1:(nReps - 1), addCol, 3,
+               Ngrp, ordIRRImc)
> # P2/P3
> bestInd <- rep(1, nReps - 1)
> trash   <- sapply(1:(nReps - 1), addCol, 2,
+                Ngrp, ordIRRImc)
> pMat    <- t(apply(pMat, 1, normalizeP))
> newGrp  <- newGrp[, newGrp3both :=
+     sapply(apply(pMat, 1, which.max), function(i){c("P1", "P2", "P4")[i]})]
> newGrp

    NSFTV_ID genPop probability   Species genPopOr     sspFixed genPopK8 newGrp4
 1:   NID401     W4      0.5446 rufipogon       W4 O. rufipogon       W1      P4
 2:   NID402     W4      1.0000 spontanea       W4   Oryza spp.       W4      P2
 3:   NID403     W1      0.5682 rufipogon       W1 O. rufipogon       W1      P1
 4:   NID404     W4      1.0000    nivara       W4    O. nivara       W4      P2
 5:   NID405     W4      1.0000 spontanea       W4    O. nivara       W4      P4
---
```

```
218:   NID755     W1      0.9289 rufipogon       W1 O. rufipogon       W1      P1
219:   NID757     W4      0.6174    nivara       W4    O. nivara       W8      P4
220:   NID759     W1      1.0000 rufipogon       W1 O. rufipogon       W1      P1
221:   NID760     W1      0.6082    nivara       W1    O. nivara       W7      P4
222:   NID762     W4      0.8332    nivara       W4    O. nivara       W8      P4
     newGrp23     grp4p newGrp3     grp3p newGrp6 newGrp8 new_Oryza_spp     GRIN_spp
  1:       P4 0.9499856      P1 0.5500570      P4      P3  O. rufipogon O. rufipogon
  2:    P2/P3 0.9499904      P2 0.4497224      P6      P8    Oryza spp.   Oryza spp.
  3:       P1 1.0000000      P1 0.9500000      P1      P1  O. rufipogon O. rufipogon
  4:    P2/P3 0.6500000      P1 0.6000032      P2      P8     O. nivara    O. nivara
  5:       P4 1.0000000      P1 0.5500000      P4      P4    Oryza spp.   Oryza spp.
 ---
218:       P1 1.0000000      P1 0.9500000      P1      P5  O. rufipogon O. rufipogon
219:       P4 0.9499198      P1 0.5501289      P4      P5     O. nivara    O. nivara
220:       P1 1.0000000      P1 0.9500000      P1      P1  O. rufipogon O. rufipogon
221:       P4 1.0000000      P1 0.5500000      P4      P4     O. nivara    O. nivara
222:       P4 0.9499996      P1 0.5500007      P4      P4     O. nivara    O. nivara
     newGrp3mhl newGrp3cor newGrp3both
  1:         P1         P2          P1
  2:         P1         P2          P1
  3:         P1         P1          P1
  4:         P1         P2          P1
  5:         P4         P4          P4
 ---
218:         P1         P1          P1
219:         P4         P4          P4
220:         P1         P1          P1
221:         P4         P4          P4
222:         P4         P2          P4
```

```
> pGrpSubsSan <- as.data.table(make_long(newGrp,
+                       newGrp3mhl, newGrp3cor, newGrp3both, newGrp23))
> pGrpSubsSan <- pGrpSubsSan[,
+         node := factor(node, levels = c("P4", "P2/P3", "P2", "P1"))]
> pGrpSubsSan <- pGrpSubsSan[,
+         next_node := factor(next_node, levels = c("P4", "P2/P3", "P2", "P1"))]
```

Plot the relationships among subset groups.

```
> pdfFlNam <- "sankeyPGrpSubsIRRIP3.pdf"
> showtext_auto()
> ggplot(data = pGrpSubsSan, aes(x = x, next_x = next_x, node = node, next_node = next_node,
+                              fill = node, label = node)) +
+     geom_sankey(flow.alpha = 0.6, node.color = "grey30") +
+     geom_sankey_label(size = 5, color = "grey20", fill = "grey90", alpha = 0.8) +
+     scale_fill_manual(values = pGrp8Colors) +
+     scale_x_discrete(labels = c("top by\nvalue\n9 traits", "top by\ncorrelation\n11 traits",
+             "value\n+ correlation\n16 traits", "all\ndata\n32 traits")) +
```

```
+     theme_sankey(base_size = 20, base_family = "myriad") +
+     theme(strip.background = element_rect(fill = "grey95", linetype = "blank"),
+             legend.position = "none") + labs(x = NULL)
> ggsave(pdfFlNam, width = 8, height = 6, units = "in",
+             device = "pdf", useDingbats = FALSE)
> cat("\\includegraphics[width=0.9\\textwidth]{", pdfFlNam, "}\n\n", sep = "")
```

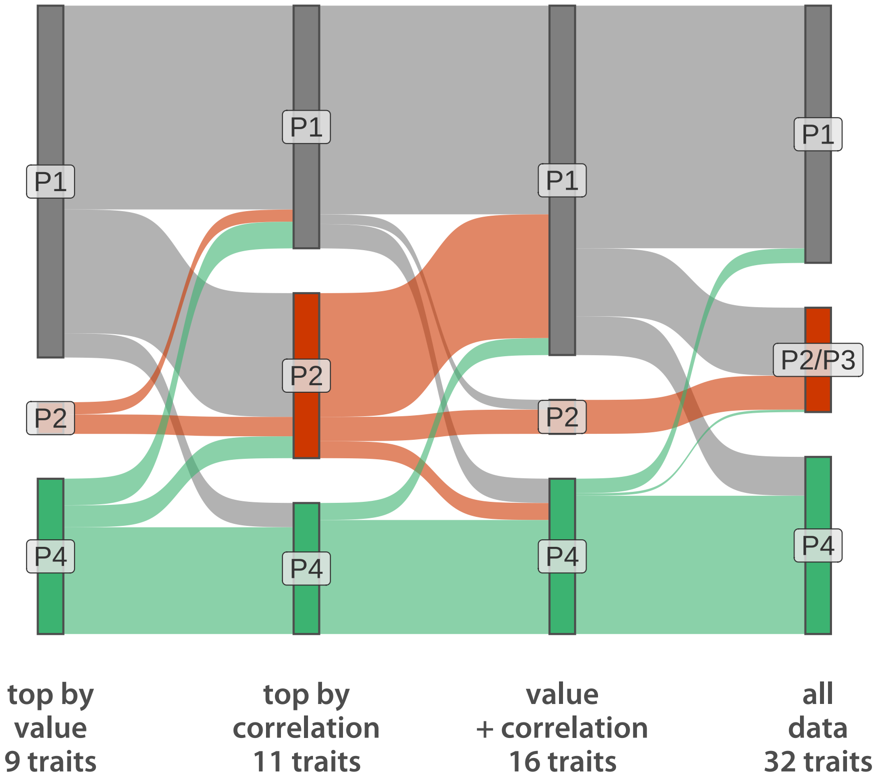

Re-run trait subsets with $N_G = 4$, again averaging among 20 runs.

```
> nReps <- 20
> Ngrp  <- 4
> Ysc   <- phenoAll[, lapply(.SD, scale), .SDcols = mhlTraits]
> names(Ysc) <- mhlTraits
> if (file.exists("ordIRRImhl.Rdata")) {
+     load(file = "ordIRRImhl.Rdata")
+ } else {
+     ordIRRImhl <- replicate(nReps,
+                     MuGaMix::quickFitModel(Ysc, mhlTraits, Ngrp, alpha0, nVBreps),
+                     simplify = FALSE)
+     save(ordIRRImhl, file = "ordIRRImhl.Rdata")
+ }
> indList <- lapply(2:nReps, function(i){1:Ngrp})
```

```
> pMat    <- ordIRRImhl[[1]]$p[, c(3, 2, 4, 1)]
> # P1
> simList <- lapply(1:(nReps - 1), bestColLst,
+           ordIRRImhl[-1], indList, pMat3[, 1], 0.9)
> bestInd <- unlist(lapply(simList, which.max))
> trash   <- sapply(1:(nReps - 1), addCol, 1,
+               Ngrp, ordIRRImhl)
> # P4
> simList <- lapply(1:(nReps - 1), bestColLst,
+           ordIRRImhl[-1], indList, pMat3[, 3], 0.9)
> bestInd <- unlist(lapply(simList, which.max))
> trash   <- sapply(1:(nReps - 1), addCol, 4,
+               Ngrp, ordIRRImhl)
> # P2
> simList <- lapply(1:(nReps - 1), bestColLst,
+            ordIRRImhl[-1], indList, pMat3[, 2], 0.9)
> bestInd <- unlist(lapply(simList, which.max))
> trash   <- sapply(1:(nReps - 1), addCol, 2,
+                Ngrp, ordIRRImhl)
> # P3
> bestInd <- rep(1, nReps - 1)
> trash   <- sapply(1:(nReps - 1), addCol, 3,
+                Ngrp, ordIRRImhl)
> pMat    <- t(apply(pMat, 1, normalizeP))
> pMat    <- pMat[, c(1, 3, 2, 4)]
> newGrp  <- newGrp[, newGrp4mhl := paste0("P", apply(pMat, 1, which.max))]
```

Next, the correlation-switching traits only.

```
> Ysc   <- phenoAll[, lapply(.SD, scale), .SDcols = corTraits]
> names(Ysc) <- corTraits
> if (file.exists("ordIRRIcor.Rdata")) {
+     load(file = "ordIRRIcor.Rdata")
+ } else {
+     ordIRRIcor <- replicate(nReps,
+                    MuGaMix::quickFitModel(Ysc, corTraits, Ngrp, alpha0, nVBreps),
+                    simplify = FALSE)
+     save(ordIRRIcor, file = "ordIRRIcor.Rdata")
+ }
> indList <- lapply(2:nReps, function(i){1:Ngrp})
> pMat    <- ordIRRIcor[[1]]$p[, c(1, 2, 4, 3)]
> # P1
> simList <- lapply(1:(nReps - 1), bestColLst,
+           ordIRRIcor[-1], indList, pMat3[, 1], 0.9)
> bestInd <- unlist(lapply(simList, which.max))
> trash   <- sapply(1:(nReps - 1), addCol, 1,
```

```
+               Ngrp, ordIRRIcor)
> # P4
> simList <- lapply(1:(nReps - 1), bestColLst,
+           ordIRRIcor[-1], indList, pMat3[, 3], 0.9)
> bestInd <- unlist(lapply(simList, which.max))
> trash   <- sapply(1:(nReps - 1), addCol, 4,
+               Ngrp, ordIRRIcor)
> # P2
> simList <- lapply(1:(nReps - 1), bestColLst,
+            ordIRRIcor[-1], indList, pMat3[, 2], 0.9)
> bestInd <- unlist(lapply(simList, which.max))
> trash   <- sapply(1:(nReps - 1), addCol, 2,
+                Ngrp, ordIRRIcor)
> # P3
> bestInd <- rep(1, nReps - 1)
> trash   <- sapply(1:(nReps - 1), addCol, 3,
+                Ngrp, ordIRRIcor)
> pMat    <- t(apply(pMat, 1, normalizeP))
> newGrp  <- newGrp[, newGrp4cor := paste0("P", apply(pMat, 1, which.max))]
```

Finally, the union of the two groups.

```
> Ysc   <- phenoAll[, lapply(.SD, scale), .SDcols = irriP4traits]
> names(Ysc) <- irriP4traits
> if (file.exists("ordIRRImc.Rdata")) {
+     load(file = "ordIRRImc.Rdata")
+ } else {
+     ordIRRImc <- replicate(nReps,
+                       MuGaMix::quickFitModel(Ysc, irriP4traits, Ngrp, alpha0, nVBreps),
+                       simplify = FALSE)
+     save(ordIRRImc, file = "ordIRRImc.Rdata")
+ }
> indList <- lapply(2:nReps, function(i){1:Ngrp})
> pMat    <- ordIRRImc[[1]]$p[, c(2, 4, 3, 1)]
> # P1
> simList <- lapply(1:(nReps - 1), bestColLst,
+           ordIRRImc[-1], indList, pMat3[, 1], 0.9)
> bestInd <- unlist(lapply(simList, which.max))
> trash   <- sapply(1:(nReps - 1), addCol, 1,
+               Ngrp, ordIRRImc)
> # P4
> simList <- lapply(1:(nReps - 1), bestColLst,
+           ordIRRImc[-1], indList, pMat3[, 3], 0.9)
> bestInd <- unlist(lapply(simList, which.max))
> trash   <- sapply(1:(nReps - 1), addCol, 4,
+               Ngrp, ordIRRImc)
```

```
> # P2
> simList <- lapply(1:(nReps - 1), bestColLst,
+            ordIRRImc[-1], indList, pMat3[, 2], 0.9)
> bestInd <- unlist(lapply(simList, which.max))
> trash   <- sapply(1:(nReps - 1), addCol, 2,
+                Ngrp, ordIRRImc)
> # P3
> bestInd <- rep(1, nReps - 1)
> trash   <- sapply(1:(nReps - 1), addCol, 3,
+                Ngrp, ordIRRImc)
> pMat    <- t(apply(pMat, 1, normalizeP))
> pMat    <- pMat[, c(1, 3, 2, 4)]
> newGrp  <- newGrp[, newGrp4both := paste0("P", apply(pMat, 1, which.max))]
> pGrpSubsSan <- as.data.table(make_long(newGrp,
+                     newGrp4mhl, newGrp4cor, newGrp4both, newGrp23))
> pGrpSubsSan <- pGrpSubsSan[,
+         node := factor(node, levels = c("P4", "P2/P3", "P3", "P2", "P1"))]
> pGrpSubsSan <- pGrpSubsSan[,
+         next_node := factor(next_node, levels = c("P4", "P2/P3", "P3", "P2", "P1"))]
```

Plot the relationships among subset groups.

```
> pdfFlNam <- "sankeyPGrpSubsIRRI.pdf"
> showtext_auto()
> ggplot(data = pGrpSubsSan, aes(x = x, next_x = next_x, node = node, next_node = next_node,
+                            fill = node, label = node)) +
+     geom_sankey(flow.alpha = 0.6, node.color = "grey30") +
+     geom_sankey_label(size = 5, color = "grey20", fill = "grey90", alpha = 0.8) +
+     scale_fill_manual(values = pGrp8Colors) +
+     scale_x_discrete(labels = c("top by\nvalue\n9 traits", "top by\ncorrelation\n11 traits",
+             "value\n+ correlation\n16 traits", "all\ndata\n32 traits")) +
+     theme_sankey(base_size = 20, base_family = "myriad") +
+     theme(strip.background = element_rect(fill = "grey95", linetype = "blank"),
+             legend.position = "none") + labs(x = NULL)
> ggsave(pdfFlNam, width = 8, height = 6, units = "in",
+             device = "pdf", useDingbats = FALSE)
> cat("\\includegraphics[width=0.9\\textwidth]{", pdfFlNam, "}\n\n", sep = "")
```

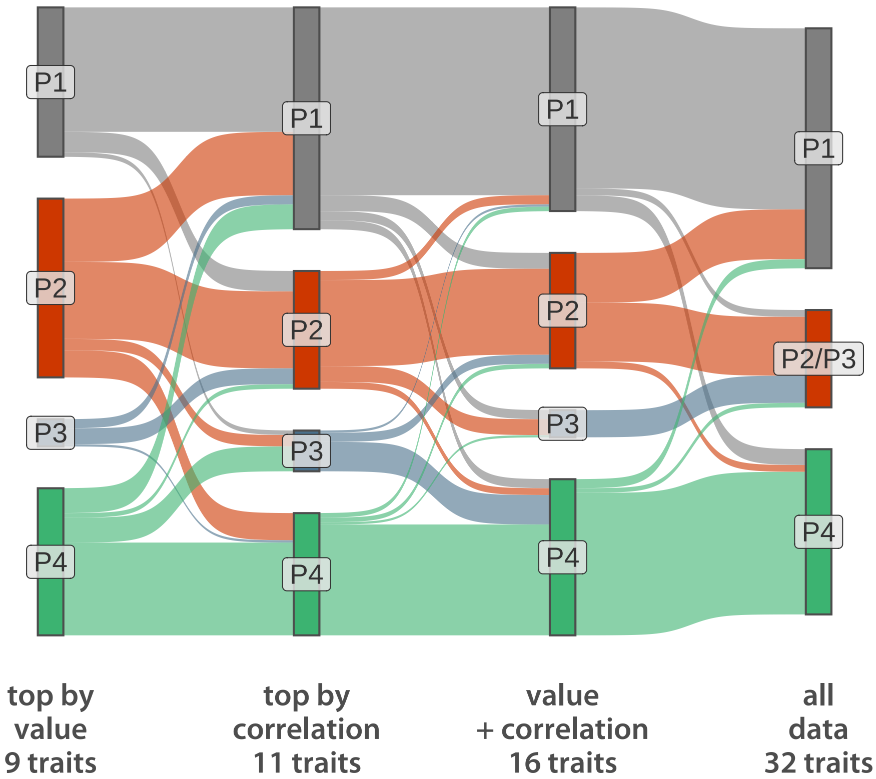

Re-create the genetic population plot with the subset groups, $N_G = 3$ first.

```
> pGrpSan      <- as.data.table(ggsankey::make_long(newGrp, GRIN_spp,
+                                                   newGrp3both, genPop))
> sankeyLevels <- c("O. nivara", "Oryza spp.", "O. rufipogon", c("P4", "P2", "P1"),
+                    paste0("W", c(5, 2, 4, 6, 1, 3)))
> pGrpSan      <- pGrpSan[, node := factor(node, levels = sankeyLevels)]
> pGrpSan      <- pGrpSan[, next_node := factor(next_node, levels = sankeyLevels)]
```

Plot.

```
> pdfFlNam <- "sankeyPGrpPopsIRRIsubsP3.pdf"
> showtext_auto()
> ggplot(data = pGrpSan, aes(x = x, next_x = next_x, node = node, next_node = next_node,
+                              fill = node, label = node)) +
+     geom_sankey(flow.alpha = 0.6, node.color = "grey30") +
+     geom_sankey_label(size = 5, color = "grey20", fill = "grey90", alpha = 0.8) +
+     scale_fill_manual(values = popGrpColors) +
+     scale_x_discrete(labels = c("species", "phenotypic\ngroups\n(16 traits)",
+                          "genetic\nsubpopulations")) +
+     theme_sankey(base_size = 20, base_family = "myriad") +
+     theme(strip.background = element_rect(fill = "grey95", linetype = "blank"),
```

```
+              legend.position = "none") + labs(x = NULL)
> ggsave(pdfFlNam, width = 8, height = 6, units = "in",
+              device = "pdf", useDingbats = FALSE)
> cat("\\includegraphics[width=0.9\\textwidth]{", pdfFlNam, "}\n\n", sep = "")
```

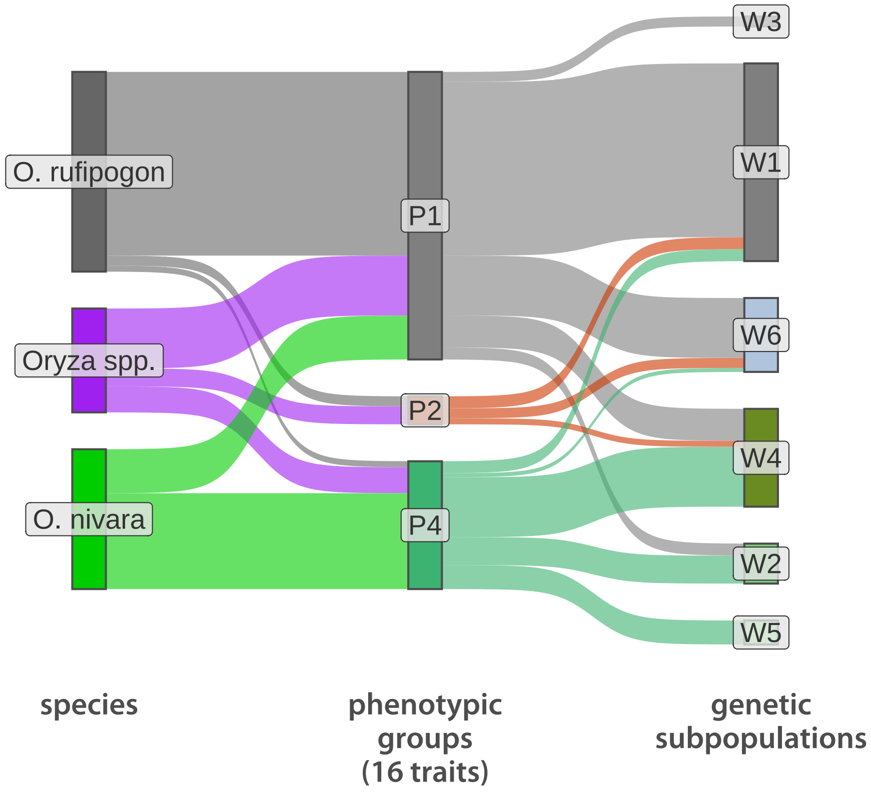

$N_G = 4$ next.

```
> pGrpSan      <- as.data.table(ggsankey::make_long(newGrp, GRIN_spp,
+                                              newGrp4both, genPop))
> sankeyLevels <- c("O. nivara", "Oryza spp.", "O. rufipogon", c("P4", "P3", "P2", "P1"),
+                  paste0("W", c(5, 2, 4, 6, 1, 3)))
> pGrpSan      <- pGrpSan[, node := factor(node, levels = sankeyLevels)]
> pGrpSan      <- pGrpSan[, next_node := factor(next_node, levels = sankeyLevels)]
```

Plot.

```
> pdfFlNam <- "sankeyPGrpPopsIRRIsubs.pdf"
> showtext_auto()
> ggplot(data = pGrpSan, aes(x = x, next_x = next_x, node = node, next_node = next_node,
+                              fill = node, label = node)) +
+     geom_sankey(flow.alpha = 0.6, node.color = "grey30") +
+     geom_sankey_label(size = 5, color = "grey20", fill = "grey90", alpha = 0.8) +
```

```
+     scale_fill_manual(values = popGrpColors) +
+     scale_x_discrete(labels = c("species", "phenotypic\ngroups\n(16 traits)",
+                            "genetic\nsubpopulations")) +
+     theme_sankey(base_size = 20, base_family = "myriad") +
+     theme(strip.background = element_rect(fill = "grey95", linetype = "blank"),
+             legend.position = "none") + labs(x = NULL)
> ggsave(pdfFlNam, width = 8, height = 6, units = "in",
+             device = "pdf", useDingbats = FALSE)
> cat("\\includegraphics[width=0.9\\textwidth]{", pdfFlNam, "}\n\n", sep = "")
```

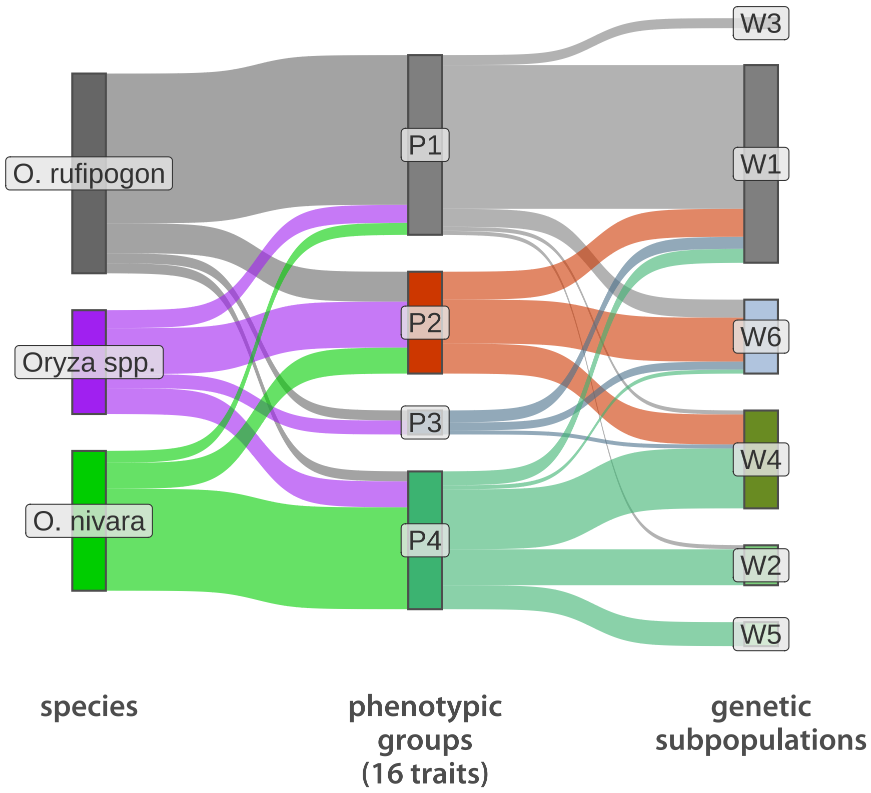

Extract *O. sativa* accessions into their own group.

```
> colSubset    <- c("NSFTV_ID", "O_sativa")
> newGrp       <- merge(newGrp, rfmix[, ..colSubset], by = "NSFTV_ID", all.x = TRUE)
> newGrp       <- newGrp[, K6sat :=
+                 ifelse(!is.na(O_sativa) & (O_sativa >= 0.5), "ADM/OSAT", genPop)]
> newGrp
```

```
   NSFTV_ID genPop probability   Species genPopOr     sspFixed genPopK8 newGrp4
1:   NID401     W4      0.5446 rufipogon       W4 O. rufipogon       W1      P4
2:   NID402     W4      1.0000 spontanea       W4   Oryza spp.       W4      P2
3:   NID403     W1      0.5682 rufipogon       W1 O. rufipogon       W1      P1
```

```
  4:   NID404     W4      1.0000    nivara       W4    O. nivara       W4      P2
  5:   NID405     W4      1.0000 spontanea       W4    O. nivara       W4      P4
 ---
218:   NID755     W1      0.9289 rufipogon       W1 O. rufipogon       W1      P1
219:   NID757     W4      0.6174    nivara       W4    O. nivara       W8      P4
220:   NID759     W1      1.0000 rufipogon       W1 O. rufipogon       W1      P1
221:   NID760     W1      0.6082    nivara       W1    O. nivara       W7      P4
222:   NID762     W4      0.8332    nivara       W4    O. nivara       W8      P4
    newGrp23     grp4p newGrp3     grp3p newGrp6 newGrp8 new_Oryza_spp     GRIN_spp
  1:       P4 0.9499856      P1 0.5500570      P4      P3  O. rufipogon O. rufipogon
  2:    P2/P3 0.9499904      P2 0.4497224      P6      P8    Oryza spp.   Oryza spp.
  3:       P1 1.0000000      P1 0.9500000      P1      P1  O. rufipogon O. rufipogon
  4:    P2/P3 0.6500000      P1 0.6000032      P2      P8     O. nivara    O. nivara
  5:       P4 1.0000000      P1 0.5500000      P4      P4    Oryza spp.   Oryza spp.
 ---
218:       P1 1.0000000      P1 0.9500000      P1      P5  O. rufipogon O. rufipogon
219:       P4 0.9499198      P1 0.5501289      P4      P5     O. nivara    O. nivara
220:       P1 1.0000000      P1 0.9500000      P1      P1  O. rufipogon O. rufipogon
221:       P4 1.0000000      P1 0.5500000      P4      P4     O. nivara    O. nivara
222:       P4 0.9499996      P1 0.5500007      P4      P4     O. nivara    O. nivara
    newGrp3mhl newGrp3cor newGrp3both newGrp4mhl newGrp4cor newGrp4both O_sativa
  1:         P1         P2          P1         P2         P4          P2     0.31
  2:         P1         P2          P1         P2         P2          P2     0.64
  3:         P1         P1          P1         P2         P1          P1     0.51
  4:         P1         P2          P1         P2         P2          P2     0.56
  5:         P4         P4          P4         P4         P4          P4     0.19
 ---
218:         P1         P1          P1         P1         P1          P1     0.02
219:         P4         P4          P4         P4         P3          P4     0.60
220:         P1         P1          P1         P2         P1          P1     0.41
221:         P4         P4          P4         P4         P4          P4     0.01
222:         P4         P2          P4         P4         P4          P4     0.28
       K6sat
  1:       W4
  2: ADM/OSAT
  3: ADM/OSAT
  4: ADM/OSAT
  5:       W4
 ---
218:       W1
219: ADM/OSAT
220:       W1
221:       W1
222:       W4
```

```
> pGrpSan      <- as.data.table(ggsankey::make_long(newGrp, GRIN_spp, newGrp23, K6sat))
> sankeyLevels <- c("O. nivara", "Oryza spp.", "O. rufipogon", c("P4", "P2/P3", "P1"),
+                      paste0("W", c(5, 2, 4)), "ADM/OSAT", paste0("W", c(6, 1, 3)))
> pGrpSan      <- pGrpSan[, node := factor(node, levels = sankeyLevels)]
> pGrpSan      <- pGrpSan[, next_node := factor(next_node, levels = sankeyLevels)]
```

Plot.

```
> pdfFlNam <- "sankeyPGrpPopsIRRIsat.pdf"
> showtext_auto()
> ggplot(data = pGrpSan, aes(x = x, next_x = next_x, node = node, next_node = next_node,
+                            fill = node, label = node)) +
+     geom_sankey(flow.alpha = 0.6, node.color = "grey30") +
+     geom_sankey_label(size = 5, color = "grey20", fill = "grey90", alpha = 0.8,
+             label = c(expression(italic("O. nivara")),
+                       expression(italic("Oryza spp.")),
+                       expression(italic("O. rufipogon")),
+                       "P4", "P2/P3", "P1",
+                       "W5", "W2", "W4", "ADM/OSAT", "W6", "W1", "W3")) +
+     scale_fill_manual(values = popGrpColors) +
+     scale_x_discrete(labels = c("species", "phenotypic\ngroups",
+                         "genetic\nsubpopulations")) +
+     theme_sankey(base_size = 20, base_family = "myriad") +
+     theme(strip.background = element_rect(fill = "grey95", linetype = "blank"),
+             legend.position = "none") + labs(x = NULL)
> ggsave(pdfFlNam, width = 8, height = 6, units = "in",
+             device = "pdf", useDingbats = FALSE)
> cat("\\includegraphics[width=0.9\\textwidth]{", pdfFlNam, "}\n\n", sep = "")
```

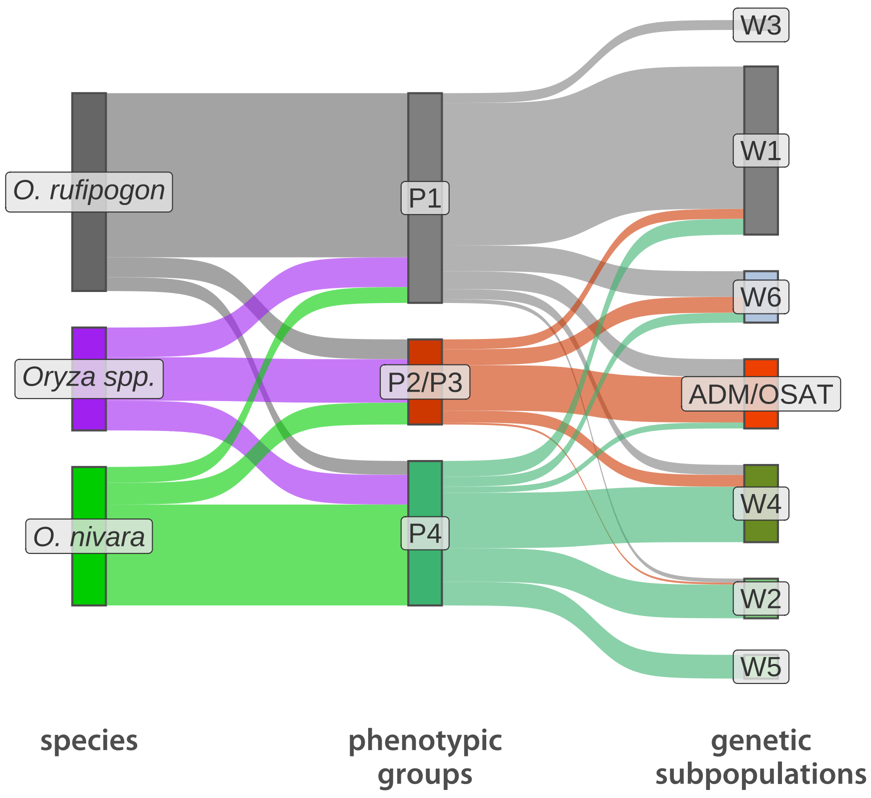

Fraction of accessions that are in the wrong P group.

```
> newGrp[(newGrp23 == "P1") &
+     (sspFixed == "O. nivara") &
+     (K6sat %in% c("W4", "W2")), .N]

[1] 4


> newGrp[(newGrp23 == "P4") &
+     (sspFixed == "O. rufipogon") &
+     (K6sat %in% c("W1", "W6")), .N]

[1] 9


> newGrp[(newGrp23 == "P1") &
+     (sspFixed == "O. nivara") &
+     (K6sat %in% c("W4", "W2")), .N]/newGrp[newGrp23 == "P1", .N]

[1] 0.03773585


> newGrp[(newGrp23 == "P4") &
+     (sspFixed == "O. rufipogon") &
+     (K6sat %in% c("W1", "W6")), .N]/newGrp[newGrp23 == "P4", .N]

[1] 0.1232877
```

Repeat for subset-based groups, starting with $N_G = 3$.

```
> pGrpSan      <- as.data.table(ggsankey::make_long(newGrp, GRIN_spp, newGrp3both, K6sat))
> sankeyLevels <- c("O. nivara", "Oryza spp.", "O. rufipogon", c("P4", "P2", "P1"),
+                   paste0("W", c(5, 2, 4)), "ADM/OSAT", paste0("W", c(6, 1, 3)))
> pGrpSan      <- pGrpSan[, node := factor(node, levels = sankeyLevels)]
> pGrpSan      <- pGrpSan[, next_node := factor(next_node, levels = sankeyLevels)]
```

Plot.

```
> pdfFlNam <- "sankeyPGrpPopsIRRIsatSubsP3.pdf"
> showtext_auto()
> ggplot(data = pGrpSan, aes(x = x, next_x = next_x, node = node, next_node = next_node,
+                            fill = node, label = node)) +
+     geom_sankey(flow.alpha = 0.6, node.color = "grey30") +
+     geom_sankey_label(size = 5, color = "grey20", fill = "grey90", alpha = 0.8,
+             label = c(expression(italic("O. nivara")),
+                       expression(italic("Oryza spp.")),
+                       expression(italic("O. rufipogon")),
+                       "P4", "P2", "P1",
```

```
+                         "W5", "W2", "W4", "ADM/OSAT", "W6", "W1", "W3")) +
+     scale_fill_manual(values = popGrpColors) +
+     scale_x_discrete(labels = c("species", "phenotypic\ngroups\n(16 traits)",
+                           "genetic\nsubpopulations")) +
+     theme_sankey(base_size = 20, base_family = "myriad") +
+     theme(strip.background = element_rect(fill = "grey95", linetype = "blank"),
+             legend.position = "none") + labs(x = NULL)
> ggsave(pdfFlNam, width = 8, height = 6, units = "in",
+             device = "pdf", useDingbats = FALSE)
> cat("\\includegraphics[width=0.9\\textwidth]{", pdfFlNam, "}\n\n", sep = "")
```

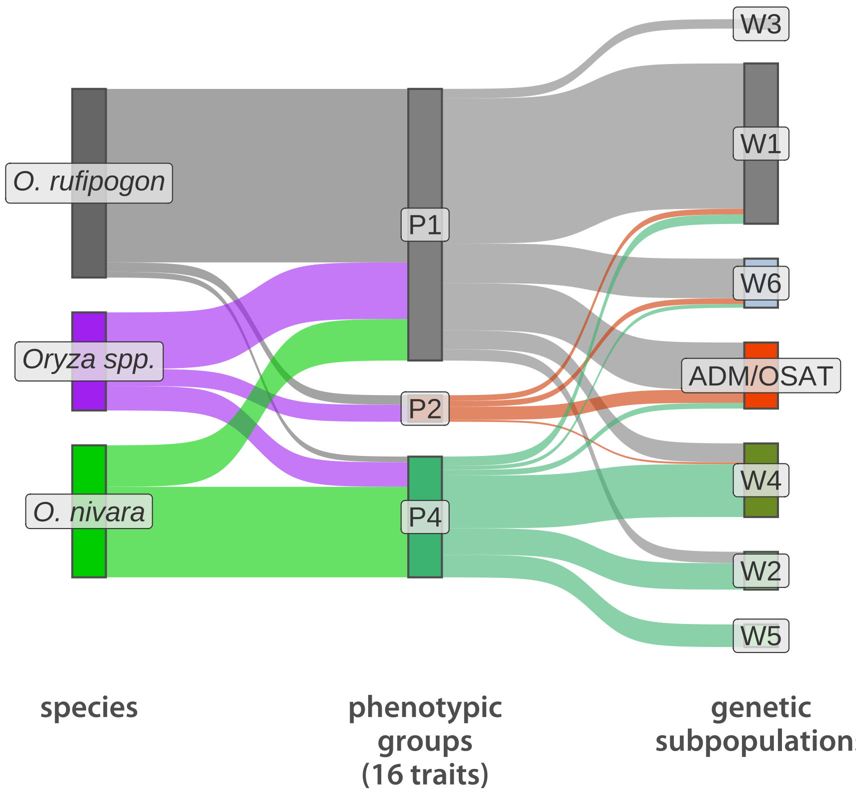

Correlation only traits next.

```
> pGrpSan      <- as.data.table(ggsankey::make_long(newGrp, GRIN_spp, newGrp3cor, K6sat))
> sankeyLevels <- c("O. nivara", "Oryza spp.", "O. rufipogon", c("P4", "P2", "P1"),
+                    paste0("W", c(5, 2, 4)), "ADM/OSAT", paste0("W", c(6, 1, 3)))
> pGrpSan      <- pGrpSan[, node := factor(node, levels = sankeyLevels)]
> pGrpSan      <- pGrpSan[, next_node := factor(next_node, levels = sankeyLevels)]
```

Plot.

```
> pdfFlNam <- "sankeyPGrpPopsIRRIsatSubsP3cor.pdf"
> showtext_auto()
```

```
> ggplot(data = pGrpSan, aes(x = x, next_x = next_x, node = node, next_node = next_node,
+                              fill = node, label = node)) +
+     geom_sankey(flow.alpha = 0.6, node.color = "grey30") +
+     geom_sankey_label(size = 5, color = "grey20", fill = "grey90", alpha = 0.8,
+             label = c(expression(italic("O. nivara")),
+                       expression(italic("Oryza spp.")),
+                       expression(italic("O. rufipogon")),
+                       "P4", "P2", "P1",
+                       "W5", "W2", "W4", "ADM/OSAT", "W6", "W1", "W3")) +
+     scale_fill_manual(values = popGrpColors) +
+     scale_x_discrete(labels = c("species", "phenotypic\ngroups\n(11 traits)",
+                         "genetic\nsubpopulations")) +
+     theme_sankey(base_size = 20, base_family = "myriad") +
+     theme(strip.background = element_rect(fill = "grey95", linetype = "blank"),
+             legend.position = "none") + labs(x = NULL)
> ggsave(pdfFlNam, width = 8, height = 6, units = "in",
+             device = "pdf", useDingbats = FALSE)
> cat("\\includegraphics[width=0.9\\textwidth]{", pdfFlNam, "}\n\n", sep = "")
```

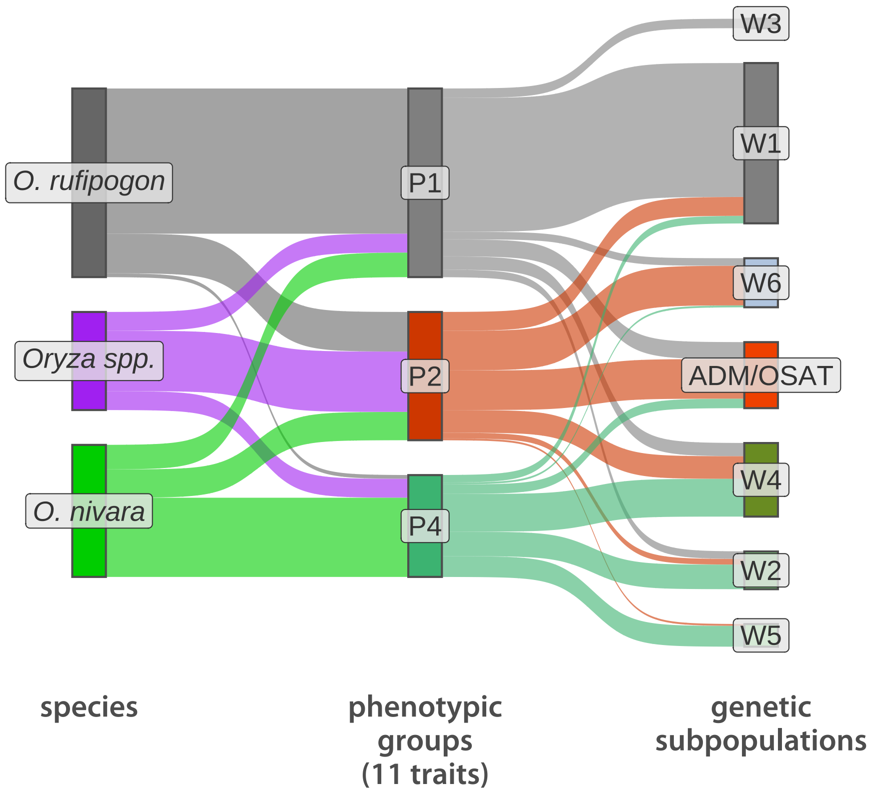

Finally, $N_G = 4$.

```
> pGrpSan      <- as.data.table(ggsankey::make_long(newGrp, GRIN_spp, newGrp4both, K6sat))
```

```
> sankeyLevels <- c("O. nivara", "Oryza spp.", "O. rufipogon", c("P4", "P2", "P3", "P1"),
+                     paste0("W", c(5, 2, 4)), "ADM/OSAT", paste0("W", c(6, 1, 3)))
> pGrpSan      <- pGrpSan[, node := factor(node, levels = sankeyLevels)]
> pGrpSan      <- pGrpSan[, next_node := factor(next_node, levels = sankeyLevels)]
```

Plot.

```
> pdfFlNam <- "sankeyPGrpPopsIRRIsatSubs.pdf"
> showtext_auto()
> ggplot(data = pGrpSan, aes(x = x, next_x = next_x, node = node, next_node = next_node,
+                              fill = node, label = node)) +
+     geom_sankey(flow.alpha = 0.6, node.color = "grey30") +
+     geom_sankey_label(size = 5, color = "grey20", fill = "grey90", alpha = 0.8,
+             label = c(expression(italic("O. nivara")),
+                       expression(italic("Oryza spp.")),
+                       expression(italic("O. rufipogon")),
+                       "P4", "P2", "P3", "P1",
+                       "W5", "W2", "W4", "ADM/OSAT", "W6", "W1", "W3")) +
+     scale_fill_manual(values = popGrpColors) +
+     scale_x_discrete(labels = c("species", "phenotypic\ngroups\n(16 traits)",
+                         "genetic\nsubpopulations")) +
+     theme_sankey(base_size = 20, base_family = "myriad") +
+     theme(strip.background = element_rect(fill = "grey95", linetype = "blank"),
+             legend.position = "none") + labs(x = NULL)
> ggsave(pdfFlNam, width = 8, height = 6, units = "in",
+             device = "pdf", useDingbats = FALSE)
> cat("\\includegraphics[width=0.9\\textwidth]{", pdfFlNam, "}\n\n", sep = "")
```

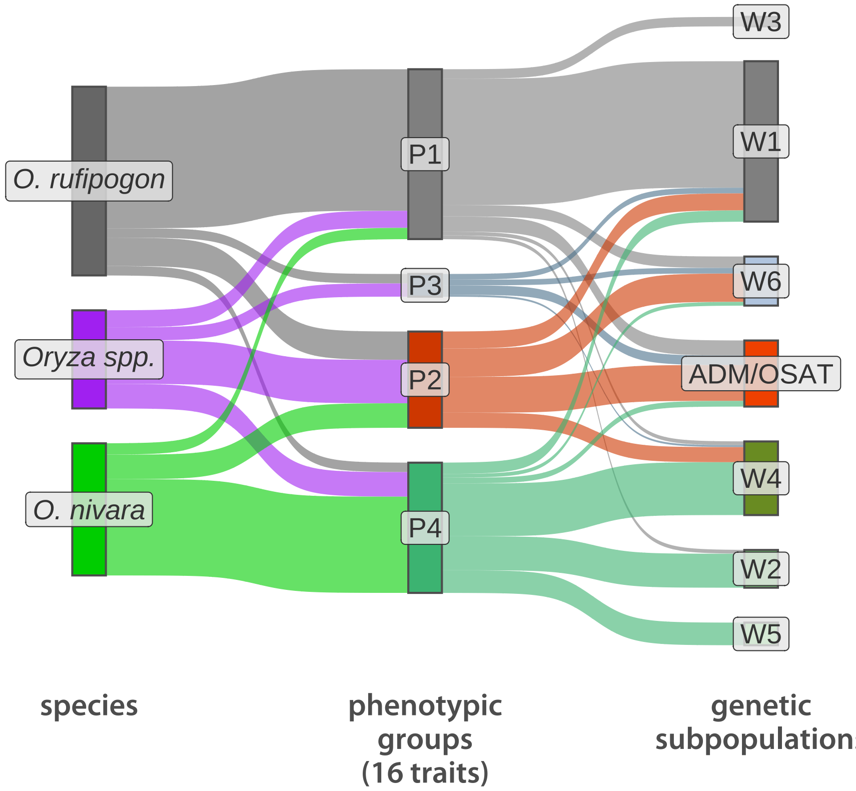

Next I make a STRUCTURE-like bar plot for *O. sativa* introgression fractions into *O. rufipogon* and *O. nivara*.

```
> sppGrp <- newGrp[, .(NSFTV_ID, GRIN_spp, O_sativa)]
> sppGrp <- sppGrp[, O_sativa := ifelse(is.na(O_sativa), 0, O_sativa)]
> sppGrp <- sppGrp[, rufiInd := ifelse(GRIN_spp == "O. rufipogon", 1 - O_sativa, 0)]
> sppGrp <- sppGrp[, nivInd := ifelse(GRIN_spp == "O. nivara", 1 - O_sativa, 0)]
> sppGrp <- sppGrp[, sponInd := ifelse(GRIN_spp == "Oryza spp.", 1 - O_sativa, 0)]
```

Check that the fractions add to 1.

```
> rowSums(sppGrp[, 3:6])
```

```
  [1] 1 1 1 1 1 1 1 1 1 1 1 1 1 1 1 1 1 1 1 1 1 1 1 1 1 1 1 1 1 1 1 1 1 1 1 1 1 1 1 1 1 1
 [43] 1 1 1 1 1 1 1 1 1 1 1 1 1 1 1 1 1 1 1 1 1 1 1 1 1 1 1 1 1 1 1 1 1 1 1 1 1 1 1 1 1 1
 [85] 1 1 1 1 1 1 1 1 1 1 1 1 1 1 1 1 1 1 1 1 1 1 1 1 1 1 1 1 1 1 1 1 1 1 1 1 1 1 1 1 1 1
[127] 1 1 1 1 1 1 1 1 1 1 1 1 1 1 1 1 1 1 1 1 1 1 1 1 1 1 1 1 1 1 1 1 1 1 1 1 1 1 1 1 1 1
[169] 1 1 1 1 1 1 1 1 1 1 1 1 1 1 1 1 1 1 1 1 1 1 1 1 1 1 1 1 1 1 1 1 1 1 1 1 1 1 1 1 1 1
[211] 1 1 1 1 1 1 1 1 1 1 1 1
```

```
> sppGrp <- melt(sppGrp, measure = c("rufiInd", "nivInd", "sponInd", "O_sativa"),
+                  variable.name = "species", value.name = "p")
```

```
> levels(sppGrp$species) <- c("O. rufipogon", "O. nivara",
+                     "Oryza spp.", "O. sativa")
> sppGrp <- sppGrp[, group := pvDT$group]
> setkey(sppGrp, NSFTV_ID, group)
> setkey(pvDT, accession, group)
> sppGrp <- sppGrp[pvDT]
```

I want to first sort by phenotyopic group (lumping P2 and P3 together), and then wihin each group by species, putting *Oryza spp.* last in P1 and first in P4.

```
> sumP   <- matrix(sppGrp$i.p, ncol = 4, byrow = TRUE)
> sumP   <- cbind(sumP[, 1],
+              rowSums(sumP[, 2:3]),
+              sumP[, 4])
> sppGrp <- sppGrp[, maxP := rep(apply(sumP, 1, max), each = 4)]
> sppGrp <- sppGrp[maxP >= 0.8, ]
> sppGrp <- sppGrp[, NSFTV_ID := factor(NSFTV_ID,
+                               levels = unique(NSFTV_ID))]
> sppGrp <- sppGrp[, orderVar1 := rep(tapply(i.p, NSFTV_ID, which.max), each = 4)]
> sppGrp <- sppGrp[, orderVar1 :=
+             ifelse(GRIN_spp == "Oryza spp.", orderVar1 + 0.5, orderVar1)]
> sppGrp <- sppGrp[, orderVar1 := ifelse(orderVar1 == 4.5, 3.5, orderVar1)]
```

Finally, within each species in a phenotypic group, I want to sort accessions by increasing *O. sativa* genome fraction in P1 and P2/P3, and decreasing fraction in P4.

```
> sppGrp <- sppGrp[, orderVar2 := ifelse(orderVar1 <= 2.5, O_sativa, 1.0 - O_sativa)]
> setorderv(sppGrp, cols = c("orderVar1", "orderVar2"), order = c(1, 1))
> sppGrp    <- sppGrp[, NSFTV_ID := factor(NSFTV_ID,
+                               levels = unique(NSFTV_ID))]
> p1range   <- range(sppGrp[group == "P1", which(i.p >= 0.8)])
> p4range   <- range(sppGrp[group == "P4", which(i.p >= 0.8)])
> p23range  <- range(sppGrp[group == "P2", which(i.p >= 0.8)])
> sppGrpCol <- c("O. sativa" = "orangered2", "O. rufipogon" = "grey40",
+         "O. nivara" = "green3", "Oryza spp." = "purple")
```

Plot the sorted data.

```
> pdfFlNam <- "structSppOsat.pdf"
> showtext_auto()
> ggplot(data = sppGrp, aes(x = NSFTV_ID, y = p, fill = species)) +
+     geom_col() +
+     scale_fill_manual(values = sppGrpCol,
+             labels = c(expression(italic("O. sativa")),
+                        expression(italic("O. rufipogon")),
+                        expression(italic("O. nivara")),
+                        expression(italic("Oryza spp.")))) +
```

```
+     geom_rect(aes(xmin = p1range[1], xmax = p1range[2],
+             ymin = -0.03, ymax = -0.01), fill = pGrp8Colors["P1"]) +
+     geom_text(aes(x = mean(p1range), y = -0.05), label = "P1",
+             size = 5, family = "myriad") +
+     geom_rect(aes(xmin = p4range[1], xmax = p4range[2],
+             ymin = -0.03, ymax = -0.01), fill = pGrp8Colors["P4"]) +
+     geom_text(aes(x = mean(p4range), y = -0.05), label = "P4",
+             size = 5, family = "myriad") +
+     geom_rect(aes(xmin = p23range[1], xmax = p23range[2],
+             ymin = -0.03, ymax = -0.01), fill = pGrp8Colors["P2/P3"]) +
+     geom_text(aes(x = mean(p23range), y = -0.05), label = "P2/P3",
+             size = 5, family = "myriad") +
+     theme_classic(base_size = 18, base_family = "myriad") +
+     theme(axis.line = element_blank(), axis.text.x = element_blank(),
+         axis.ticks.x  = element_blank(), legend.text.align = 0) +
+     xlab("accessions")
> ggsave(pdfFlNam, width = 8, height = 6, units = "in",
+             device = "pdf", useDingbats = FALSE)
> cat("\\includegraphics[width = 0.95\\textwidth]{", pdfFlNam, "}\n\n", sep = "")
```

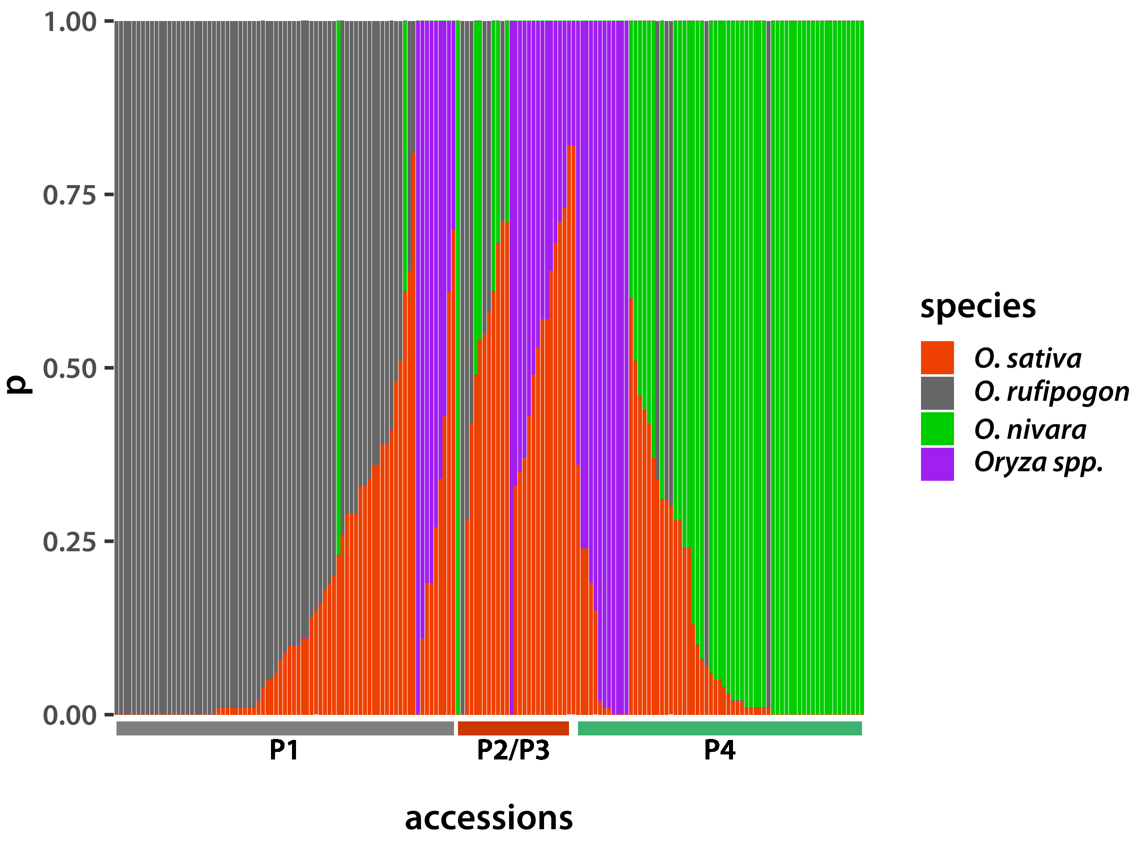

5 Cornell and Dale Bumpers data

I next add the Cornell and Dale Bumpers data. Start by reading the accession means.

```
> dbPheno <- fread("./LNmodeDB.tsv")
> cuPheno <- fread("./LNmodeCU.tsv")
```

5.1 Dale Bumpers data groups

I re-estimate group IDs averaging p-values from 20 runs as before for IRRI data.

```
> trtNamesDB <- names(dbPheno)[-1]
> trtNamesDB <- trtNamesDB[trtNamesDB != "HULGRSURFAR"]
> Ysc   <- dbPheno[, lapply(.SD, scale), .SDcols = trtNamesDB]
> Ngrp  <- 3
> nReps <- 20
> names(Ysc) <- trtNamesDB
> if (file.exists("ordDBp3.Rdata")) {
+     load(file = "ordDBp3.Rdata")
+ } else {
+     ordDB <- replicate(nReps,
+                     MuGaMix::quickFitModel(Ysc, trtNamesDB, Ngrp, alpha0, nVBreps),
+                     simplify = FALSE)
+     save(ordDB, file = "ordDBp3.Rdata")
+ }
> pMat3db <- pMat3[phenoAll$NSFTV_ID %in% dbPheno$NSFTV_ID, ]
> pMat    <- ordDB[[1]]$p
> pMat1   <- pMat[dbPheno$NSFTV_ID %in% phenoAll$NSFTV_ID, ]
> sum(which(pMat1[, 1] > 0.9) %in% which(pMat3db[, 1] > 0.6))

[1] 19

> sum(which(pMat1[, 1] > 0.9) %in% which(pMat3db[, 3] > 0.6))

[1] 15

> sum(which(pMat1[, 2] > 0.9) %in% which(pMat3db[, 1] > 0.6))

[1] 1

> sum(which(pMat1[, 2] > 0.9) %in% which(pMat3db[, 3] > 0.6))

[1] 0

> sum(which(pMat1[, 3] > 0.9) %in% which(pMat3db[, 1] > 0.6))
```

```
[1] 15
```

```
> sum(which(pMat1[, 3] > 0.9) %in% which(pMat3db[, 3] > 0.6))
```

```
[1] 29
```

```
> indList <- lapply(2:nReps, function(i){1:Ngrp})
> # P1
> simList <- lapply(1:(nReps - 1), bestColLst,
+            ordDB[-1], indList, pMat[, 1], 0.6)
> bestInd <- unlist(lapply(simList, which.max))
> trash   <- sapply(1:(nReps - 1), addCol, 1,
+                Ngrp, ordDB)
> # P4
> simList <- lapply(1:(nReps - 1), bestColLst,
+            ordDB[-1], indList, pMat[, 3], 0.6)
> bestInd <- unlist(lapply(simList, which.max))
> trash   <- sapply(1:(nReps - 1), addCol, 3,
+                Ngrp, ordDB)
> # P2
> bestInd <- rep(1, nReps - 1)
> trash   <- sapply(1:(nReps - 1), addCol, 2,
+                 Ngrp, ordDB)
> pMat    <- t(apply(pMat, 1, normalizeP))
```

Relate the trait groups ascertained from Dale Bumpers and Cornell data to the IRRI groups.

```
> dbPCP <- data.table(NSFTV_ID = dbPheno[, NSFTV_ID],
+                     db3 = paste0("P", apply(pMat, 1, which.max)))
> dbPCP <- dbPCP[, db3 := ifelse(db3 == "P3", "P4", db3)]
> dbPCP <- dbPCP[newGrp[, .(NSFTV_ID, newGrp23, newGrp4both, GRIN_spp)],
+                             on = "NSFTV_ID", nomatch = 0]
```

Repeat with Cornell data.

```
> trtNamesCU <- names(cuPheno)[-1]
> Ysc   <- cuPheno[, lapply(.SD, scale), .SDcols = trtNamesCU]
> nReps <- 20
> names(Ysc) <- trtNamesCU
> if (file.exists("ordCUp3.Rdata")) {
+     load(file = "ordCUp3.Rdata")
+ } else {
+     ordCU <- replicate(nReps,
+                     MuGaMix::quickFitModel(Ysc, trtNamesCU, Ngrp, alpha0, nVBreps),
+                     simplify = FALSE)
```

```
+     save(ordCU, file = "ordCUp3.Rdata")
+ }
> pMat3cu <- pMat3[phenoAll$NSFTV_ID %in% cuPheno$NSFTV_ID, ]
> pMat    <- ordCU[[1]]$p
> pMat1   <- pMat[cuPheno$NSFTV_ID %in% phenoAll$NSFTV_ID, ]
> sum(which(pMat1[, 1] > 0.9) %in% which(pMat3cu[, 1] > 0.6))

[1] 8

> sum(which(pMat1[, 1] > 0.9) %in% which(pMat3cu[, 3] > 0.6))

[1] 30

> sum(which(pMat1[, 2] > 0.9) %in% which(pMat3cu[, 1] > 0.6))

[1] 6

> sum(which(pMat1[, 2] > 0.9) %in% which(pMat3cu[, 3] > 0.6))

[1] 0

> sum(which(pMat1[, 3] > 0.9) %in% which(pMat3cu[, 1] > 0.6))

[1] 14

> sum(which(pMat1[, 3] > 0.9) %in% which(pMat3cu[, 3] > 0.6))

[1] 6

> pMat    <- pMat[, 3:1]
> indList <- lapply(2:nReps, function(i){1:Ngrp})
> # P1
> simList <- lapply(1:(nReps - 1), bestColLst,
+           ordCU[-1], indList, pMat[, 1], 0.6)
> bestInd <- unlist(lapply(simList, which.max))
> trash   <- sapply(1:(nReps - 1), addCol, 1,
+               Ngrp, ordCU)
> # P4
> simList <- lapply(1:(nReps - 1), bestColLst,
+           ordCU[-1], indList, pMat[, 3], 0.6)
> bestInd <- unlist(lapply(simList, which.max))
> trash   <- sapply(1:(nReps - 1), addCol, 3,
+               Ngrp, ordCU)
```

```
> # P2
> bestInd <- rep(1, nReps - 1)
> trash   <- sapply(1:(nReps - 1), addCol, 2,
+                 Ngrp, ordCU)
> pMat    <- t(apply(pMat, 1, normalizeP))
> cuPCP   <- data.table(NSFTV_ID = cuPheno[, NSFTV_ID],
+                       cu3 = paste0("P", apply(pMat, 1, which.max)))
> cuPCP   <- cuPCP[, cu3 := ifelse(cu3 == "P3", "P4", cu3)]
> cuPCP   <- cuPCP[newGrp[, .(NSFTV_ID, newGrp23, newGrp4both, GRIN_spp)],
+                               on = "NSFTV_ID", nomatch = 0]
```

Finally, I compare groups determined from the Dale Bumpers and Cornell data with the IRRI groups and species.

```
> bothPCP <- dbPCP[cuPCP[, .(NSFTV_ID, cu3)], on = "NSFTV_ID", nomatch = 0]
```

Plot $N_G = 3$ first.

```
> pGrpSan       <- as.data.table(make_long(bothPCP, GRIN_spp, db3, cu3, newGrp23))
> sankeyLevels0 <- c("O. nivara", "Oryza spp.", "O. rufipogon",
+             c("P4", "P3", "P2/P3", "P2", "P1"))
> pGrpSan       <- pGrpSan[, node := factor(node, levels = sankeyLevels0)]
> pGrpSan       <- pGrpSan[, next_node := factor(next_node, levels = sankeyLevels0)]
```

Plot.

```
> pdfFlNam <- "sankeyPGrpCUDBp3.pdf"
> showtext_auto()
> ggplot(data = pGrpSan, aes(x = x, next_x = next_x, node = node, next_node = next_node,
+                              fill = node, label = node)) +
+     geom_sankey(flow.alpha = 0.6, node.color = "grey30") +
+     geom_sankey_label(size = 5, color = "grey20", fill = "grey90", alpha = 0.8,
+             label = c(expression(italic("O. nivara")),
+                       expression(italic("Oryza spp.")),
+                       expression(italic("O. rufipogon")),
+                       "P4", "P2", "P1", "P4", "P2", "P1",
+                       "P4", "P2/P3", "P1")) +
+     scale_fill_manual(values = pGrp8Colors) +
+     scale_x_discrete(labels = c("species", "Dale\nBumpers",
+                                 "Cornell", "IRRI\nall traits")) +
+     theme_sankey(base_size = 20, base_family = "myriad") +
+     theme(strip.background = element_rect(fill = "grey95", linetype = "blank"),
+             legend.position = "none", axis.title = element_blank())
> ggsave(pdfFlNam, width = 8, height = 6, units = "in",
+             device = "pdf", useDingbats = FALSE)
> cat("\\includegraphics[width=0.9\\textwidth]{", pdfFlNam, "}\n\n", sep = "")
```

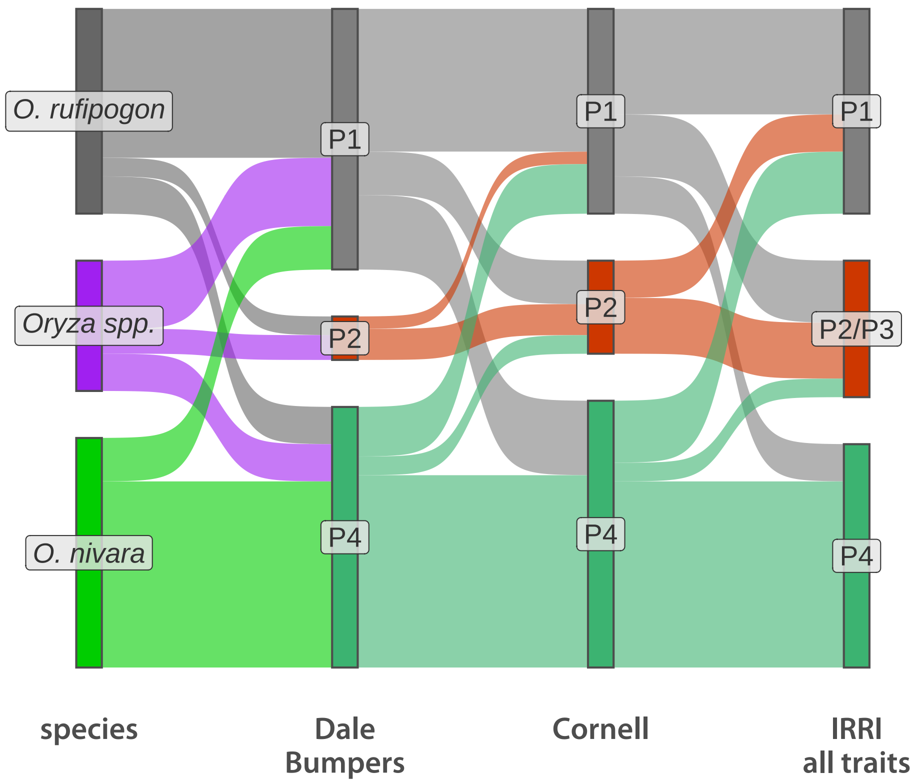

5.2 Cross-location correlations

I start by looking at correlations between values of the same traits measured at multiple locations.

```
> commonTraits <- c("CULM_ANGLE", "CUNO", "DTHD", "FLFLG",
+              "FLFWD", "FLFLWD", "PNLG", "PNNB", "PTHT", "CULT")
> trtNamesUnion <- sort(c(paste(trtNamesDB, "DB", sep = "."),
+                      paste(trtNamesCU, "CU", sep = "."),
+                      paste(trtNamesNB, "IRRI", sep = ".")))
> trtNamesUnion <- trtNamesUnion[grep(paste(commonTraits, collapse = "|"),
+                                trtNamesUnion)]
> trtNamesUnion <- c("CUHABIT.IRRI",
+                      sort(c(trtNamesUnion[-grep("CULT|FLFWD|FLFLWD", trtNamesUnion)],
+                      "FLFLWD.DB", "FLFWD.IRRI")),
+                      c("CULT.IRRI", "PBRNB.DB", "PANBASE.IRRI",
+                      "SEED_S2_BAG.CU", "SEED_S2_NONBAG.CU",
+                      "UNFILGRNB.DB", "UNFILLED.IRRI", "FERT.IRRI",
+                      "SPKWD.IRRI", "HULGRWD.DB", "SPKLT.IRRI", "HULGRLG.DB",
+                      "HULGRVOL.DB",
+                      "AWNPLU.CU", "AWNPLU.DB", "AWNWD.IRRI", "AWNLT.IRRI"))
> trtNamesUnion
```

```
 [1] "CUHABIT.IRRI"      "CULM_ANGLE.CU"     "CULM_ANGLE.DB"     "CUNO.CU"
 [5] "CUNO.IRRI"         "DTHD.CU"           "DTHD.DB"           "DTHD.IRRI"
 [9] "FLFLG.CU"          "FLFLG.DB"          "FLFLG.IRRI"        "FLFLWD.DB"
[13] "FLFWD.IRRI"        "PNLG.CU"           "PNLG.DB"           "PNLG.IRRI"
[17] "PNNB.CU"           "PNNB.DB"           "PNNB.IRRI"         "PTHT.CU"
[21] "PTHT.DB"           "CULT.IRRI"         "PBRNB.DB"          "PANBASE.IRRI"
[25] "SEED_S2_BAG.CU"    "SEED_S2_NONBAG.CU" "UNFILGRNB.DB"      "UNFILLED.IRRI"
[29] "FERT.IRRI"         "SPKWD.IRRI"        "HULGRWD.DB"        "SPKLT.IRRI"
[33] "HULGRLG.DB"        "HULGRVOL.DB"       "AWNPLU.CU"         "AWNPLU.DB"
[37] "AWNWD.IRRI"        "AWNLT.IRRI"
```

```
> irriCTnames <- sapply(strsplit(trtNamesUnion[grep("\\.IRRI", trtNamesUnion)],
+                       "\\."), `[`, 1)
> irriCTnames <- c("NSFTV_ID", irriCTnames)
> dbCTnames   <- sapply(strsplit(trtNamesUnion[grep("\\.DB", trtNamesUnion)],
+                       "\\."), `[`, 1)
> dbCTnames   <- c("NSFTV_ID", dbCTnames)
> cuCTnames   <- sapply(strsplit(trtNamesUnion[grep("\\.CU", trtNamesUnion)],
+                       "\\."), `[`, 1)
> cuCTnames   <- c("NSFTV_ID", cuCTnames)
```

Put the relevant traits together in one `data.table`.

```
> ctDT <- merge(phenoAll[, ..irriCTnames], dbPheno[, ..dbCTnames],
+             by = "NSFTV_ID", all.x = FALSE, all.y = TRUE)
> ctDT <- merge(ctDT, cuPheno[, ..cuCTnames],
+             by = "NSFTV_ID", all.x = TRUE, all.y = TRUE)
> names(ctDT) <- c("NSFTV_ID", paste(irriCTnames[-1], "IRRI", sep = "."),
+                 paste(dbCTnames[-1], "DB", sep = "."),
+                 paste(cuCTnames[-1], "CU", sep = "."))
> trtNamesUnion <- c("NSFTV_ID", trtNamesUnion)
> ctDT <- ctDT[, ..trtNamesUnion]
> trtNamesUnion <- trtNamesUnion[-1]
```

Calculate correlations and prepare for plotting.

```
> cmnTrtCor    <- cor(as.matrix(ctDT[, ..trtNamesUnion]),
+                       use = "pairwise.complete.obs")
> cmnTrtCor[row(cmnTrtCor) > col(cmnTrtCor)] <- NA
> diag(cmnTrtCor) <- 0.0
> d.cor <- ncol(cmnTrtCor)
> corDT <- data.table(correlation = array(cmnTrtCor),
+                       x = rep(trtNamesUnion, each = d.cor),
+                       y = rep(trtNamesUnion, times = d.cor))
> corDT <- corDT[!is.na(correlation), ]
> corDT <- corDT[, x := factor(x, levels = trtNamesUnion)]
> corDT <- corDT[, y := factor(y, levels = trtNamesUnion)]
```

```
> fwrite(corDT[x != y, .(correlation = round(correlation, 3), x, y)],
+     file = "crossLocationCorrelations.tsv",
+     sep = "\t", quote = FALSE)
```

Plot.

```
> pdfFlNam <- "commonTraitCorALL.pdf"
> showtext_auto()
> ggplot(data = corDT, aes(x = x, y = y, fill = correlation)) +
+     geom_tile() +
+     scale_fill_gradient2(low = "royalblue4", high = "mediumvioletred",
+                 mid = "white", midpoint = 0, limit = c(-1, 1)) +
+     scale_x_discrete(expand = c(0.2, 0.0)) +
+     scale_y_discrete(expand = c(0.2, 0.0)) +
+     theme_minimal(base_size = 18, base_family = "myriad") +
+     theme(axis.title = element_blank(),
+             axis.ticks = element_blank(),
+             axis.text = element_blank(),
+             panel.grid.major = element_blank(),
+             legend.position = c(0.32, 0.67),
+             legend.direction = "horizontal",
+             legend.justification = c(1, 0)) +
+     geom_text(data = corDT[x == y, ],
+             aes(x = x, y = y, label = y),
+             hjust = 1.0, angle = -45, fontface = "bold", size = 4) +
+     guides(fill = guide_colorbar(barwidth = 9, barheight = 1,
+                     title.position = "top", title.hjust = 0.5)) +
+     coord_fixed()
> ggsave(pdfFlNam, width = 8, height = 8, units = "in",
+             device = "pdf", useDingbats = FALSE)
> cat("\\includegraphics[width = 0.95\\textwidth]{", pdfFlNam, "}\n\n", sep = "")
```

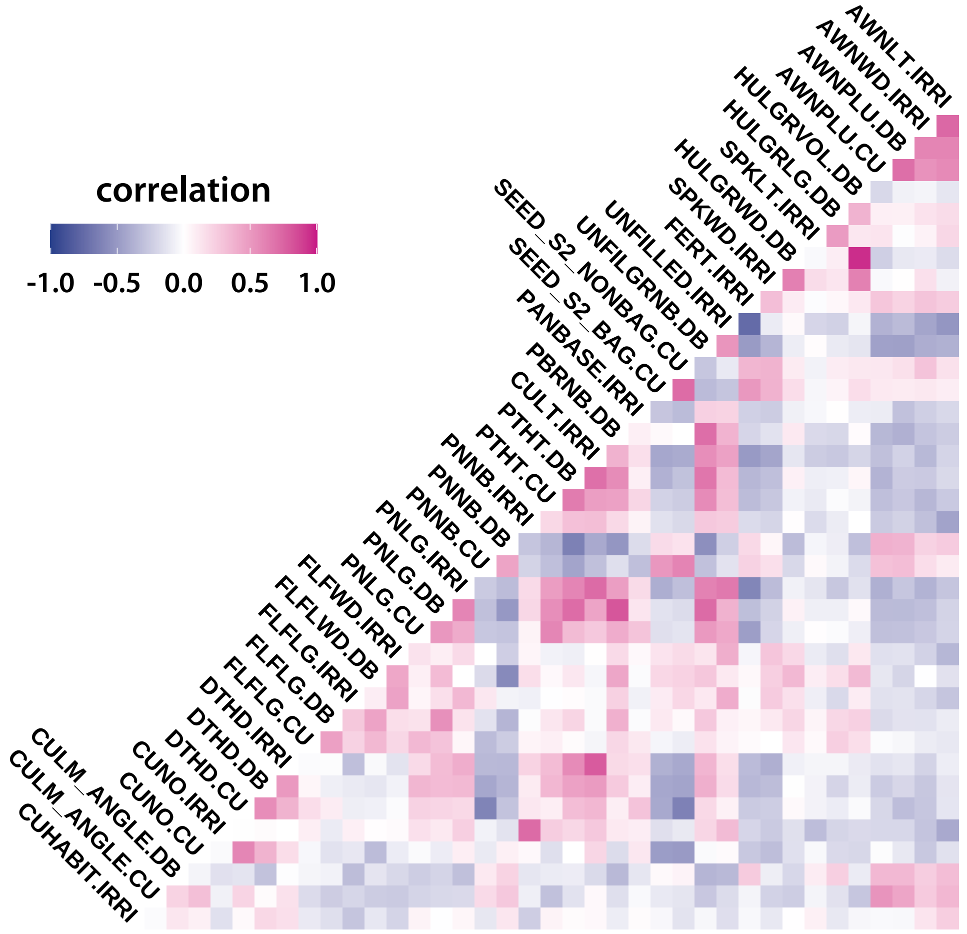

5.3 Relationships to IRRI groups

I start by ranking Dale Bumpers and Cornell traits by value against IRRI-determined groups.

```
> N       <- dbPheno[, .N]
> pMatDB  <- as.data.table(pMat3[, -3])
> pMatDB  <- pMatDB[, NSFTV_ID := newGrp[, NSFTV_ID]]
> pMatDB  <- dbPheno[pMatDB, on = "NSFTV_ID", nomatch = 0]
> YscDB   <- dbPheno[, lapply(.SD, scale), .SDcols = trtNamesDB]
> YscDB   <- matrix(unlist(YscDB), nrow = N)
> YscDB   <- YscDB[dbPheno[, NSFTV_ID] %in% pMatDB[, NSFTV_ID], ]
> YscDB   <- scale(YscDB, scale = FALSE)
```

```
> pMatDB  <- as.matrix(pMatDB[, paste0("V", 1:2)])
> Psc     <- scale(pMatDB, scale = FALSE)
> betaEst <- solve(crossprod(YscDB), crossprod(YscDB, Psc))
> Rsd     <- Psc - YscDB%*%betaEst
> Sest    <- crossprod(Rsd)
> Sest    <- chol2inv(chol(Sest))
> mhlDist <- apply(betaEst, 1, mhl, Sest)
> XtX     <- colSums(YscDB*YscDB)
> mhlDist <- mhlDist*XtX
> mhlDT   <- data.table(mhlD = mhlDist, traits = trtNamesDB)
> mhlDT   <- setorder(mhlDT, -mhlD)
> mhlDT   <- mhlDT[, traits := factor(traits, levels = unique(traits))]
> mhlDT
```

```
            mhlD       traits
 1: 1.7003361696     HULGRVOL
 2: 1.1464494880      HULGRWD
 3: 0.2232899755         PTHT
 4: 0.2169209955      HULGRLG
 5: 0.1432562610       AWNPLU
 6: 0.0459394787      HULGRCO
 7: 0.0451933409 STOLON_BINARY
 8: 0.0374498096       FLFLWD
 9: 0.0339855418    UNFILGRNB
10: 0.0297355014         PNNB
11: 0.0261613616         PNLG
12: 0.0209333095        PBRNB
13: 0.0202108247   CULM_ANGLE
14: 0.0112852894  SDSH_BINARY
15: 0.0094595861         DTHD
16: 0.0077974376        FLFLG
17: 0.0005277351   AWN_BINARY
```

```
> fwrite(mhlDT, file = "./MahalanobisDB.tsv", sep = "\t", quote = FALSE)
```

Plot the sorted distances.

```
> pdfFlNam <- "traitMhlP4DB.pdf"
> showtext_auto()
> ggplot(data = mhlDT, aes(x = traits, y = mhlD)) +
+     geom_col(fill = "grey80") +
+     theme_classic(base_size = 18, base_family="myriad") +
+     theme(axis.text.x = element_text(angle = 45, hjust = 1,
+                              vjust = 1, size = 12)) +
+     ylab(expression("Hotelling T"^2)) + xlab("Trait name")
> ggsave(pdfFlNam, width = 8, height = 6, units = "in",
+             device = "pdf", useDingbats = FALSE)
> cat("\\includegraphics{", pdfFlNam, "}\n\n", sep = "")
```

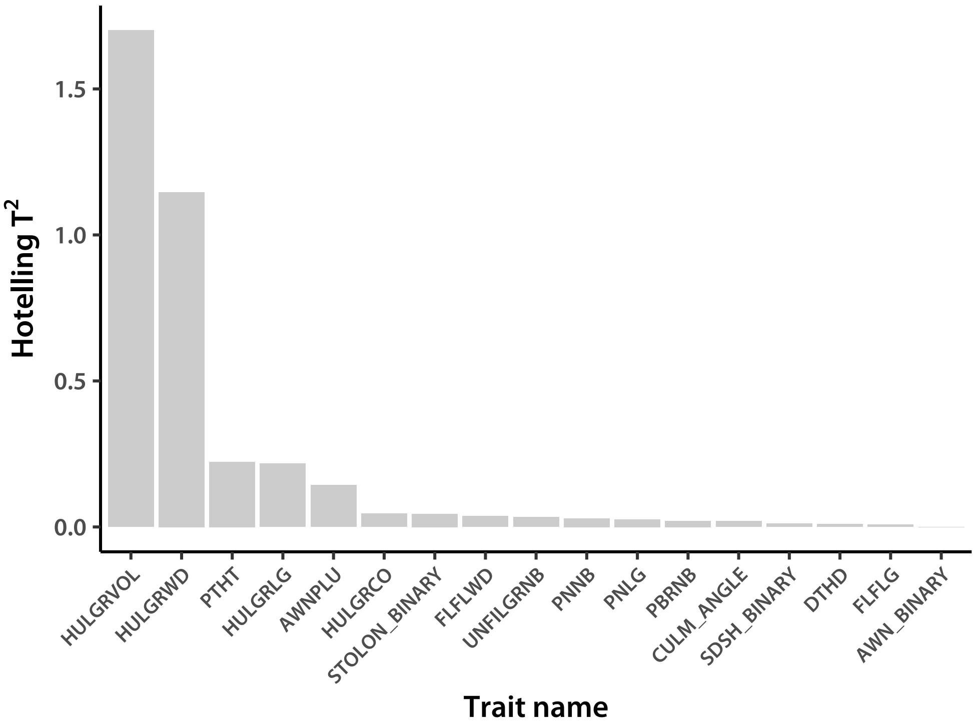

Build trait value boxplots.

```
> colSubs <- c("NSFTV_ID", "newGrp23", trtNamesDB)
> dbPCP   <- dbPCP[dbPheno, on = "NSFTV_ID", nomatch = 0]
> traitLG <- melt(dbPCP, id.vars = c("NSFTV_ID", "newGrp23"),
+              variable.name = "trait",
+              measure.vars = colSubs[-(1:2)], value.name = "value")
> traitLG <- traitLG[, newGrp23 := factor(newGrp23, levels = c("P1", "P2/P3", "P4"))]
> traitLG <- traitLG[, trait := factor(trait, levels = mhlDT$traits)]

> pdfFlNam <- "traitsDBbxpP4.pdf"
> showtext_auto()
> ggplot(data = traitLG, aes(x = newGrp23, y = value, fill = newGrp23)) +
+     geom_boxplot(show.legend = FALSE) +
+     scale_fill_manual(values = popGrpColors) +
+     facet_wrap(~trait, ncol = 4, scales = "free_y") +
+     theme_classic(base_size = 12, base_family = "myriad") +
+     theme(strip.background = element_rect(fill = "grey95", linetype = "blank"),
+             strip.text = element_text(size = 10, margin = margin(c(1, 0, 1, 0), "pt"))) +
+     xlab("phenotypic group")
> ggsave(pdfFlNam, width = 8, height = 10, units = "in",
+             device = "pdf", useDingbats = FALSE)
> cat("\\includegraphics[width = 0.95\\textwidth]{", pdfFlNam, "}\n\n", sep = "")
```

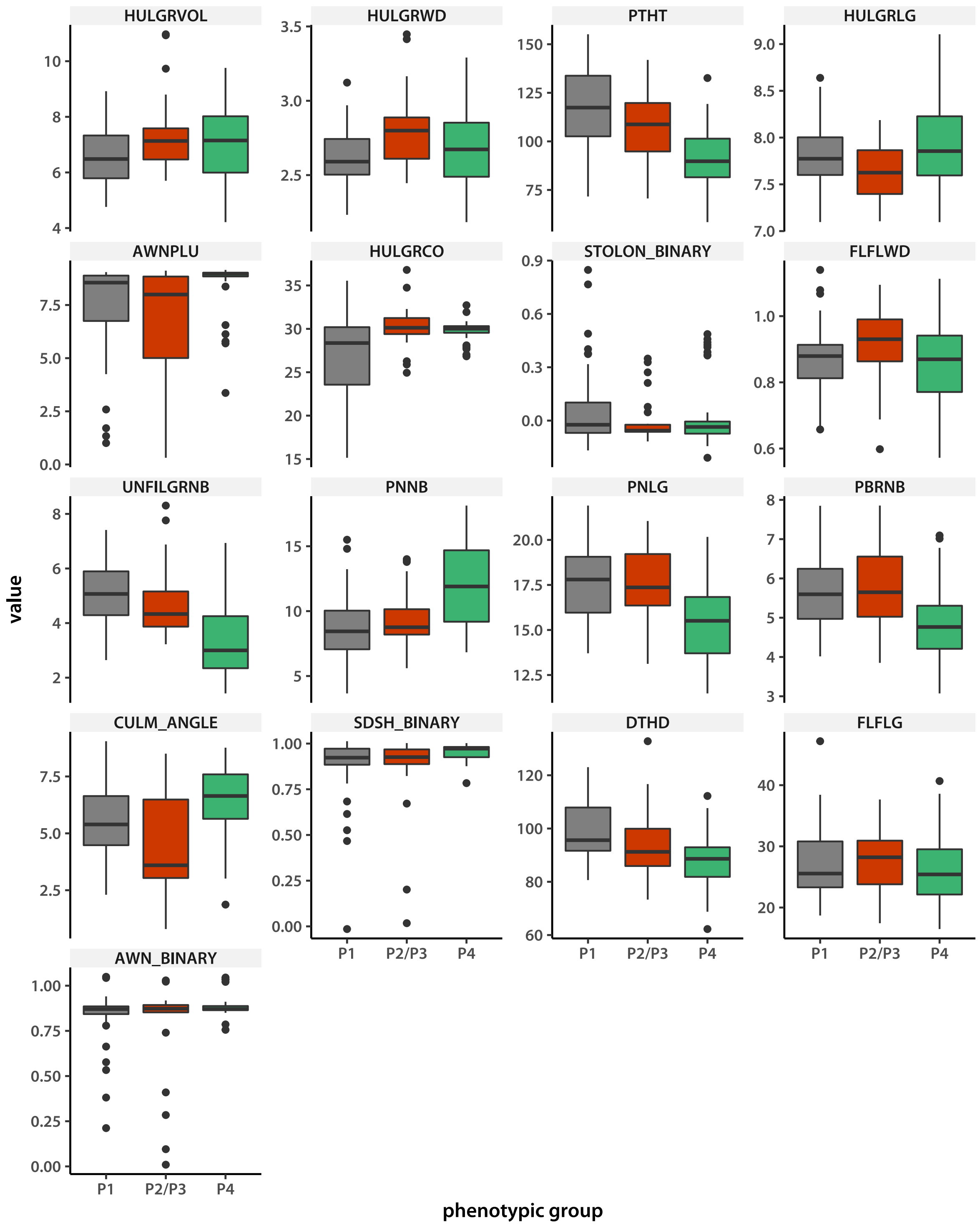

Plot the grain trait pair.

```
> pdfFlNam <- "grainsDB.pdf"
> showtext_auto()
```

```
> ggplot(data = dbPCP, aes(x = HULGRVOL, y = HULGRWD, color = newGrp23)) +
+     geom_point(size = 2) +
+     scale_color_manual(values = popGrpColors[c(1, 2, 5)]) +
+     theme_classic(base_size = 16, base_family = "myriad")
> ggsave(pdfFlNam, width = 10, height = 8, units = "in",
+             device = "pdf", useDingbats = FALSE)
> cat("\\includegraphics[width = 0.95\\textwidth]{", pdfFlNam, "}\n\n", sep = "")
```

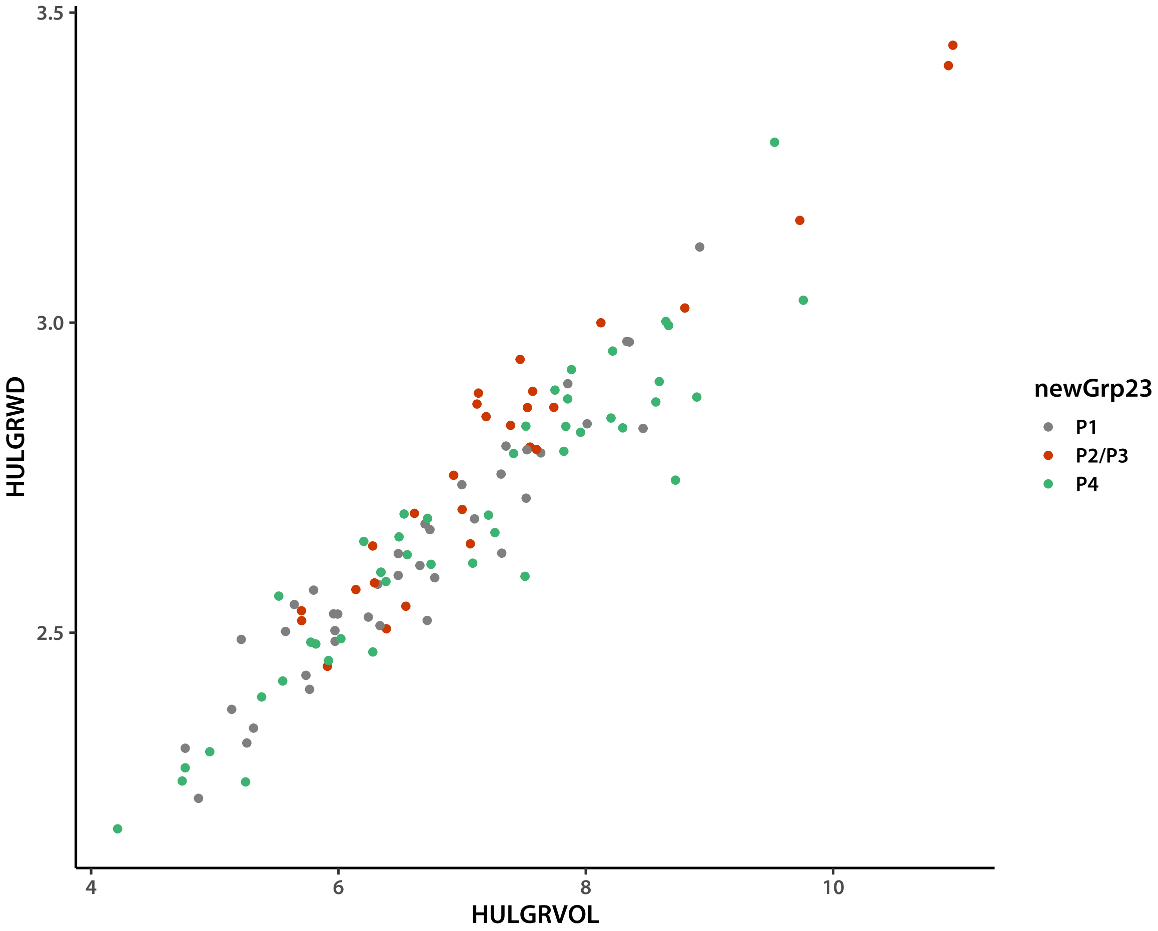

Trait coefficients of variation next.

```
> traitVar <- traitLG[, .(traitCV = sd(value)), by = .(newGrp23, trait)]
> traitVar <- traitVar[, traitCV :=
+             traitCV/rep(traitLG[, mean(value), by = trait]$V1, each = 3)]
> traitVar <- traitVar[, cvCV := sd(traitCV)/mean(traitCV), by = trait]
> traitVar <- traitVar[, trait := factor(trait, levels = mhlDT$traits)]
```

Plot.

```
> pdfFlNam <- "traitSdDBbpP4.pdf"
> showtext_auto()
```

```
> ggplot(data = traitVar,
+         aes(x = newGrp23, y = traitCV, fill = newGrp23)) +
+     geom_col(show.legend = FALSE) +
+     scale_fill_manual(values = popGrpColors) +
+     facet_wrap(~trait, ncol = 4, scales = "free_y") +
+     theme_classic(base_size = 14, base_family = "myriad") +
+     theme(strip.background = element_rect(fill = "grey95", linetype = "blank")) +
+     ylab("coefficient of variation") + xlab("phenotypic group")
> ggsave(pdfFlNam, width = 8, height = 10, units = "in",
+             device = "pdf", useDingbats = FALSE)
> cat("\\includegraphics[width = 0.95\\textwidth]{", pdfFlNam, "}\n\n", sep = "")
```

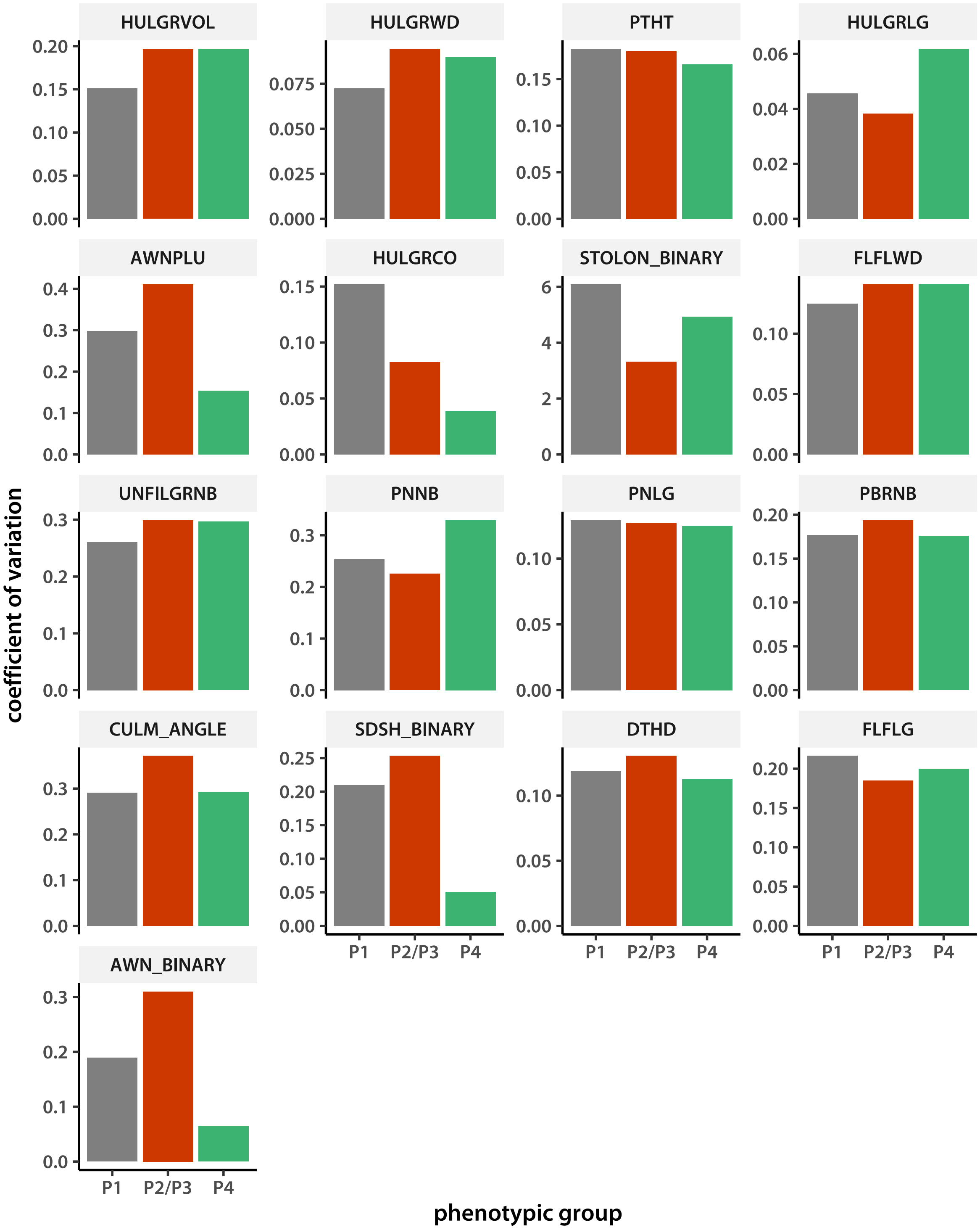

Now repeat this for Cornell data.

```
> N       <- cuPheno[, .N]
> pMatCU  <- as.data.table(pMat3[, -3])
```

```
> pMatCU  <- pMatCU[, NSFTV_ID := newGrp[, NSFTV_ID]]
> pMatCU  <- cuPheno[pMatCU, on = "NSFTV_ID", nomatch = 0]
> YscCU   <- cuPheno[, lapply(.SD, scale), .SDcols = trtNamesCU]
> YscCU   <- matrix(unlist(YscCU), nrow = N)
> YscCU   <- YscCU[cuPheno[, NSFTV_ID] %in% pMatCU[, NSFTV_ID], ]
> YscCU   <- scale(YscCU, scale = FALSE)
> pMatCU  <- as.matrix(pMatCU[, paste0("V", 1:2)])
> Psc     <- scale(pMatCU, scale = FALSE)
> betaEst <- solve(crossprod(YscCU), crossprod(YscCU, Psc))
> Rsd     <- Psc - YscCU%*%betaEst
> Sest    <- crossprod(Rsd)
> Sest    <- chol2inv(chol(Sest))
> mhlDist <- apply(betaEst, 1, mhl, Sest)
> XtX     <- colSums(YscCU*YscCU)
> mhlDist <- mhlDist*XtX
> mhlDT   <- data.table(mhlD = mhlDist, traits = trtNamesCU)
> mhlDT   <- setorder(mhlDT, -mhlD)
> mhlDT   <- mhlDT[, traits := factor(traits, levels = unique(traits))]
> mhlDT
```

```
           mhlD             traits
 1: 0.245946271     SEED_S2_NONBAG
 2: 0.217436802             AWNPLU
 3: 0.166729004        SEED_S2_BAG
 4: 0.152924304         AWN_BINARY
 5: 0.080647756               PTHT
 6: 0.079428412      STOLON_BINARY
 7: 0.076178071        SDSH_BINARY
 8: 0.064867156               CUNO
 9: 0.064208859 PERICARPCOL_BINARY
10: 0.060365838              FLFLG
11: 0.056150501         CULM_ANGLE
12: 0.032442568     STEMCOL_BINARY
13: 0.028873291               DTHD
14: 0.024415857               PNNB
15: 0.022886391              HULCL
16: 0.005340721               PNLG
```

```
> fwrite(mhlDT, file = "./MahalanobisCU.tsv", sep = "\t", quote = FALSE)
```

Plot the sorted distances.

```
> pdfFlNam <- "traitMhlP4CU.pdf"
> showtext_auto()
> ggplot(data = mhlDT, aes(x = traits, y = mhlD)) +
+     geom_col(fill = "grey80") +
+     theme_classic(base_size = 18, base_family="myriad") +
```

```
+     theme(axis.text.x = element_text(angle = 45, hjust = 1,
+                              vjust = 1, size = 12)) +
+     ylab(expression("Hotelling T"^2)) + xlab("Trait name")
> ggsave(pdfFlNam, width = 8, height = 6, units = "in",
+             device = "pdf", useDingbats = FALSE)
> cat("\\includegraphics{", pdfFlNam, "}\n\n", sep = "")
```

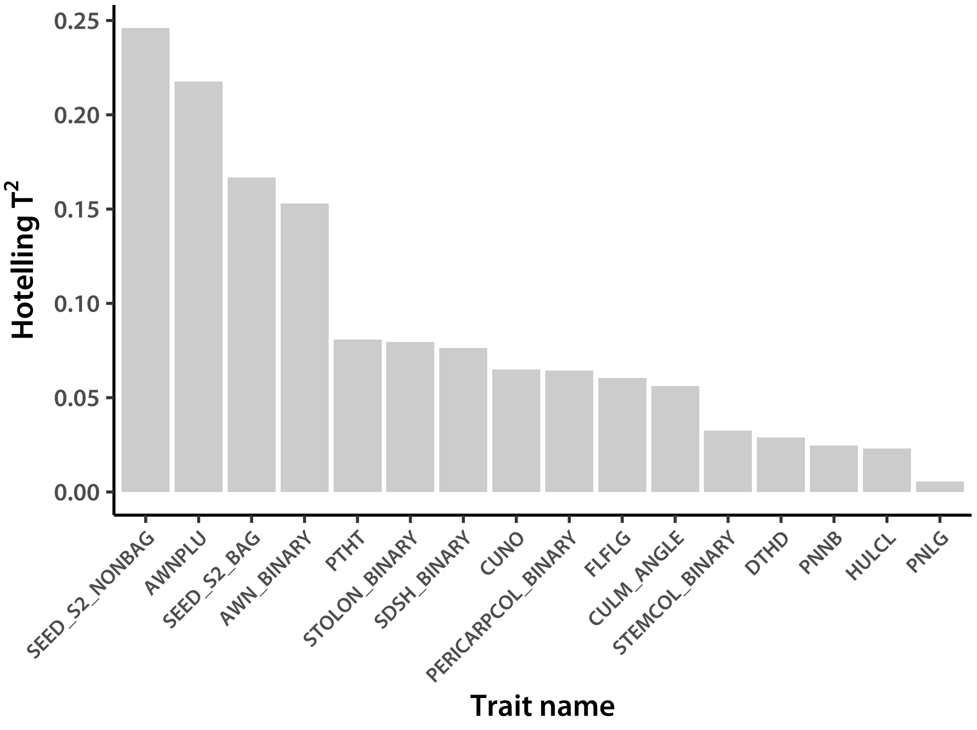

Build trait value boxplots.

```
> colSubs <- c("NSFTV_ID", "newGrp23", trtNamesCU)
> cuPCP   <- cuPCP[cuPheno, on = "NSFTV_ID", nomatch = 0]
> traitLG <- melt(cuPCP, id.vars = c("NSFTV_ID", "newGrp23"),
+             variable.name = "trait",
+             measure.vars = colSubs[-(1:2)], value.name = "value")
> traitLG <- traitLG[, newGrp23 := factor(newGrp23, levels = c("P1", "P2/P3", "P4"))]
> traitLG <- traitLG[, trait := factor(trait, levels = mhlDT$traits)]

> pdfFlNam <- "traitsCUbxpP4.pdf"
> showtext_auto()
> ggplot(data = traitLG, aes(x = newGrp23, y = value, fill = newGrp23)) +
+     geom_boxplot(show.legend = FALSE) +
+     scale_fill_manual(values = popGrpColors) +
+     facet_wrap(~trait, ncol = 4, scales = "free_y") +
+     theme_classic(base_size = 12, base_family = "myriad") +
```

```
+     theme(strip.background = element_rect(fill = "grey95", linetype = "blank"),
+             strip.text = element_text(size = 10, margin = margin(c(1, 0, 1, 0), "pt"))) +
+     xlab("phenotypic group")
> ggsave(pdfFlNam, width = 8, height = 10, units = "in",
+             device = "pdf", useDingbats = FALSE)
> cat("\\includegraphics[width = 0.95\\textwidth]{", pdfFlNam, "}\n\n", sep = "")
```

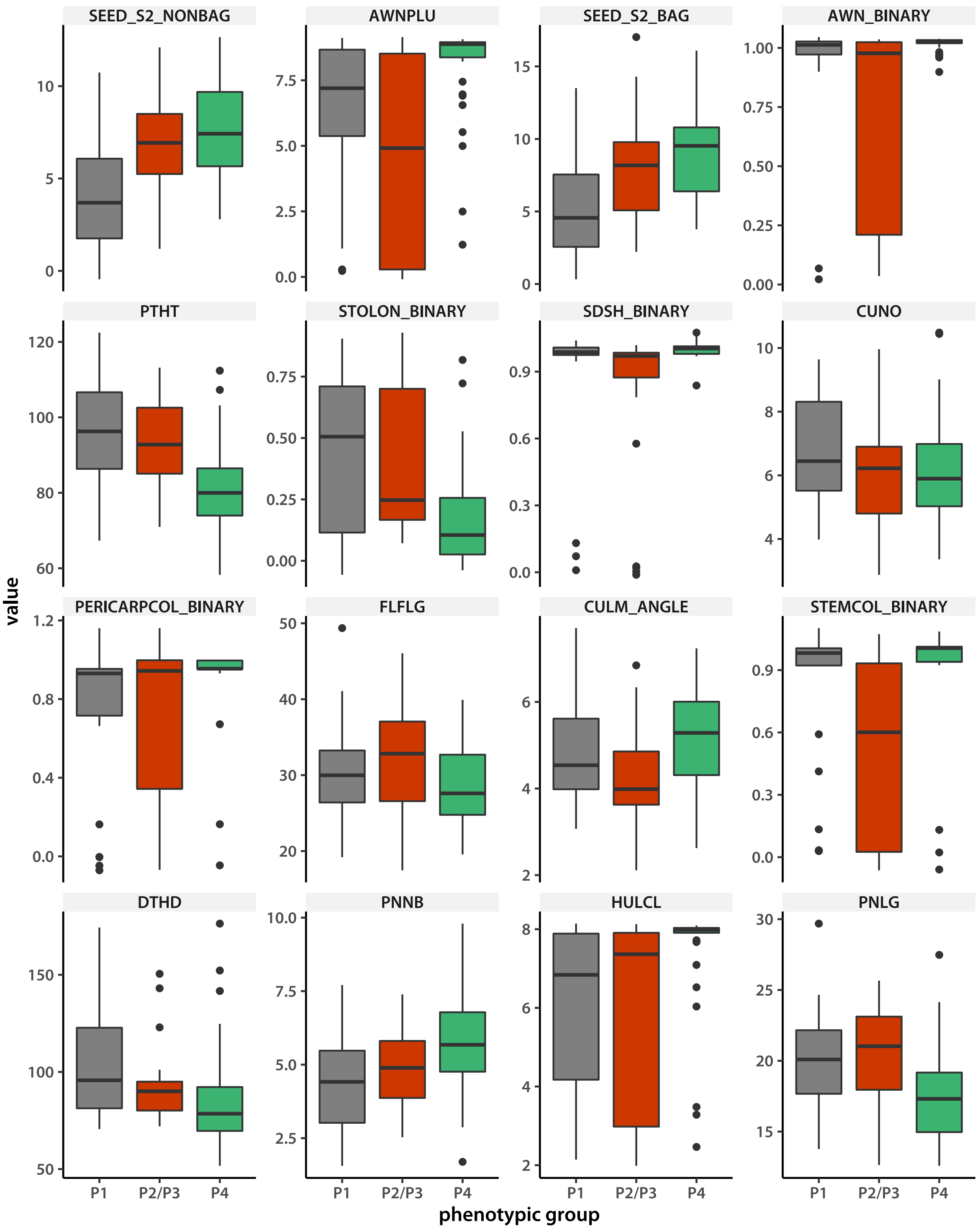

Trait coefficients of variation next.

```
> traitVar <- traitLG[, .(traitCV = sd(value)), by = .(newGrp23, trait)]
> traitVar <- traitVar[, traitCV :=
```

```
+             traitCV/rep(traitLG[, mean(value), by = trait]$V1, each = 3)]
> traitVar <- traitVar[, cvCV := sd(traitCV)/mean(traitCV), by = trait]
> traitVar <- traitVar[, trait := factor(trait, levels = mhlDT$traits)]
```

Plot.

```
> pdfFlNam <- "traitSdCUbpP4.pdf"
> showtext_auto()
> ggplot(data = traitVar,
+         aes(x = newGrp23, y = traitCV, fill = newGrp23)) +
+     geom_col(show.legend = FALSE) +
+     scale_fill_manual(values = popGrpColors) +
+     facet_wrap(~trait, ncol = 4, scales = "free_y") +
+     theme_classic(base_size = 13, base_family = "myriad") +
+     theme(strip.background = element_rect(fill = "grey95", linetype = "blank")) +
+     ylab("coefficient of variation") + xlab("phenotypic group")
> ggsave(pdfFlNam, width = 8, height = 10, units = "in",
+             device = "pdf", useDingbats = FALSE)
> cat("\\includegraphics[width = 0.95\\textwidth]{", pdfFlNam, "}\n\n", sep = "")
```

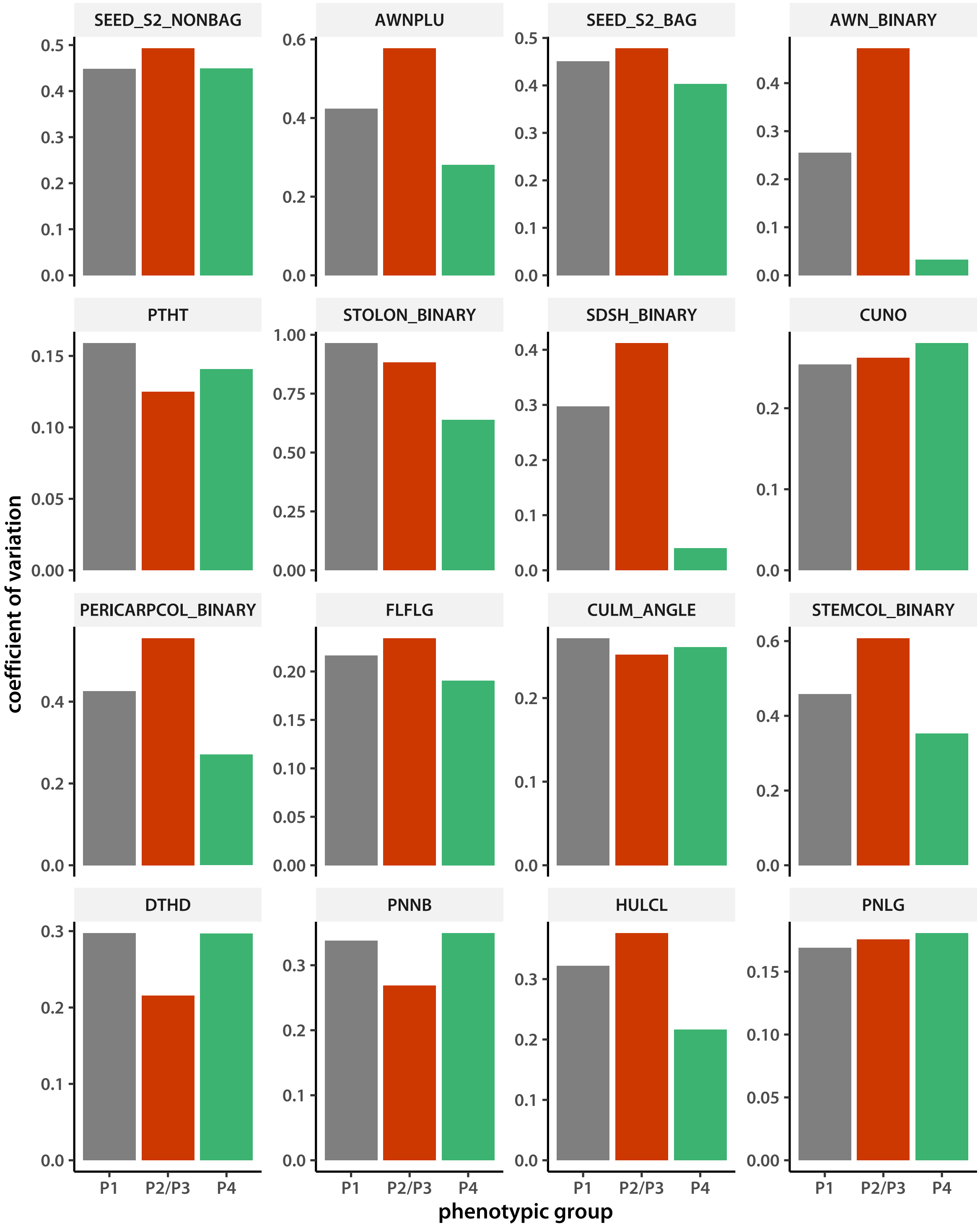
SEED_S2_NONBAG
AWNPLU
SEED_S2_BAG
AWN_BINARY
PTHT
STOLON_BINARY
SDSH_BINARY
CUNO
PERICARPCOL_BINARY
FLFLG
CULM_ANGLE
STEMCOL_BINARY
DTHD
PNNB
HULCL
PNLG
coefficient of variation
P1
P2/P3
P4
phenotypic group

6 Group re-inference from Dale Bumpers and Cornell trait subsets

I next use trait subsets from Cornell and Dale Bumpers data to re-infer groups. I start with the top two by value traits from Dale Bumpers data.

```
> trtNamesVW <- c("HULGRVOL", "HULGRWD")
> Ysc   <- dbPheno[, lapply(.SD, scale), .SDcols = trtNamesVW]
> names(Ysc) <- trtNamesVW
> if (file.exists("ordDBVWp3.Rdata")) {
+     load(file = "ordDBVWp3.Rdata")
+ } else {
+     ordDBVW <- replicate(nReps,
+                     MuGaMix::quickFitModel(Ysc, trtNamesVW, Ngrp, alpha0, nVBreps),
+                     simplify = FALSE)
+     save(ordDBVW, file = "ordDBVWp3.Rdata")
+ }
> pMat <- ordDBVW[[1]]$p
> sum(which(pMat[, 1] > 0.9) %in% which(pMat3db[, 1] > 0.6))

[1] 35

> sum(which(pMat[, 1] > 0.9) %in% which(pMat3db[, 3] > 0.6))

[1] 44

> sum(which(pMat[, 2] > 0.9) %in% which(pMat3db[, 1] > 0.6))

[1] 0

> sum(which(pMat[, 2] > 0.9) %in% which(pMat3db[, 3] > 0.6))

[1] 0

> sum(which(pMat[, 3] > 0.9) %in% which(pMat3db[, 1] > 0.6))

[1] 0

> sum(which(pMat[, 3] > 0.9) %in% which(pMat3db[, 3] > 0.6))

[1] 0

> pMat <- pMat[, 3:1]
```

Assignments to IRRI-derived groups are not that clear. I will use *de novo* inference.

```
> indList <- lapply(2:nReps, function(i){1:Ngrp})
> # P1
> simList <- lapply(1:(nReps - 1), bestColLst,
+           ordDBVW[-1], indList, pMat[, 1], 0.6)
> bestInd <- unlist(lapply(simList, which.max))
> trash   <- sapply(1:(nReps - 1), addCol, 1,
+               Ngrp, ordDBVW)
> # P4
> simList <- lapply(1:(nReps - 1), bestColLst,
+           ordDBVW[-1], indList, pMat[, 3], 0.6)
> bestInd <- unlist(lapply(simList, which.max))
> trash   <- sapply(1:(nReps - 1), addCol, 3,
+               Ngrp, ordDBVW)
> # P2
> bestInd <- rep(1, nReps - 1)
> trash   <- sapply(1:(nReps - 1), addCol, 2,
+                Ngrp, ordDBVW)
> pMat    <- t(apply(pMat, 1, normalizeP))
> pMat    <- pMat[dbPheno$NSFTV_ID %in% dbPCP$NSFTV_ID, ]
> apply(pMat, 1, which.max)
```

```
  [1] 1 1 1 1 1 1 1 1 1 1 1 1 1 1 1 1 1 1 1 1 1 1 1 1 1 1 1 1 1 1 1 1 1 1 1 1 1 1 1 1 1 1
 [43] 1 1 1 1 1 1 1 1 1 1 1 1 1 1 1 1 1 1 1 1 1 1 1 1 1 1 1 1 1 1 1 1 1 1 1 1 1 1 1 1 1 1
 [85] 1 1 1 1 1 1 1 1 1 1 1 1 1 1 1 1 1 1 1 1 1 1 1 1 1 1
```

There is only one group. To see what groups can be inferred from a larger set of traits, I pick three each from Dale Bumpers and Cornell.

```
> bothPheno        <- dbPheno[cuPheno, on = "NSFTV_ID", nomatch = 0]
> names(bothPheno) <- c("NSFTV_ID", paste0(names(dbPheno)[-1], "_DB"),
+                       paste0(trtNamesCU, "_CU"))
> trtNamesDBCU <- paste0(c("PTHT", "HULGRLG", "AWNPLU", "SEED_S2_BAG", "AWNPLU", "PTHT"),
+                 rep(c("_DB", "_CU"), each = 3))
> Ysc          <- bothPheno[, lapply(.SD, scale), .SDcols = trtNamesDBCU]
> names(Ysc)   <- trtNamesDBCU
> if (file.exists("ordDBCUp3.Rdata")) {
+     load(file = "ordDBCUp3.Rdata")
+ } else {
+     ordDBCU <- replicate(nReps,
+                       MuGaMix::quickFitModel(Ysc, trtNamesDBCU, Ngrp, alpha0, nVBreps),
+                       simplify = FALSE)
+     save(ordDBCU, file = "ordDBCUp3.Rdata")
+ }
> pMat <- ordDBCU[[1]]$p
> sum(which(pMat[bothPheno$NSFTV_ID %in% bothPCP$NSFTV_ID, 1] > 0.9)
+     %in% which(pMat3db[, 1] > 0.6))
```

```
[1] 8
```

```
> sum(which(pMat[bothPheno$NSFTV_ID %in% bothPCP$NSFTV_ID, 1] > 0.9)
+     %in% which(pMat3db[, 3] > 0.6))
```

```
[1] 10
```

```
> sum(which(pMat[bothPheno$NSFTV_ID %in% bothPCP$NSFTV_ID, 2] > 0.9)
+     %in% which(pMat3db[, 1] > 0.6))
```

```
[1] 19
```

```
> sum(which(pMat[bothPheno$NSFTV_ID %in% bothPCP$NSFTV_ID, 2] > 0.9)
+     %in% which(pMat3db[, 3] > 0.6))
```

```
[1] 14
```

```
> sum(which(pMat[bothPheno$NSFTV_ID %in% bothPCP$NSFTV_ID, 3] > 0.9)
+     %in% which(pMat3db[, 1] > 0.6))
```

```
[1] 4
```

```
> sum(which(pMat[bothPheno$NSFTV_ID %in% bothPCP$NSFTV_ID, 3] > 0.9)
+     %in% which(pMat3db[, 3] > 0.6))
```

```
[1] 5
```

The group correspondence is not that clear, so I use the *de novo* approach again.

```
> pMat    <- pMat[, c(1, 3, 2)]
> indList <- lapply(2:nReps, function(i){1:Ngrp})
> # P1
> simList <- lapply(1:(nReps - 1), bestColLst,
+           ordDBCU[-1], indList, pMat[, 1], 0.6)
> bestInd <- unlist(lapply(simList, which.max))
> trash   <- sapply(1:(nReps - 1), addCol, 1,
+               Ngrp, ordDBCU)
> # P4
> simList <- lapply(1:(nReps - 1), bestColLst,
+           ordDBCU[-1], indList, pMat[, 3], 0.6)
> bestInd <- unlist(lapply(simList, which.max))
> trash   <- sapply(1:(nReps - 1), addCol, 3,
+               Ngrp, ordDBCU)
> # P2
```

```
> bestInd <- rep(1, nReps - 1)
> trash   <- sapply(1:(nReps - 1), addCol, 2,
+                 Ngrp, ordDBCU)
> pMat    <- t(apply(pMat, 1, normalizeP))
> pMat    <- pMat[bothPheno$NSFTV_ID %in% bothPCP$NSFTV_ID, ]
> bothPCP <- bothPCP[, dbcuSub := paste0("P", apply(pMat, 1, which.max))]
> bothPCP <- bothPCP[, dbcuSub := ifelse(dbcuSub == "P3", "P4", dbcuSub)]
```

Add days to heading.

```
> trtNamesDBCU <- paste0(c("PTHT", "HULGRLG", "AWNPLU", "DTHD",
+                      "SEED_S2_BAG", "AWNPLU", "PTHT", "DTHD"),
+                  rep(c("_DB", "_CU"), each = 4))
> Ysc          <- bothPheno[, lapply(.SD, scale), .SDcols = trtNamesDBCU]
> names(Ysc)   <- trtNamesDBCU
> if (file.exists("ordDBCUp3t8.Rdata")) {
+     load(file = "ordDBCUp3t8.Rdata")
+ } else {
+     ordDBCU8 <- replicate(nReps,
+                       MuGaMix::quickFitModel(Ysc, trtNamesDBCU, Ngrp, alpha0, nVBreps),
+                       simplify = FALSE)
+     save(ordDBCU8, file = "ordDBCUp3t8.Rdata")
+ }
> pMat <- ordDBCU8[[1]]$p
> sum(which(pMat[bothPheno$NSFTV_ID %in% bothPCP$NSFTV_ID, 1] > 0.9)
+     %in% which(pMat3db[, 1] > 0.6))

[1] 14

> sum(which(pMat[bothPheno$NSFTV_ID %in% bothPCP$NSFTV_ID, 1] > 0.9)
+     %in% which(pMat3db[, 3] > 0.6))

[1] 10

> sum(which(pMat[bothPheno$NSFTV_ID %in% bothPCP$NSFTV_ID, 2] > 0.9)
+     %in% which(pMat3db[, 1] > 0.6))

[1] 9

> sum(which(pMat[bothPheno$NSFTV_ID %in% bothPCP$NSFTV_ID, 2] > 0.9)
+     %in% which(pMat3db[, 3] > 0.6))

[1] 8
```

```
> sum(which(pMat[bothPheno$NSFTV_ID %in% bothPCP$NSFTV_ID, 3] > 0.9)
+     %in% which(pMat3db[, 1] > 0.6))

[1] 8
```

```
> sum(which(pMat[bothPheno$NSFTV_ID %in% bothPCP$NSFTV_ID, 3] > 0.9)
+     %in% which(pMat3db[, 3] > 0.6))

[1] 11
```

Again, use *de novo* inference.

```
> pMat    <- pMat[, c(2, 3, 1)]
> indList <- lapply(2:nReps, function(i){1:Ngrp})
> # P1
> simList <- lapply(1:(nReps - 1), bestColLst,
+           ordDBCU8[-1], indList, pMat[, 1], 0.6)
> bestInd <- unlist(lapply(simList, which.max))
> trash   <- sapply(1:(nReps - 1), addCol, 1,
+               Ngrp, ordDBCU8)
> # P4
> simList <- lapply(1:(nReps - 1), bestColLst,
+           ordDBCU8[-1], indList, pMat[, 3], 0.6)
> bestInd <- unlist(lapply(simList, which.max))
> trash   <- sapply(1:(nReps - 1), addCol, 3,
+               Ngrp, ordDBCU8)
> # P2
> bestInd <- rep(1, nReps - 1)
> trash   <- sapply(1:(nReps - 1), addCol, 2,
+                Ngrp, ordDBCU8)
> pMat    <- t(apply(pMat, 1, normalizeP))
> pMat    <- pMat[bothPheno$NSFTV_ID %in% bothPCP$NSFTV_ID, ]
> bothPCP <- bothPCP[, dbcuSub8 := paste0("P", apply(pMat, 1, which.max))]
> bothPCP <- bothPCP[, dbcuSub8 := ifelse(dbcuSub8 == "P3", "P4", dbcuSub8)]
```

Arrange variables for Sankey plots.

```
> pGrpSan       <- as.data.table(make_long(bothPCP, GRIN_spp, dbcuSub,
+                                  dbcuSub8, db3, cu3, newGrp23))
> sankeyLevels0 <- c("O. nivara", "Oryza spp.", "O. rufipogon",
+              c("P4", "P2/P3", "P2", "P1"))
> pGrpSan       <- pGrpSan[, node := factor(node, levels = sankeyLevels0)]
> pGrpSan       <- pGrpSan[, next_node := factor(next_node, levels = sankeyLevels0)]
```

Plot.

```
> pdfFlNam <- "sankeyPGrpDBCUsubset.pdf"
> showtext_auto()
> ggplot(data = pGrpSan, aes(x = x, next_x = next_x, node = node, next_node = next_node,
+                             fill = node, label = node)) +
+     geom_sankey(flow.alpha = 0.6, node.color = "grey30") +
+     geom_sankey_label(size = 5, color = "grey20", fill = "grey90", alpha = 0.8,
+             label = c(expression(italic("O. nivara")),
+                         expression(italic("Oryza spp.")),
+                         expression(italic("O. rufipogon")),
+                         "P4", "P2", "P1", "P4", "P2", "P1",
+                         "P4", "P2", "P1",
+                         "P4", "P2", "P1", "P4", "P2/P3", "P1")) +
+     scale_fill_manual(values = pGrp8Colors) +
+     scale_x_discrete(labels = c("species", "Cornell\nDale Bumpers\n2x3 traits",
+                                 "Cornell\nDale Bumpers\n2x4 traits", "Dale\nBumpers",
+                                 "Cornell", "IRRI\nall traits")) +
+     theme_sankey(base_size = 18, base_family = "myriad") +
+     theme(strip.background = element_rect(fill = "grey95", linetype = "blank"),
+             legend.position = "none", axis.title = element_blank())
> ggsave(pdfFlNam, width = 8, height = 6, units = "in",
+             device = "pdf", useDingbats = FALSE)
> cat("\\includegraphics[width=0.9\\textwidth]{", pdfFlNam, "}\n\n", sep = "")
```

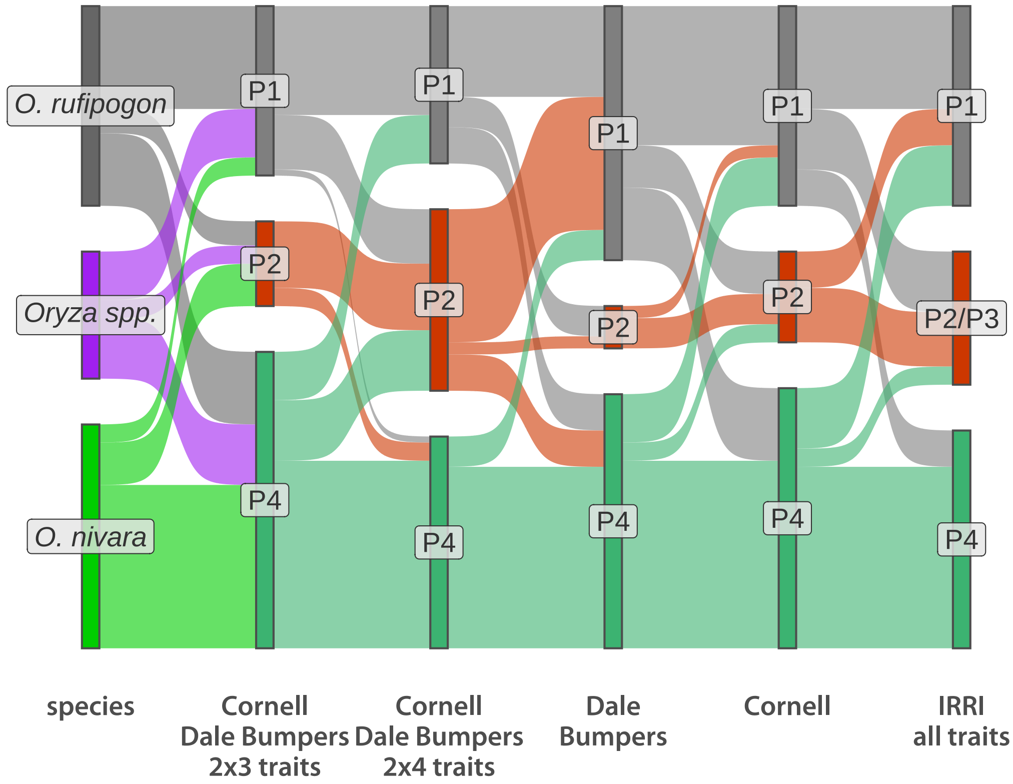

These trait subsets seem mostly good at discrimination between *O. nivara* and the rest of the *ORSC* accessions, and not very consistent in identifying lines with *O. sativa* introgression. Last, I try the trait sets that correspond to the 11 traits identified in IRRI data. I will use the Dale Bumpers and Cornell data separately for this.

6.1 Dale Bumpers intersection with 11 traits

Start with Dale Bumpers data.

```
> trtNamesDB11 <- c("PTHT", "UNFILGRNB", "HULGRWD", "HULGRLG",
+                  "PNLG", "DTHD", "FLFLWD", "FLFLG")
> Ysc   <- dbPheno[, lapply(.SD, scale), .SDcols = trtNamesDB11]
> names(Ysc) <- trtNamesDB11
> if (file.exists("ordDB11p3.Rdata")) {
+     load(file = "ordDB11p3.Rdata")
+ } else {
+     ordDB11 <- replicate(nReps,
+                       MuGaMix::quickFitModel(Ysc, trtNamesDB11, Ngrp, alpha0, nVBreps),
+                       simplify = FALSE)
+     save(ordDB11, file = "ordDB11p3.Rdata")
+ }
```

```
> pMat <- ordDB11[[1]]$p
> sum(which(pMat[, 1] > 0.9) %in% which(pMat3db[, 1] > 0.6))

[1] 7


> sum(which(pMat[, 1] > 0.9) %in% which(pMat3db[, 3] > 0.6))

[1] 5


> sum(which(pMat[, 2] > 0.9) %in% which(pMat3db[, 1] > 0.6))

[1] 11


> sum(which(pMat[, 2] > 0.9) %in% which(pMat3db[, 3] > 0.6))

[1] 12


> sum(which(pMat[, 3] > 0.9) %in% which(pMat3db[, 1] > 0.6))

[1] 11


> sum(which(pMat[, 3] > 0.9) %in% which(pMat3db[, 3] > 0.6))

[1] 21


> pMat <- pMat[, c(2, 1, 3)]
> indList <- lapply(2:nReps, function(i){1:Ngrp})
> # P1
> simList <- lapply(1:(nReps - 1), bestColLst,
+           ordDB11[-1], indList, pMat[, 1], 0.9)
> bestInd <- unlist(lapply(simList, which.max))
> trash   <- sapply(1:(nReps - 1), addCol, 1,
+               Ngrp, ordDB11)
> # P4
> simList <- lapply(1:(nReps - 1), bestColLst,
+           ordDB11[-1], indList, pMat[, 3], 0.9)
> bestInd <- unlist(lapply(simList, which.max))
> trash   <- sapply(1:(nReps - 1), addCol, 3,
+               Ngrp, ordDB11)
> # P2
> bestInd <- rep(1, nReps - 1)
> trash   <- sapply(1:(nReps - 1), addCol, 2,
+                Ngrp, ordDB11)
```

```
> pMat    <- t(apply(pMat, 1, normalizeP))
> pMat    <- pMat[dbPheno$NSFTV_ID %in% dbPCP$NSFTV_ID, ]
> dbPCP   <- dbPCP[, db3s11 := paste0("P", apply(pMat, 1, which.max))]
> dbPCP   <- dbPCP[, db3s11 := ifelse(db3s11 == "P3", "P4", db3s11)]
> pGrpSan <- as.data.table(make_long(dbPCP, GRIN_spp, db3s11, db3, newGrp23))
> sankeyLevelsO <- c("O. nivara", "Oryza spp.", "O. rufipogon",
+             c("P4", "P2/P3", "P2", "P1"))
> pGrpSan       <- pGrpSan[, node := factor(node, levels = sankeyLevelsO)]
> pGrpSan       <- pGrpSan[, next_node := factor(next_node, levels = sankeyLevelsO)]
```

Plot.

```
> pdfFlNam <- "sankeyPGrpDBirri11.pdf"
> showtext_auto()
> ggplot(data = pGrpSan, aes(x = x, next_x = next_x, node = node, next_node = next_node,
+                            fill = node, label = node)) +
+     geom_sankey(flow.alpha = 0.6, node.color = "grey30") +
+     geom_sankey_label(size = 5, color = "grey20", fill = "grey90", alpha = 0.8,
+             label = c(expression(italic("O. nivara")),
+                         expression(italic("Oryza spp.")),
+                         expression(italic("O. rufipogon")),
+                         "P4", "P2", "P1", "P4", "P2", "P1",
+                         "P4", "P2/P3", "P1")) +
+     scale_fill_manual(values = pGrp8Colors) +
+     scale_x_discrete(labels = c("species",
+                                 "Dale Bumpers\nIRRI 8 (11) traits",
+                                 "Dale\nBumpers\nall traits",
+                                 "IRRI\nall traits")) +
+     theme_sankey(base_size = 20, base_family = "myriad") +
+     theme(strip.background = element_rect(fill = "grey95", linetype = "blank"),
+             legend.position = "none", axis.title = element_blank())
> ggsave(pdfFlNam, width = 8, height = 6, units = "in",
+             device = "pdf", useDingbats = FALSE)
> cat("\\includegraphics[width=0.9\\textwidth]{", pdfFlNam, "}\n\n", sep = "")
```

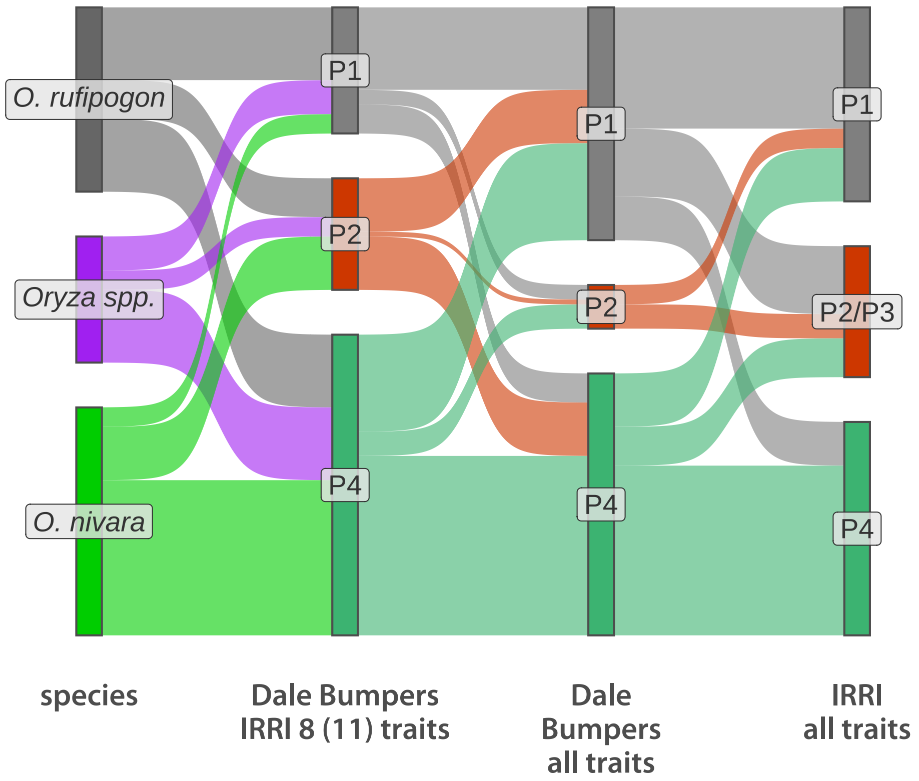

6.2 Cornell traits in the IRRI 11

Repeat with Cornell data.

```
> trtNamesCU11 <- c("PTHT", "SEED_S2_BAG", "PNLG", "DTHD", "FLFLG")
> Ysc    <- cuPheno[, lapply(.SD, scale), .SDcols = trtNamesCU11]
> names(Ysc) <- trtNamesCU11
> if (file.exists("ordCU11p3.Rdata")) {
+     load(file = "ordCU11p3.Rdata")
+ } else {
+     ordCU11 <- replicate(nReps,
+                         MuGaMix::quickFitModel(Ysc, trtNamesCU11, Ngrp, alpha0, nVBreps),
+                         simplify = FALSE)
+     save(ordCU11, file = "ordCU11p3.Rdata")
+ }
> pMat <- ordCU11[[1]]$p
> sum(which(pMat[, 1] > 0.9) %in% which(pMat3cu[, 1] > 0.6))

[1] 15

> sum(which(pMat[, 1] > 0.9) %in% which(pMat3cu[, 3] > 0.6))
```

```
[1] 22
```

```
> sum(which(pMat[, 2] > 0.9) %in% which(pMat3cu[, 1] > 0.6))
```

```
[1] 4
```

```
> sum(which(pMat[, 2] > 0.9) %in% which(pMat3cu[, 3] > 0.6))
```

```
[1] 3
```

```
> sum(which(pMat[, 3] > 0.9) %in% which(pMat3cu[, 1] > 0.6))
```

```
[1] 5
```

```
> sum(which(pMat[, 3] > 0.9) %in% which(pMat3cu[, 3] > 0.6))
```

```
[1] 7
```

```
> pMat    <- pMat[, 3:1]
> indList <- lapply(2:nReps, function(i){1:Ngrp})
> # P1
> simList <- lapply(1:(nReps - 1), bestColLst,
+            ordCU11[-1], indList, pMat[, 1], 0.9)
> bestInd <- unlist(lapply(simList, which.max))
> trash   <- sapply(1:(nReps - 1), addCol, 1,
+                Ngrp, ordCU11)
> # P4
> simList <- lapply(1:(nReps - 1), bestColLst,
+            ordCU11[-1], indList, pMat[, 3], 0.9)
> bestInd <- unlist(lapply(simList, which.max))
> trash   <- sapply(1:(nReps - 1), addCol, 3,
+                Ngrp, ordCU11)
> # P2
> bestInd <- rep(1, nReps - 1)
> trash   <- sapply(1:(nReps - 1), addCol, 2,
+                 Ngrp, ordCU11)
> pMat    <- t(apply(pMat, 1, normalizeP))
> pMat    <- pMat[cuPheno$NSFTV_ID %in% cuPCP$NSFTV_ID, ]
> cuPCP   <- cuPCP[, cu3s11 := paste0("P", apply(pMat, 1, which.max))]
> cuPCP   <- cuPCP[, cu3s11 := ifelse(cu3s11 == "P3", "P4", cu3s11)]
> pGrpSan <- as.data.table(make_long(cuPCP, GRIN_spp, cu3s11, cu3, newGrp23))
> sankeyLevelsO <- c("O. nivara", "Oryza spp.", "O. rufipogon",
+              c("P4", "P2/P3", "P2", "P1"))
> pGrpSan <- pGrpSan[, node := factor(node, levels = sankeyLevelsO)]
> pGrpSan <- pGrpSan[, next_node := factor(next_node, levels = sankeyLevelsO)]
```

Plot.

```
> pdfFlNam <- "sankeyPGrpCUirri11.pdf"
> showtext_auto()
> ggplot(data = pGrpSan, aes(x = x, next_x = next_x, node = node, next_node = next_node,
+                             fill = node, label = node)) +
+     geom_sankey(flow.alpha = 0.6, node.color = "grey30") +
+     geom_sankey_label(size = 5, color = "grey20", fill = "grey90", alpha = 0.8,
+             label = c(expression(italic("O. nivara")),
+                         expression(italic("Oryza spp.")),
+                         expression(italic("O. rufipogon")),
+                         "P4", "P2", "P1", "P4", "P2", "P1",
+                         "P4", "P2/P3", "P1")) +
+     scale_fill_manual(values = pGrp8Colors) +
+     scale_x_discrete(labels = c("species",
+                                 "Cornell\nIRRI 5 (11) traits",
+                                 "Cornell\nall traits",
+                                 "IRRI\nall traits")) +
+     theme_sankey(base_size = 20, base_family = "myriad") +
+     theme(strip.background = element_rect(fill = "grey95", linetype = "blank"),
+             legend.position = "none", axis.title = element_blank())
> ggsave(pdfFlNam, width = 8, height = 6, units = "in",
+             device = "pdf", useDingbats = FALSE)
> cat("\\includegraphics[width=0.9\\textwidth]{", pdfFlNam, "}\n\n", sep = "")
```

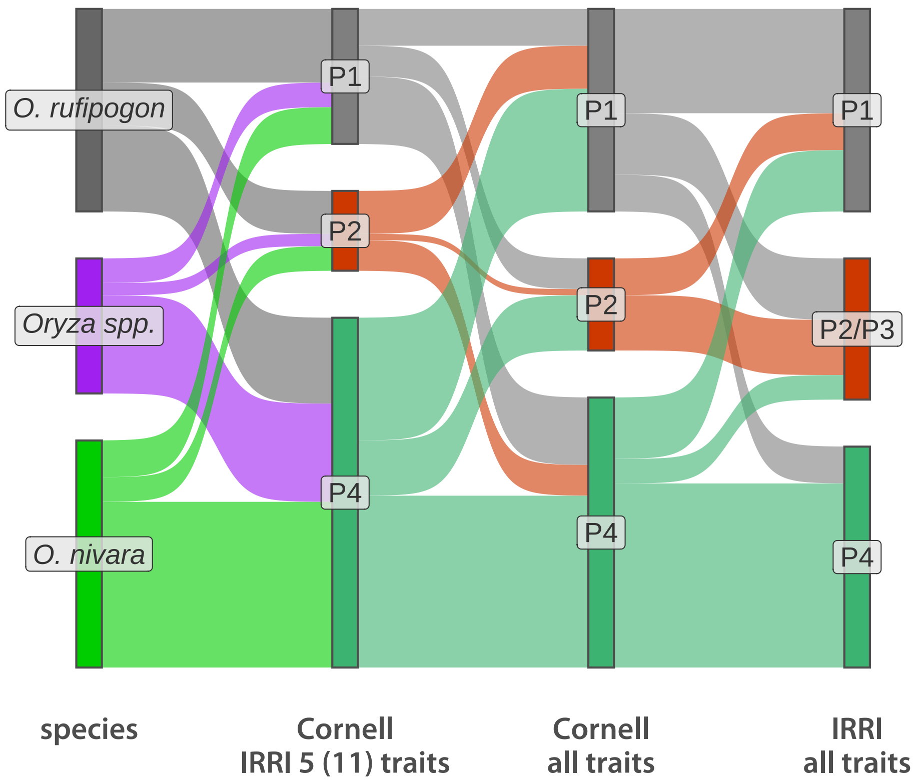

7 Dale Bumpers and Cornell within-group correlations

I next estimate among-trait correlation in the Cornell and Dale Bumpers data sets within IRRI data derived groups.

7.1 Dale Bumpers

```
> d       <- length(trtNamesDB)
> Ngrp    <- 3
> corList <- list(P1 = NULL, "P2/P3" = NULL, P4 = NULL)
> grpCor  <- cor(matrix(unlist(dbPCP[newGrp23 == "P1", ..trtNamesDB]),
+                         ncol = d))
> colnames(grpCor) <- trtNamesDB
> rownames(grpCor) <- trtNamesDB
> trtNamesDB       <- trtNamesDB[hclust(as.dist(1.0 - grpCor), method = "centroid")$order]
> grpCor           <- grpCor[trtNamesDB, trtNamesDB]
> corList$P1       <- grpCor
> grpCor[row(grpCor) >= col(grpCor)] <- NA
> corDT <- data.table(correlation = array(grpCor),
+                     x     = paste(rep(trtNamesDB, each = d), "P1", sep = "."),
```

```
+                      y     = rep(trtNamesDB, times = d),
+                      group = rep("P1", times = d^2))
> corDT <- corDT[!is.na(correlation), ]
> for (grp in c("P2/P3", "P4")){
+     grpCor <- cor(matrix(unlist(dbPCP[newGrp23 == grp, ..trtNamesDB]),
+                          ncol = d))
+     colnames(grpCor) <- trtNamesDB
+     rownames(grpCor) <- trtNamesDB
+     corList[[grp]]   <- grpCor
+     if (grp == "P2/P3"){
+         grpCor[row(grpCor) > col(grpCor)] <- NA
+         diag(grpCor) <- 0.0
+     } else {
+         grpCor[row(grpCor) >= col(grpCor)] <- NA
+     }
+     corDT <- rbind(corDT, data.table(correlation = array(grpCor),
+                          x     = paste(rep(trtNamesDB, each = d), grp, sep = "."),
+                          y     = rep(trtNamesDB, times = d),
+                          group = rep(grp, times = d^2)))
+     corDT <- corDT[!is.na(correlation), ]
+     corDT[paste(y, "P1", sep = ".") == x, group := NA]
+ }
> corDT <- corDT[, x := factor(x, levels = paste(rep(trtNamesDB, each = Ngrp),
+                                  rep(c("P1", "P2/P3", "P4"), times = d), sep = "."))]
> corDT <- corDT[, y := factor(y, levels = trtNamesDB)]
> oneSample <- function(){
+     tmpDT <- dbPCP[, .(corPer = lowTrCor(.SD)),
+                  by = sample(newGrp23), .SDcols = trtNamesDB]
+     tmpDT <- tmpDT[, traitPair := ..traitPair]
+     tmpDT <- tmpDT[, .(corSpanPer = diff(range(corPer))), by = traitPair]
+     return(tmpDT[, corSpanPer])
+ }
> if (file.exists("corPvalDB.tsv")) {
+     pValDT <- fread("corPvalDB.tsv")
+ } else {
+     traitPair  <- corDT[sub("\\.P.+", "", x) != y, ]
+     traitPair  <- traitPair[, traitPair := paste(sub("\\.P.+", "", x), y, sep = ".")]
+     traitPair  <- traitPair[, traitPair]
+     ltCorDT    <- dbPCP[, .(corTrue = lowTrCor(.SD)),
+                      by = newGrp23, .SDcols = trtNamesDB]
+     ltCorDT    <- ltCorDT[, traitPair := ..traitPair]
+     ltCorDT    <- ltCorDT[, .(corSpan = diff(range(corTrue))), by = traitPair]
+     spanPerMat <- replicate(9999, oneSample())
+     spanPerMat <- cbind(ltCorDT[, corSpan], spanPerMat)
+     traitPmat  <- matrix(unlist(strsplit(ltCorDT[, traitPair], "\\.")),
```

```
+                          ncol = 2, byrow = TRUE)
+     pValDT     <- data.table(x = factor(traitPmat[, 1], levels = trtNamesDB),
+                          y = factor(traitPmat[, 2], levels = trtNamesDB),
+                          p = apply(spanPerMat, 1, getPval))
+     pValDT <- pValDT[, trtPair := paste(x, y, sep = ".")]
+     corDT  <- corDT[, trtPair := paste(sub("\\.P.+", "", x), y, sep = ".")]
+     pValDT <- pValDT[corDT, on = "trtPair"]
+     pValDT <- pValDT[is.na(p), p := 1.0]
+     fwrite(pValDT, file = "corPvalDB.tsv", quote = FALSE, sep = "\t")
+ }
> pValDT <- pValDT[, i.x := factor(i.x, levels = paste(rep(trtNamesDB, each = Ngrp),
+                                  rep(c("P1", "P2/P3", "P4"), times = d), sep = "."))]
> pValDT <- pValDT[, i.y := factor(i.y, levels = trtNamesDB)]
> corPvalsP1 <- setorder(pValDT, p)
> corPvalsP1[p <= 0.01, .(x, y, p, correlation, group)]
```

```
               x          y      p correlation group
1:         PBRNB       PTHT 0.0046  0.44423547    P1
2:         PBRNB       PTHT 0.0046  0.04539092 P2/P3
3:         PBRNB       PTHT 0.0046  0.69128637    P4
4: STOLON_BINARY CULM_ANGLE 0.0061  0.67631043    P1
5: STOLON_BINARY CULM_ANGLE 0.0061  0.05138335 P2/P3
6: STOLON_BINARY CULM_ANGLE 0.0061  0.45200242    P4
7:         FLFLG    HULGRWD 0.0086 -0.13148740    P1
8:         FLFLG    HULGRWD 0.0086  0.63394178 P2/P3
9:         FLFLG    HULGRWD 0.0086  0.23228566    P4
```

```
> fwrite(corPvalsP1[p <= 0.01, .(x, y, p, correlation, group)],
+         file = "corSwitchDB.tsv", sep = "\t", quote = FALSE)
```

Plot.

```
> pdfFlNam <- "traitCorDBp4.pdf"
> showtext_auto()
> ggplot(data = corDT, aes(x = x, y = y, fill = correlation)) +
+     geom_tile() +
+     scale_fill_gradient2(low = "royalblue4", high = "mediumvioletred",
+                 mid = "white", midpoint = 0, limit = c(-1, 1)) +
+     geom_point(data = pValDT[grep("P2/P3", i.x), ],
+                 aes(x = as.integer(i.x) - 2.0, y = i.y, color = p), size = 2) +
+     scale_color_gradient2(low = "#27015d", high = "#fbf9fd",
+                          mid = "#6801f9", midpoint = 0.05, limit = c(0, 1)) +
+     scale_x_discrete(expand = c(0.1, 0.0)) +
+     scale_y_discrete(expand = c(0.12, 0.0)) +
+     geom_segment(data = corDT[grep("P4", x), ],
+                 aes(x = as.integer(x) - 4.5, y = 0.5,
```

```
+                     xend = as.integer(x) - 4.5, yend = as.integer(y) + 0.5),
+                 color = "grey30", size = 0.75) +
+     geom_segment(data = corDT[grep("P1", x), ],
+                 aes(x = as.integer(x) - 2.5, y = as.integer(y) + 0.5,
+                     xend = max(as.integer(x)) + 0.5, yend = as.integer(y) + 0.5),
+                 color = "grey30", size = 0.75) +
+     geom_segment(x = 1.5, y = 0.5, xend = d*Ngrp - 1.5, yend = 0.5,
+                 color = "grey30", size = 0.75) +
+     geom_segment(x = d*Ngrp - 1.5, y = 0.5, xend = d*Ngrp - 1.5,
+                     yend = d - 0.5, color = "grey30", size = 0.75) +
+     theme_minimal(base_size = 18, base_family = "myriad") +
+     theme(axis.title = element_blank(),
+             axis.ticks = element_blank(),
+             axis.text = element_blank(),
+             panel.grid.major = element_blank(),
+             legend.position = c(0.32, 0.67),
+             legend.direction = "horizontal",
+             legend.justification = c(1, 0)) +
+     geom_text(data = corDT[x == paste(y, "P2/P3", sep = "."), ],
+             aes(x = x, y = y, label = y),
+             hjust = 1.0, angle = -45, fontface = "bold", size = 5) +
+     guides(fill = guide_colorbar(barwidth = 9, barheight = 1,
+                     title.position = "top", title.hjust = 0.5),
+             color = guide_colorbar(barwidth = 9, barheight = 1,
+                     title.position = "top", title.hjust = 0.5))
> ggsave(pdfFlNam, width = 12, height = 10, units = "in",
+             device = "pdf", useDingbats = FALSE)
> cat("\\includegraphics[width = 0.95\\textwidth]{", pdfFlNam, "}\n\n", sep = "")
```

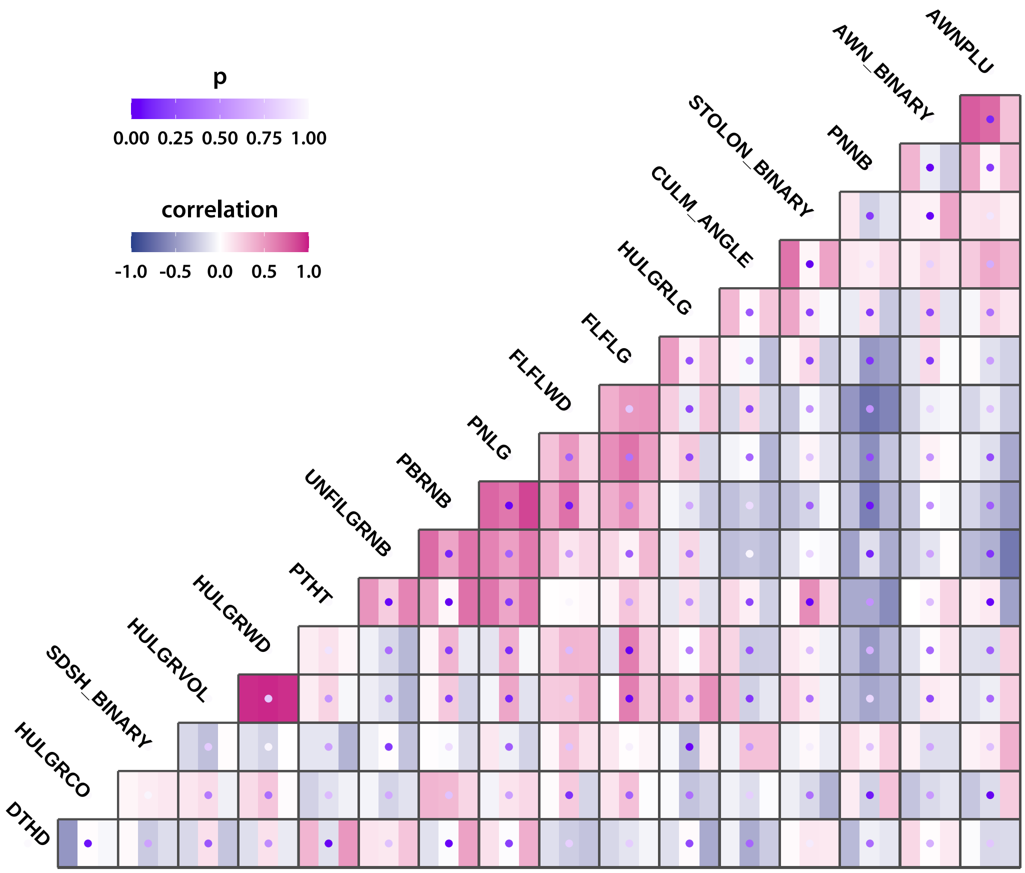

Now use the top correlation-switching traits to re-run the mixture model.

```
> trtNamesDBcs <- unique(unlist(corPvalsP1[p <= 0.01, .(x, y)]))
> Ysc          <- dbPheno[, lapply(.SD, scale), .SDcols = trtNamesDBcs]
> names(Ysc) <- trtNamesDBcs
> if (file.exists("ordDBCORp3.Rdata")) {
+     load(file = "ordDBCORp3.Rdata")
+ } else {
+     ordDBcs <- replicate(nReps,
+                     MuGaMix::quickFitModel(Ysc, trtNamesDBcs, Ngrp, alpha0, nVBreps),
+                     simplify = FALSE)
+     save(ordDBcs, file = "ordDBCORp3.Rdata")
+ }
> pMat <- ordDBcs[[1]]$p
> sum(which(pMat[, 1] > 0.9) %in% which(pMat3db[, 1] > 0.6))
```

```
[1] 2
```

```
> sum(which(pMat[, 1] > 0.9) %in% which(pMat3db[, 3] > 0.6))
[1] 1

> sum(which(pMat[, 2] > 0.9) %in% which(pMat3db[, 1] > 0.6))
[1] 6

> sum(which(pMat[, 2] > 0.9) %in% which(pMat3db[, 3] > 0.6))
[1] 11

> sum(which(pMat[, 3] > 0.9) %in% which(pMat3db[, 1] > 0.6))
[1] 26

> sum(which(pMat[, 3] > 0.9) %in% which(pMat3db[, 3] > 0.6))
[1] 30
```

No straight-forward correspondence to IRRI groups, so running *de novo*.

```
> indList <- lapply(2:nReps, function(i){1:Ngrp})
> # P1
> simList <- lapply(1:(nReps - 1), bestColLst,
+            ordDBcs[-1], indList, pMat[, 1], 0.9)
> bestInd <- unlist(lapply(simList, which.max))
> trash   <- sapply(1:(nReps - 1), addCol, 1,
+                Ngrp, ordDBcs)
> # P4
> simList <- lapply(1:(nReps - 1), bestColLst,
+            ordDBcs[-1], indList, pMat[, 3], 0.9)
> bestInd <- unlist(lapply(simList, which.max))
> trash   <- sapply(1:(nReps - 1), addCol, 3,
+                Ngrp, ordDBcs)
> # P2
> bestInd <- rep(1, nReps - 1)
> trash   <- sapply(1:(nReps - 1), addCol, 2,
+                 Ngrp, ordDBcs)
> pMat    <- t(apply(pMat, 1, normalizeP))
> pMat    <- pMat[dbPheno$NSFTV_ID %in% dbPCP$NSFTV_ID, ]
> dbPCP   <- dbPCP[, db3cor := paste0("P", apply(pMat, 1, which.max))]
> dbPCP   <- dbPCP[, db3cor := ifelse(db3cor == "P3", "P4", db3cor)]
> pGrpSan <- as.data.table(make_long(dbPCP, GRIN_spp, db3cor, db3, newGrp23))
> sankeyLevelsO <- c("O. nivara", "Oryza spp.", "O. rufipogon",
+              c("P4", "P2/P3", "P2", "P1"))
> pGrpSan <- pGrpSan[, node := factor(node, levels = sankeyLevelsO)]
> pGrpSan <- pGrpSan[, next_node := factor(next_node, levels = sankeyLevelsO)]
```

Plot.

```
> pdfFlNam <- "sankeyPGrpDBcor.pdf"
> showtext_auto()
> ggplot(data = pGrpSan, aes(x = x, next_x = next_x, node = node, next_node = next_node,
+                             fill = node, label = node)) +
+     geom_sankey(flow.alpha = 0.6, node.color = "grey30") +
+     geom_sankey_label(size = 5, color = "grey20", fill = "grey90", alpha = 0.8,
+             label = c(expression(italic("O. nivara")),
+                         expression(italic("Oryza spp.")),
+                         expression(italic("O. rufipogon")),
+                         "P4", "P2", "P1", "P4", "P2", "P1",
+                         "P4", "P2/P3", "P1")) +
+     scale_fill_manual(values = pGrp8Colors) +
+     scale_x_discrete(labels = c("species",
+                                 "Dale Bumpers\ncorrelation (6)",
+                                 "Dale\nBumpers\nall traits",
+                                 "IRRI\nall traits")) +
+     theme_sankey(base_size = 20, base_family = "myriad") +
+     theme(strip.background = element_rect(fill = "grey95", linetype = "blank"),
+             legend.position = "none", axis.title = element_blank())
> ggsave(pdfFlNam, width = 8, height = 6, units = "in",
+             device = "pdf", useDingbats = FALSE)
> cat("\\includegraphics[width=0.9\\textwidth]{", pdfFlNam, "}\n\n", sep = "")
```

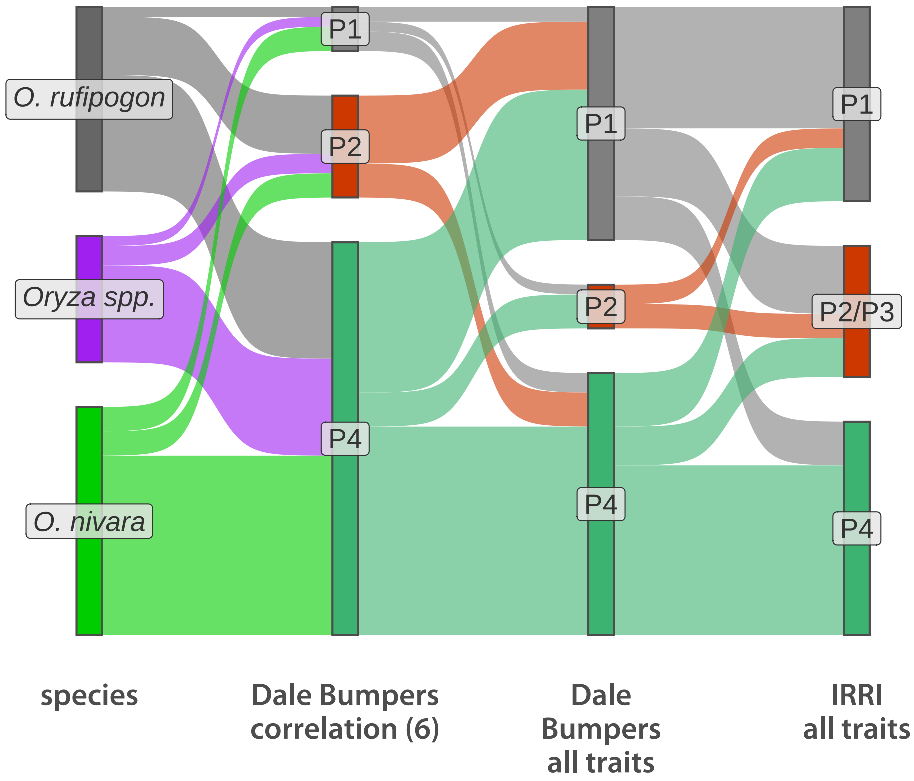

7.2 Cornell

```
> cuPCP   <- cuPCP[cuPheno, on = "NSFTV_ID", nomatch = 0]
> d       <- length(trtNamesCU)
> Ngrp    <- 3
> corList <- list(P1 = NULL, "P2/P3" = NULL, P4 = NULL)
> grpCor  <- cor(matrix(unlist(cuPCP[newGrp23 == "P1", ..trtNamesCU]),
+                            ncol = d))
> colnames(grpCor) <- trtNamesCU
> rownames(grpCor) <- trtNamesCU
> trtNamesCU       <- trtNamesCU[hclust(as.dist(1.0 - grpCor), method = "centroid")$order]
> grpCor           <- grpCor[trtNamesCU, trtNamesCU]
> corList$P1       <- grpCor
> grpCor[row(grpCor) >= col(grpCor)] <- NA
> corDT <- data.table(correlation = array(grpCor),
+                     x     = paste(rep(trtNamesCU, each = d), "P1", sep = "."),
+                     y     = rep(trtNamesCU, times = d),
+                     group = rep("P1", times = d^2))
> corDT <- corDT[!is.na(correlation), ]
> for (grp in c("P2/P3", "P4")){
+     grpCor <- cor(matrix(unlist(cuPCP[newGrp23 == grp, ..trtNamesCU]),
```

```
+                          ncol = d))
+     colnames(grpCor) <- trtNamesCU
+     rownames(grpCor) <- trtNamesCU
+     corList[[grp]]   <- grpCor
+     if (grp == "P2/P3"){
+         grpCor[row(grpCor) > col(grpCor)] <- NA
+         diag(grpCor) <- 0.0
+     } else {
+         grpCor[row(grpCor) >= col(grpCor)] <- NA
+     }
+     corDT <- rbind(corDT, data.table(correlation = array(grpCor),
+                          x     = paste(rep(trtNamesCU, each = d), grp, sep = "."),
+                          y     = rep(trtNamesCU, times = d),
+                          group = rep(grp, times = d^2)))
+     corDT <- corDT[!is.na(correlation), ]
+     corDT[paste(y, "P1", sep = ".") == x, group := NA]
+ }
> corDT <- corDT[, x := factor(x, levels = paste(rep(trtNamesCU, each = Ngrp),
+                                  rep(c("P1", "P2/P3", "P4"), times = d), sep = "."))]
> corDT <- corDT[, y := factor(y, levels = trtNamesCU)]
> oneSample <- function(){
+     tmpDT <- cuPCP[, .(corPer = lowTrCor(.SD)),
+                 by = sample(newGrp23), .SDcols = trtNamesCU]
+     tmpDT <- tmpDT[, traitPair := ..traitPair]
+     tmpDT <- tmpDT[, .(corSpanPer = diff(range(corPer))), by = traitPair]
+     return(tmpDT[, corSpanPer])
+ }
> if (file.exists("corPvalCU.tsv")) {
+     pValDT <- fread("corPvalCU.tsv")
+ } else {
+     traitPair  <- corDT[sub("\\.P.+", "", x) != y, ]
+     traitPair  <- traitPair[, traitPair := paste(sub("\\.P.+", "", x), y, sep = ".")]
+     traitPair  <- traitPair[, traitPair]
+     ltCorDT    <- cuPCP[, .(corTrue = lowTrCor(.SD)),
+                     by = newGrp23, .SDcols = trtNamesCU]
+     ltCorDT    <- ltCorDT[, traitPair := ..traitPair]
+     ltCorDT    <- ltCorDT[, .(corSpan = diff(range(corTrue))), by = traitPair]
+     spanPerMat <- replicate(9999, oneSample())
+     spanPerMat <- cbind(ltCorDT[, corSpan], spanPerMat)
+     traitPmat  <- matrix(unlist(strsplit(ltCorDT[, traitPair], "\\.")),
+                          ncol = 2, byrow = TRUE)
+     pValDT     <- data.table(x = factor(traitPmat[, 1], levels = trtNamesCU),
+                          y = factor(traitPmat[, 2], levels = trtNamesCU),
+                          p = apply(spanPerMat, 1, getPval))
+     pValDT <- pValDT[, trtPair := paste(x, y, sep = ".")]
```

```
+     corDT  <- corDT[, trtPair := paste(sub("\\.P.+", "", x), y, sep = ".")]
+     pValDT <- pValDT[corDT, on = "trtPair"]
+     pValDT <- pValDT[is.na(p), p := 1.0]
+     fwrite(pValDT, file = "corPvalCU.tsv", quote = FALSE, sep = "\t")
+ }
> pValDT <- pValDT[, i.x := factor(i.x, levels = paste(rep(trtNamesCU, each = Ngrp),
+                                 rep(c("P1", "P2/P3", "P4"), times = d), sep = "."))]
> pValDT <- pValDT[, i.y := factor(i.y, levels = trtNamesCU)]
> corPvalsP1 <- setorder(pValDT, p)
> corPvalsP1[p <= 0.01, .(x, y, p, correlation, group)]
```

```
              x              y      p correlation group
 1: SEED_S2_BAG           PNNB 0.0035  0.59745996    P1
 2: SEED_S2_BAG           PNNB 0.0035 -0.05346446 P2/P3
 3: SEED_S2_BAG           PNNB 0.0035  0.55210038    P4
 4:       HULCL          FLFLG 0.0048  0.36929946    P1
 5:       HULCL          FLFLG 0.0048  0.21143570 P2/P3
 6:       HULCL          FLFLG 0.0048 -0.40768852    P4
 7: SEED_S2_BAG           PNLG 0.0057 -0.15317897    P1
 8: SEED_S2_BAG           PNLG 0.0057  0.49440716 P2/P3
 9: SEED_S2_BAG           PNLG 0.0057 -0.33377214    P4
10:  CULM_ANGLE STEMCOL_BINARY 0.0097  0.06138276    P1
11:  CULM_ANGLE STEMCOL_BINARY 0.0097  0.63436569 P2/P3
12:  CULM_ANGLE STEMCOL_BINARY 0.0097  0.32532670    P4
```

```
> fwrite(corPvalsP1[p <= 0.01, .(x, y, p, correlation, group)],
+         file = "corSwitchCU.tsv", sep = "\t", quote = FALSE)
```

Plot.

```
> pdfFlNam <- "traitCorCUp4.pdf"
> showtext_auto()
> ggplot(data = corDT, aes(x = x, y = y, fill = correlation)) +
+     geom_tile() +
+     scale_fill_gradient2(low = "royalblue4", high = "mediumvioletred",
+                 mid = "white", midpoint = 0, limit = c(-1, 1)) +
+     geom_point(data = pValDT[grep("P2/P3", i.x), ],
+                 aes(x = as.integer(i.x) - 2.0, y = i.y, color = p), size = 2) +
+     scale_color_gradient2(low = "#27015d", high = "#fbf9fd",
+                         mid = "#6801f9", midpoint = 0.05, limit = c(0, 1)) +
+     scale_x_discrete(expand = c(0.13, 0.0)) +
+     scale_y_discrete(expand = c(0.12, 0.0)) +
+     geom_segment(data = corDT[grep("P4", x), ],
+                 aes(x = as.integer(x) - 4.5, y = 0.5,
+                     xend = as.integer(x) - 4.5, yend = as.integer(y) + 0.5),
+                 color = "grey30", size = 0.75) +
+     geom_segment(data = corDT[grep("P1", x), ],
```

```
+                 aes(x = as.integer(x) - 2.5, y = as.integer(y) + 0.5,
+                     xend = max(as.integer(x)) + 0.5, yend = as.integer(y) + 0.5),
+                 color = "grey30", size = 0.75) +
+     geom_segment(x = 1.5, y = 0.5, xend = d*Ngrp - 1.5, yend = 0.5,
+                 color = "grey30", size = 0.75) +
+     geom_segment(x = d*Ngrp - 1.5, y = 0.5, xend = d*Ngrp - 1.5,
+                     yend = d - 0.5, color = "grey30", size = 0.75) +
+     theme_minimal(base_size = 18, base_family = "myriad") +
+     theme(axis.title = element_blank(),
+             axis.ticks = element_blank(),
+             axis.text = element_blank(),
+             panel.grid.major = element_blank(),
+             legend.position = c(0.32, 0.67),
+             legend.direction = "horizontal",
+             legend.justification = c(1, 0)) +
+     geom_text(data = corDT[x == paste(y, "P2/P3", sep = "."), ],
+             aes(x = x, y = y, label = y),
+             hjust = 1.0, angle = -45, fontface = "bold", size = 5) +
+     guides(fill = guide_colorbar(barwidth = 9, barheight = 1,
+                     title.position = "top", title.hjust = 0.5),
+             color = guide_colorbar(barwidth = 9, barheight = 1,
+                     title.position = "top", title.hjust = 0.5))
> ggsave(pdfFlNam, width = 12, height = 10, units = "in",
+             device = "pdf", useDingbats = FALSE)
> cat("\\includegraphics[width = 0.95\\textwidth]{", pdfFlNam, "}\n\n", sep = "")
```

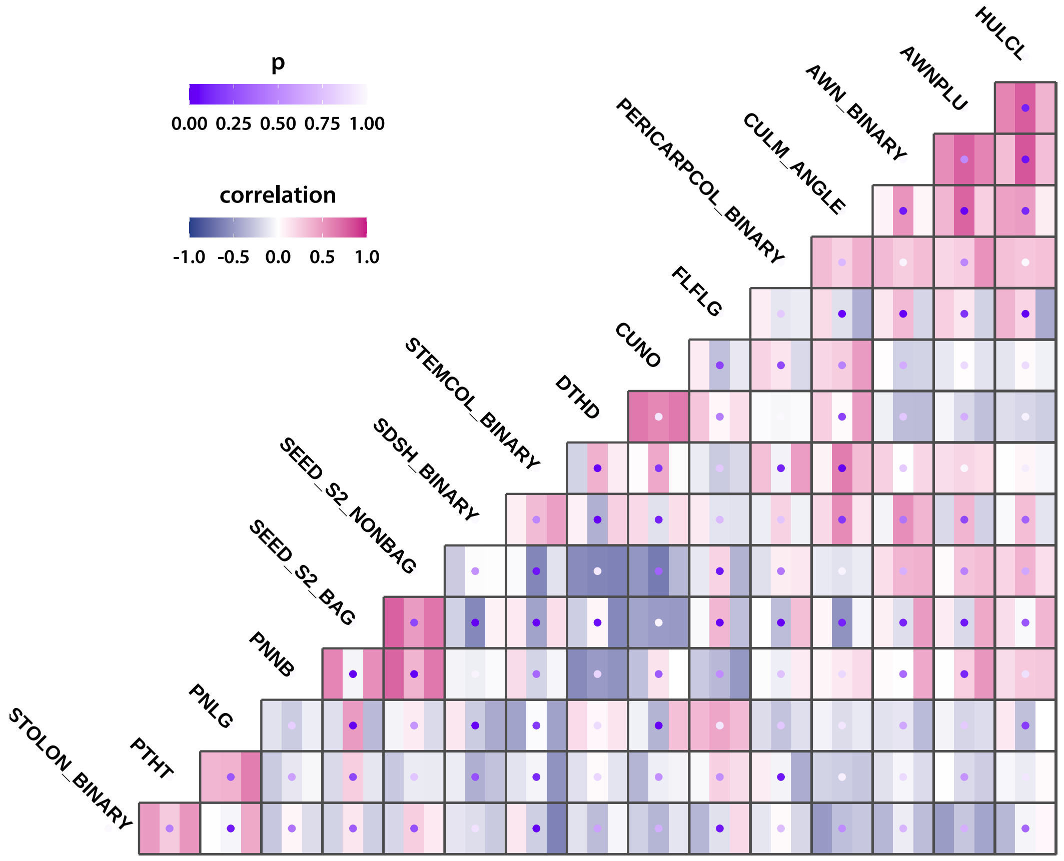

Again, use the top correlation-switching traits to re-run the mixture model.

```
> trtNamesCUcs <- unique(unlist(corPvalsP1[p <= 0.01, .(x, y)]))
> Ysc          <- cuPheno[, lapply(.SD, scale), .SDcols = trtNamesCUcs]
> names(Ysc)   <- trtNamesCUcs
> if (file.exists("ordCUCORp3.Rdata")) {
+     load(file = "ordCUCORp3.Rdata")
+ } else {
+     ordCUcs <- replicate(nReps,
+                     MuGaMix::quickFitModel(Ysc, trtNamesCUcs, Ngrp, alpha0, nVBreps),
+                     simplify = FALSE)
+     save(ordCUcs, file = "ordCUCORp3.Rdata")
+ }
> pMat <- ordCUcs[[1]]$p
> sum(which(pMat[, 1] > 0.9) %in% which(pMat3cu[, 1] > 0.6))

[1] 9
```

```
> sum(which(pMat[, 1] > 0.9) %in% which(pMat3cu[, 3] > 0.6))
[1] 10

> sum(which(pMat[, 2] > 0.9) %in% which(pMat3cu[, 1] > 0.6))
[1] 7

> sum(which(pMat[, 2] > 0.9) %in% which(pMat3cu[, 3] > 0.6))
[1] 4

> sum(which(pMat[, 3] > 0.9) %in% which(pMat3cu[, 1] > 0.6))
[1] 12

> sum(which(pMat[, 3] > 0.9) %in% which(pMat3cu[, 3] > 0.6))
[1] 22
```

No straight-forward correspondence to IRRI groups again, so running *de novo*.

```
> indList <- lapply(2:nReps, function(i){1:Ngrp})
> # P1
> simList <- lapply(1:(nReps - 1), bestColLst,
+           ordCUcs[-1], indList, pMat[, 1], 0.9)
> bestInd <- unlist(lapply(simList, which.max))
> trash   <- sapply(1:(nReps - 1), addCol, 1,
+               Ngrp, ordCUcs)
> # P4
> simList <- lapply(1:(nReps - 1), bestColLst,
+           ordCUcs[-1], indList, pMat[, 3], 0.9)
> bestInd <- unlist(lapply(simList, which.max))
> trash   <- sapply(1:(nReps - 1), addCol, 3,
+               Ngrp, ordCUcs)
> # P2
> bestInd <- rep(1, nReps - 1)
> trash   <- sapply(1:(nReps - 1), addCol, 2,
+                Ngrp, ordCUcs)
> pMat    <- t(apply(pMat, 1, normalizeP))
> pMat    <- pMat[cuPheno$NSFTV_ID %in% cuPCP$NSFTV_ID, ]
> cuPCP   <- cuPCP[, cu3cor := paste0("P", apply(pMat, 1, which.max))]
> cuPCP   <- cuPCP[, cu3cor := ifelse(cu3cor == "P3", "P4", cu3cor)]
> pGrpSan <- as.data.table(make_long(cuPCP, GRIN_spp, cu3cor, cu3, newGrp23))
> sankeyLevelsO <- c("O. nivara", "Oryza spp.", "O. rufipogon",
+             c("P4", "P2/P3", "P2", "P1"))
> pGrpSan <- pGrpSan[, node := factor(node, levels = sankeyLevelsO)]
> pGrpSan <- pGrpSan[, next_node := factor(next_node, levels = sankeyLevelsO)]
```

Plot.

```
> pdfFlNam <- "sankeyPGrpCUcor.pdf"
> showtext_auto()
> ggplot(data = pGrpSan, aes(x = x, next_x = next_x, node = node, next_node = next_node,
+                             fill = node, label = node)) +
+     geom_sankey(flow.alpha = 0.6, node.color = "grey30") +
+     geom_sankey_label(size = 5, color = "grey20", fill = "grey90", alpha = 0.8,
+             label = c(expression(italic("O. nivara")),
+                         expression(italic("Oryza spp.")),
+                         expression(italic("O. rufipogon")),
+                         "P4", "P2", "P1", "P4", "P2", "P1",
+                         "P4", "P2/P3", "P1")) +
+     scale_fill_manual(values = pGrp8Colors) +
+     scale_x_discrete(labels = c("species",
+                                 "Cornell\ncorrelation (7)",
+                                 "Cornell\nall traits",
+                                 "IRRI\nall traits")) +
+     theme_sankey(base_size = 20, base_family = "myriad") +
+     theme(strip.background = element_rect(fill = "grey95", linetype = "blank"),
+             legend.position = "none", axis.title = element_blank())
> ggsave(pdfFlNam, width = 8, height = 6, units = "in",
+             device = "pdf", useDingbats = FALSE)
> cat("\\includegraphics[width=0.9\\textwidth]{", pdfFlNam, "}\n\n", sep = "")
```

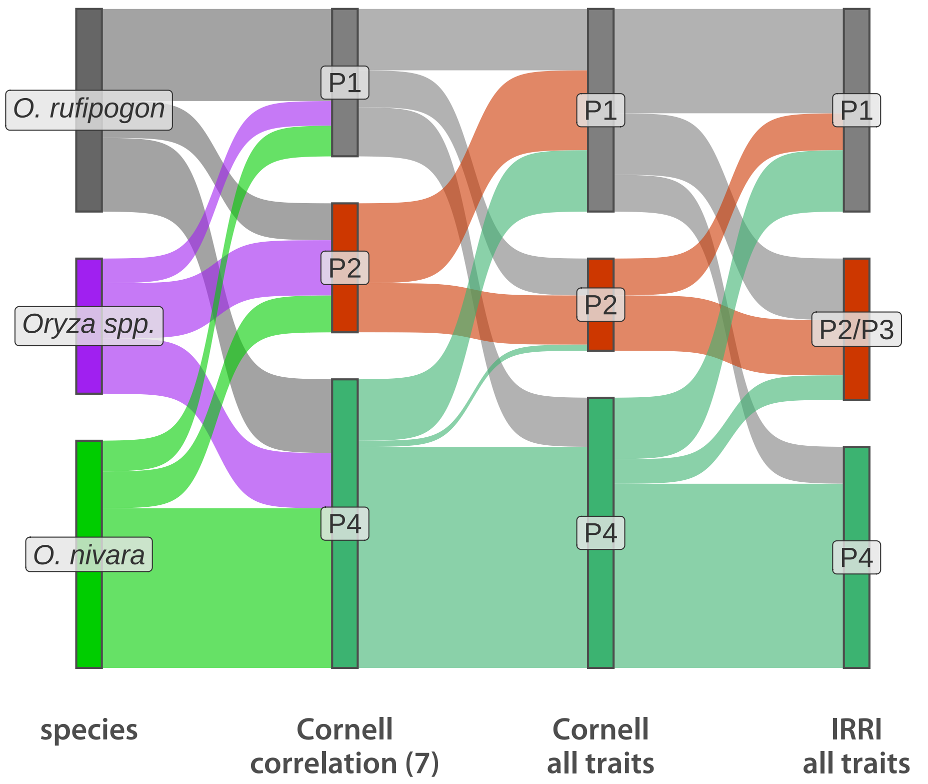
O. rufipogon
Oryza spp.
O. nivara
P1
P2
P4
P1
P2
P4
P1
P2/P3
P4
species
Cornell
correlation (7)
Cornell
all traits
IRRI
all traits
