# Supplementary material for: Phenotypic Variation and the Impact of Admixture in the Oryza rufipogon Species Complex (ORSC)
Source: Front Plant Sci. 2022 Jun 13;13:787703. doi: 10.3389/fpls.2022.787703 (PMC9235872; doi:10.3389/fpls.2022.787703)
Supplement: Supplementary file 8 [file Data_Sheet_1.ZIP › Rplots.pdf]

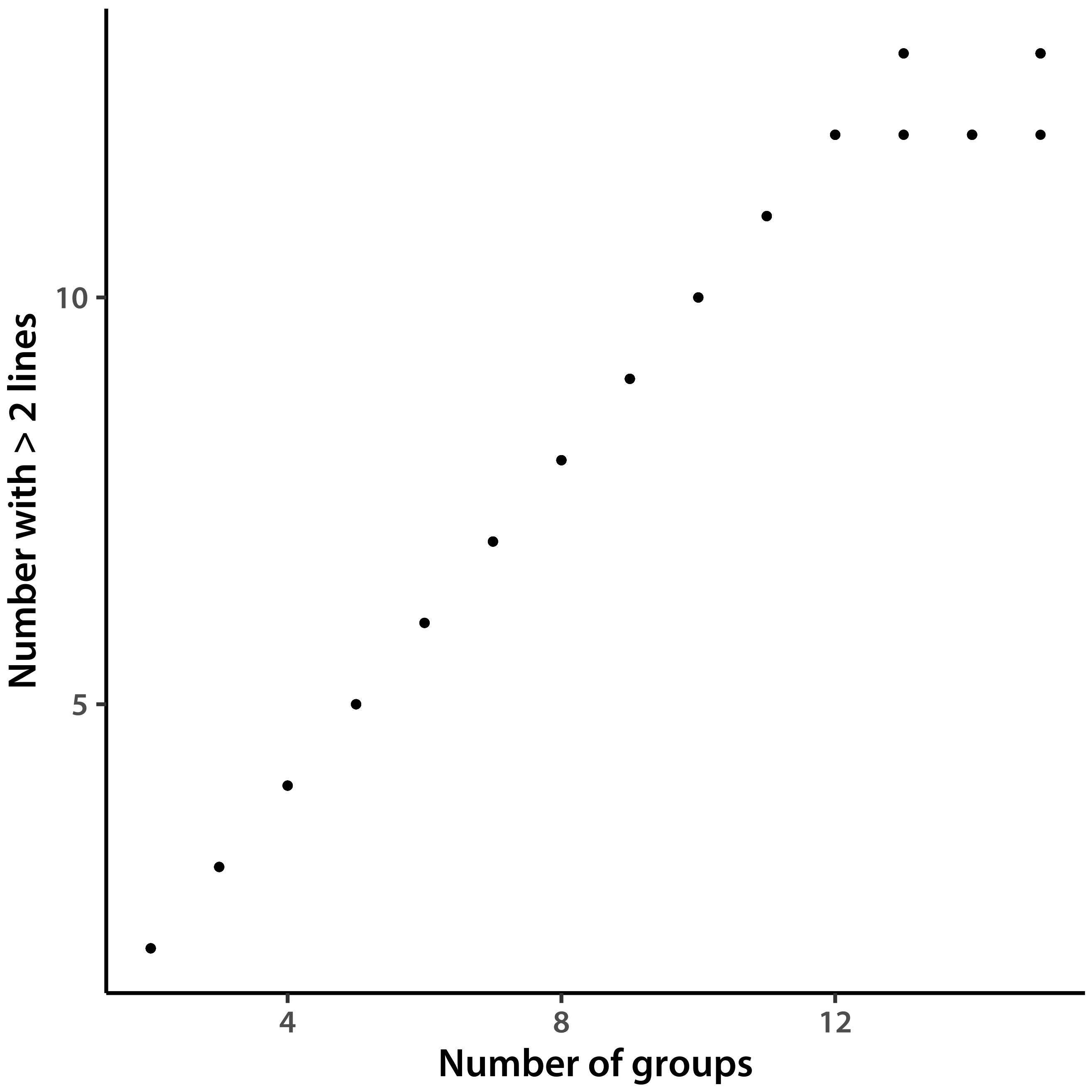

Number with > 2 lines
10
5
4
8
12
Number of groups

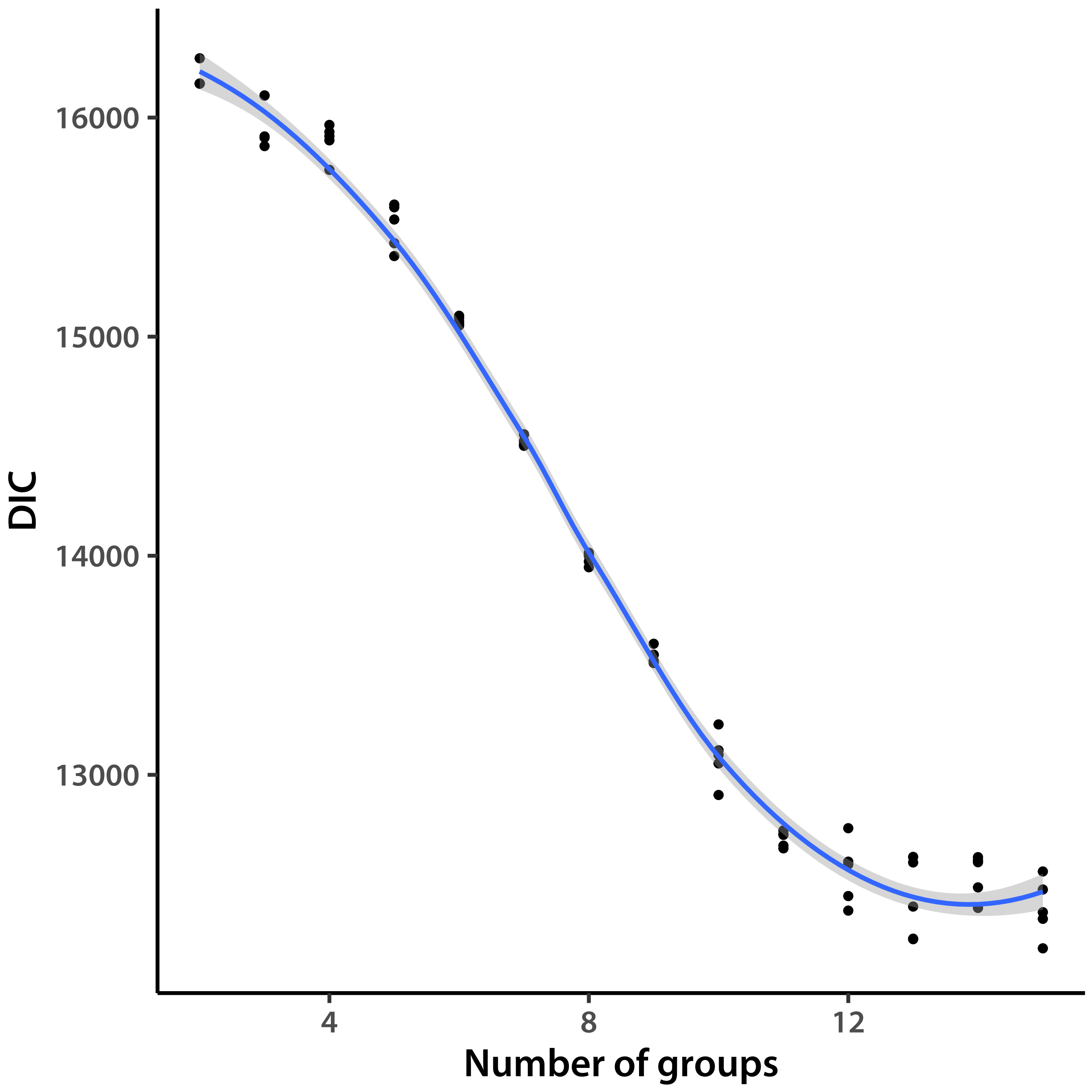
DIC
16000
15000
14000
13000
4
8
12
Number of groups

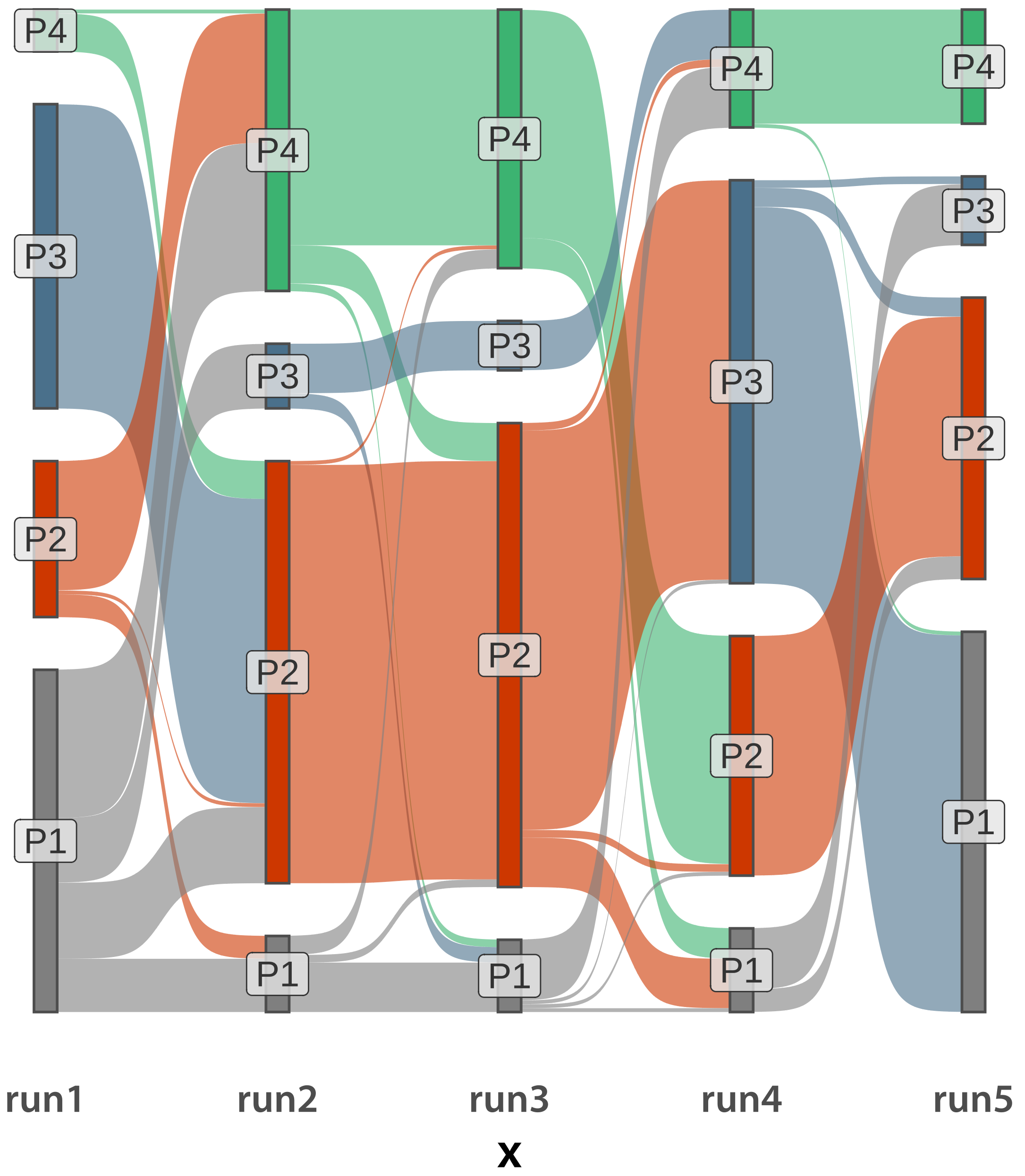
P4
P3
P2
P1
P4
P3
P2
P1
P4
P3
P2
P1
P4
P3
P2
P1
P4
P3
P2
P1
run1
run2
run3
run4
run5
x

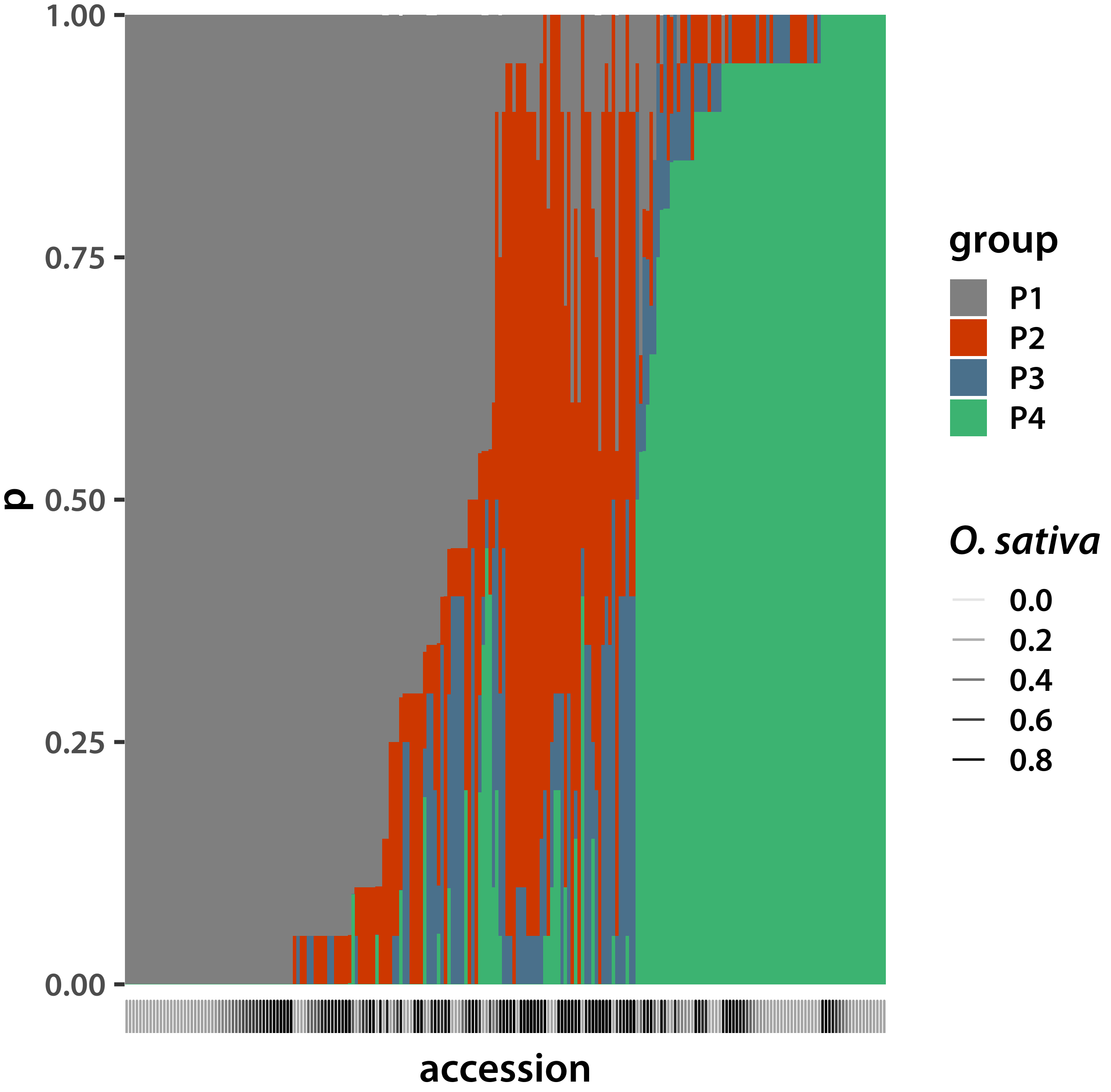
1.00
0.75
0.50
0.25
0.00
p
accession
group
P1
P2
P3
P4
O. sativa
0.0
0.2
0.4
0.6
0.8

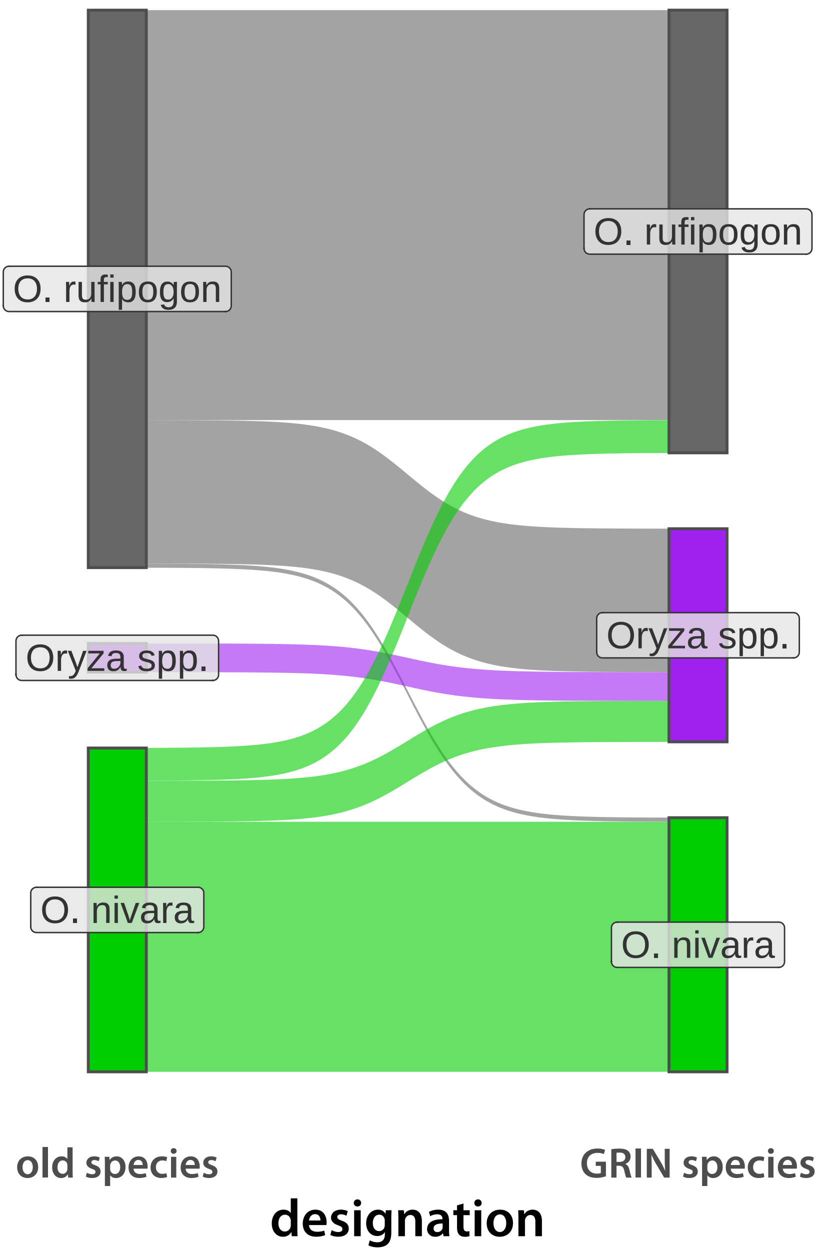
O. rufipogon
Oryza spp.
O. nivara
O. rufipogon
Oryza spp.
O. nivara
old species
GRIN species
designation

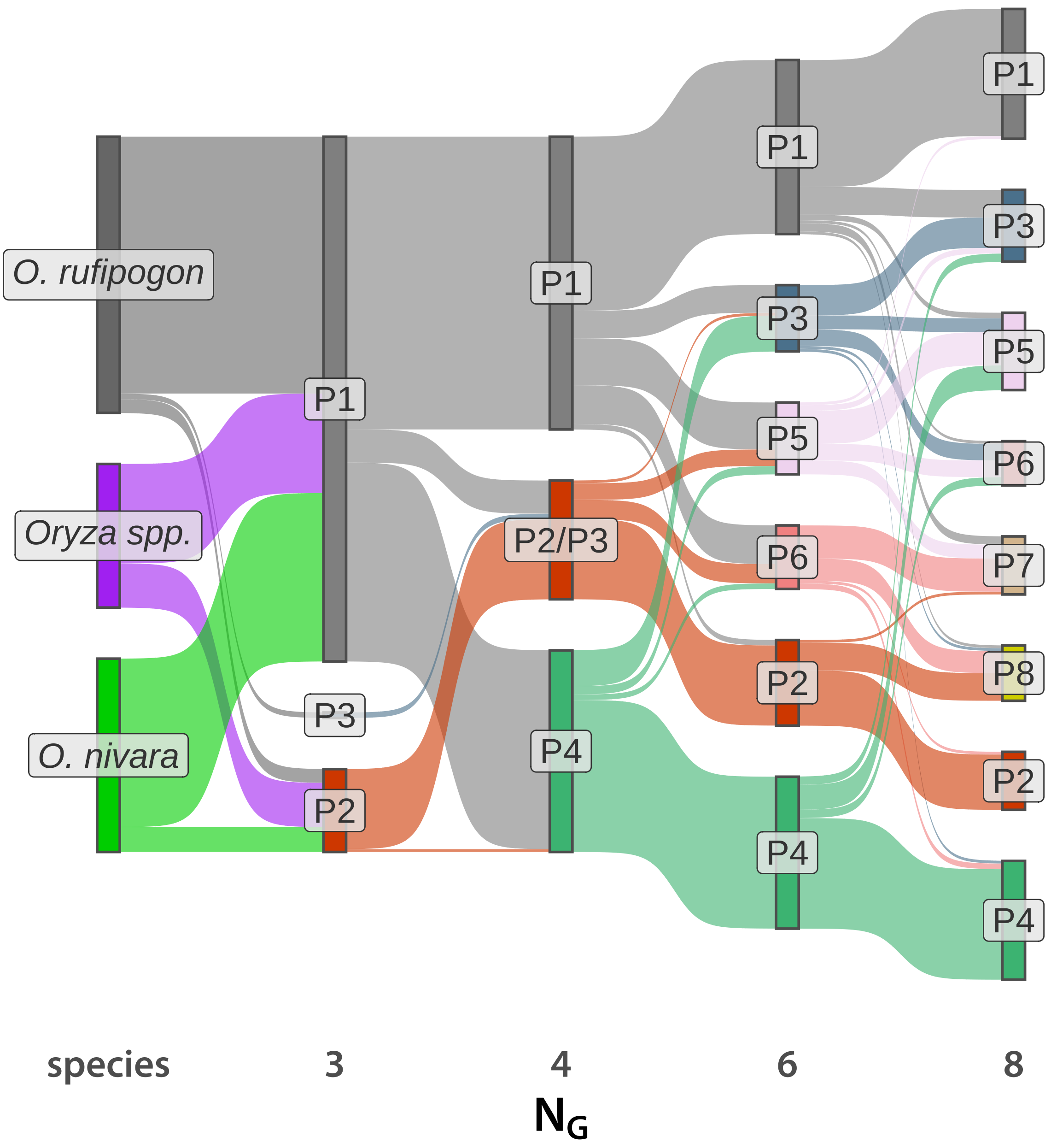
O. rufipogon
Oryza spp.
O. nivara
P1
P2
P3
P1
P2/P3
P4
P1
P3
P5
P6
P2
P4
P1
P3
P5
P6
P7
P8
P2
P4
species
3
4
6
8
N_G

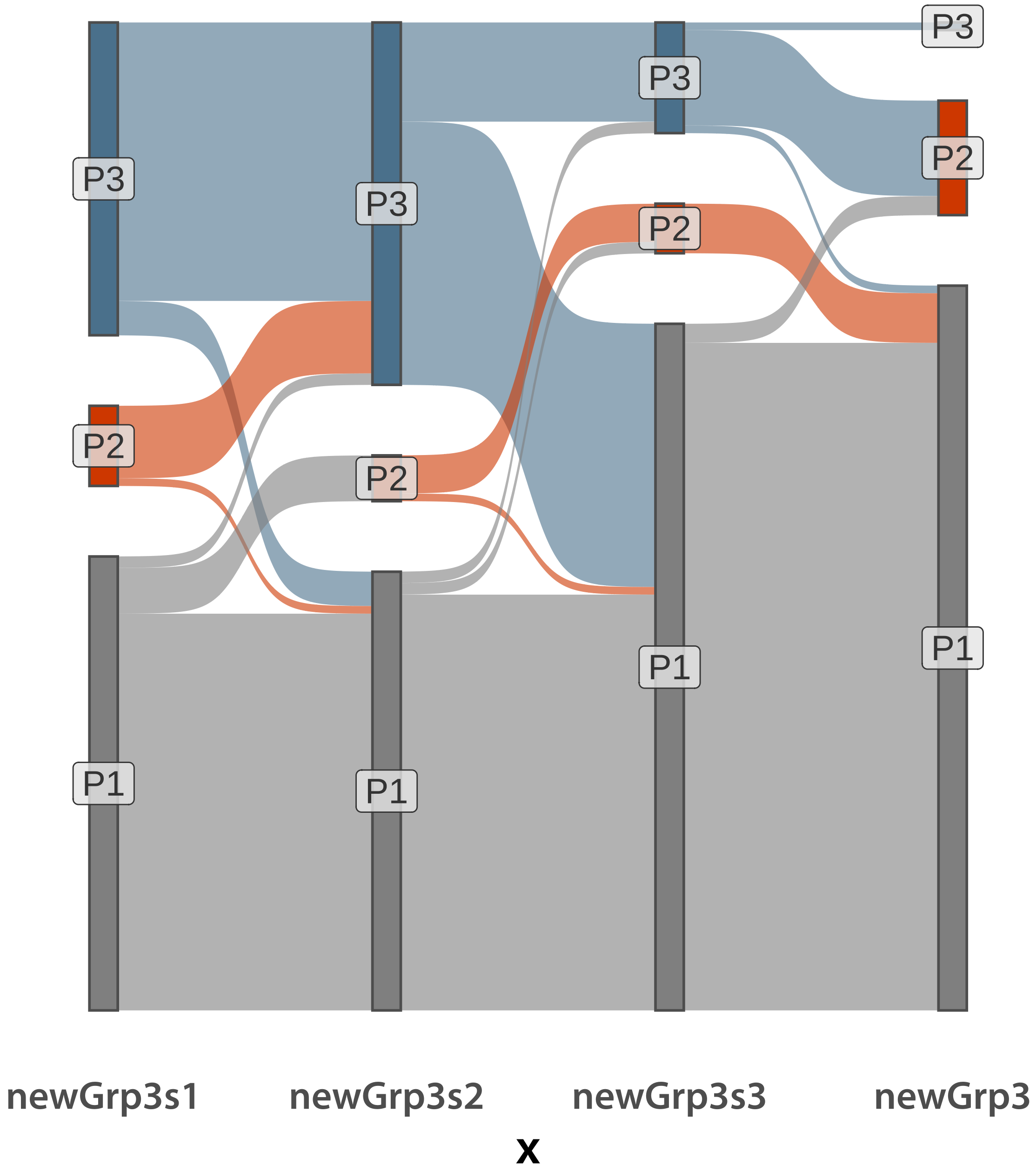
P3
P2
P1
P3
P2
P1
P3
P2
P1
P3
P2
P1
newGrp3s1
newGrp3s2
newGrp3s3
newGrp3
x

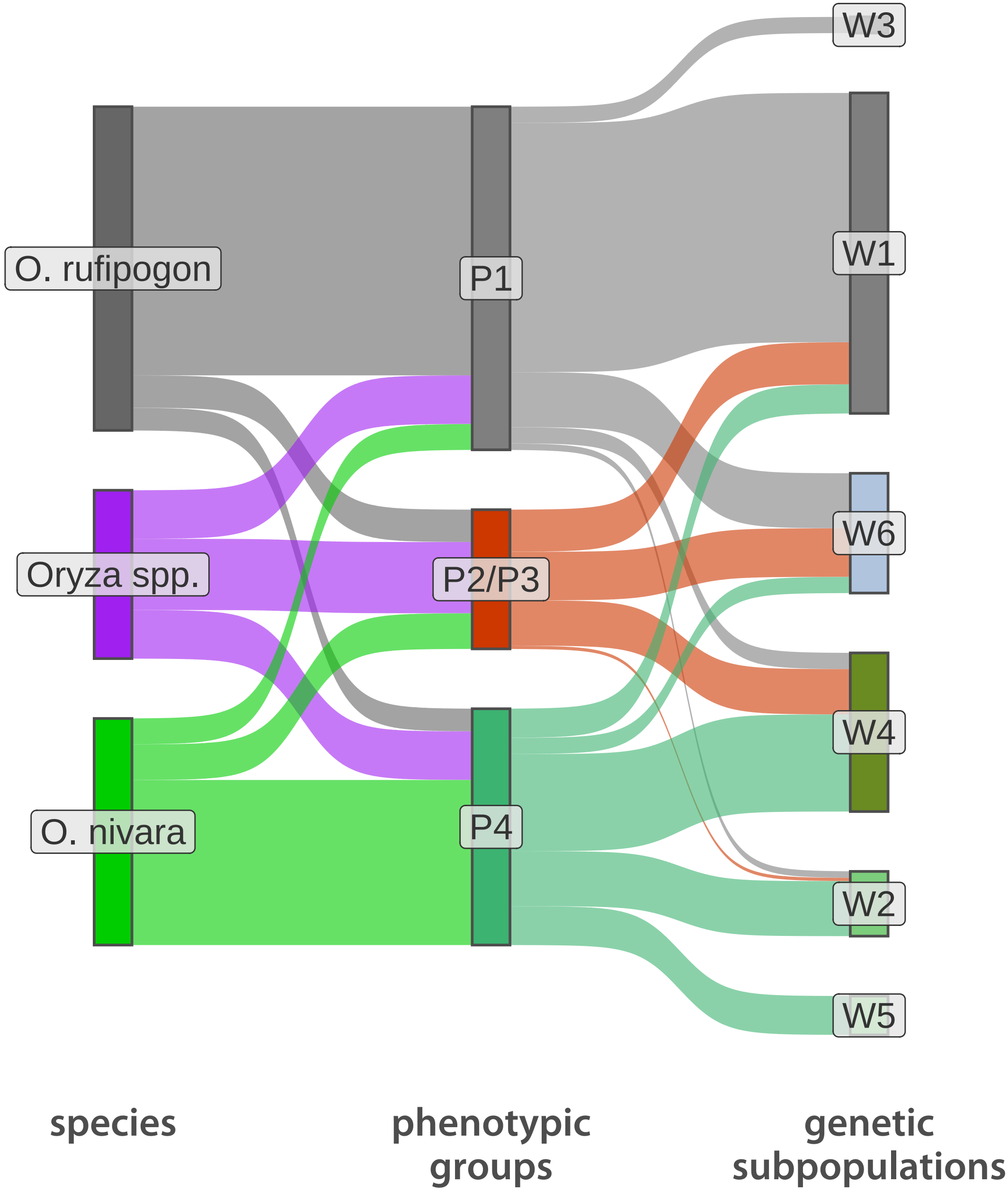
O. rufipogon
Oryza spp.
O. nivara
P1
P2/P3
P4
W3
W1
W6
W4
W2
W5
species
phenotypic groups
genetic subpopulations

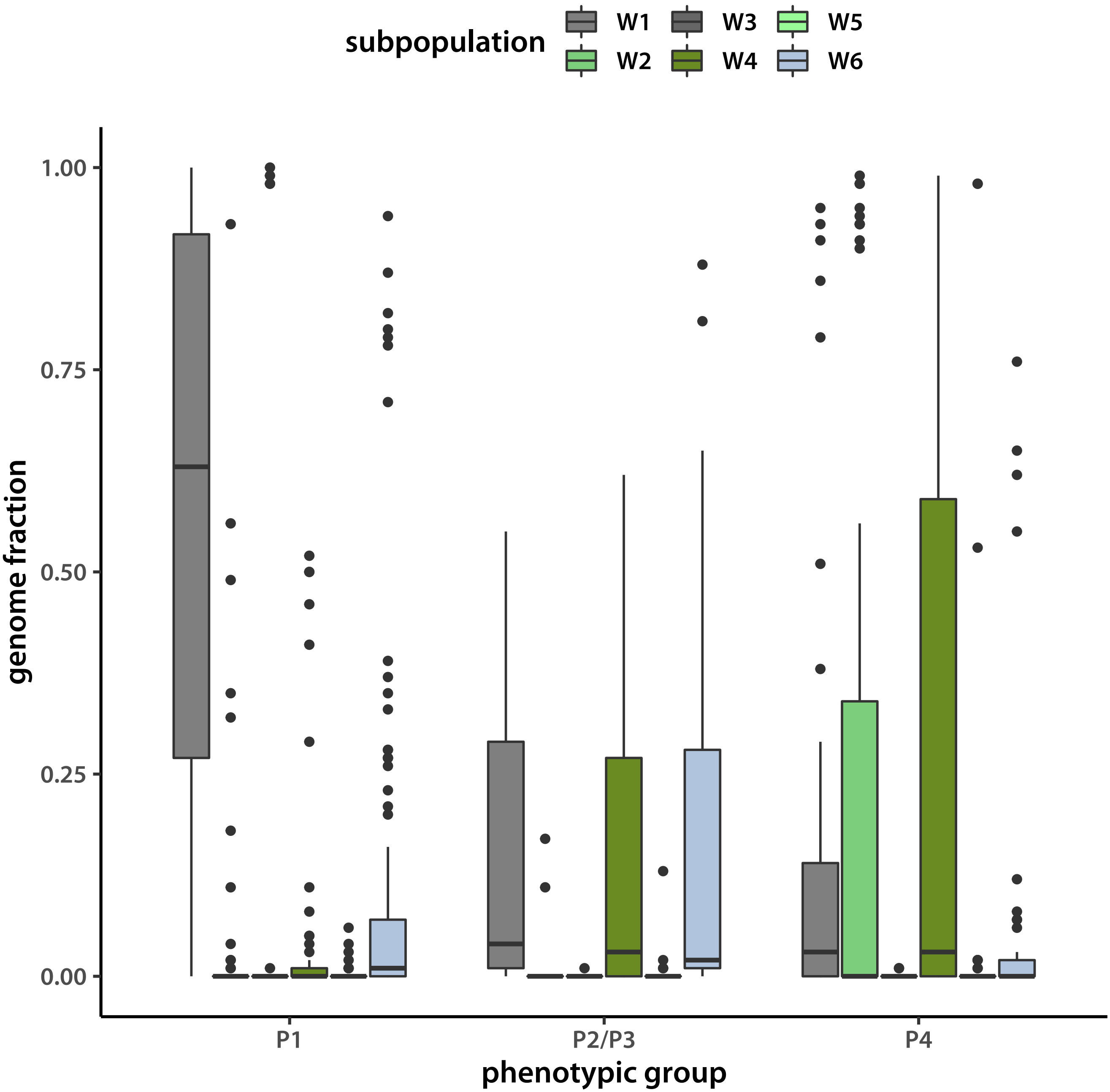
subpopulation
W1
W2
W3
W4
W5
W6
1.00
0.75
0.50
0.25
0.00
genome fraction
P1
P2/P3
P4
phenotypic group

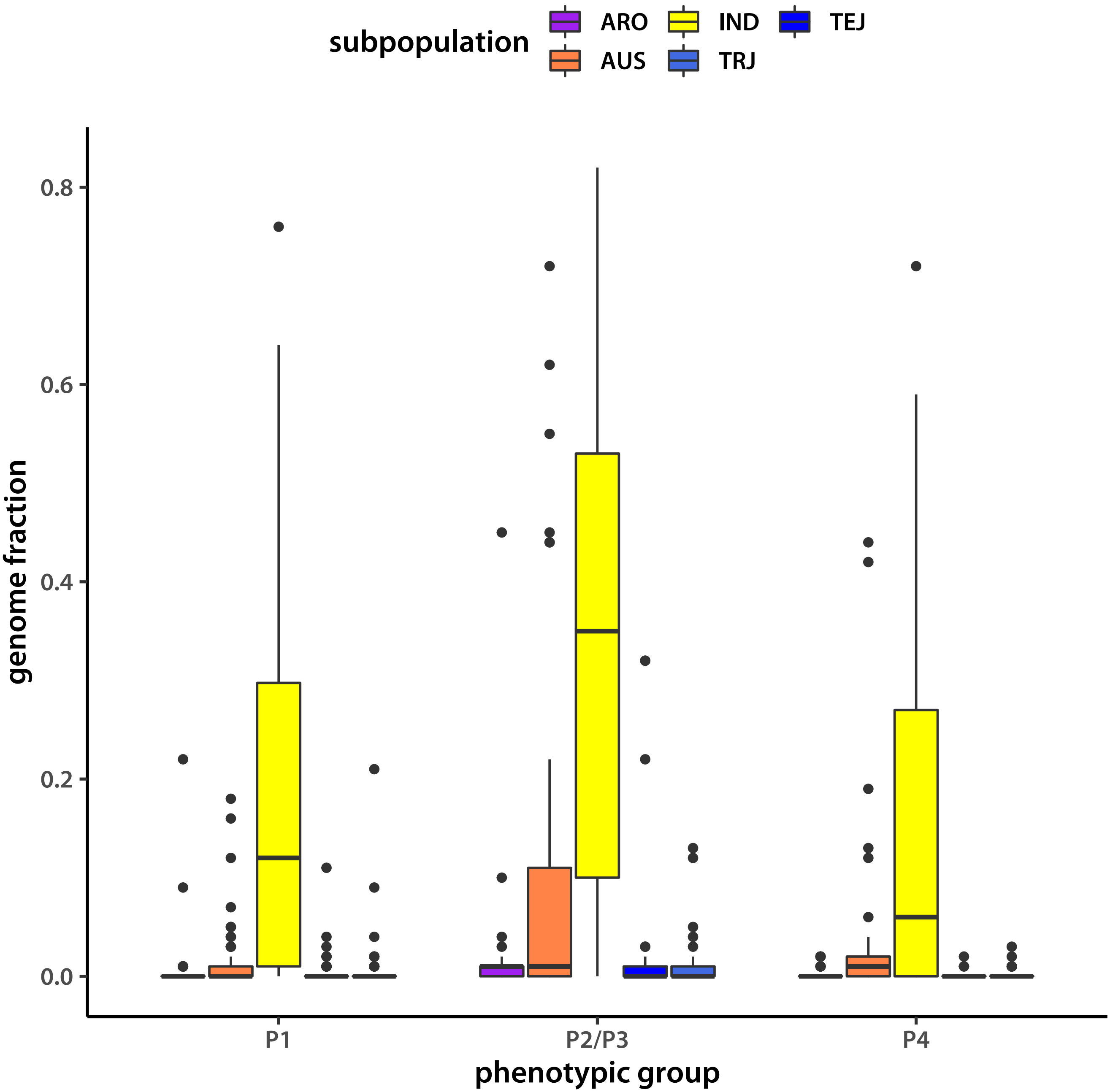
subpopulation
ARO
AUS
IND
TRJ
TEJ
genome fraction
0.0
0.2
0.4
0.6
0.8
P1
P2/P3
P4
phenotypic group

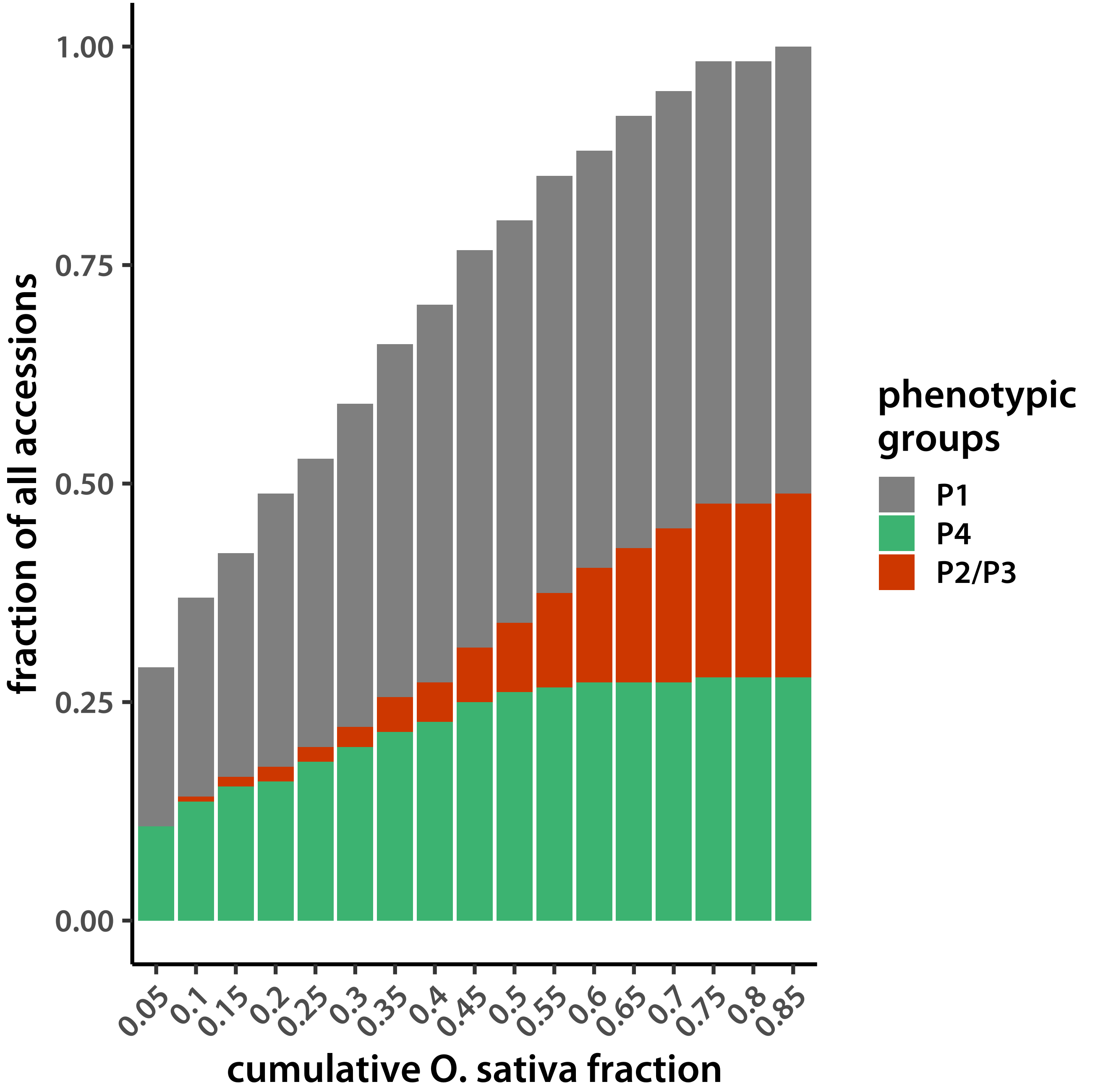

fraction of all accessions
1.00
0.75
0.50
0.25
0.00
0.05
0.1
0.15
0.2
0.25
0.3
0.35
0.4
0.45
0.5
0.55
0.6
0.65
0.7
0.75
0.8
0.85
cumulative O. sativa fraction
phenotypic groups
P1
P4
P2/P3

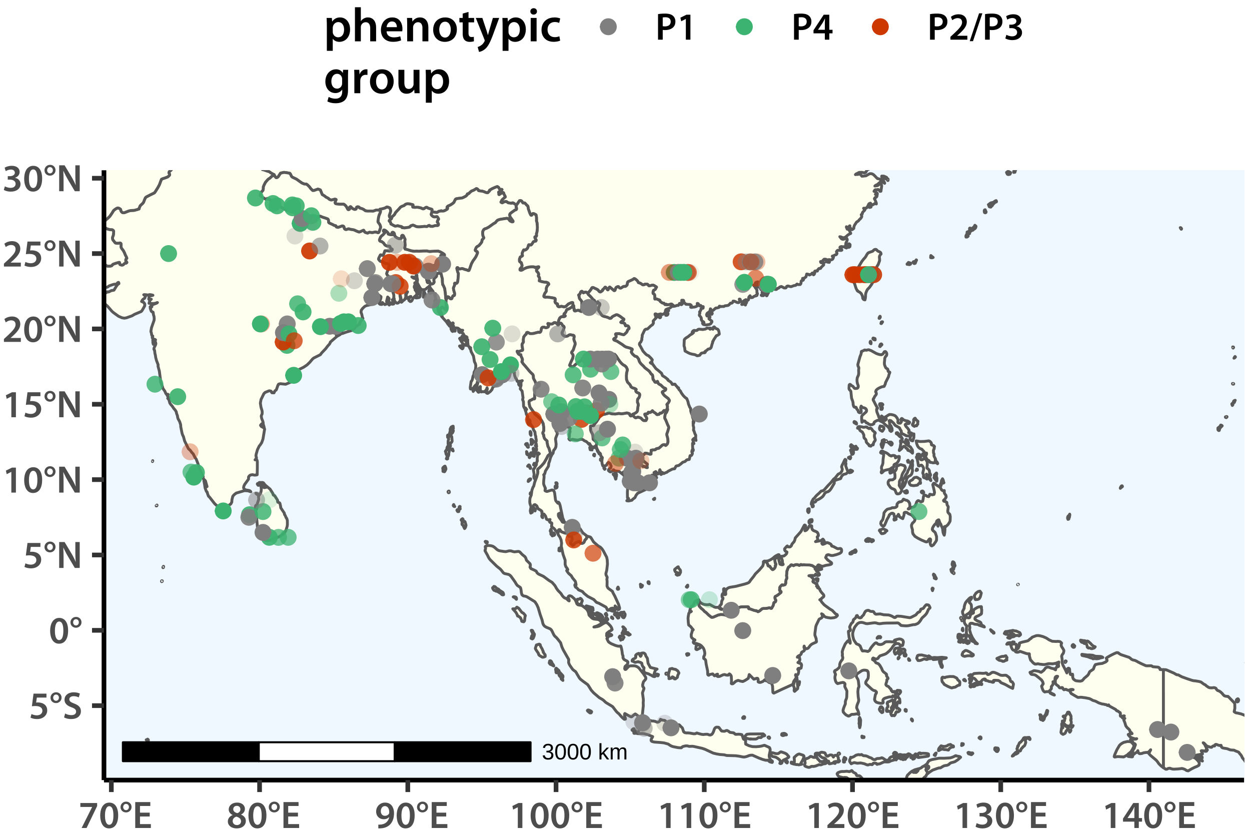
phenotypic group
P1
P4
P2/P3
30°N
25°N
20°N
15°N
10°N
5°N
0°
5°S
3000 km
70°E
80°E
90°E
100°E
110°E
120°E
130°E
140°E

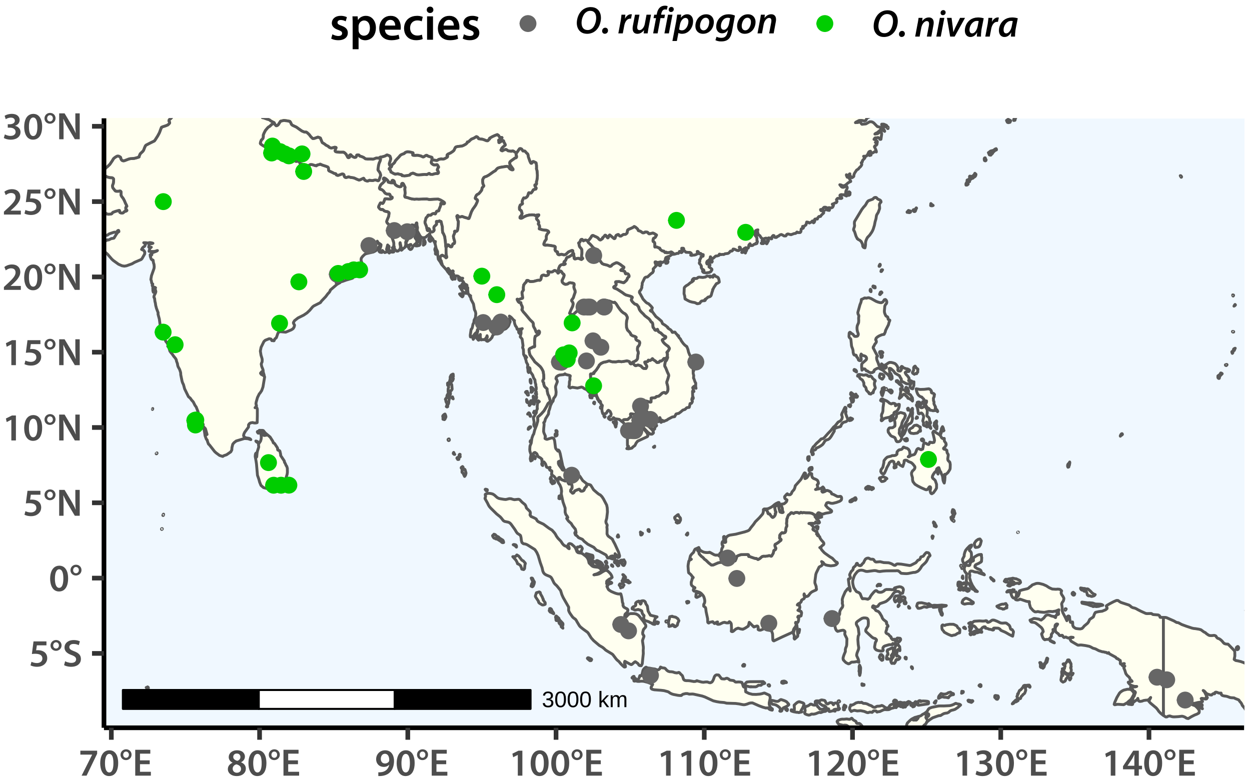
species
O. rufipogon
O. nivara
30°N
25°N
20°N
15°N
10°N
5°N
0°
5°S
3000 km
70°E
80°E
90°E
100°E
110°E
120°E
130°E
140°E

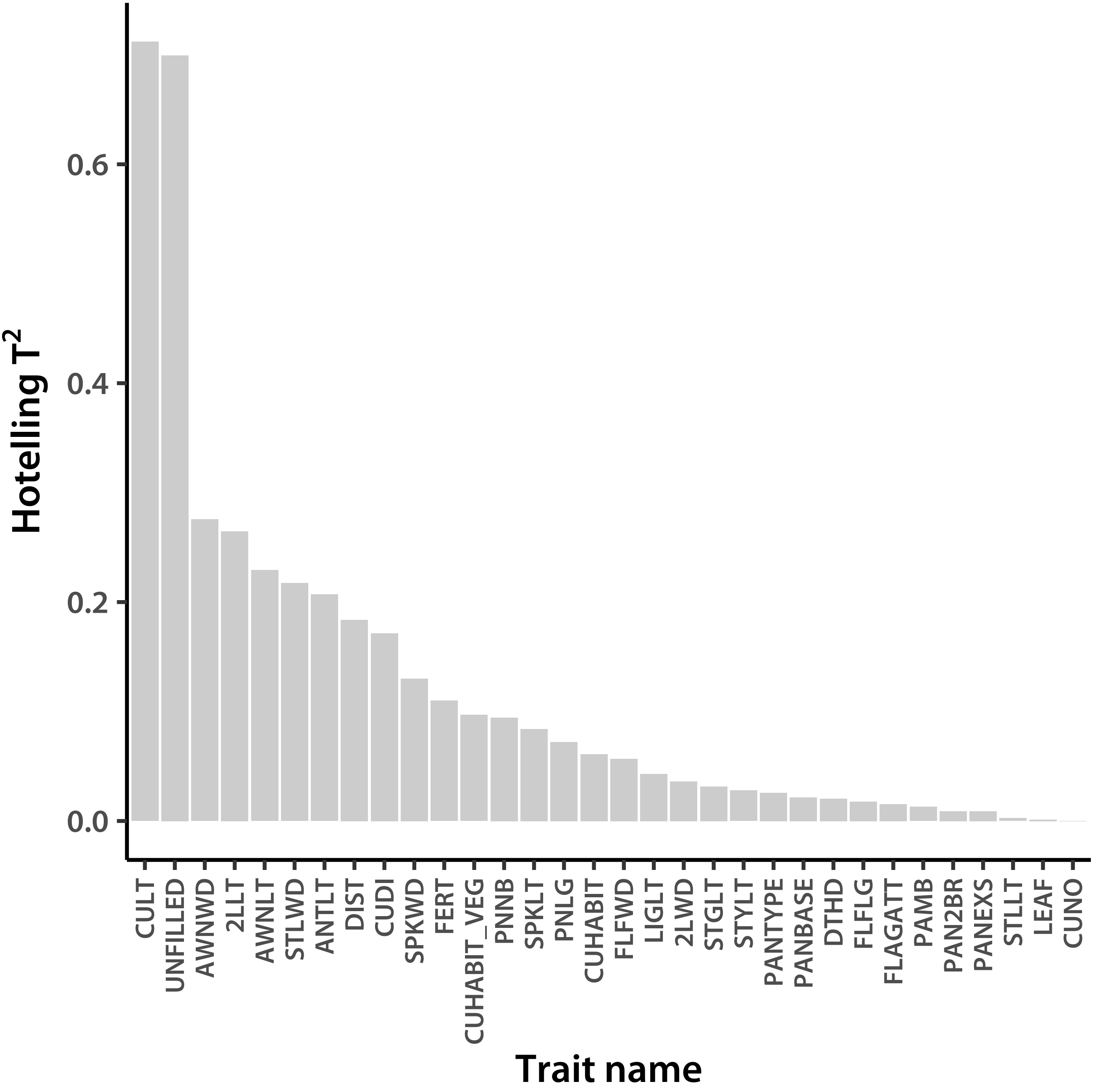

Hotelling T^2
0.6
0.4
0.2
0.0
CULT
UNFILLED
AWNWD
2LLT
AWNLT
STLWD
ANTLT
DIST
CUDI
SPKWD
FERT
CUHABIT_VEG
PNNB
SPKLT
PNLG
CUHABIT
FLFWD
LIGLT
2LWD
STGLT
STYLT
PANTYPE
PANBASE
DTHD
FLFLG
FLAGATT
PAMB
PAN2BR
PANEXS
STLLT
LEAF
CUNO
Trait name

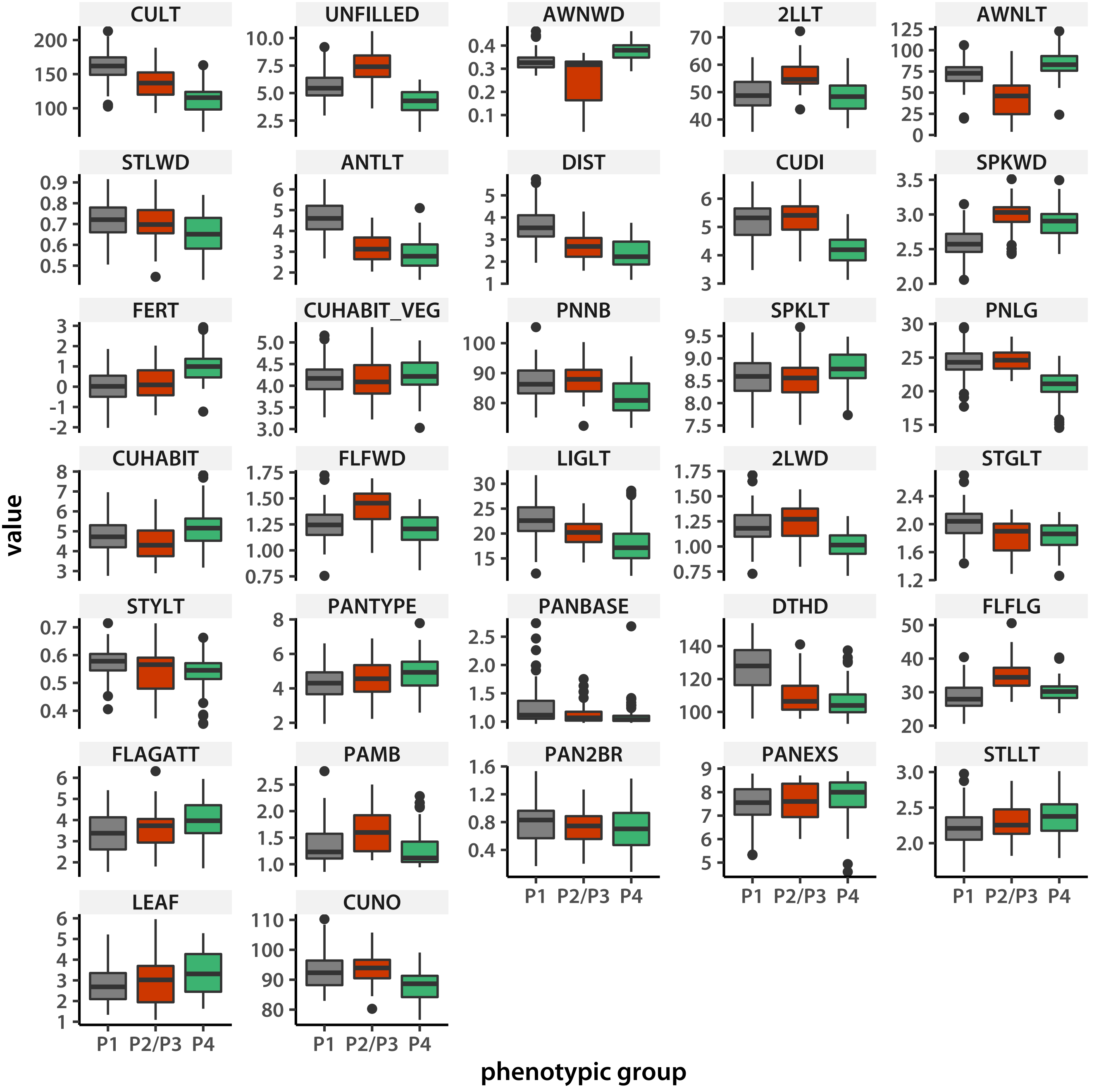
CULT
UNFILLED
AWNWD
2LLT
AWNLT
STLWD
ANTLT
DIST
CUDI
SPKWD
FERT
CUHABIT_VEG
PNNB
SPKLT
PNLG
CUHABIT
FLFWD
LIGLT
2LWD
STGLT
STYLT
PANTYPE
PANBASE
DTHD
FLFLG
FLAGATT
PAMB
PAN2BR
PANEXS
STLLT
LEAF
CUNO
value
P1
P2/P3
P4
phenotypic group

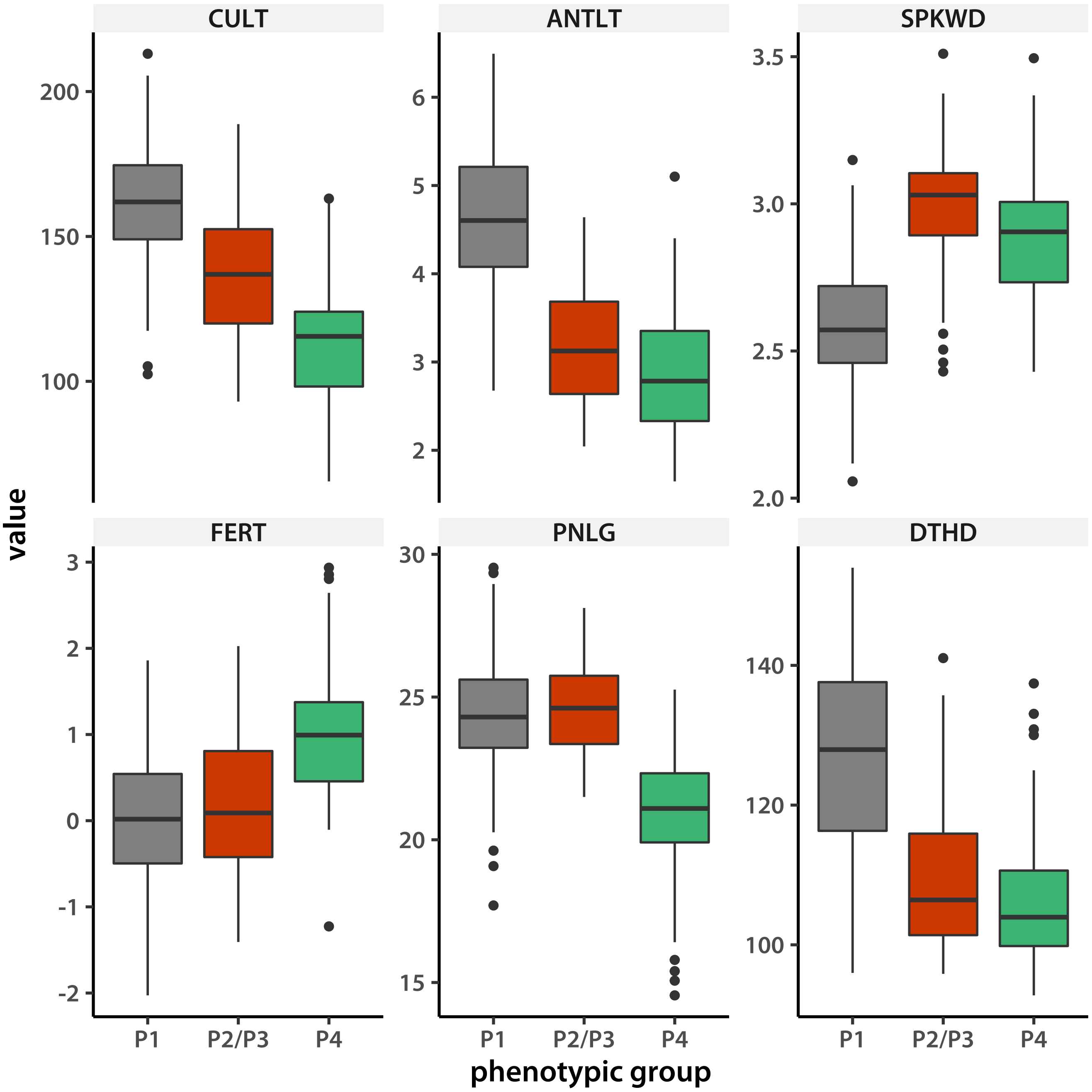

CULT
ANTLT
SPKWD
FERT
PNLG
DTHD
value
phenotypic group
P1
P2/P3
P4
200
150
100
6
5
4
3
2
3.5
3.0
2.5
2.0
1
0
-1
-2
30
25
20
15
140
120
100

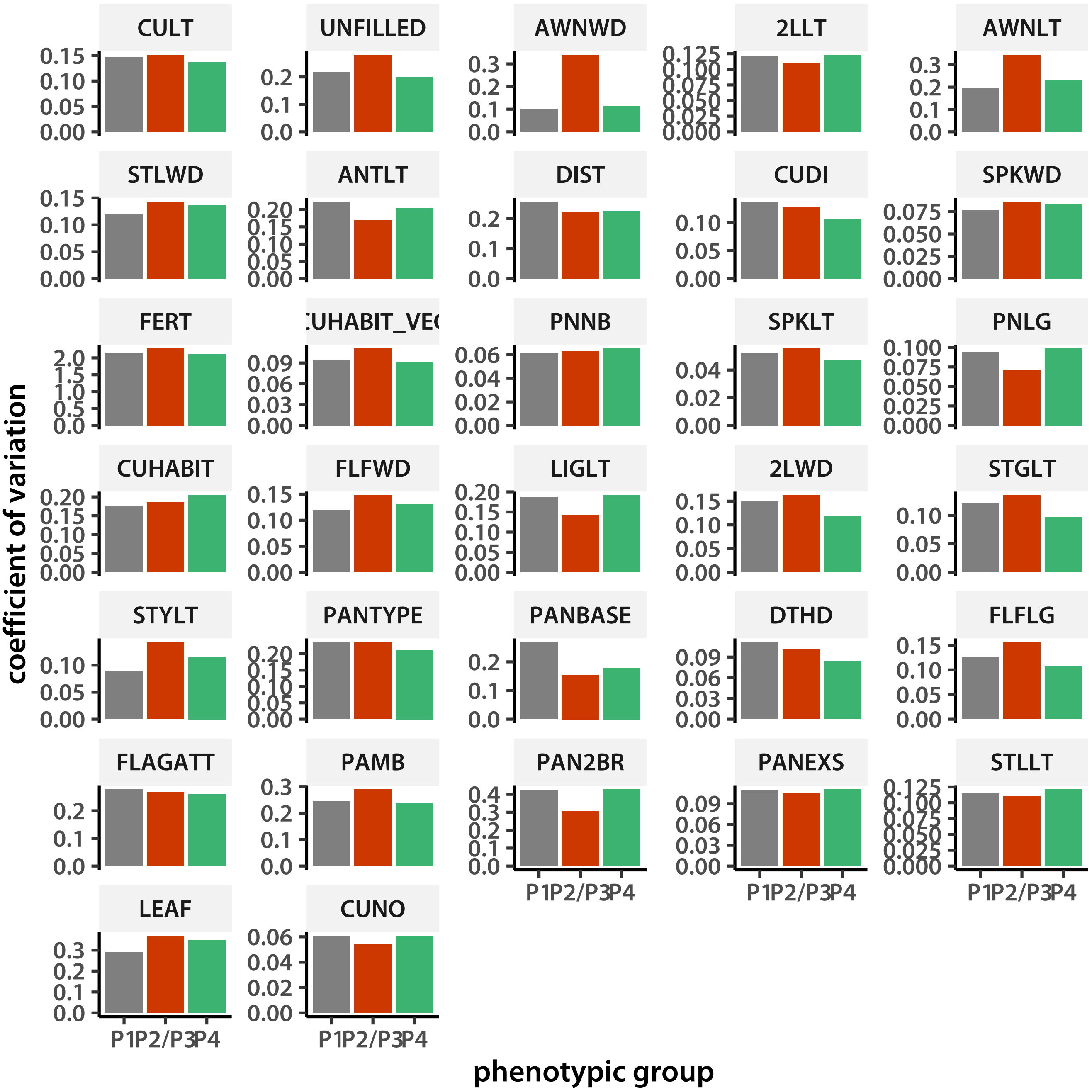
CULT
UNFILLED
AWNWD
2LLT
AWNLT
STLWD
ANTLT
DIST
CUDI
SPKWD
FERT
CUHABIT_VEG
PNNB
SPKLT
PNLG
CUHABIT
FLFWD
LIGLT
2LWD
STGLT
STYLT
PANTYPE
PANBASE
DTHD
FLFLG
FLAGATT
PAMB
PAN2BR
PANEXS
STLLT
LEAF
CUNO
P1
P2/P3
P4
coefficient of variation
phenotypic group

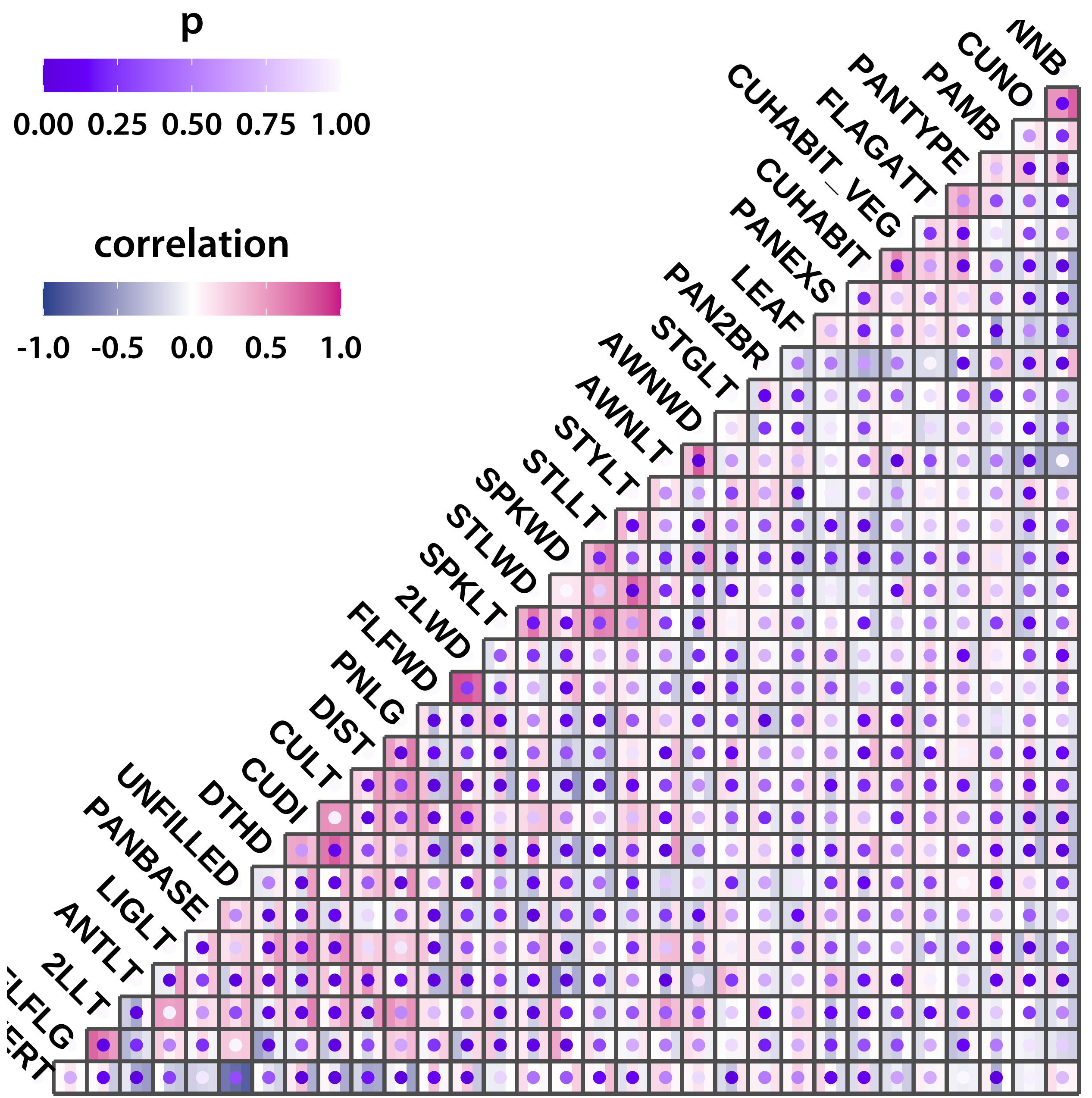
p
0.00 0.25 0.50 0.75 1.00
correlation
-1.0 -0.5 0.0 0.5 1.0
CUNO
PAMB
PANTYPE
FLAGATT
CUHABIT_VEG
CUHABIT
PANEXS
LEAF
PAN2BR
STGLT
AWNWD
AWNLT
STYLT
STLLT
SPKWD
STLWD
SPKLT
2LWD
FLFWD
PNLG
DIST
CULT
CUDI
DTHD
UNFILLED
PANBASE
LIGLT
ANTLT
2LLT

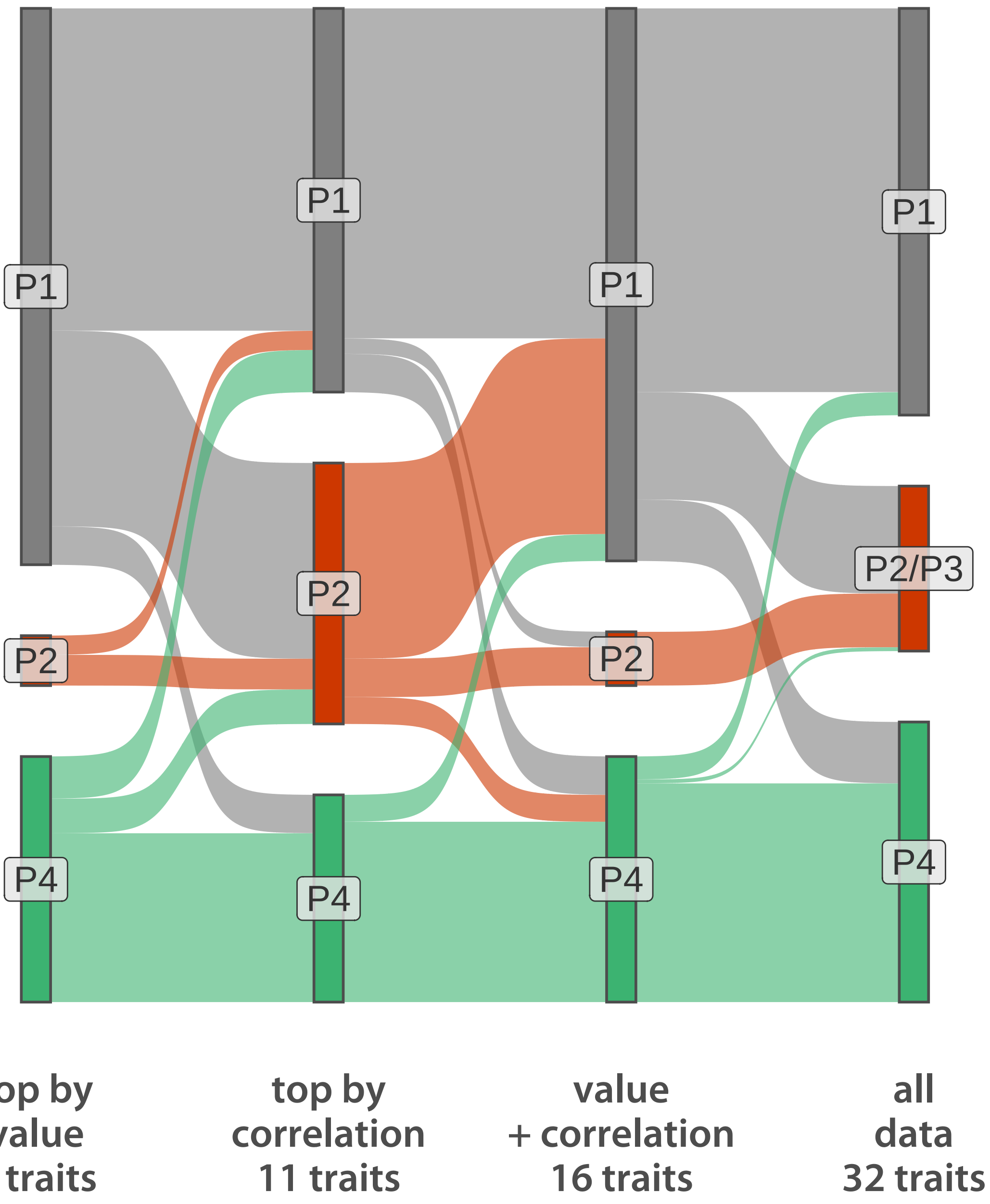
P1
P2
P4
P1
P2
P4
P1
P2
P4
P1
P2/P3
P4
top by
value
9 traits
top by
correlation
11 traits
value
+ correlation
16 traits
all
data
32 traits

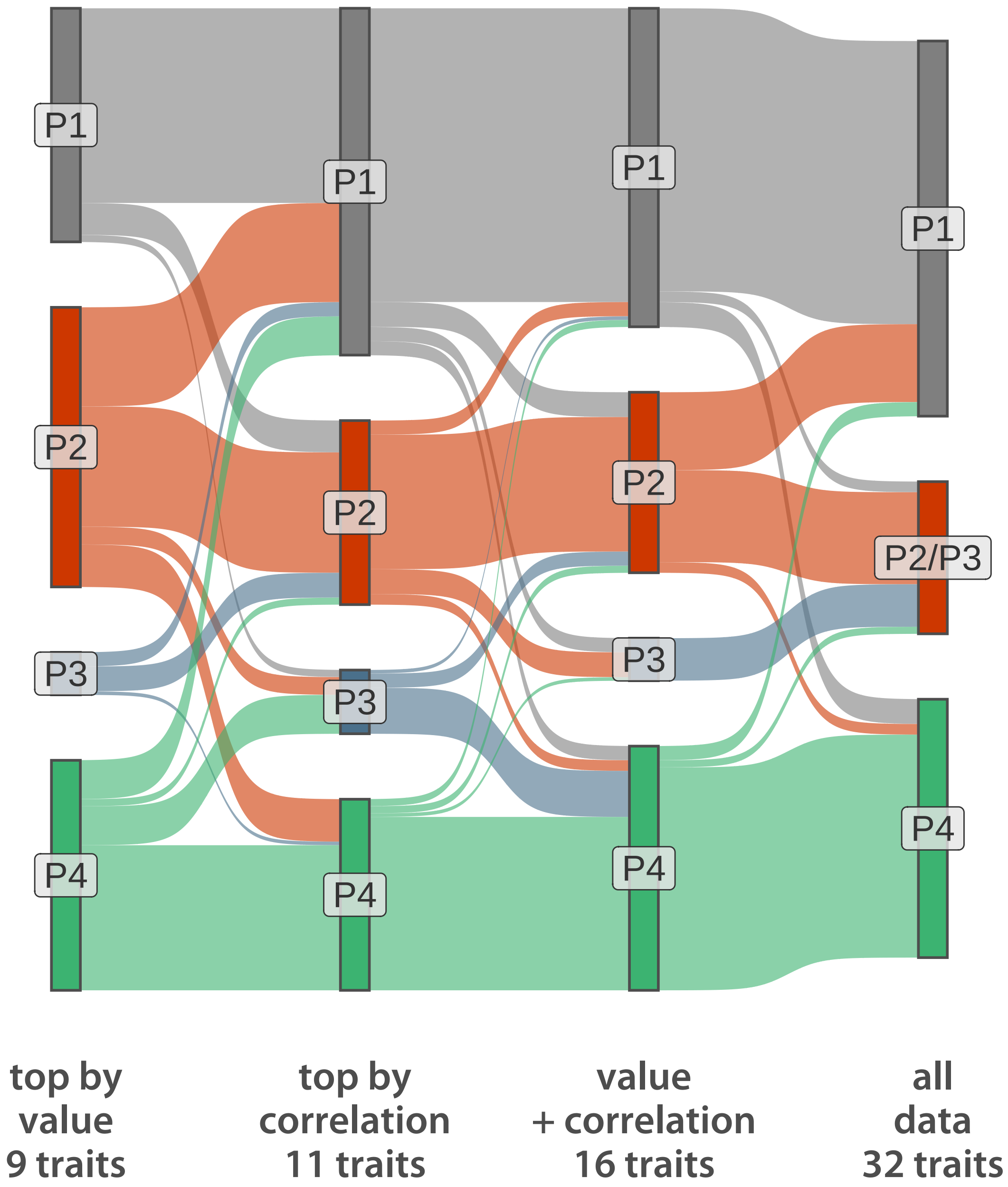
P1
P2
P3
P4
P1
P2
P3
P4
P1
P2
P3
P4
P1
P2/P3
P4
top by value 9 traits
top by correlation 11 traits
value + correlation 16 traits
all data 32 traits

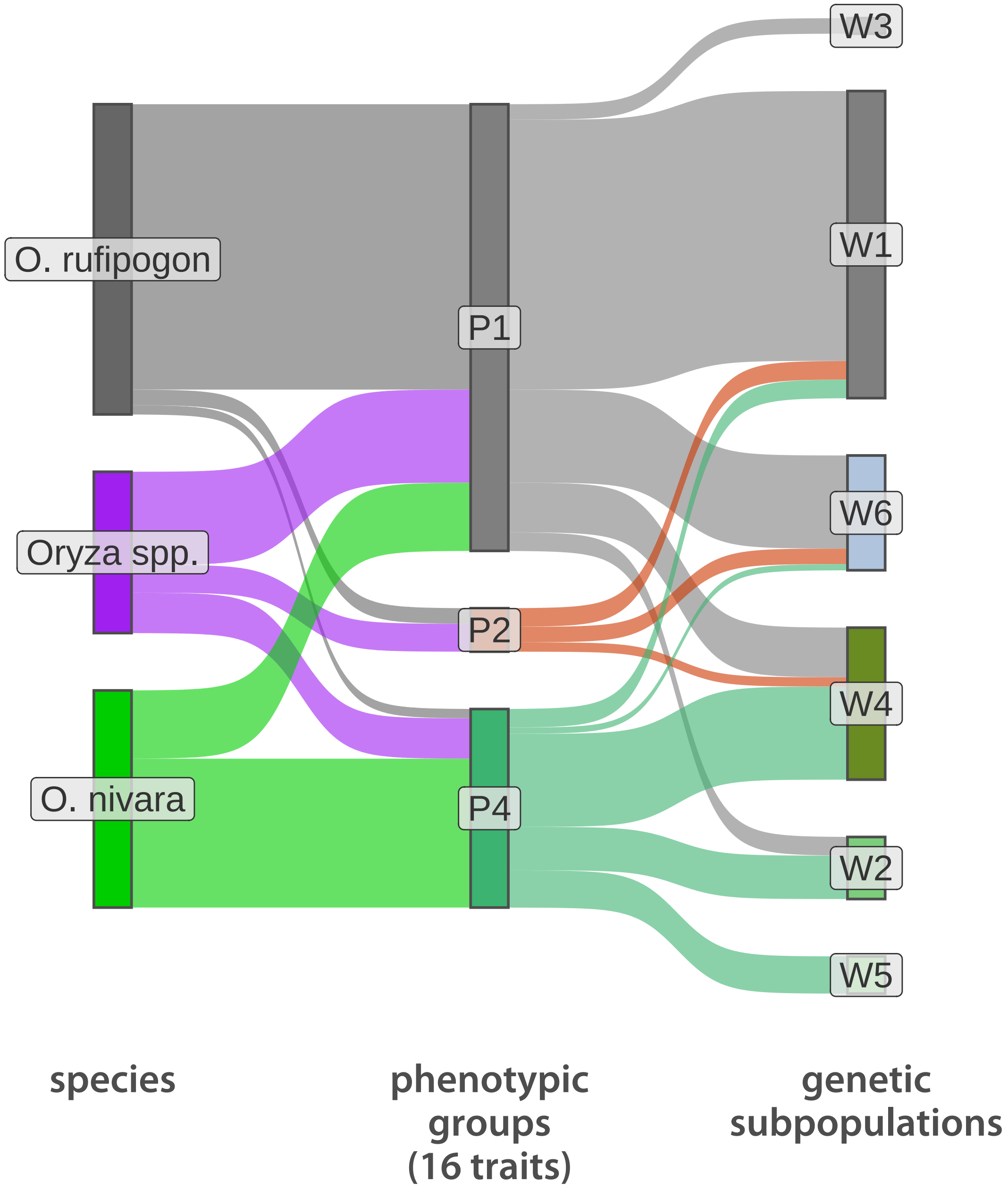
O. rufipogon
Oryza spp.
O. nivara
P1
P2
P4
W3
W1
W6
W4
W2
W5
species
phenotypic
groups
(16 traits)
genetic
subpopulations

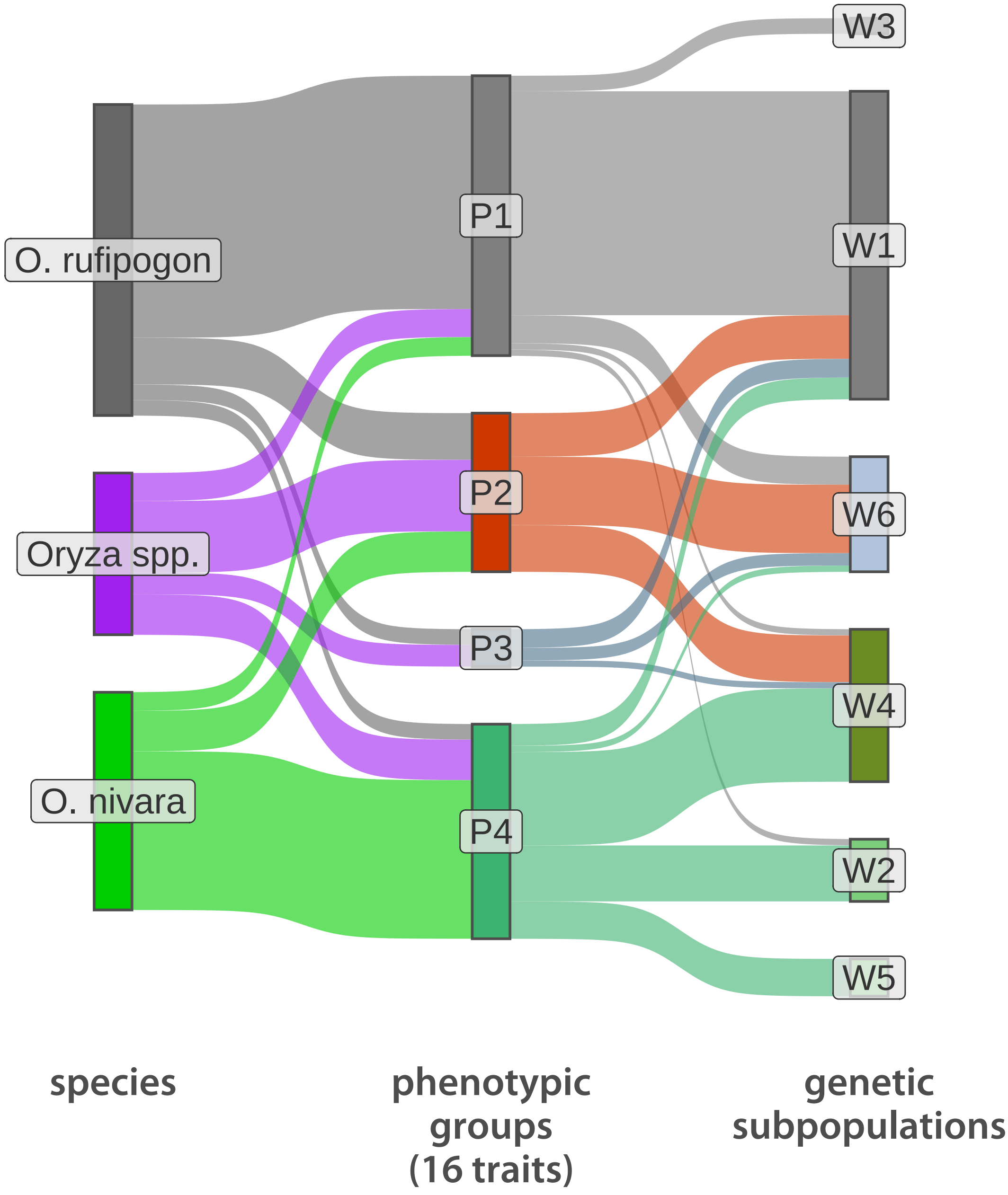
W3
O. rufipogon
P1
W1
P2
W6
Oryza spp.
P3
W4
O. nivara
P4
W2
W5
species
phenotypic
groups
(16 traits)
genetic
subpopulations

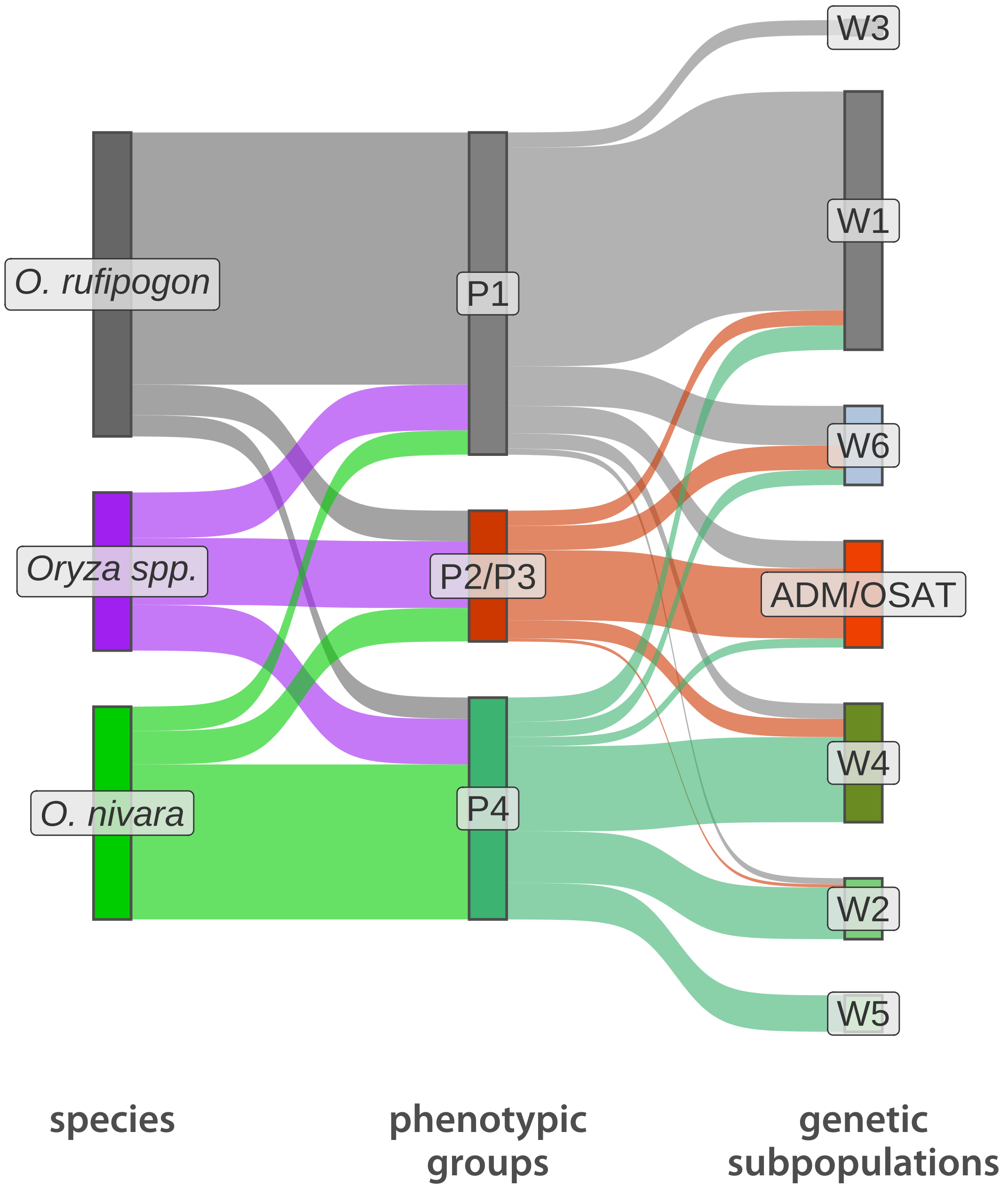
O. rufipogon
Oryza spp.
O. nivara
P1
P2/P3
P4
W3
W1
W6
ADM/OSAT
W4
W2
W5
species
phenotypic groups
genetic subpopulations

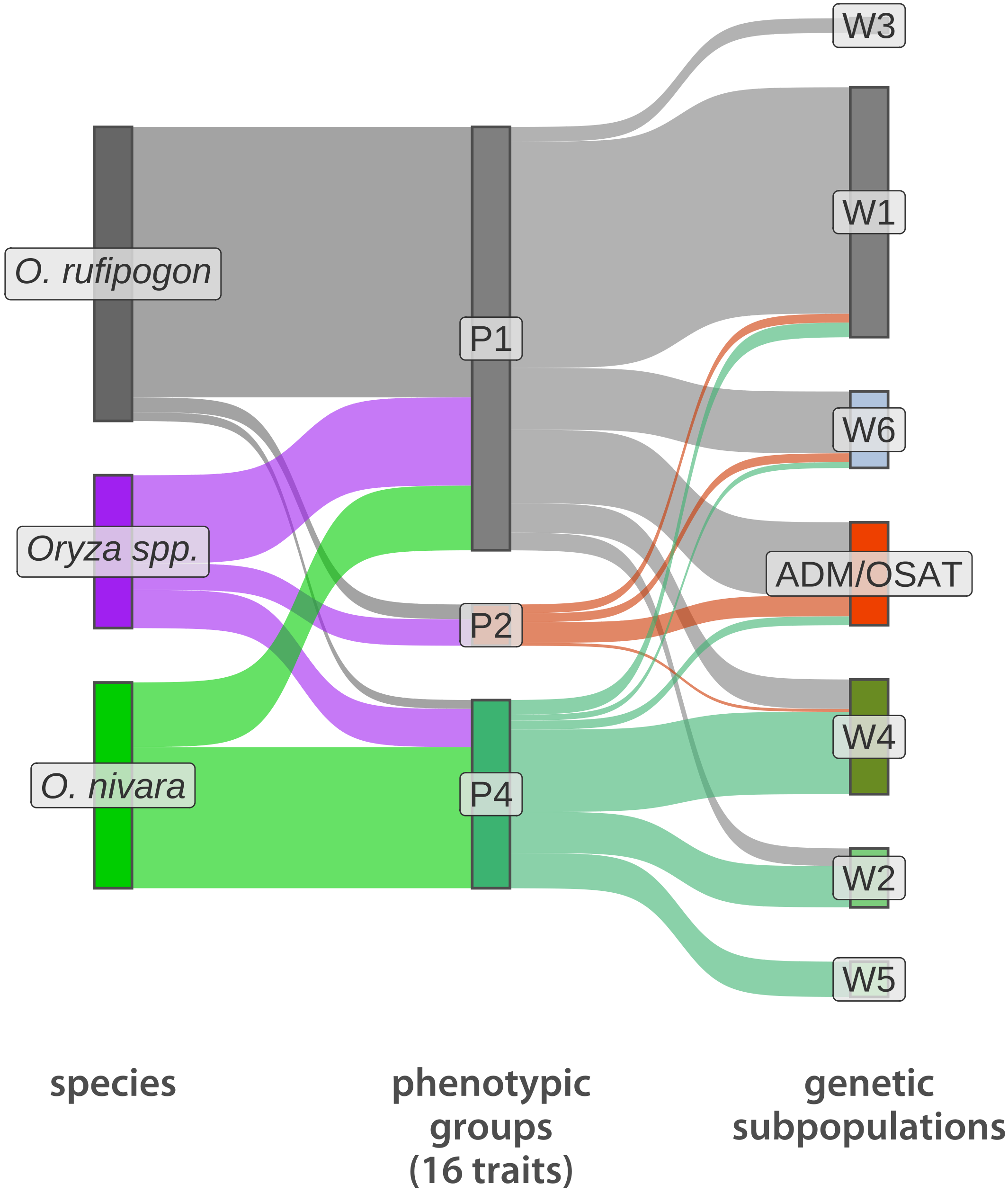
W3
W1
O. rufipogon
P1
W6
Oryza spp.
ADM/OSAT
P2
W4
O. nivara
P4
W2
W5
species
phenotypic groups (16 traits)
genetic subpopulations

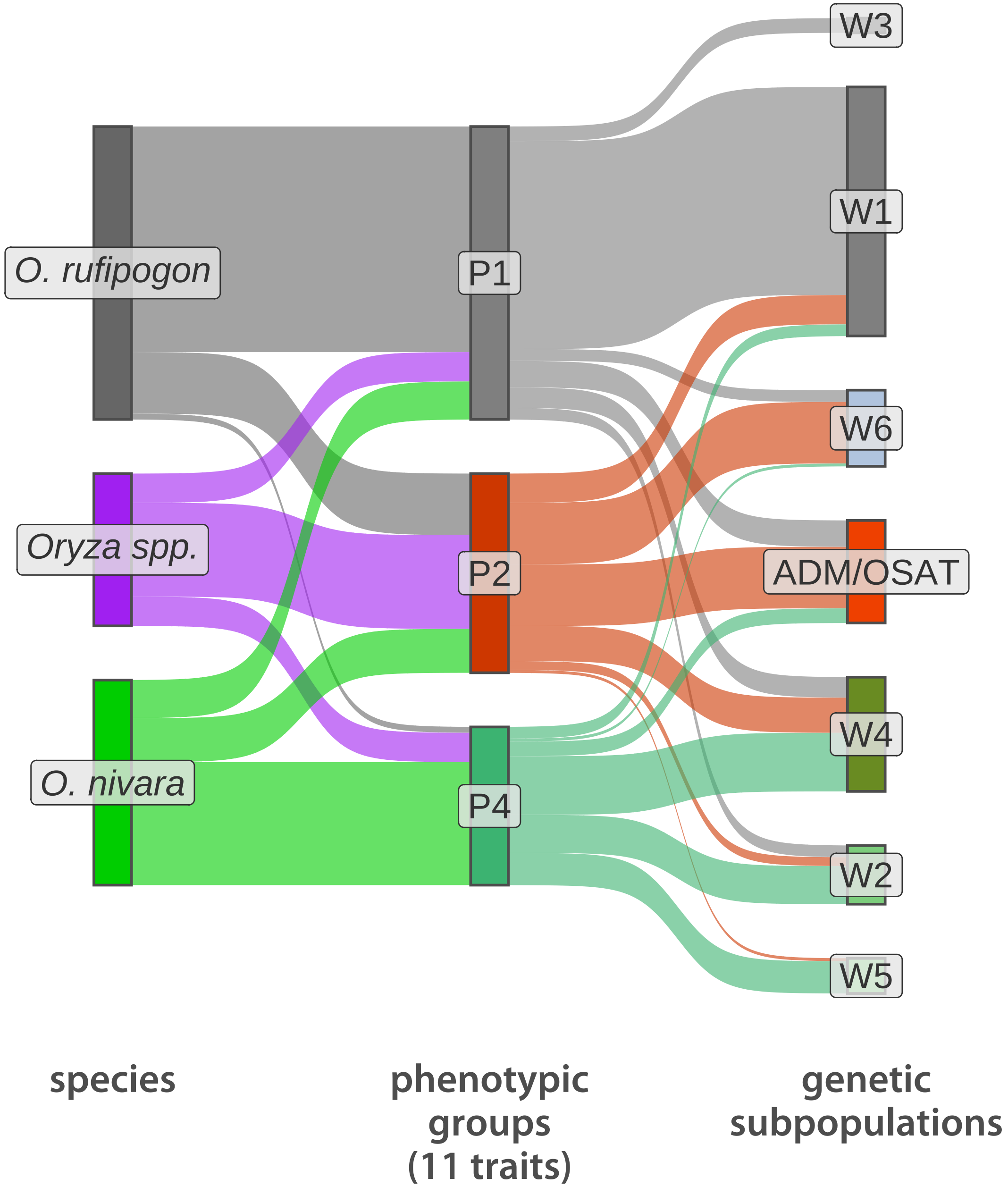
W3
W1
O. rufipogon
P1
W6
Oryza spp.
P2
ADM/OSAT
W4
O. nivara
P4
W2
W5
species
phenotypic groups (11 traits)
genetic subpopulations

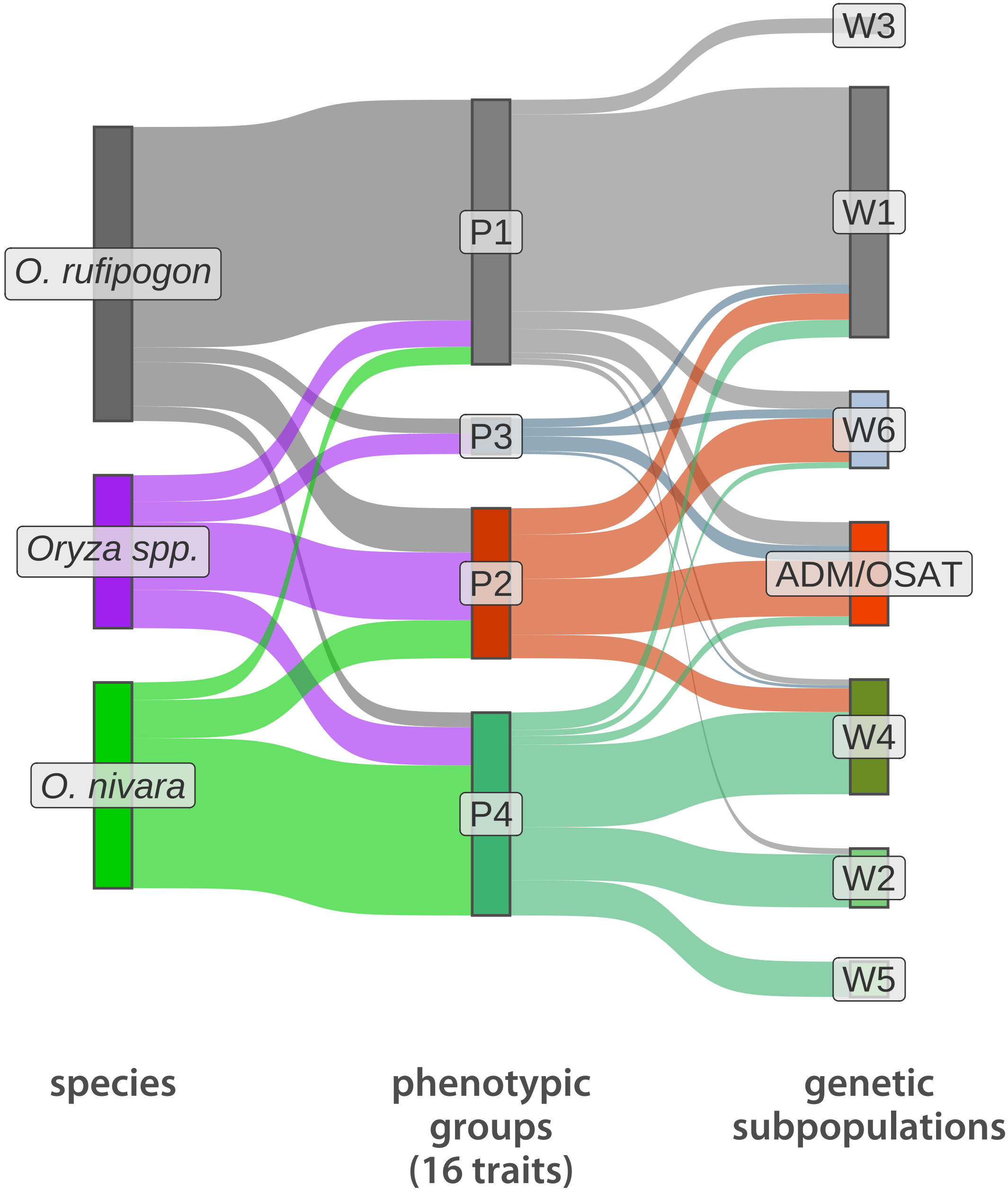
W3
P1
W1
O. rufipogon
P3
W6
Oryza spp.
P2
ADM/OSAT
W4
O. nivara
P4
W2
W5
species
phenotypic
groups
(16 traits)
genetic
subpopulations

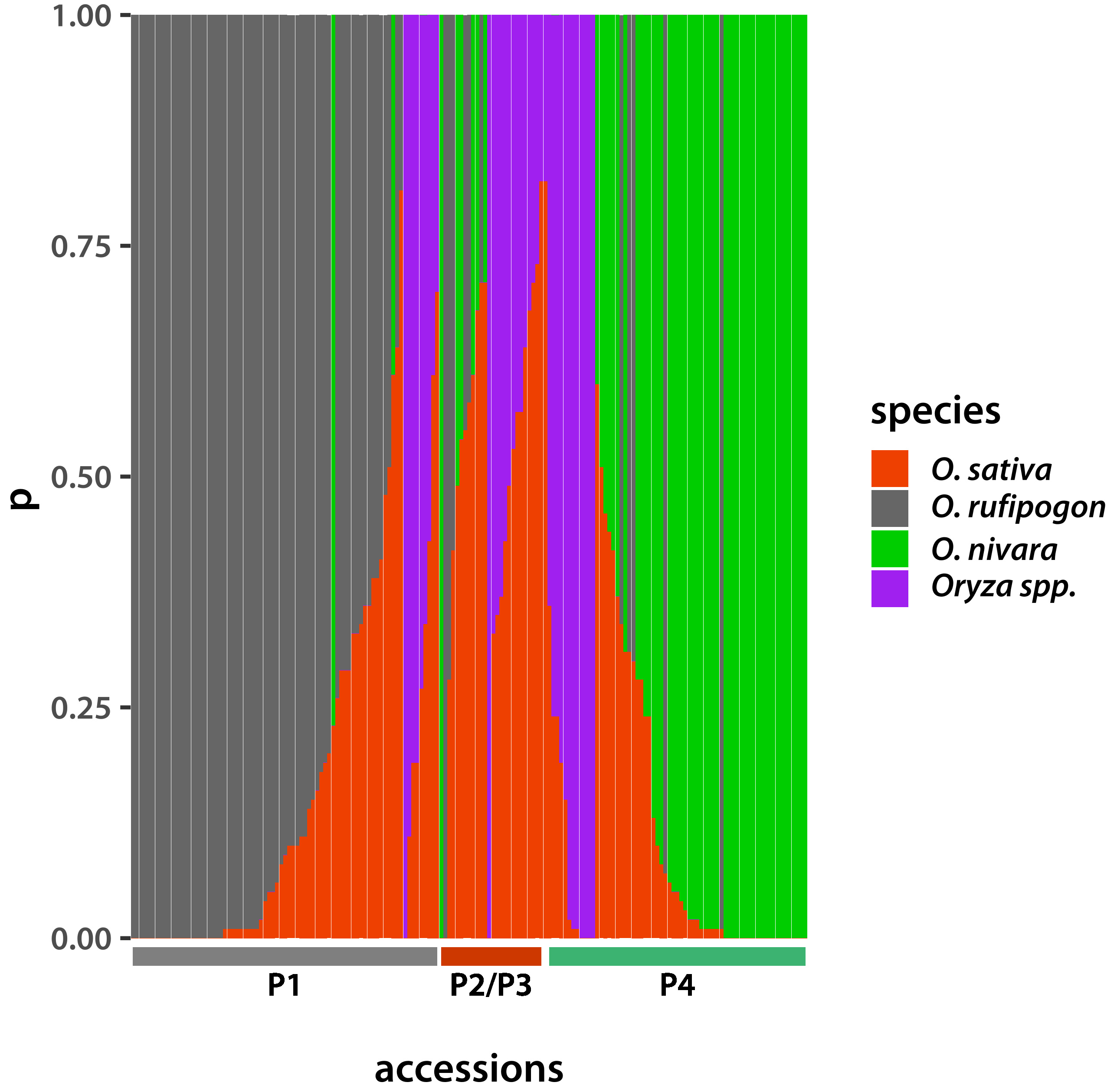

1.00
0.75
0.50
0.25
0.00
p
P1
P2/P3
P4
accessions
species
O. sativa
O. rufipogon
O. nivara
Oryza spp.

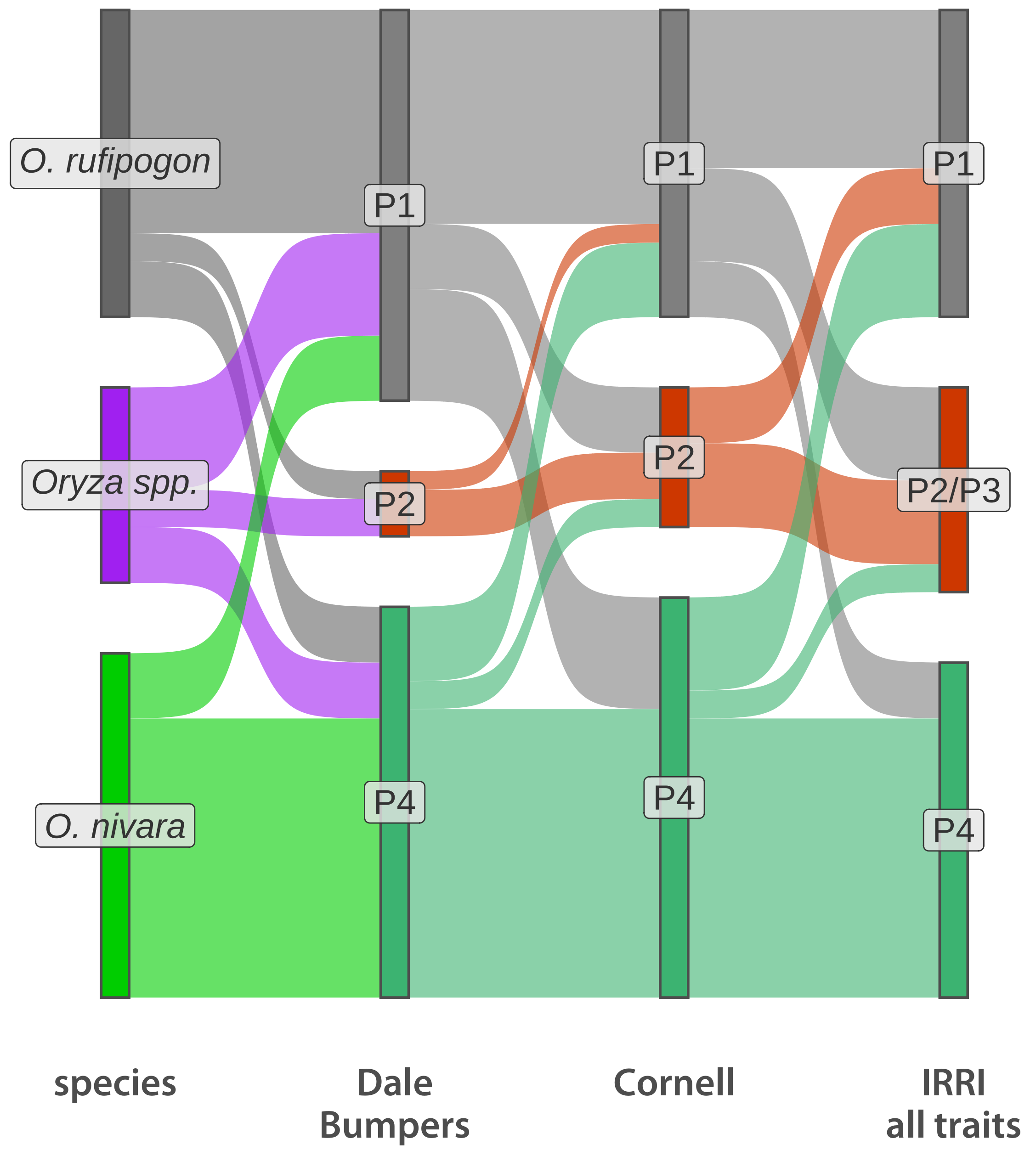
O. rufipogon
Oryza spp.
O. nivara
P1
P2
P4
P1
P2
P4
P1
P2/P3
P4
species
Dale Bumpers
Cornell
IRRI all traits

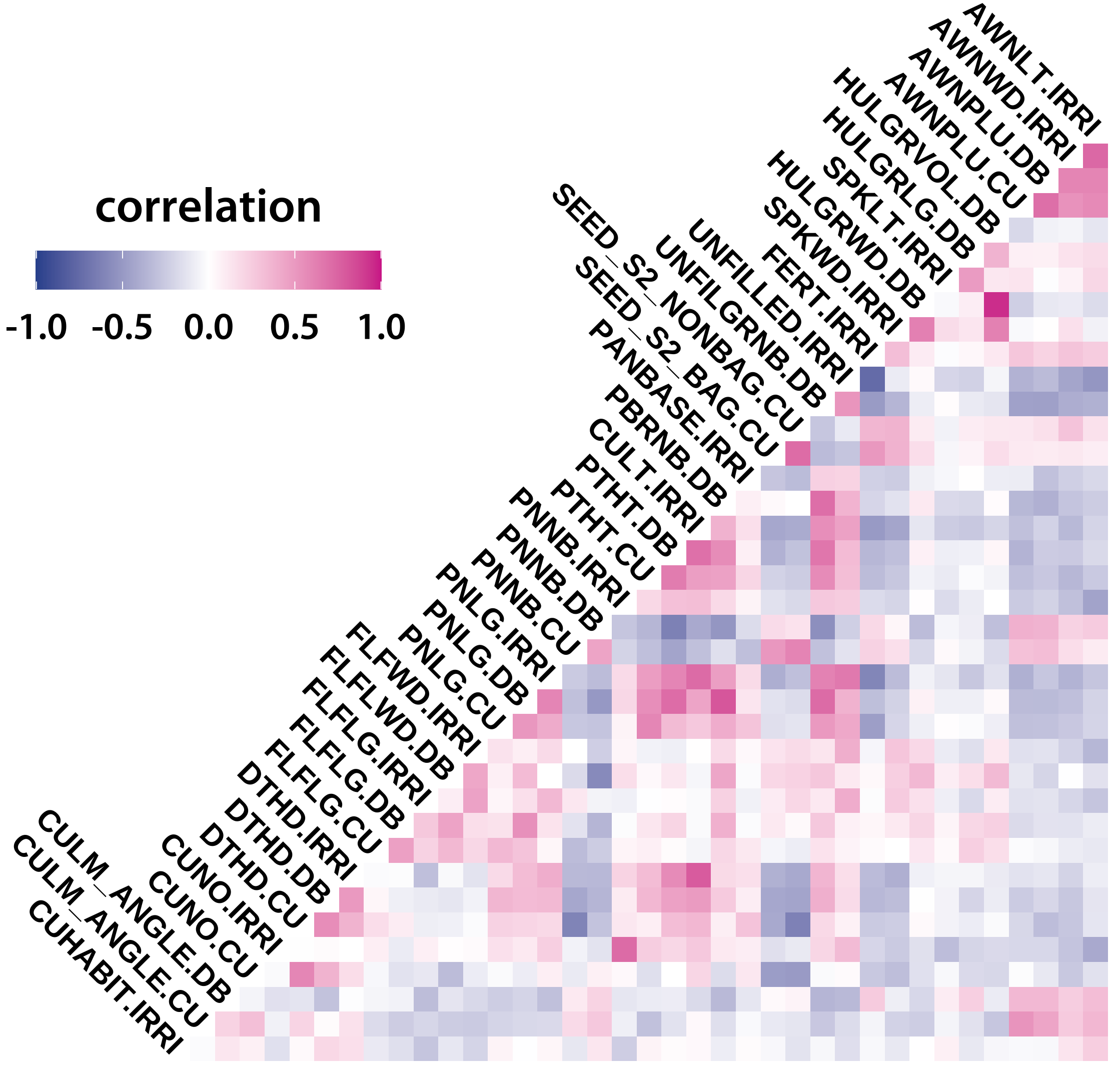
correlation
-1.0
-0.5
0.0
0.5
1.0
AWNLT.IRRI
AWNWD.IRRI
AWNPLU.IRRI
AWNPLU.DB
AWNPLU.CU
HULGRVOL.DB
HULGRLG.DB
SPKLT.IRRI
HULGRWD.DB
SPKWD.IRRI
FERT.IRRI
UNFILLED.IRRI
UNFILGRNB.DB
SEED_S2_NONBAG.CU
SEED_S2_BAG.CU
PANBASE.IRRI
PBRNB.DB
CULT.IRRI
PTHT.DB
PTHT.CU
PNNB.IRRI
PNNB.DB
PNNB.CU
PNLG.IRRI
PNLG.DB
PNLG.CU
FLFWD.IRRI
FLFLWD.DB
FLFLG.IRRI
FLFLG.DB
FLFLG.CU
DTHD.IRRI
DTHD.DB
DTHD.CU
CUNO.IRRI
CUNO.CU
CULM_ANGLE.DB
CULM_ANGLE.CU
CUHABIT.IRRI

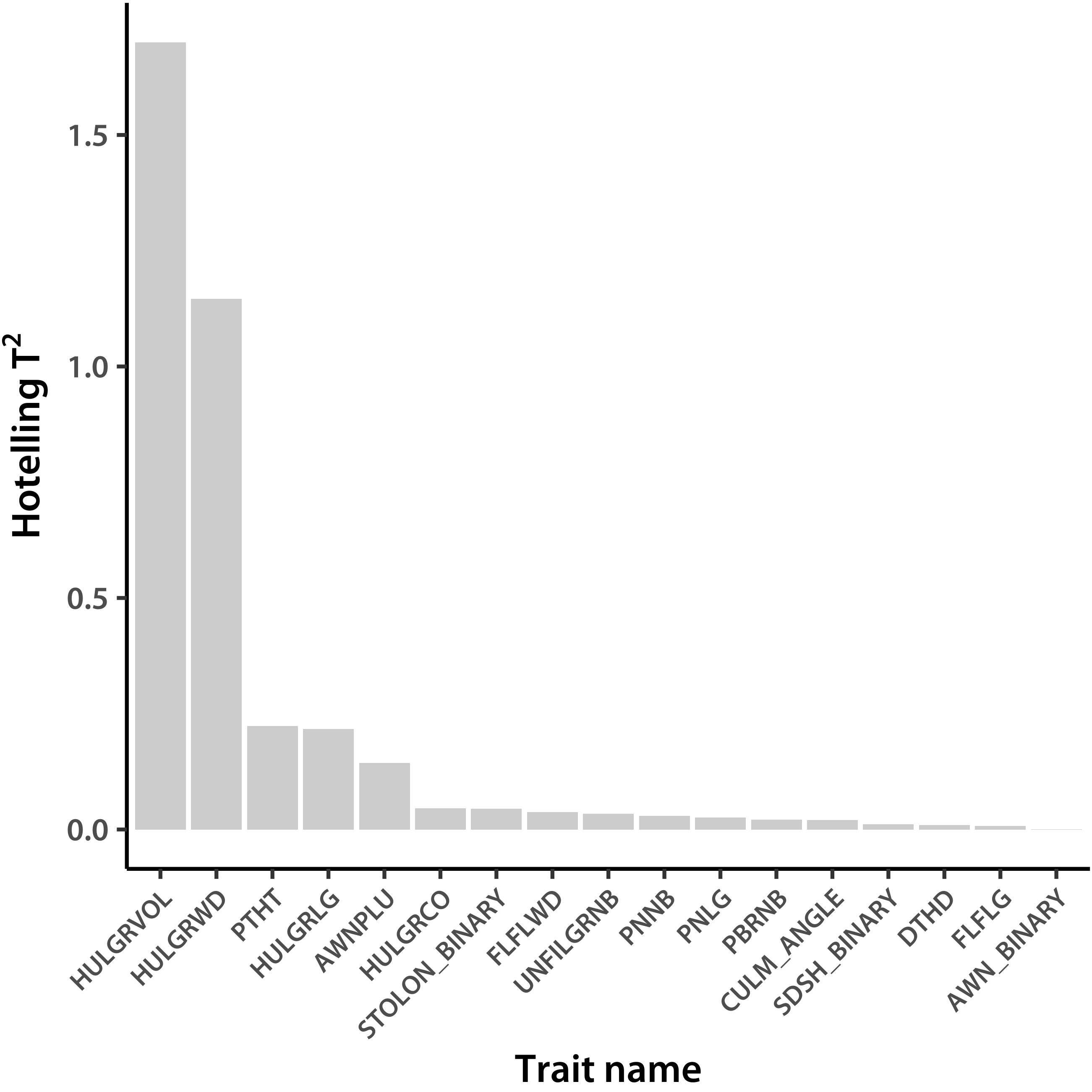

Hotelling T^2
1.5
1.0
0.5
0.0
HULGRVOL
HULGRWD
PTHT
HULGRLG
AWNPLU
HULGRCO
STOLON_BINARY
FLFLWD
UNFILGRNB
PNNB
PNLG
PBRNB
CULM_ANGLE
SDSH_BINARY
DTHD
FLFLG
AWN_BINARY
Trait name

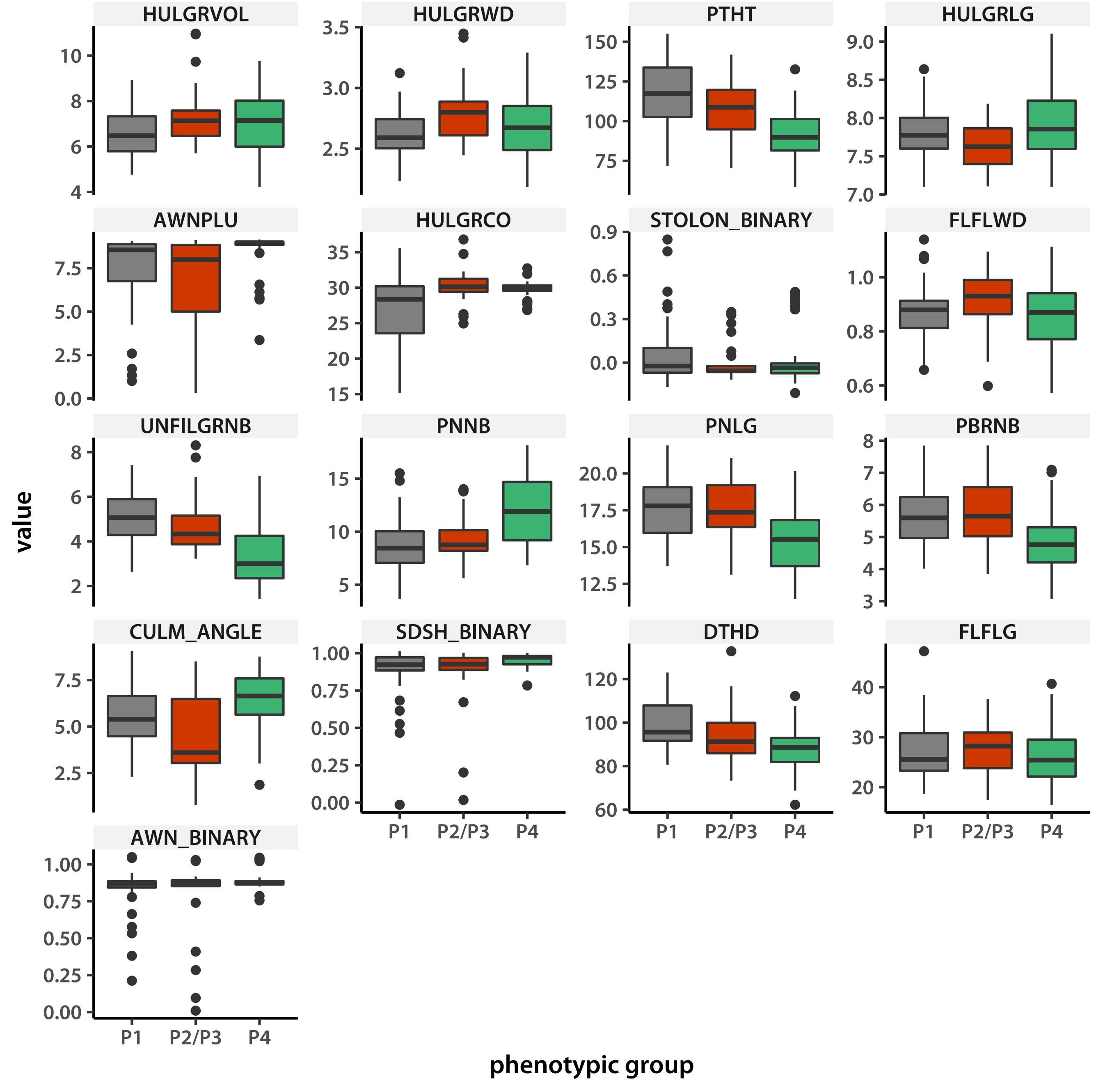

HULGRVOL
HULGRWD
PTHT
HULGRLG
AWNPLU
HULGRCO
STOLON_BINARY
FLFLWD
UNFILGRNB
PNNB
PNLG
PBRNB
CULM_ANGLE
SDSH_BINARY
DTHD
FLFLG
AWN_BINARY
value
P1
P2/P3
P4
phenotypic group

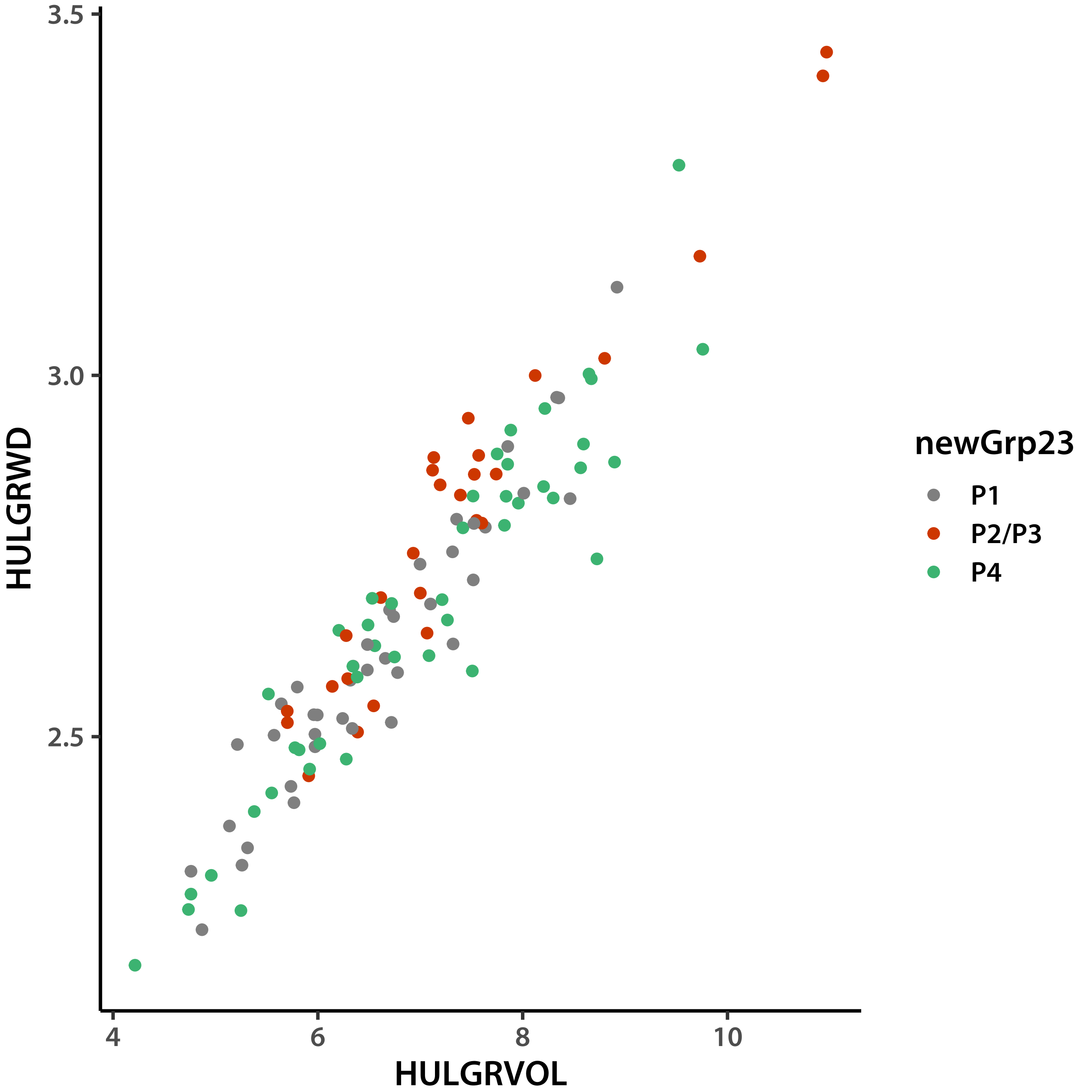

3.5
3.0
2.5
HULGRWD
4
6
8
10
HULGRVOL
newGrp23
P1
P2/P3
P4

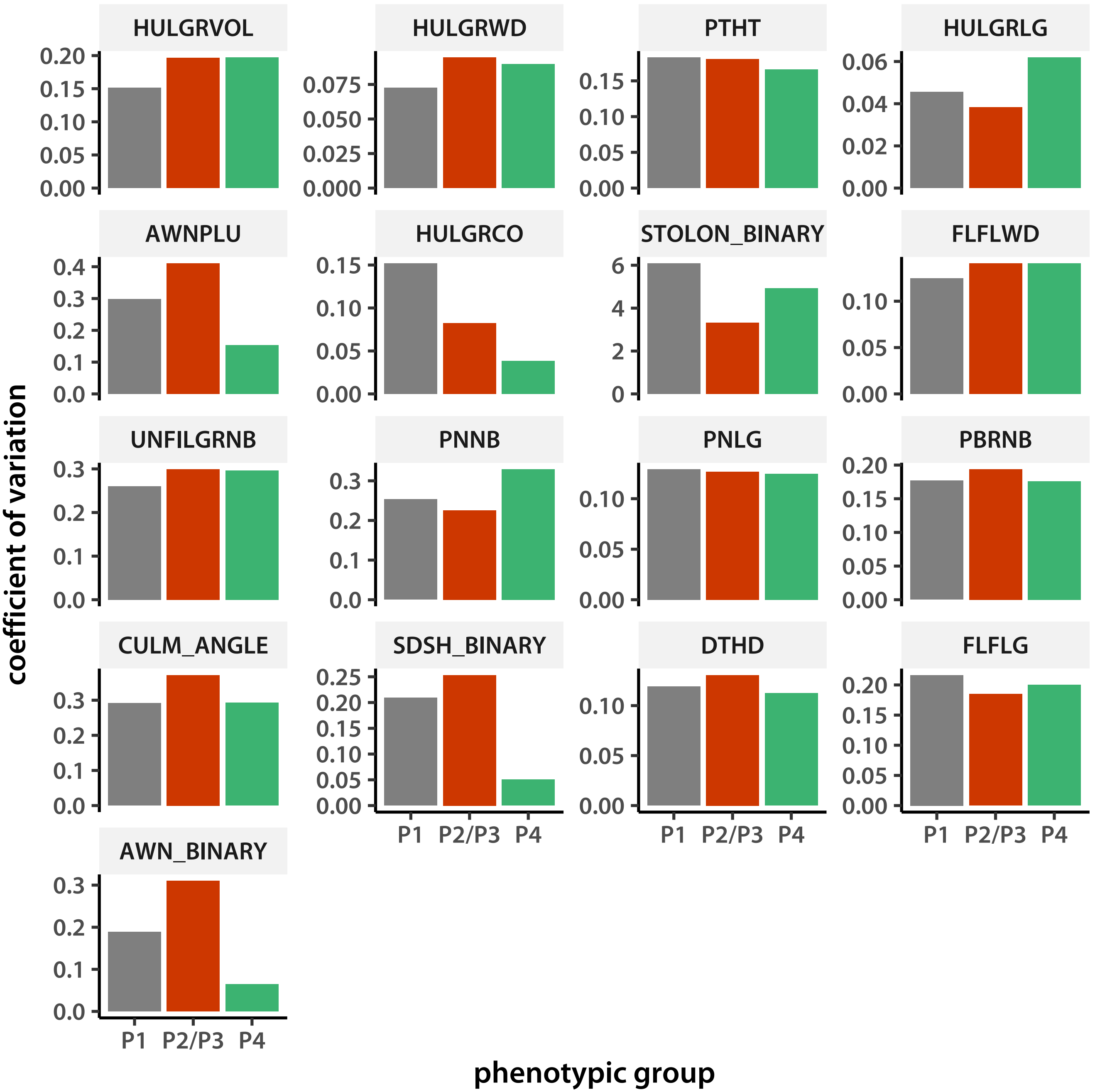

HULGRVOL
HULGRWD
PTHT
HULGRLG
AWNPLU
HULGRCO
STOLON_BINARY
FLFLWD
UNFILGRNB
PNNB
PNLG
PBRNB
CULM_ANGLE
SDSH_BINARY
DTHD
FLFLG
AWN_BINARY
P1
P2/P3
P4
coefficient of variation
phenotypic group

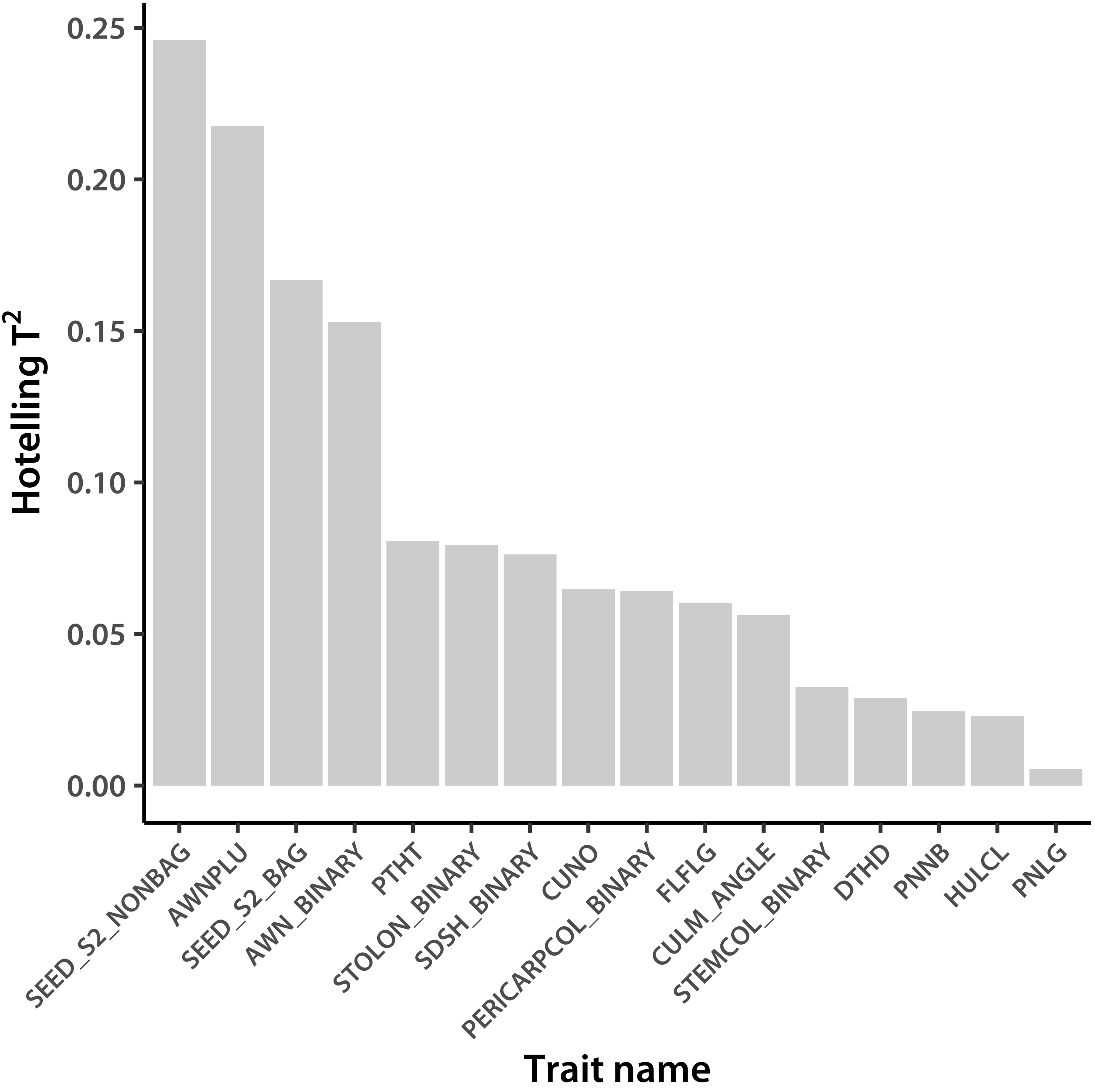

Hotelling T^2
0.25
0.20
0.15
0.10
0.05
0.00
SEED_S2_NONBAG
AWNPLU
SEED_S2_BAG
AWN_BINARY
PTHT
STOLON_BINARY
SDSH_BINARY
CUNO
PERICARPCOL_BINARY
FLFLG
CULM_ANGLE
STEMCOL_BINARY
DTHD
PNNB
HULCL
PNLG
Trait name

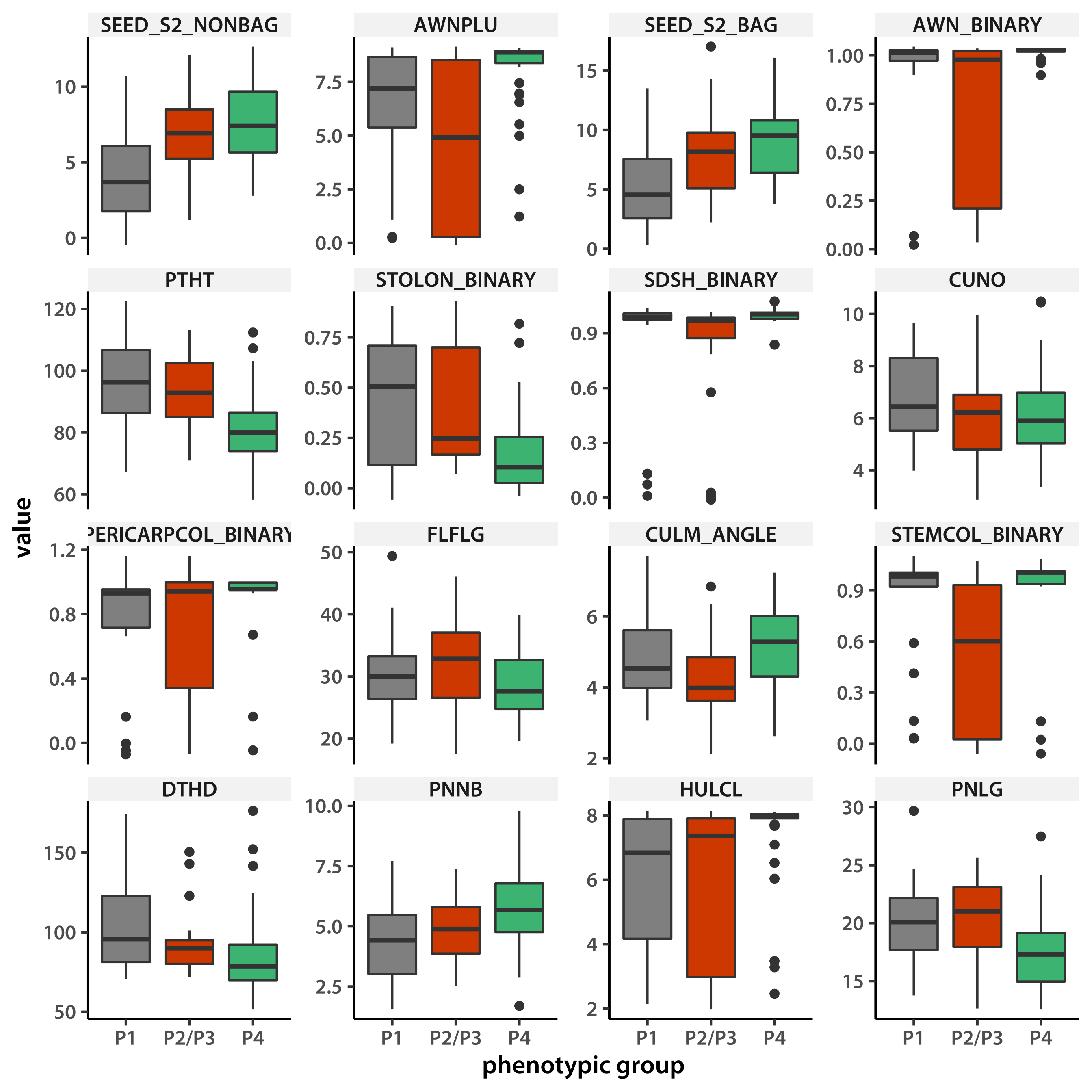

SEED_S2_NONBAG
AWNPLU
SEED_S2_BAG
AWN_BINARY
PTHT
STOLON_BINARY
SDSH_BINARY
CUNO
PERICARPCOL_BINARY
FLFLG
CULM_ANGLE
STEMCOL_BINARY
DTHD
PNNB
HULCL
PNLG
value
P1
P2/P3
P4
phenotypic group

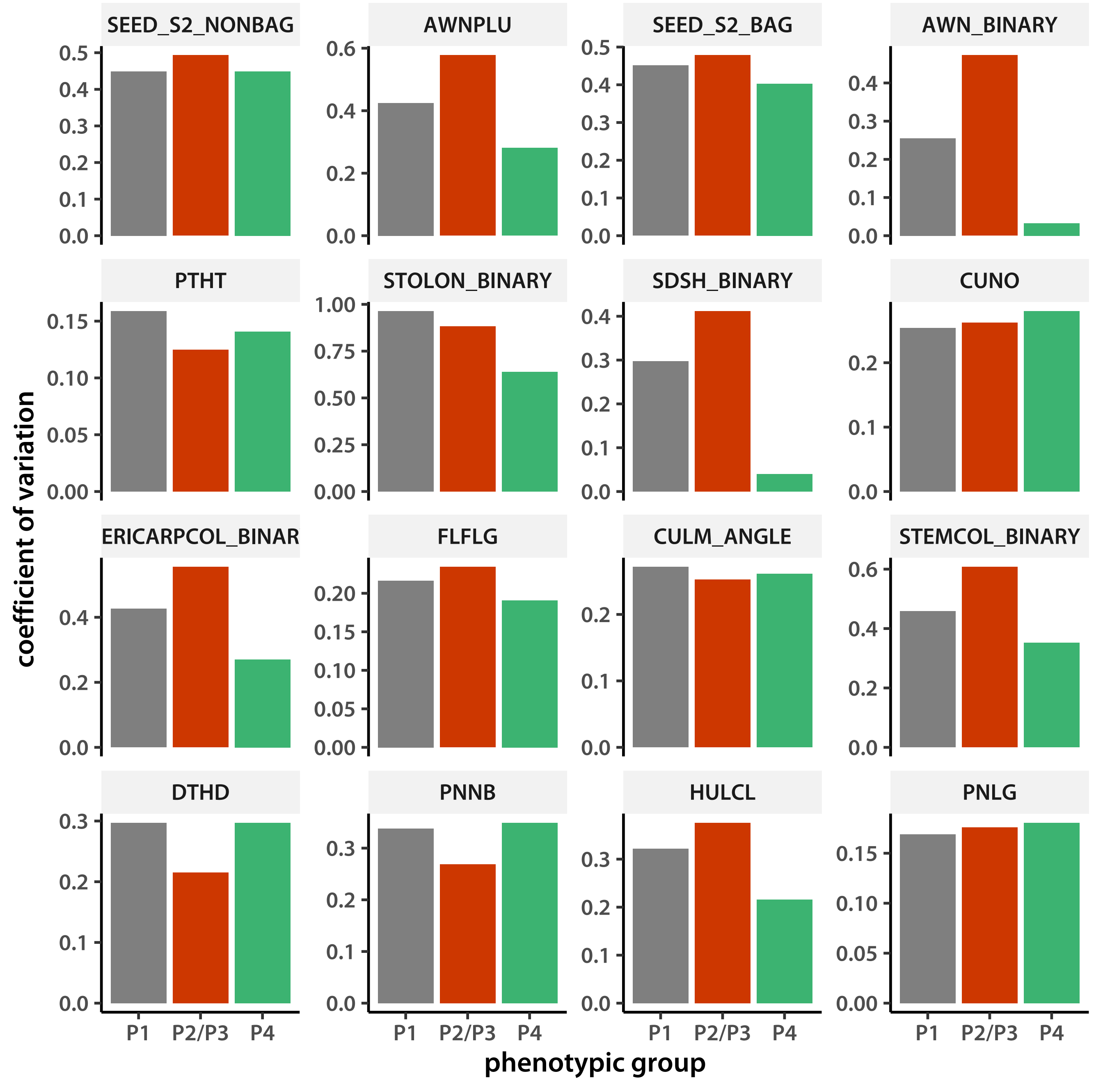

SEED_S2_NONBAG
AWNPLU
SEED_S2_BAG
AWN_BINARY
PTHT
STOLON_BINARY
SDSH_BINARY
CUNO
ERICARPCOL_BINAR
FLFLG
CULM_ANGLE
STEMCOL_BINARY
DTHD
PNNB
HULCL
PNLG
P1
P2/P3
P4
coefficient of variation
phenotypic group

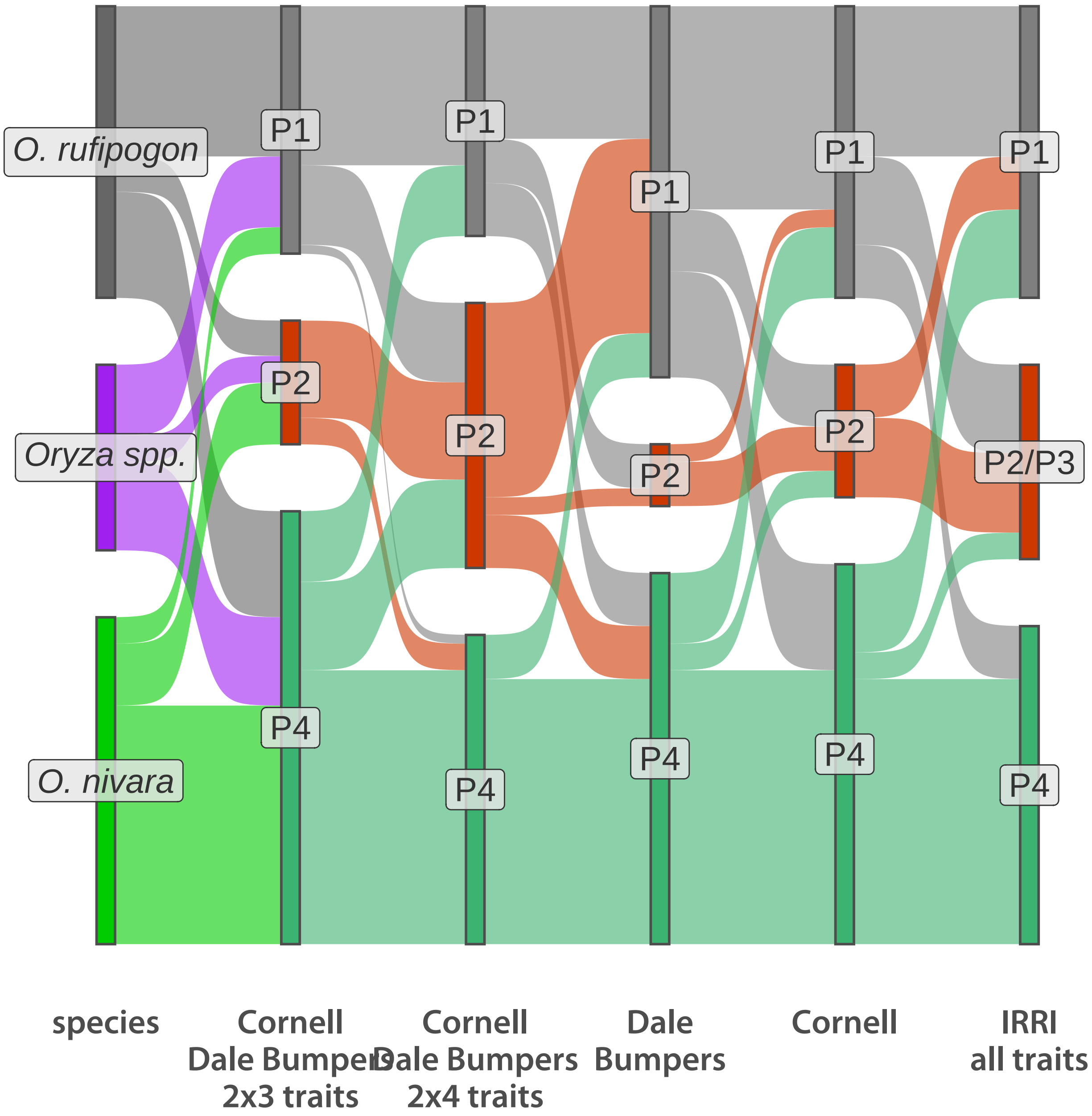
O. rufipogon
Oryza spp.
O. nivara
P1
P2
P4
P1
P2
P4
P1
P2
P4
P1
P2
P4
P1
P2/P3
P4
species
Cornell
Dale Bumpers
2x3 traits
Cornell
Dale Bumpers
2x4 traits
Dale
Bumpers
Cornell
IRRI
all traits

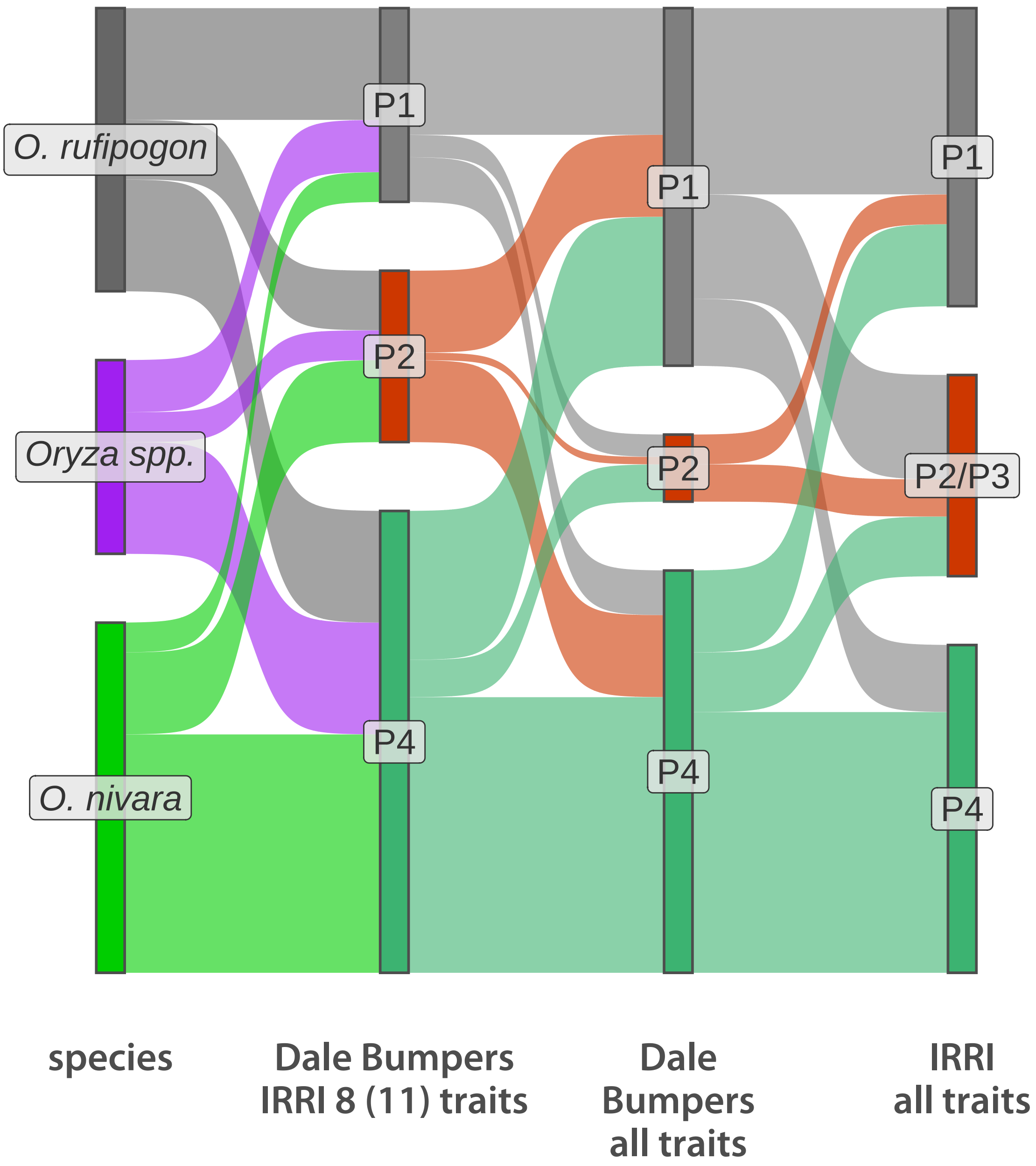

O. rufipogon
Oryza spp.
O. nivara
P1
P2
P4
P1
P2
P4
P1
P2/P3
P4
species
Dale Bumpers IRRI 8 (11) traits
Dale Bumpers all traits
IRRI all traits

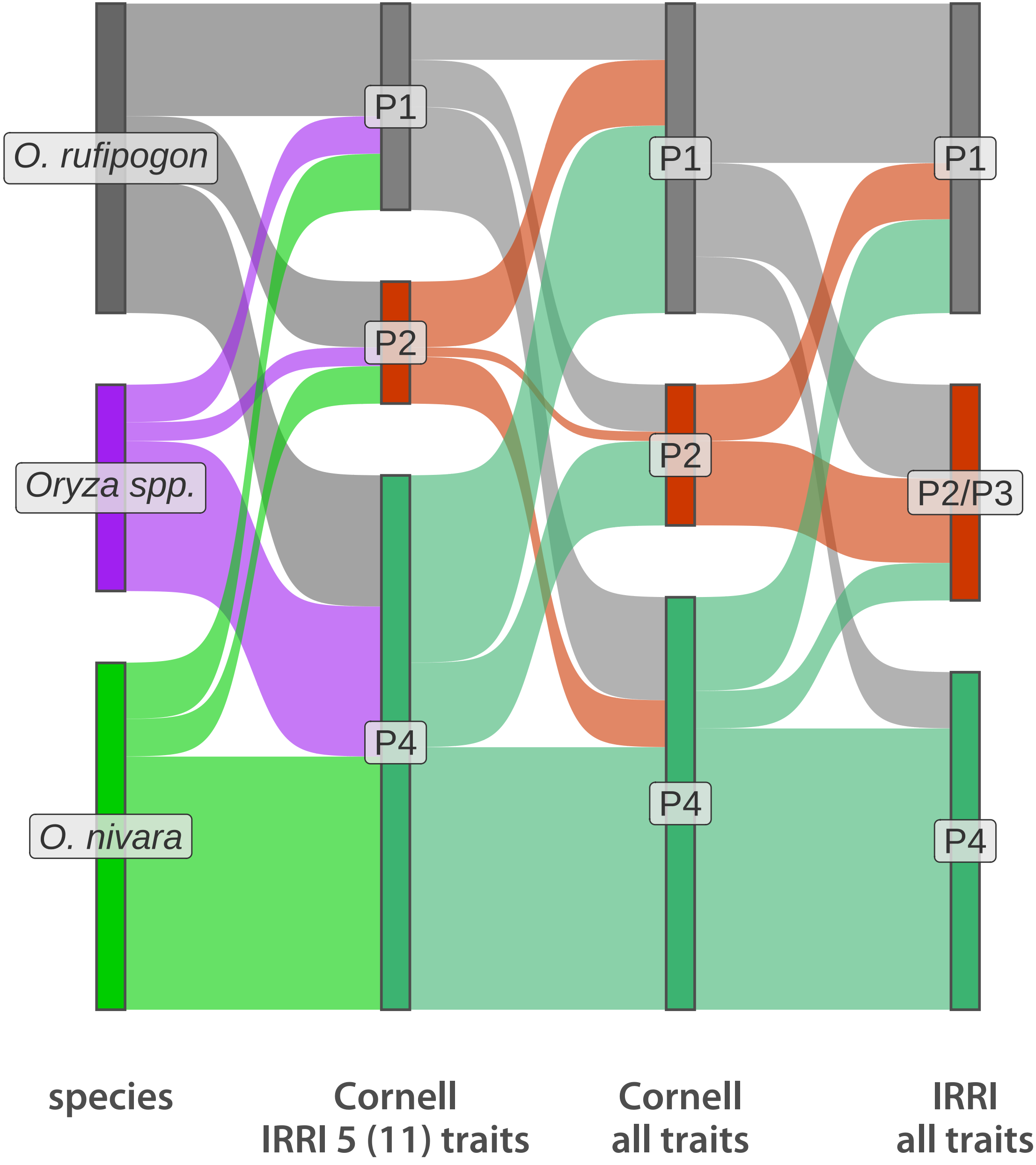
O. rufipogon
Oryza spp.
O. nivara
P1
P2
P4
P1
P2
P4
P1
P2/P3
P4
species
Cornell
IRRI 5 (11) traits
Cornell
all traits
IRRI
all traits

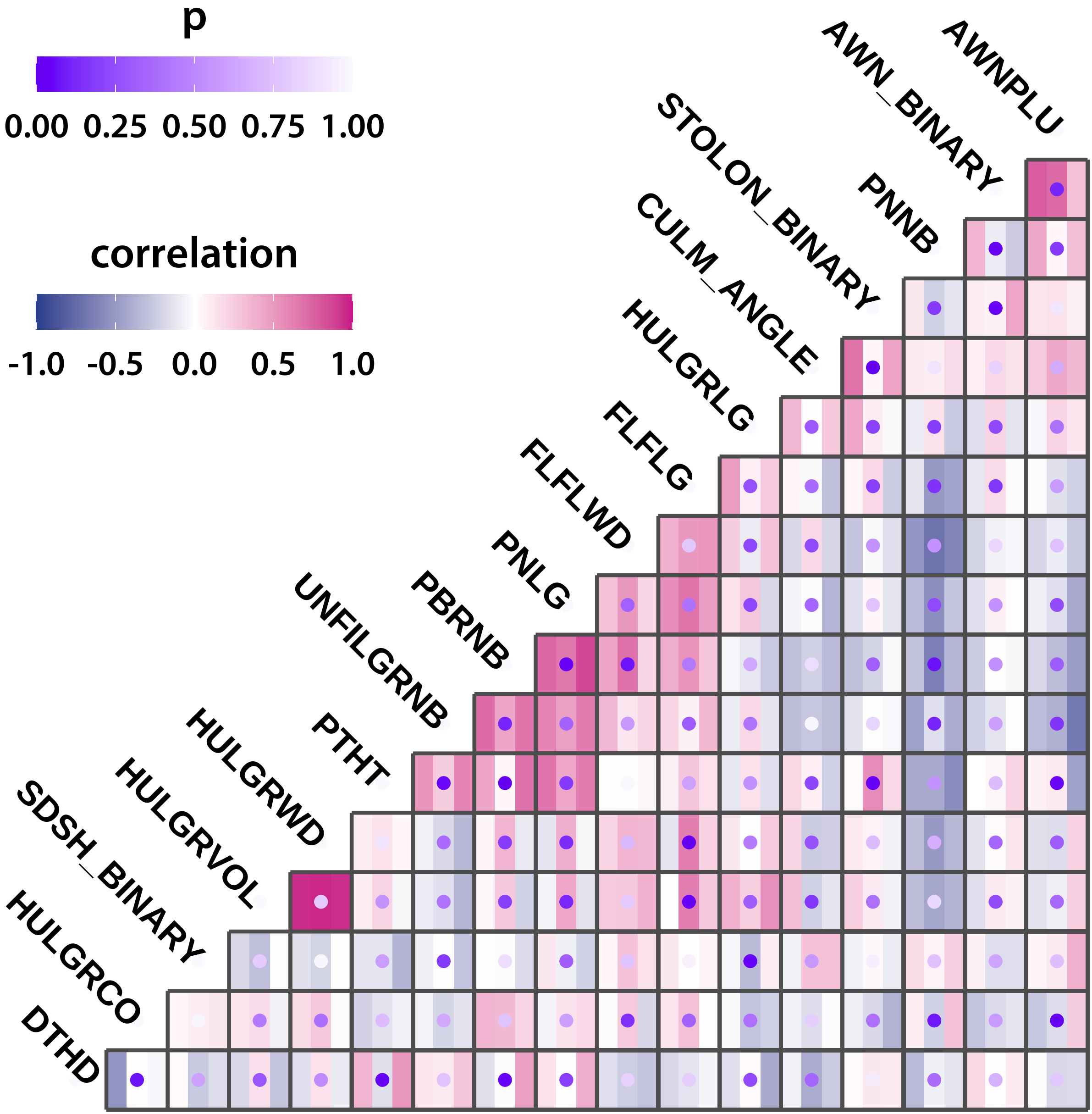

p
0.00 0.25 0.50 0.75 1.00
correlation
-1.0 -0.5 0.0 0.5 1.0
AWNPLU
AWN_BINARY
PNNB
STOLON_BINARY
CULM_ANGLE
HULGRLG
FLFLG
FLFLWD
PNLG
PBRNB
UNFILGRNB
PTHT
HULGRWD
HULGRVOL
SDSH_BINARY
HULGRCO
DTHD

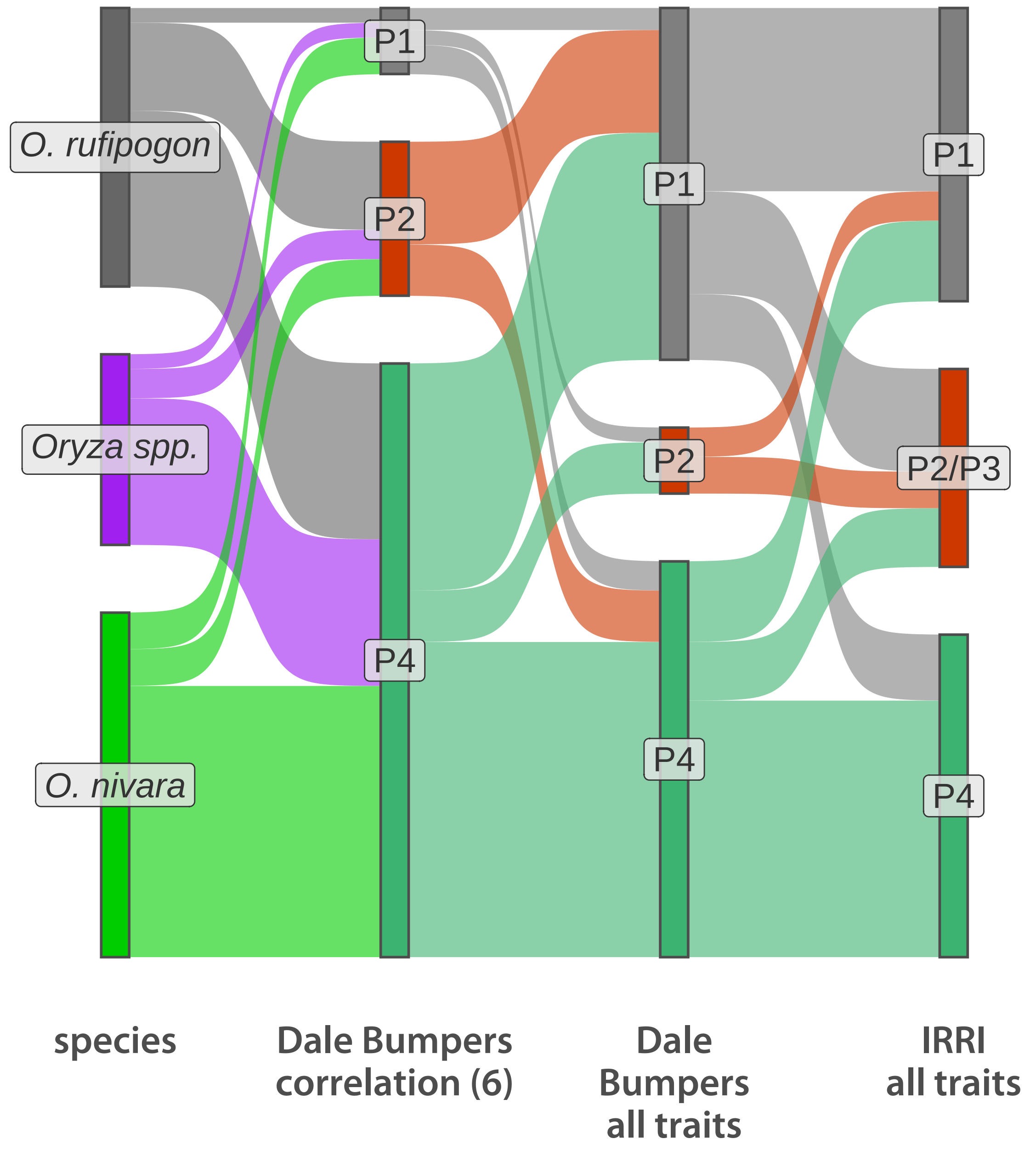
O. rufipogon
Oryza spp.
O. nivara
P1
P2
P4
P1
P2
P4
P1
P2/P3
P4
species
Dale Bumpers correlation (6)
Dale Bumpers all traits
IRRI all traits

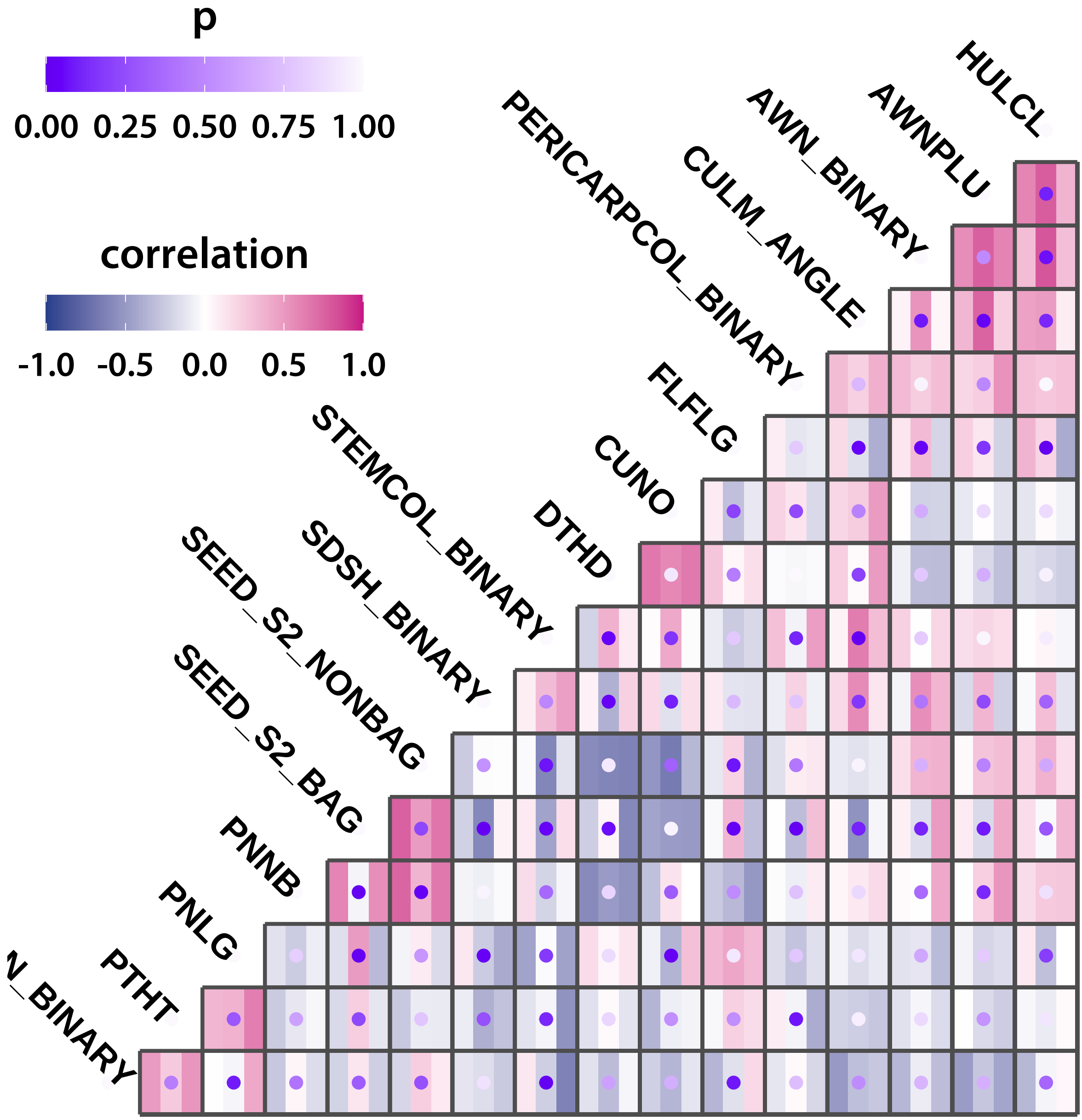
p
0.00
0.25
0.50
0.75
1.00
correlation
-1.0
-0.5
0.0
0.5
1.0
HULCL
AWNPLU
AWN_BINARY
CULM_ANGLE
PERICARPCOL_BINARY
FLFLG
CUNO
DTHD
STEMCOL_BINARY
SDSH_BINARY
SEED_S2_NONBAG
SEED_S2_BAG
PNNB
PNLG
PTHT
N_BINARY

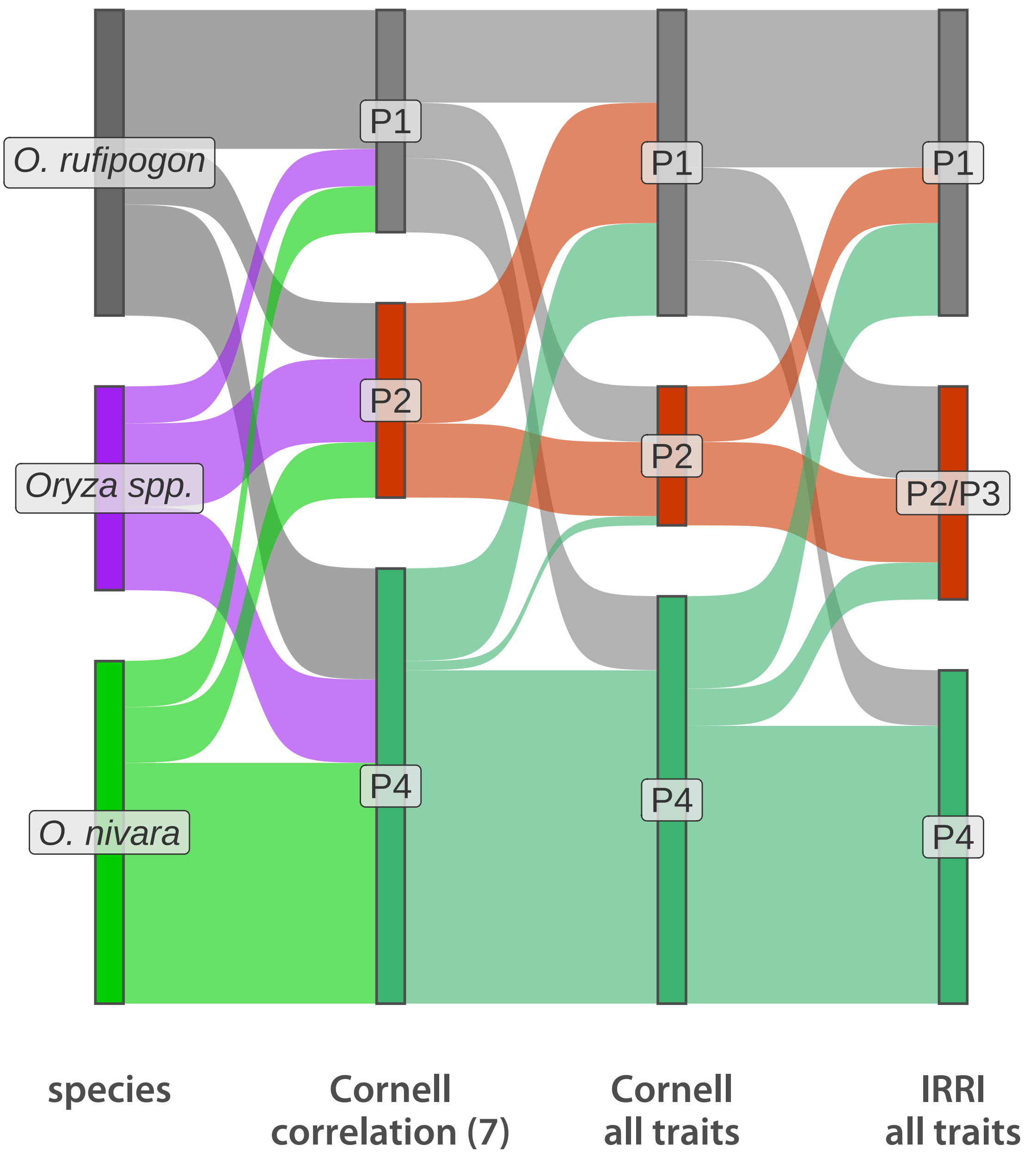
O. rufipogon
Oryza spp.
O. nivara
P1
P2
P4
P1
P2
P4
P1
P2/P3
P4
species
Cornell correlation (7)
Cornell all traits
IRRI all traits
