# Supplementary material for: Phenotypic Variation and the Impact of Admixture in the Oryza rufipogon Species Complex (ORSC)
Source: Front Plant Sci. 2022 Jun 13;13:787703. doi: 10.3389/fpls.2022.787703 (PMC9235872; doi:10.3389/fpls.2022.787703)
Supplement: Supplementary file 8 [file Data_Sheet_1.ZIP › sankeyPGrpDBCUsubset.pdf]

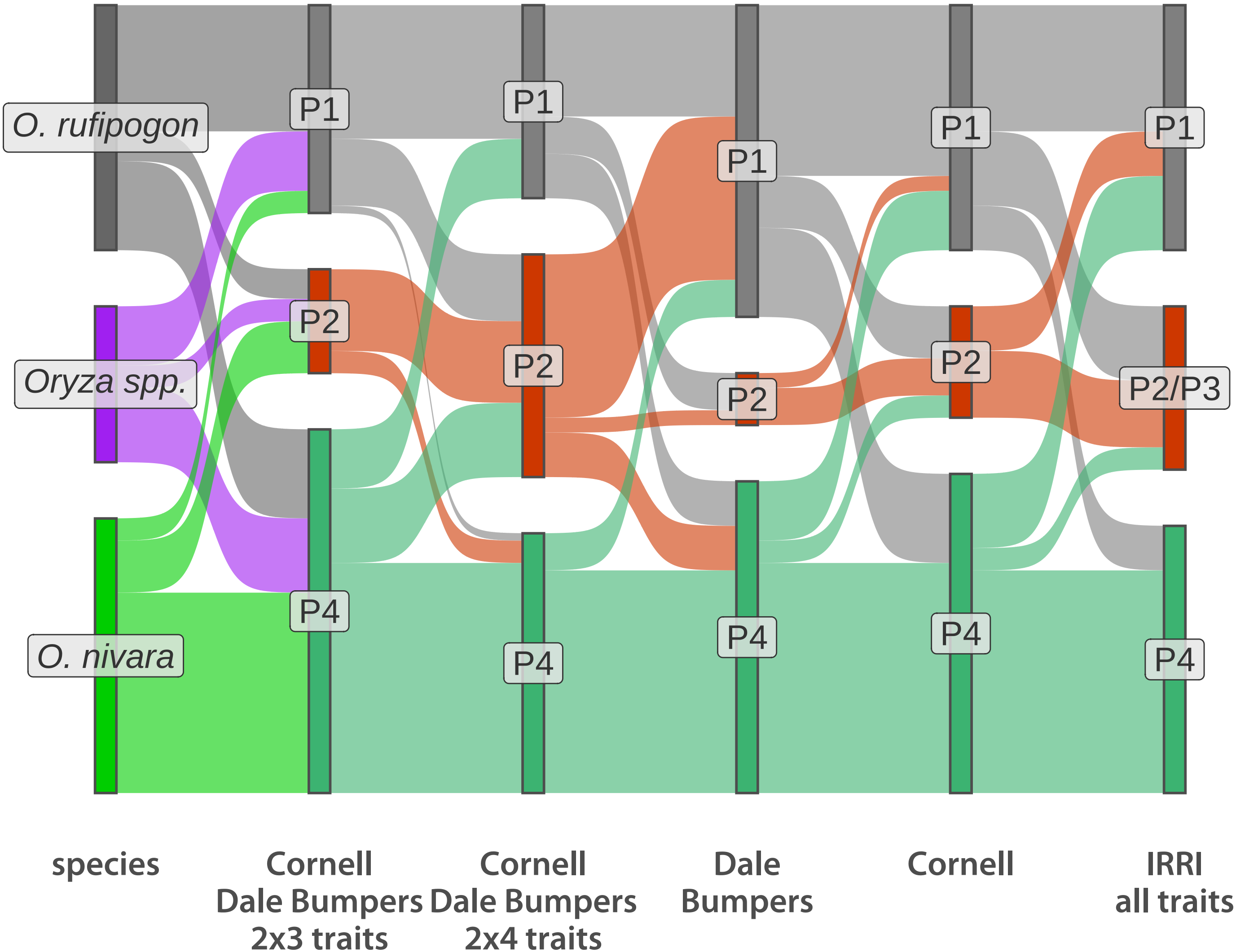
O. rufipogon
Oryza spp.
O. nivara
P1
P2
P4
P1
P2
P4
P1
P2
P4
P1
P2
P4
P1
P2/P3
P4
species
Cornell
Dale Bumpers
2x3 traits
Cornell
Dale Bumpers
2x4 traits
Dale
Bumpers
Cornell
IRRI
all traits
