# Supplementary figures and images for: Phenotypic Variation and the Impact of Admixture in the Oryza rufipogon Species Complex (ORSC)
Source: Front Plant Sci. 2022 Jun 13;13:787703. doi: 10.3389/fpls.2022.787703 (PMC9235872; doi:10.3389/fpls.2022.787703)

correlation

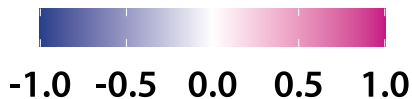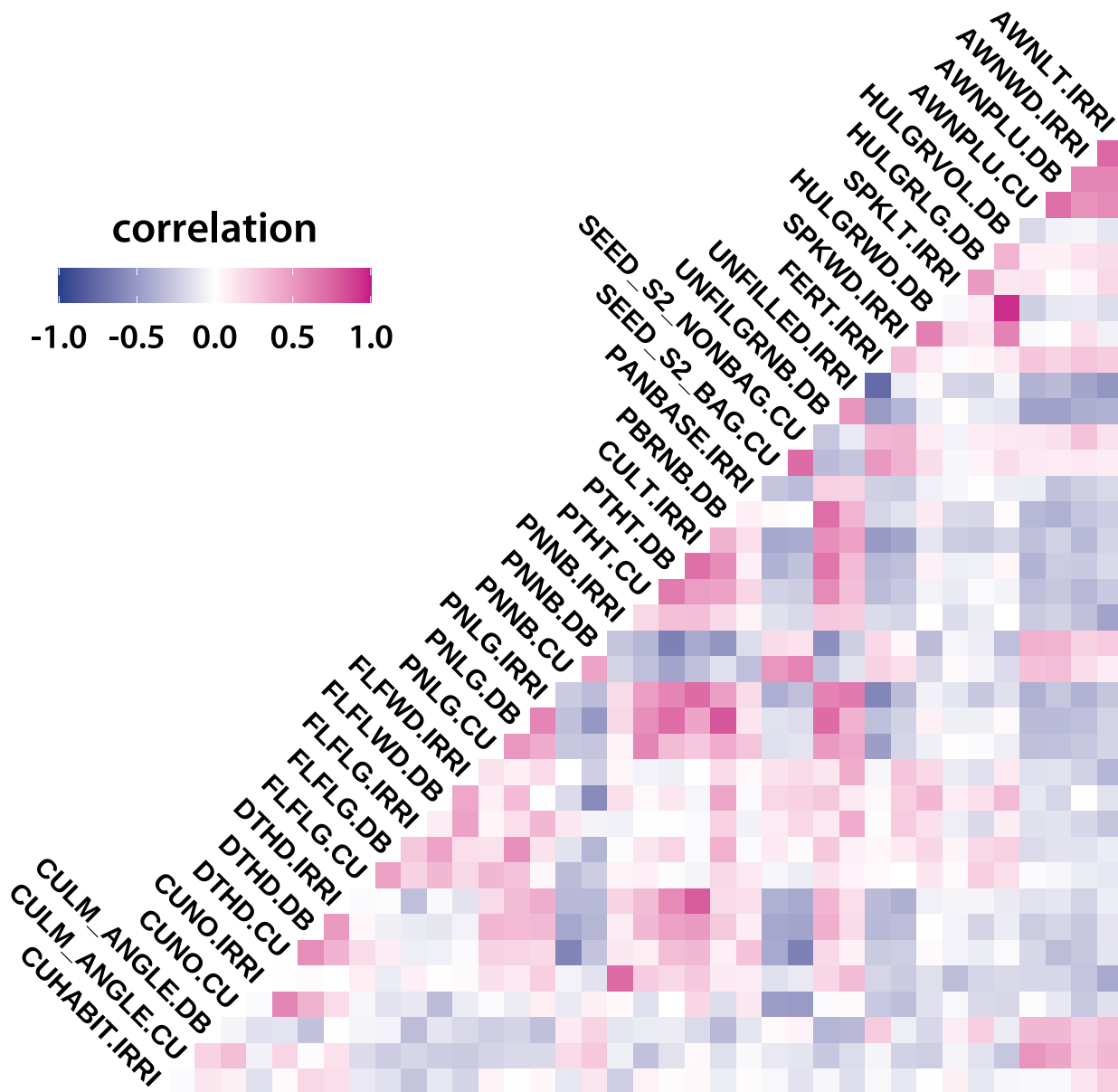

Supplement: Supplementary file 8 [file Data_Sheet_1.ZIP › commonTraitCorALL.pdf]

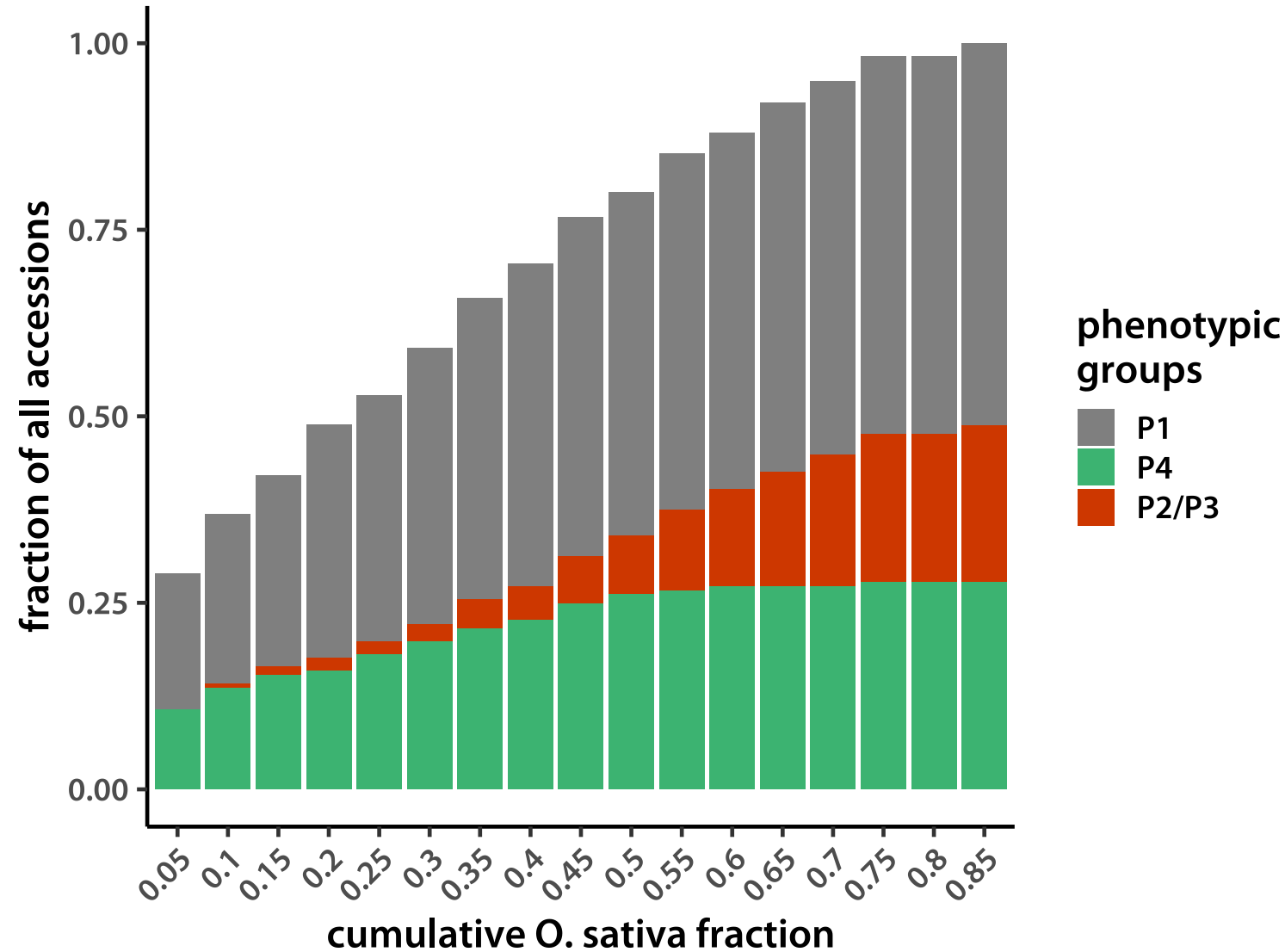

Supplement: Supplementary file 8 [file Data_Sheet_1.ZIP › cumOsatFrac.pdf]

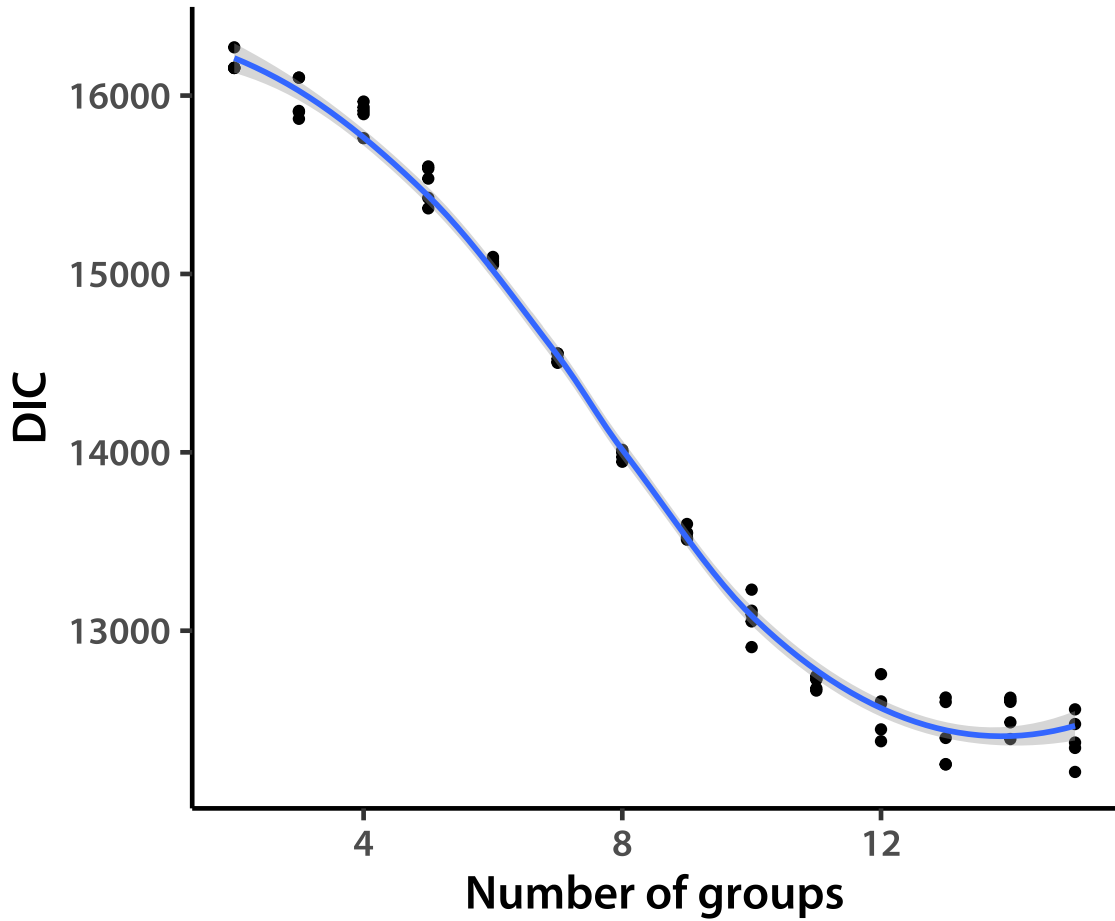

Supplement: Supplementary file 8 [file Data_Sheet_1.ZIP › dicIRRI.pdf]

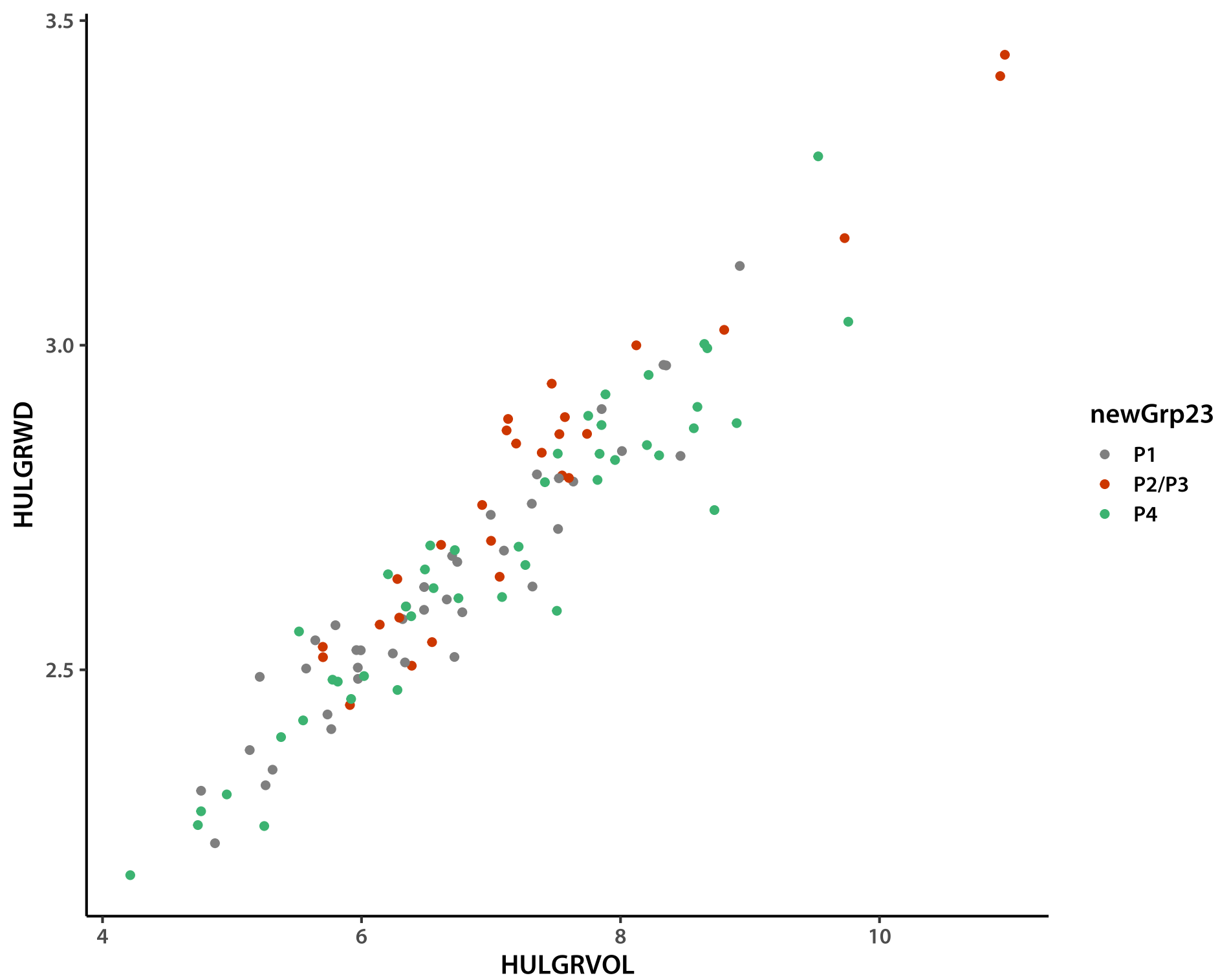

Supplement: Supplementary file 8 [file Data_Sheet_1.ZIP › grainsDB.pdf]

Number with > 2 lines

10

5

4

8

12

Number of groups

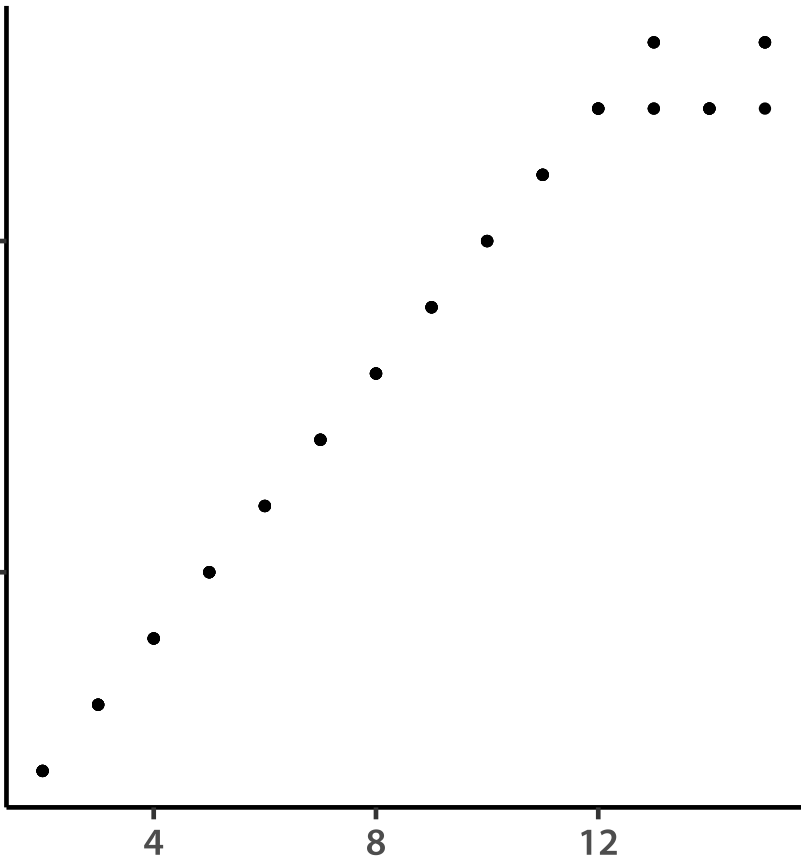

Supplement: Supplementary file 8 [file Data_Sheet_1.ZIP › gt2GrpIRRI.pdf]

phenotypic group

●

P1

●

P4

●

P2/P3

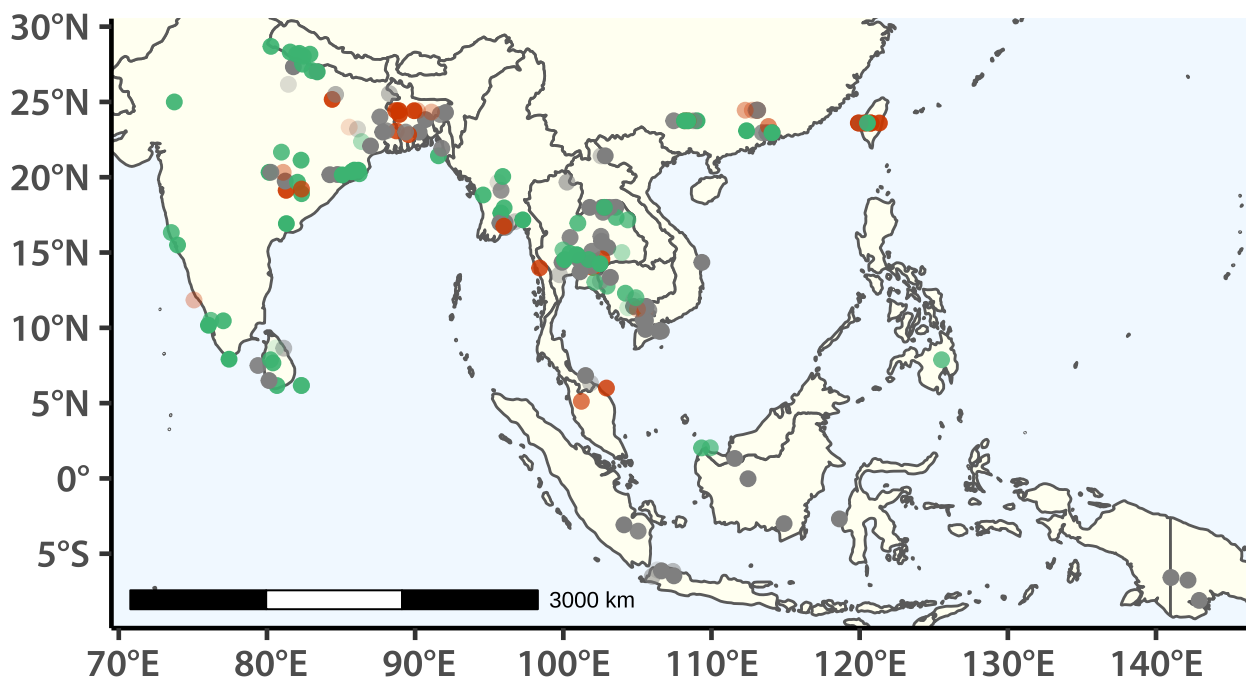

Supplement: Supplementary file 8 [file Data_Sheet_1.ZIP › mapIRRIp4.pdf]

species    ● *O. rufipogon*    ● *O. nivara*

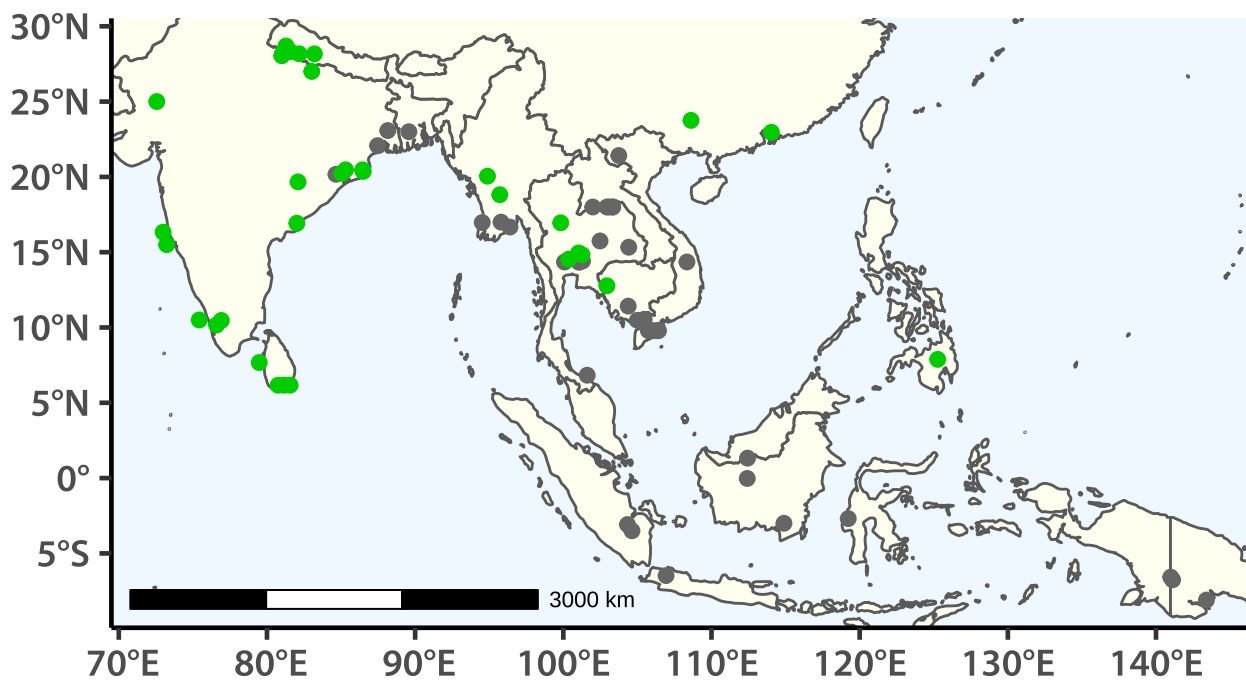

Supplement: Supplementary file 8 [file Data_Sheet_1.ZIP › mapPureSpp.pdf]

subpopulation

|    |    |    |
|----|----|----|
| W1 | W3 | W5 |
| W2 | W4 | W6 |

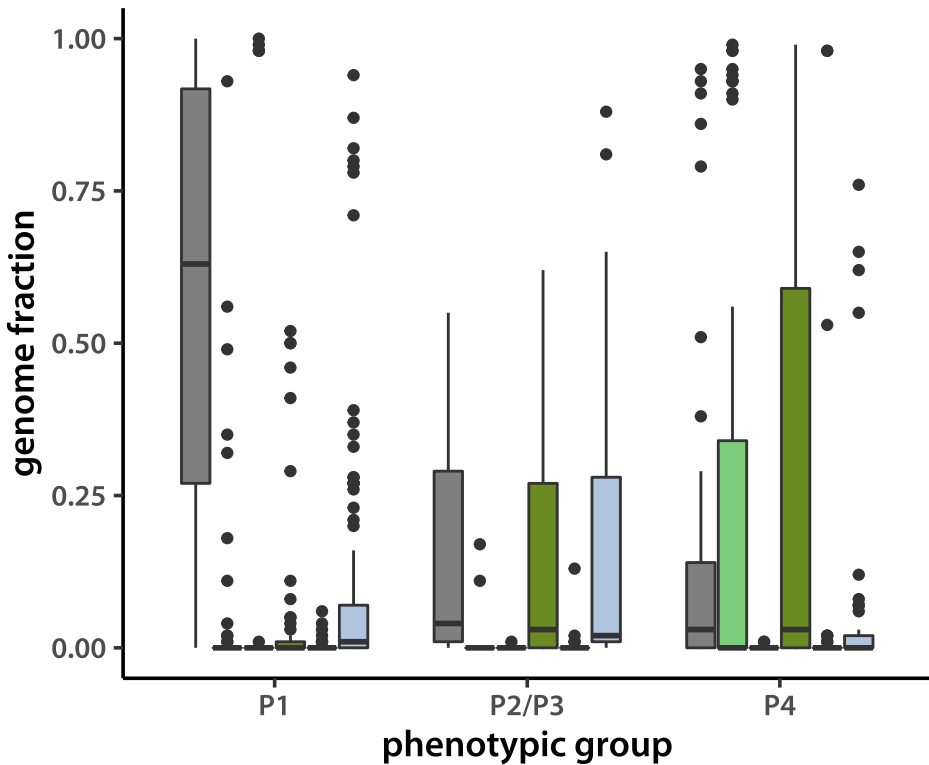

Supplement: Supplementary file 8 [file Data_Sheet_1.ZIP › rfmixRufiGrp4.pdf]

subpopulation

|     |     |     |
|-----|-----|-----|
| ARO | IND | TEJ |
| AUS | TRJ |     |

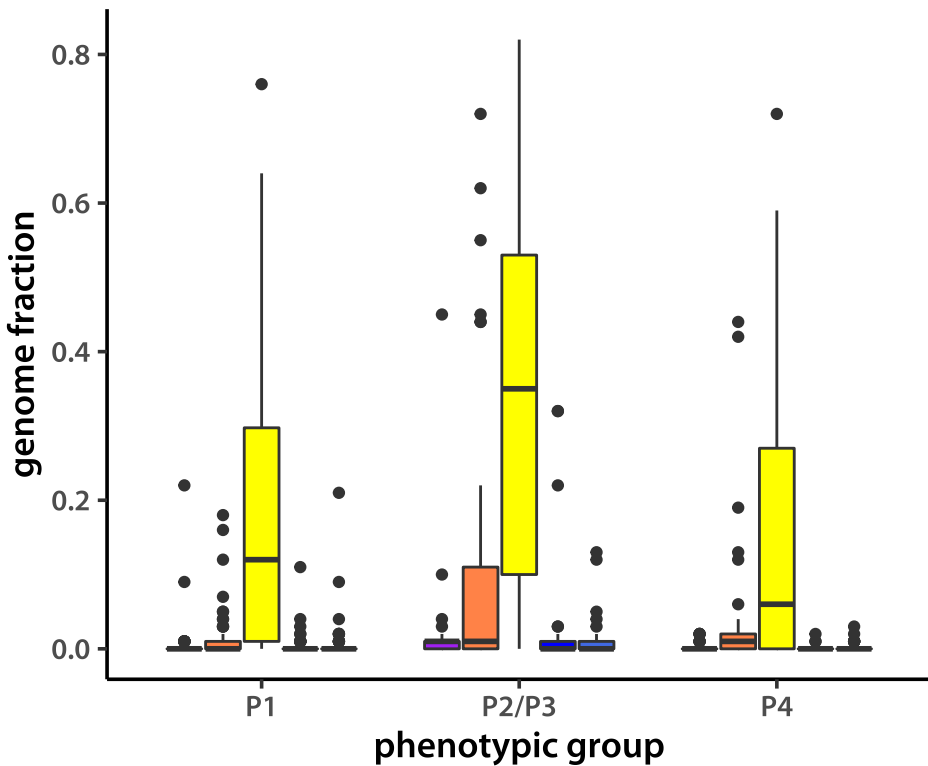

Supplement: Supplementary file 8 [file Data_Sheet_1.ZIP › rfmixSativaGrp4.pdf]

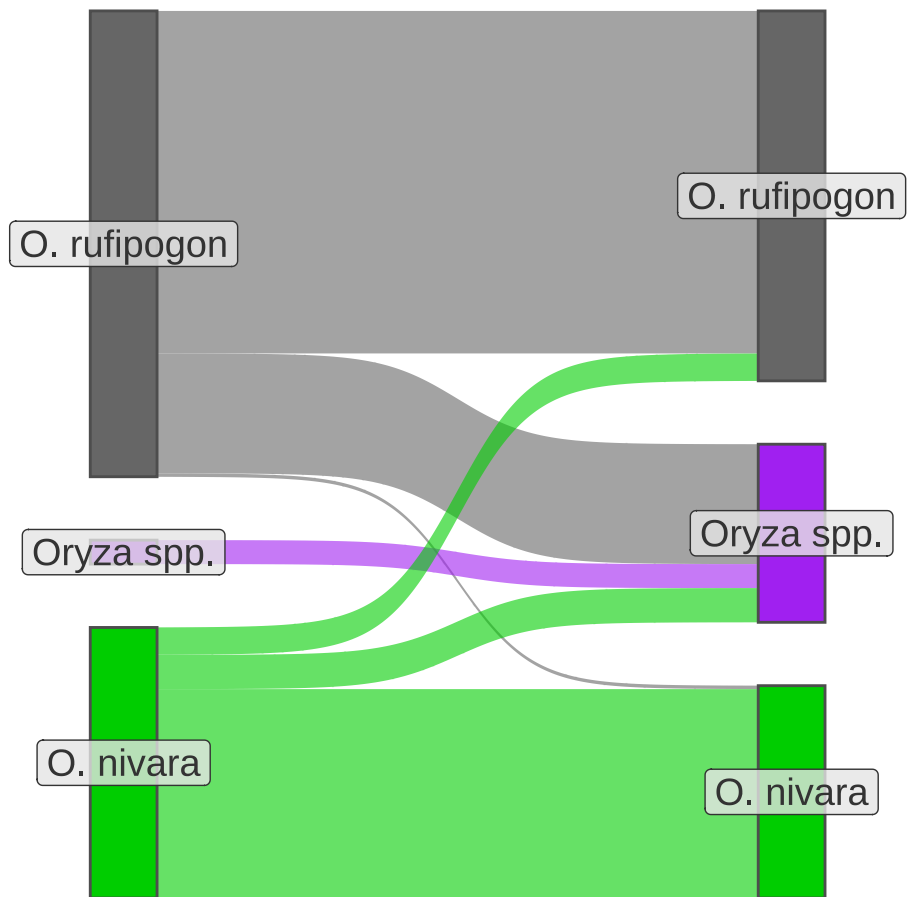

old species

GRIN species

designation

Supplement: Supplementary file 8 [file Data_Sheet_1.ZIP › sankeyGRINsppCompare.pdf]

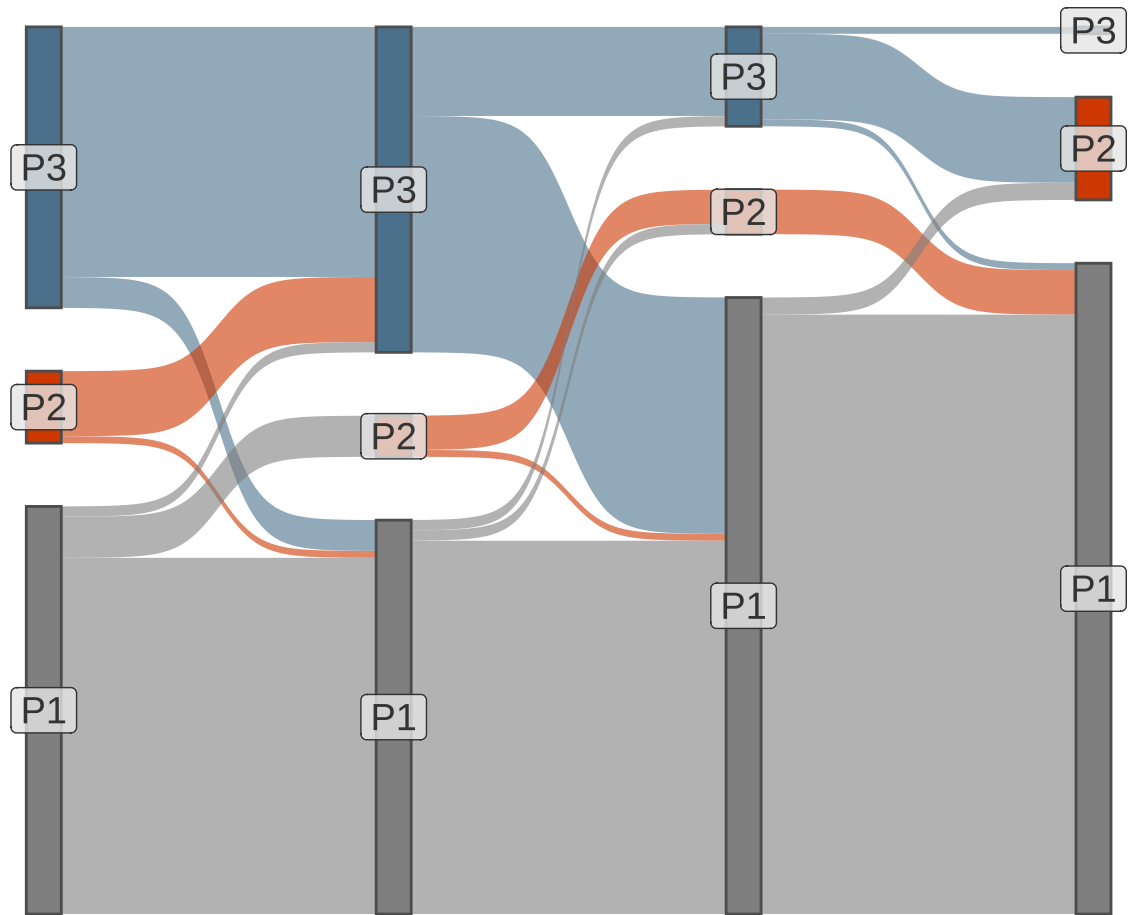

newGrp3s1

newGrp3s2

newGrp3s3

newGrp3

X

Supplement: Supplementary file 8 [file Data_Sheet_1.ZIP › sankeyPGrp3IRRI.pdf]

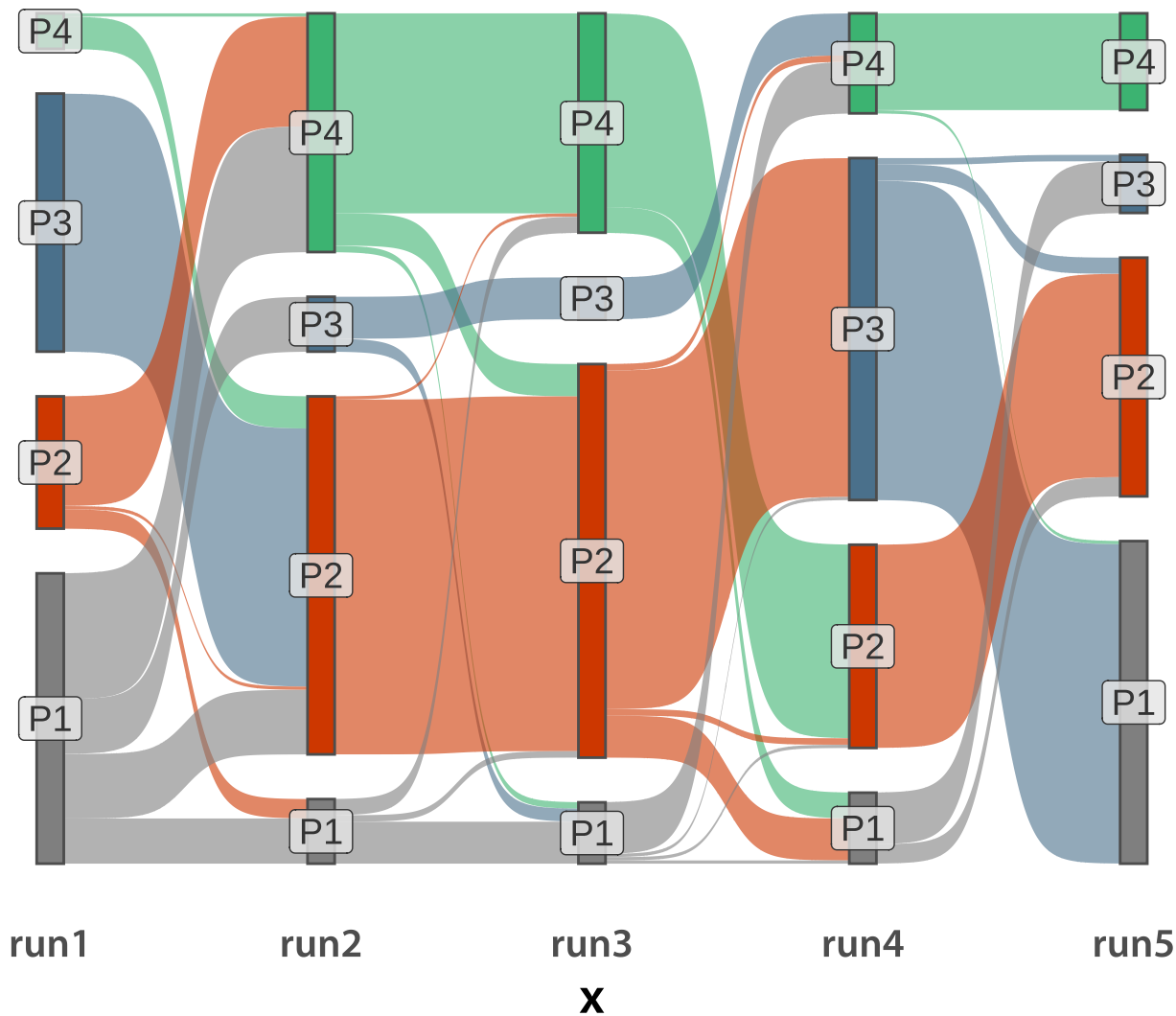

Supplement: Supplementary file 8 [file Data_Sheet_1.ZIP › sankeyPGrp4IRRIraw.pdf]

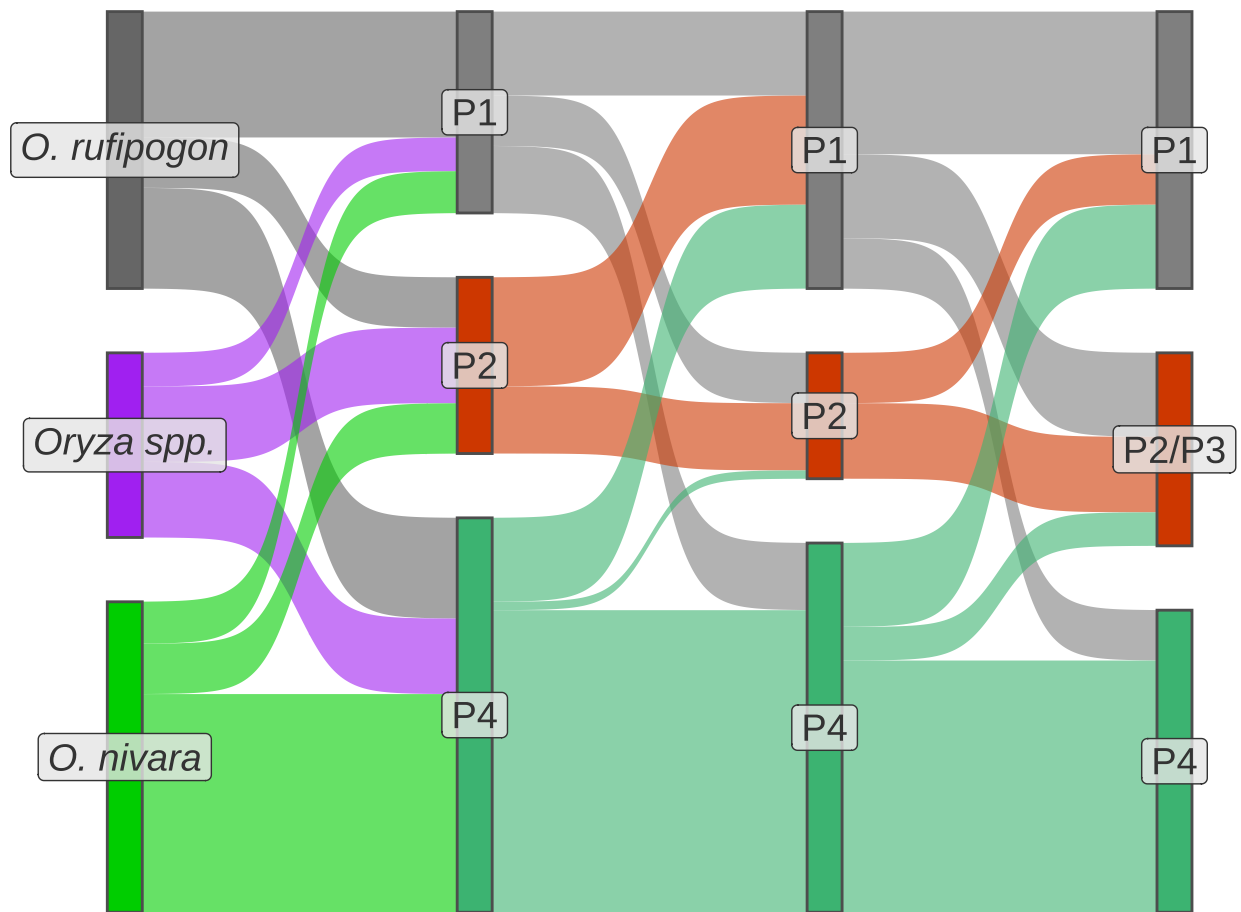

species

Cornell  
correlation (7)

Cornell  
all traits

IRRI  
all traits

Supplement: Supplementary file 8 [file Data_Sheet_1.ZIP › sankeyPGrpCUcor.pdf]

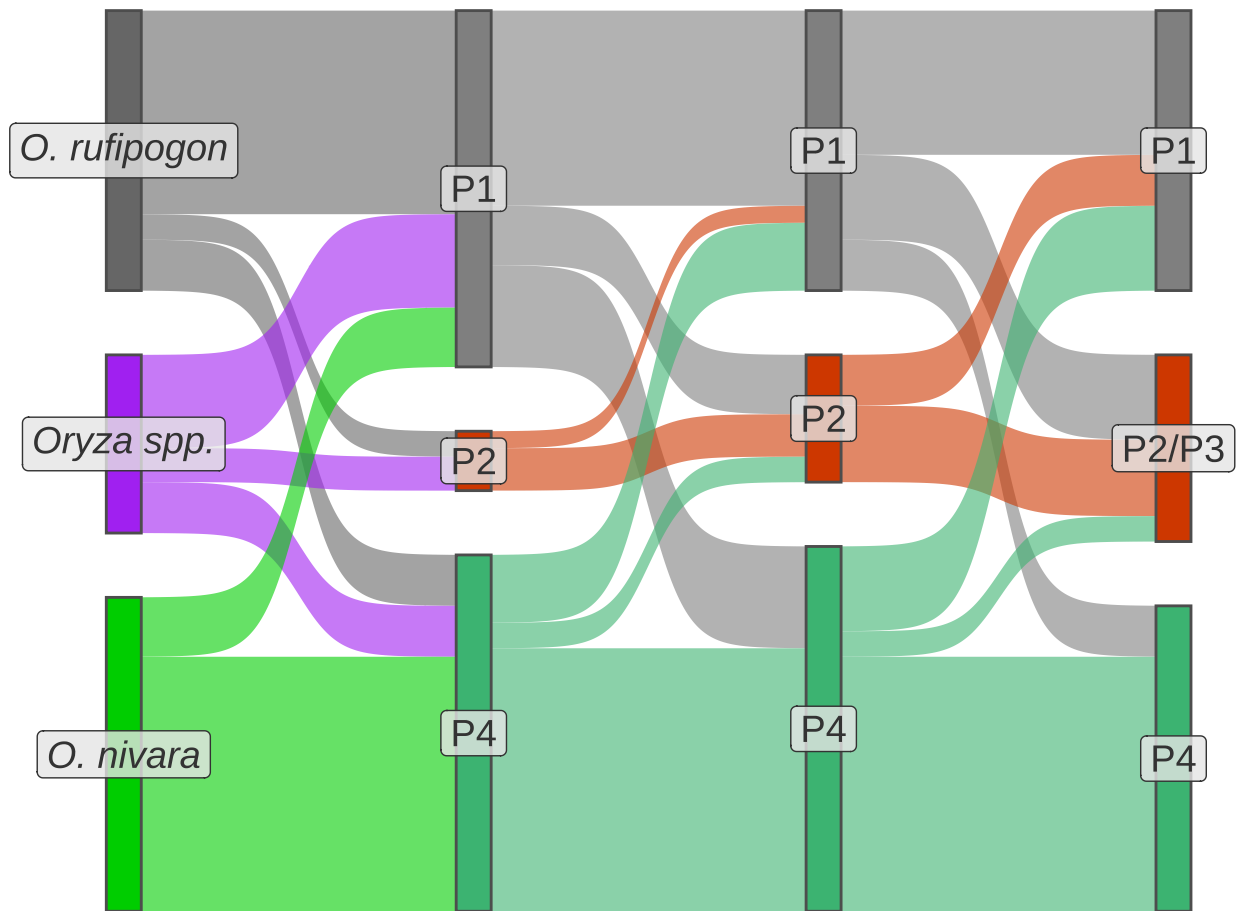

species

Dale  
Bumpers

Cornell

IRRI  
all traits

Supplement: Supplementary file 8 [file Data_Sheet_1.ZIP › sankeyPGrpCUDBp3.pdf]

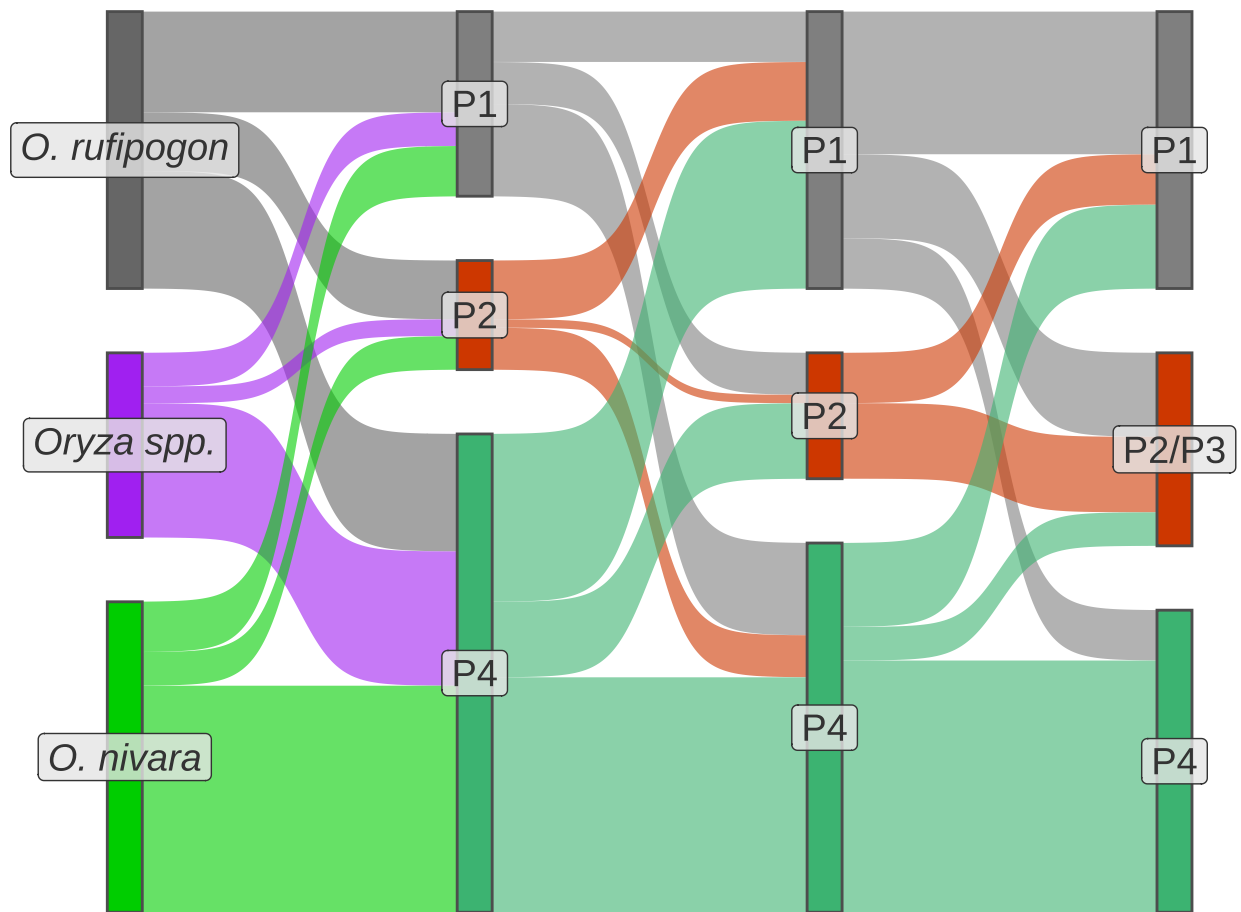

species

Cornell  
IRR1 5 (11) traits

Cornell  
all traits

IRR1  
all traits

Supplement: Supplementary file 8 [file Data_Sheet_1.ZIP › sankeyPGrpCUirri11.pdf]

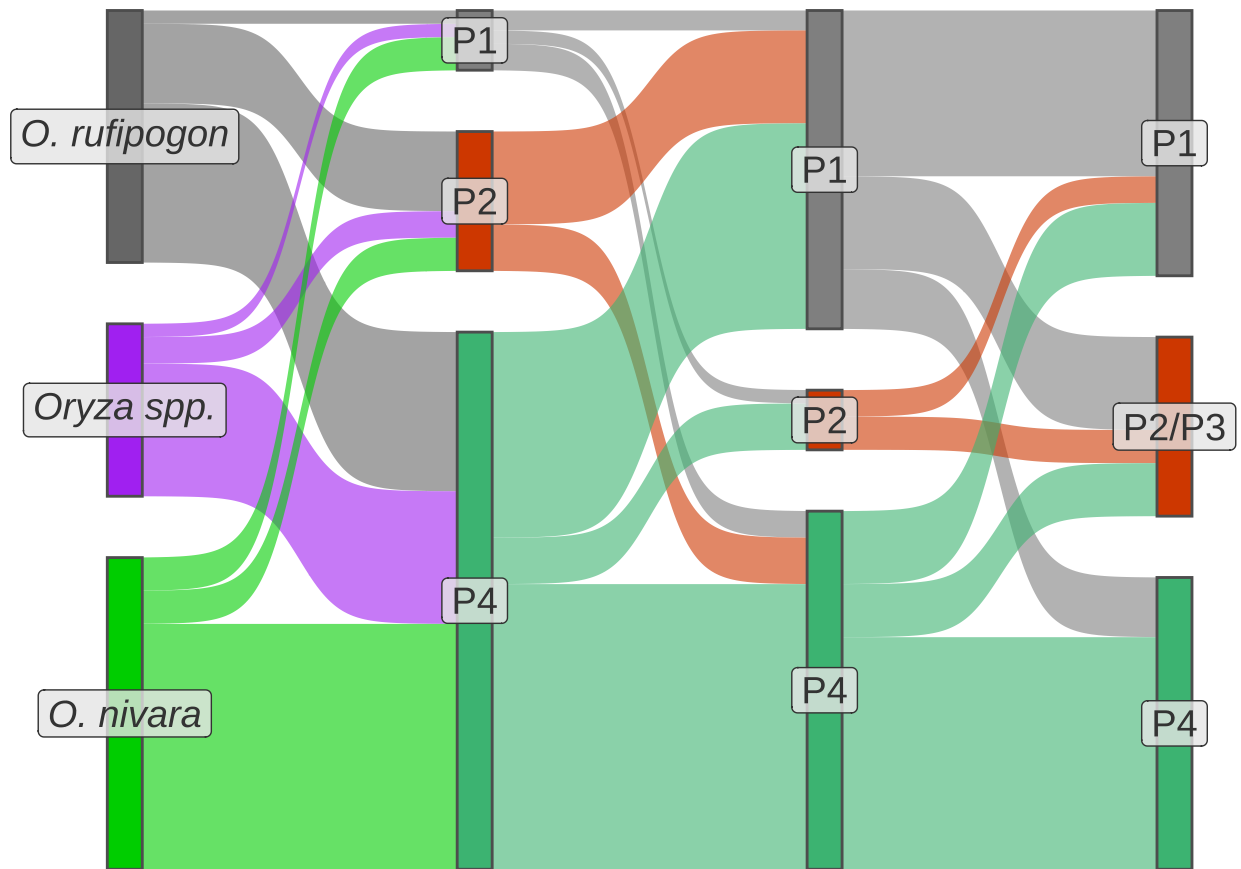

species

Dale Bumpers  
correlation (6)

Dale  
Bumpers  
all traits

IRRI  
all traits

Supplement: Supplementary file 8 [file Data_Sheet_1.ZIP › sankeyPGrpDBcor.pdf]

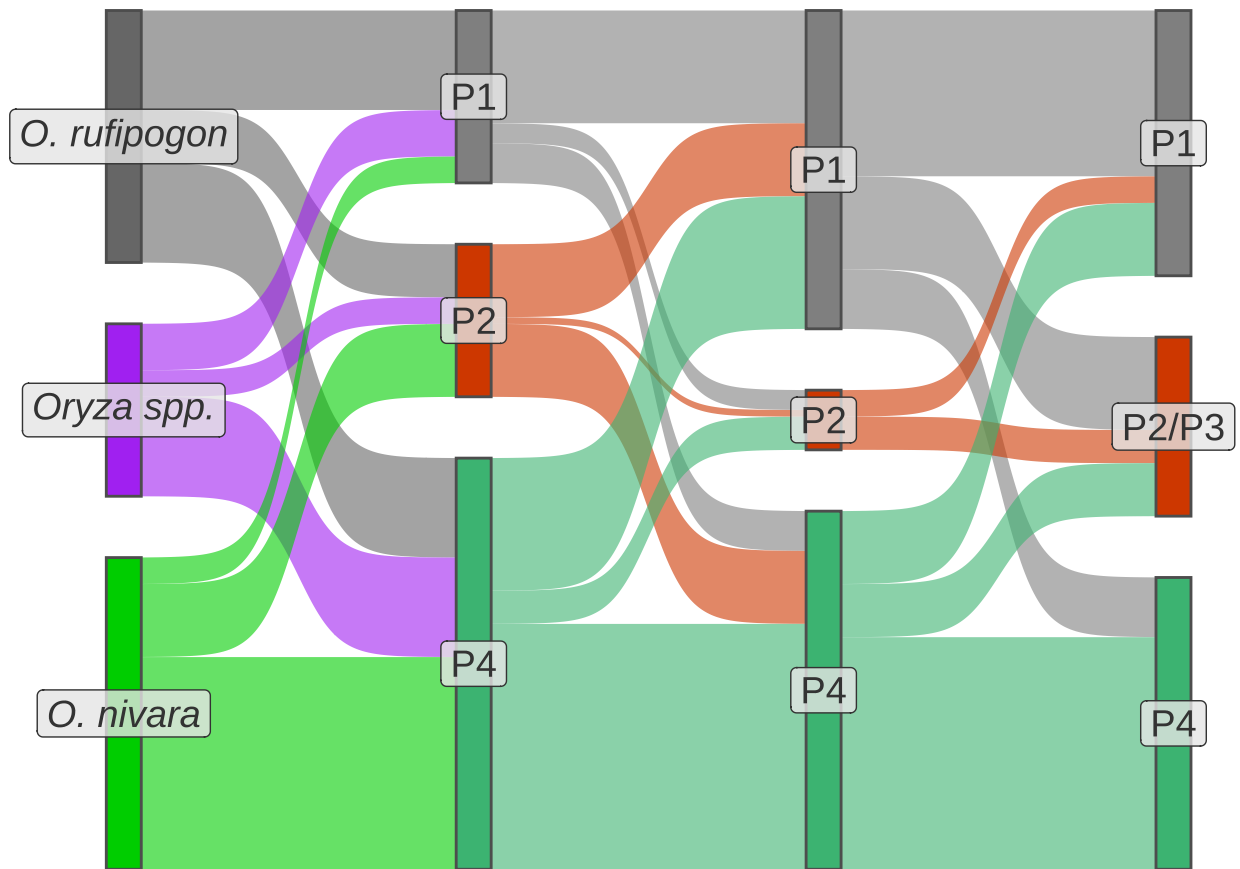

species

Dale Bumpers  
IRRI 8 (11) traits

Dale  
Bumpers  
all traits

IRRI  
all traits

Supplement: Supplementary file 8 [file Data_Sheet_1.ZIP › sankeyPGrpDBirri11.pdf]

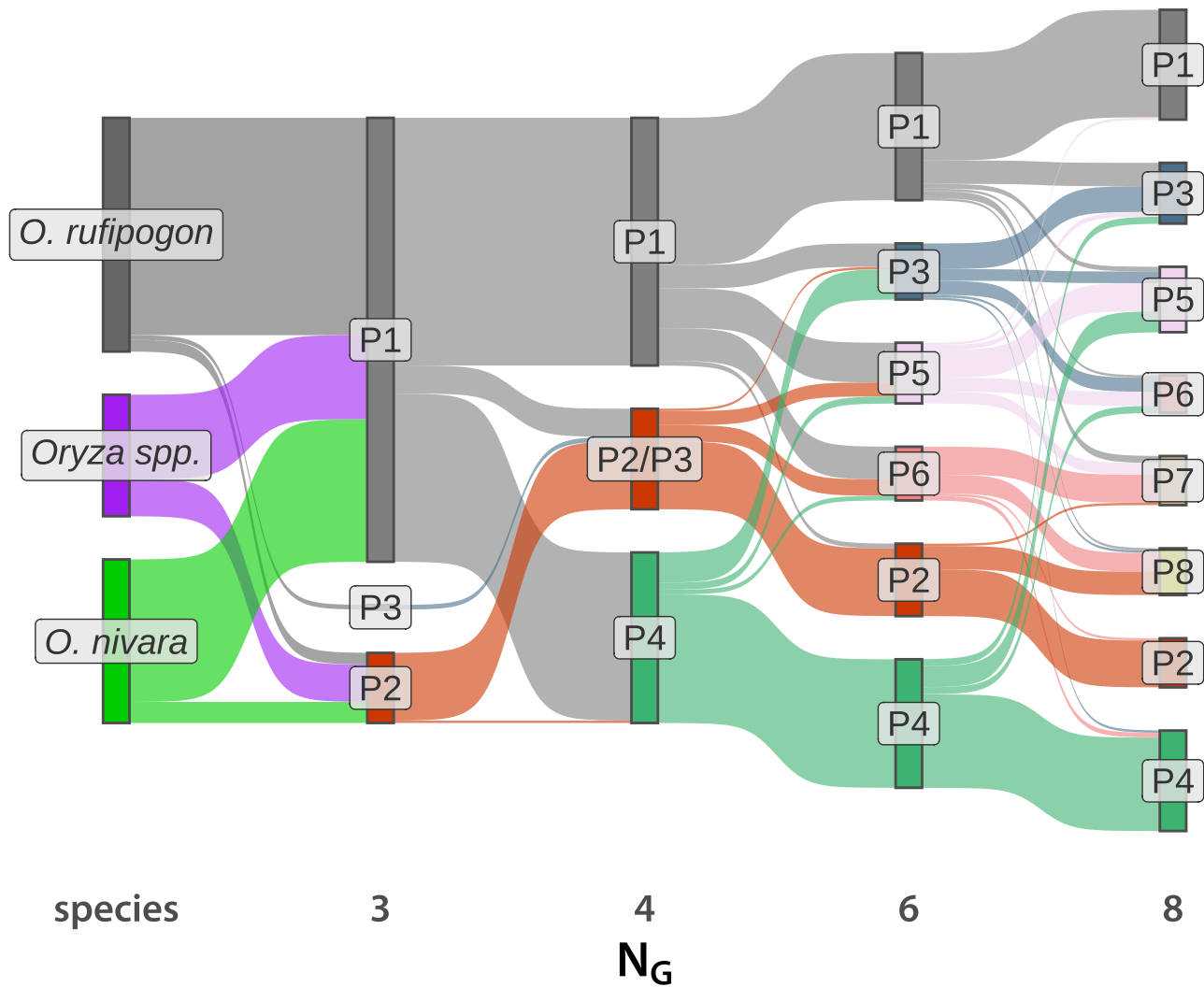

Supplement: Supplementary file 8 [file Data_Sheet_1.ZIP › sankeyPGrpIRRI.pdf]

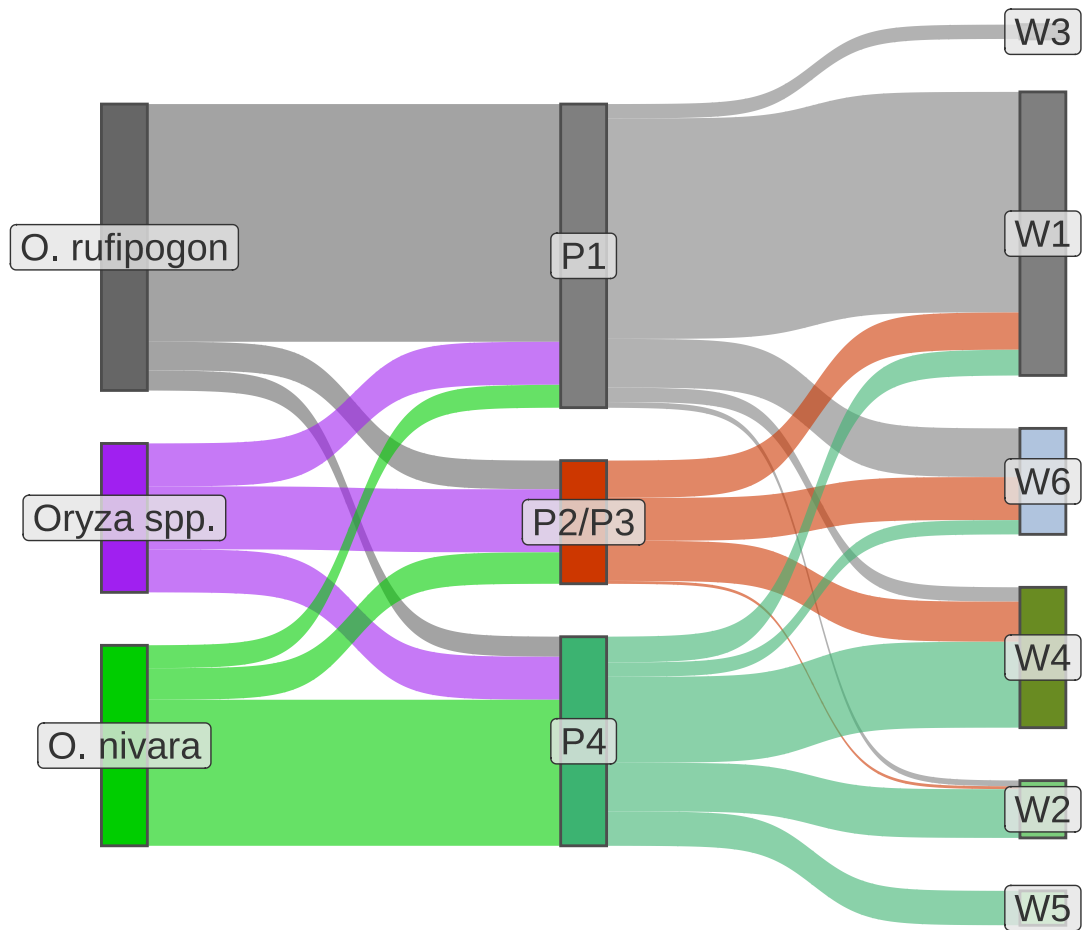

species

phenotypic  
groups

genetic  
subpopulations

Supplement: Supplementary file 8 [file Data_Sheet_1.ZIP › sankeyPGrpPopsIRRI.pdf]

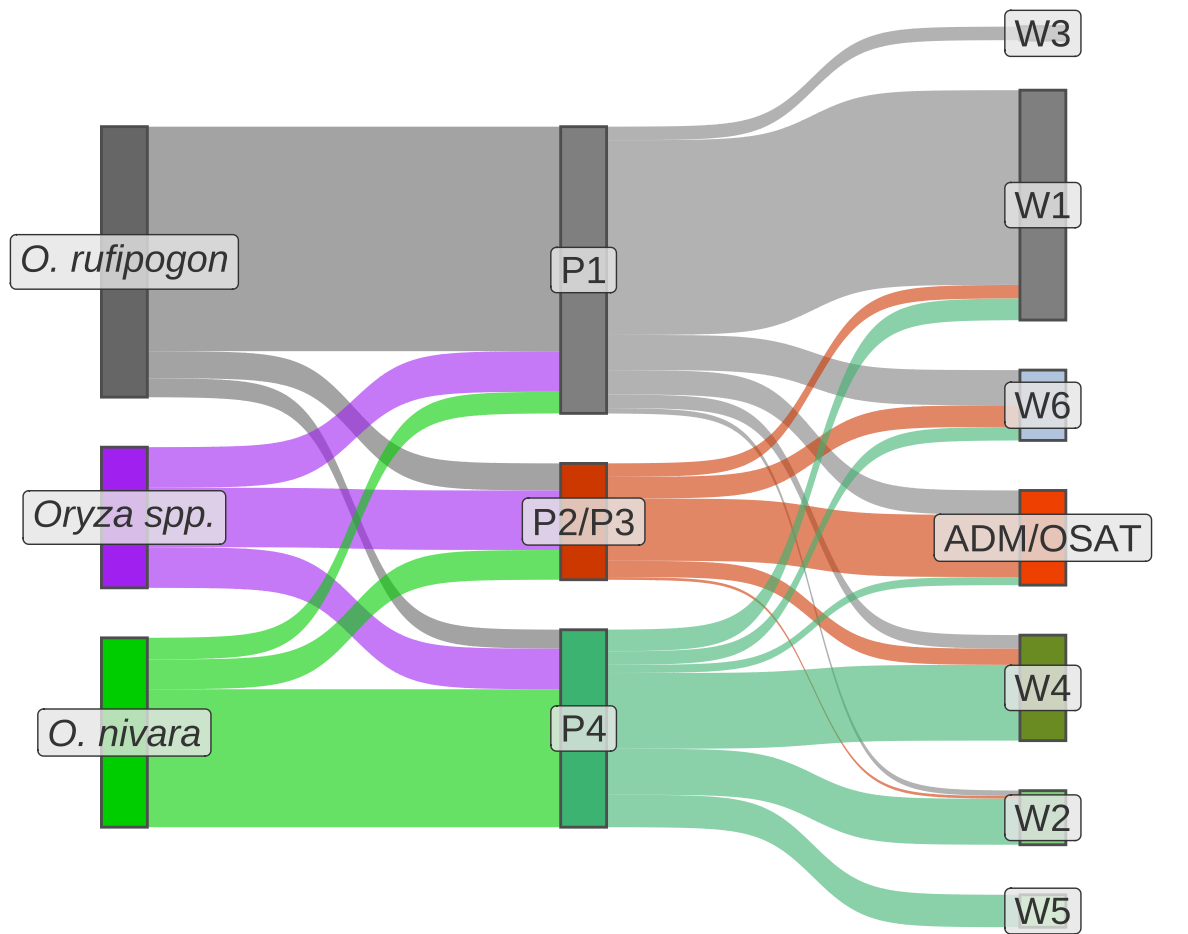

species

phenotypic  
groups

genetic  
subpopulations

Supplement: Supplementary file 8 [file Data_Sheet_1.ZIP › sankeyPGrpPopsIRRIsat.pdf]

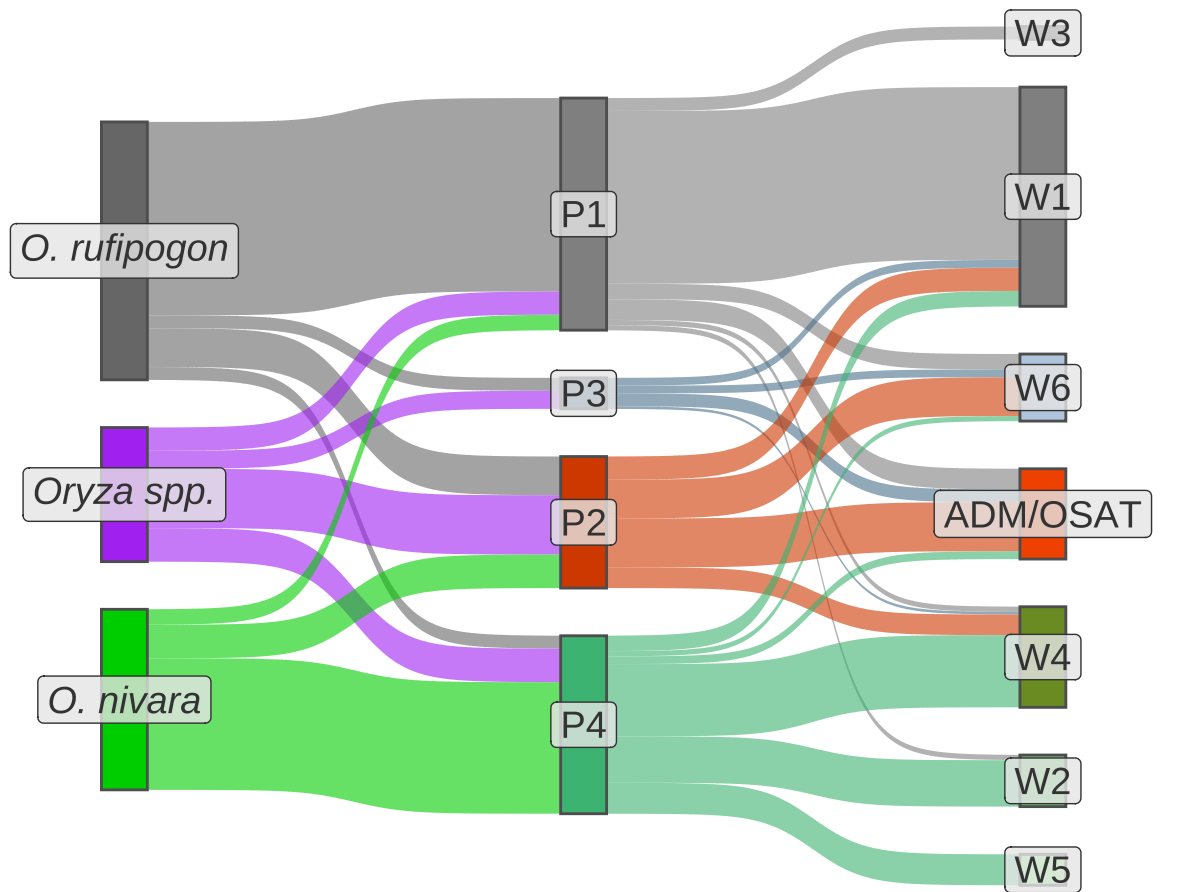

species

phenotypic  
groups  
(16 traits)

genetic  
subpopulations

Supplement: Supplementary file 8 [file Data_Sheet_1.ZIP › sankeyPGrpPopsIRRIsatSubs.pdf]

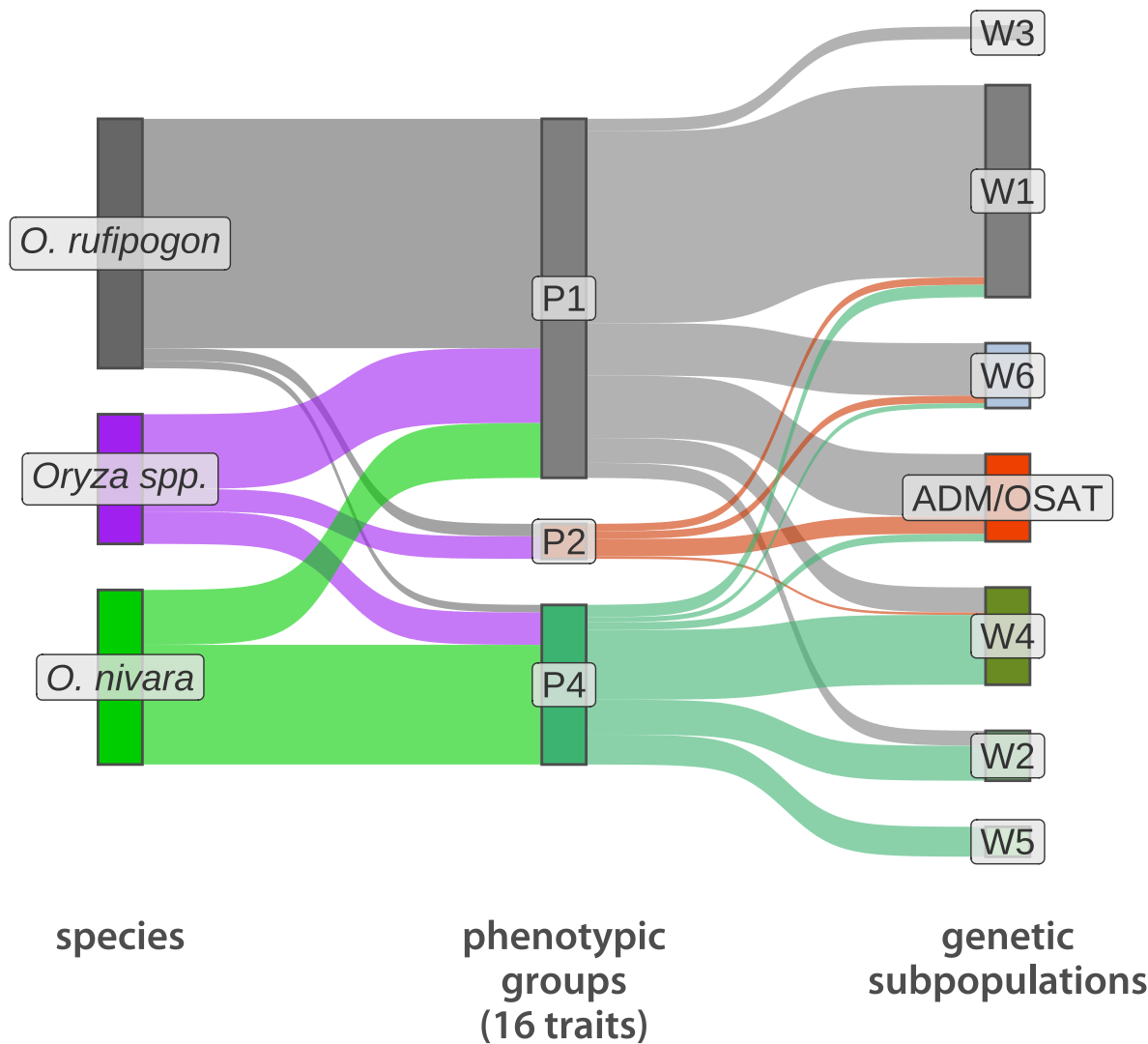

Supplement: Supplementary file 8 [file Data_Sheet_1.ZIP › sankeyPGrpPopsIRRIsatSubsP3.pdf]

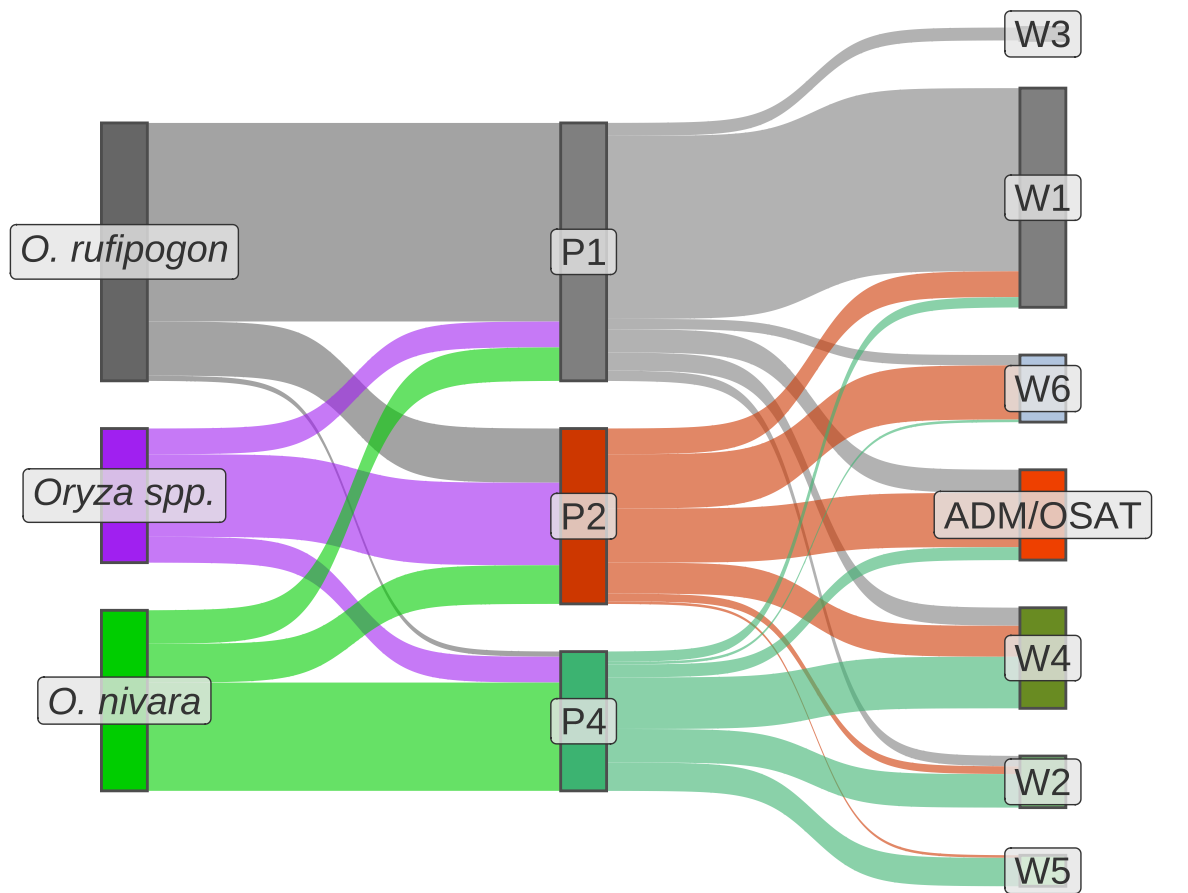

species

phenotypic  
groups  
(11 traits)

genetic  
subpopulations

Supplement: Supplementary file 8 [file Data_Sheet_1.ZIP › sankeyPGrpPopsIRRIsatSubsP3cor.pdf]

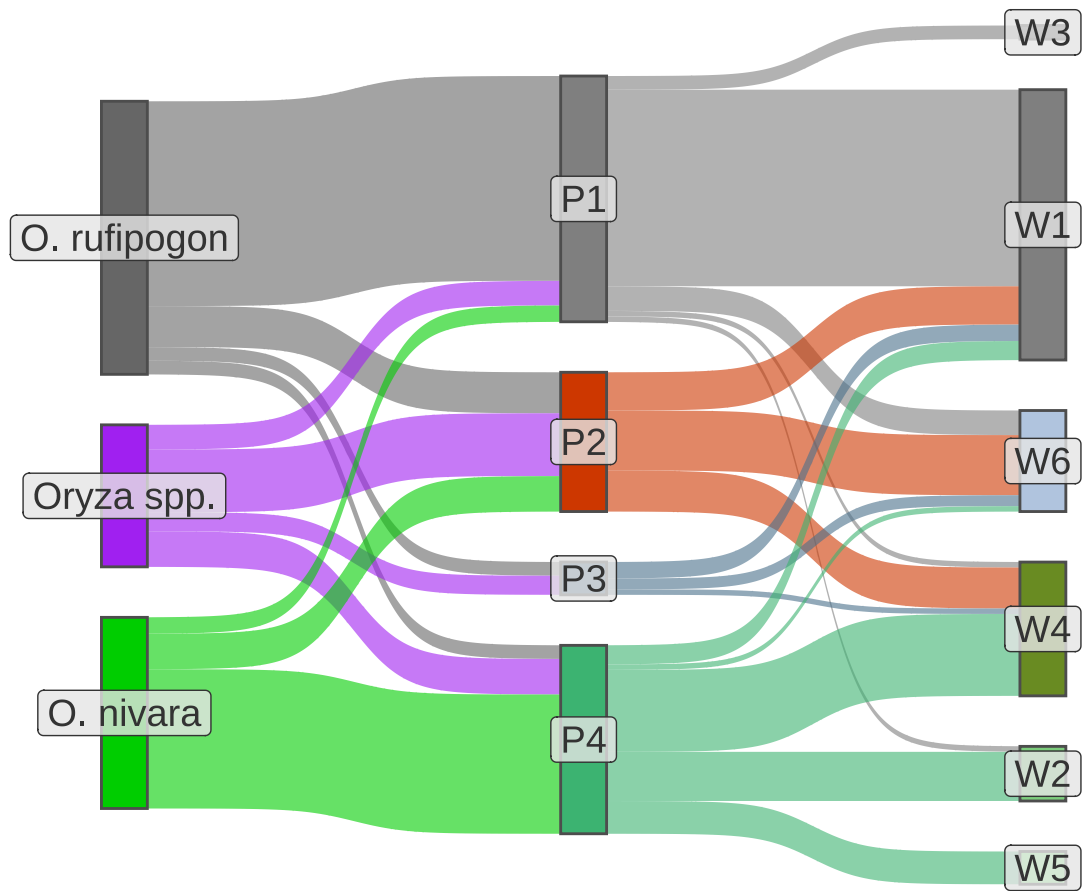

species

phenotypic  
groups  
(16 traits)

genetic  
subpopulations

Supplement: Supplementary file 8 [file Data_Sheet_1.ZIP › sankeyPGrpPopsIRRIsubs.pdf]

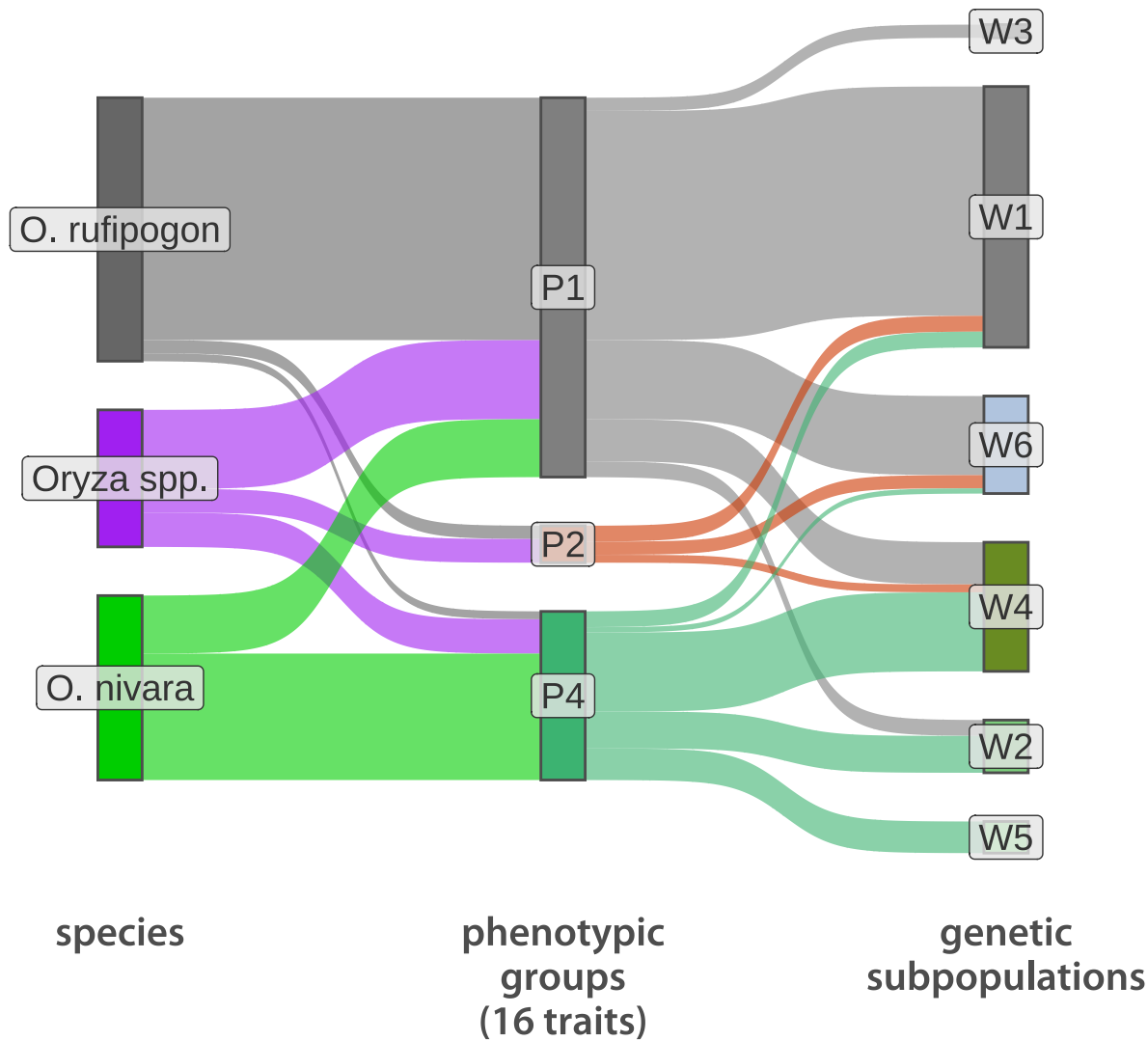

Supplement: Supplementary file 8 [file Data_Sheet_1.ZIP › sankeyPGrpPopsIRRIsubsP3.pdf]

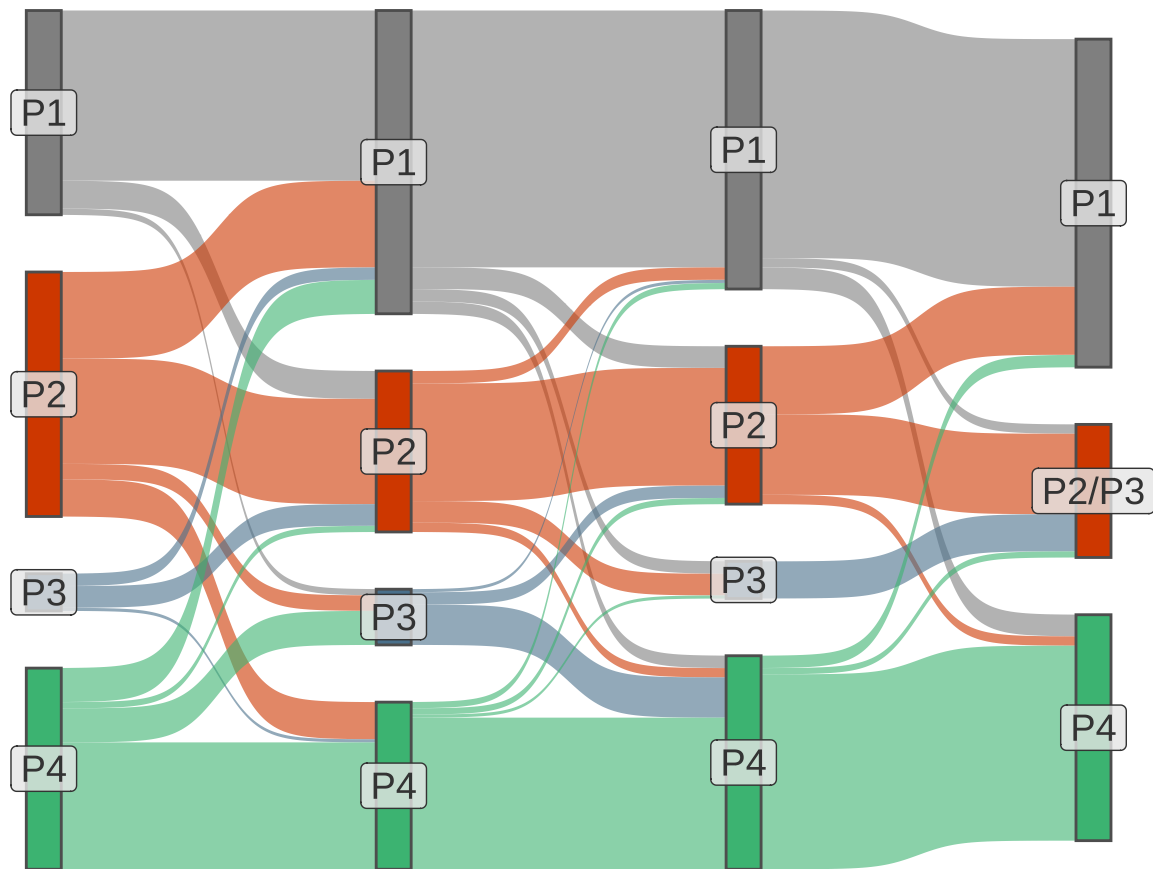

top by  
value  
9 traits

top by  
correlation  
11 traits

value  
+ correlation  
16 traits

all  
data  
32 traits

Supplement: Supplementary file 8 [file Data_Sheet_1.ZIP › sankeyPGrpSubsIRRI.pdf]

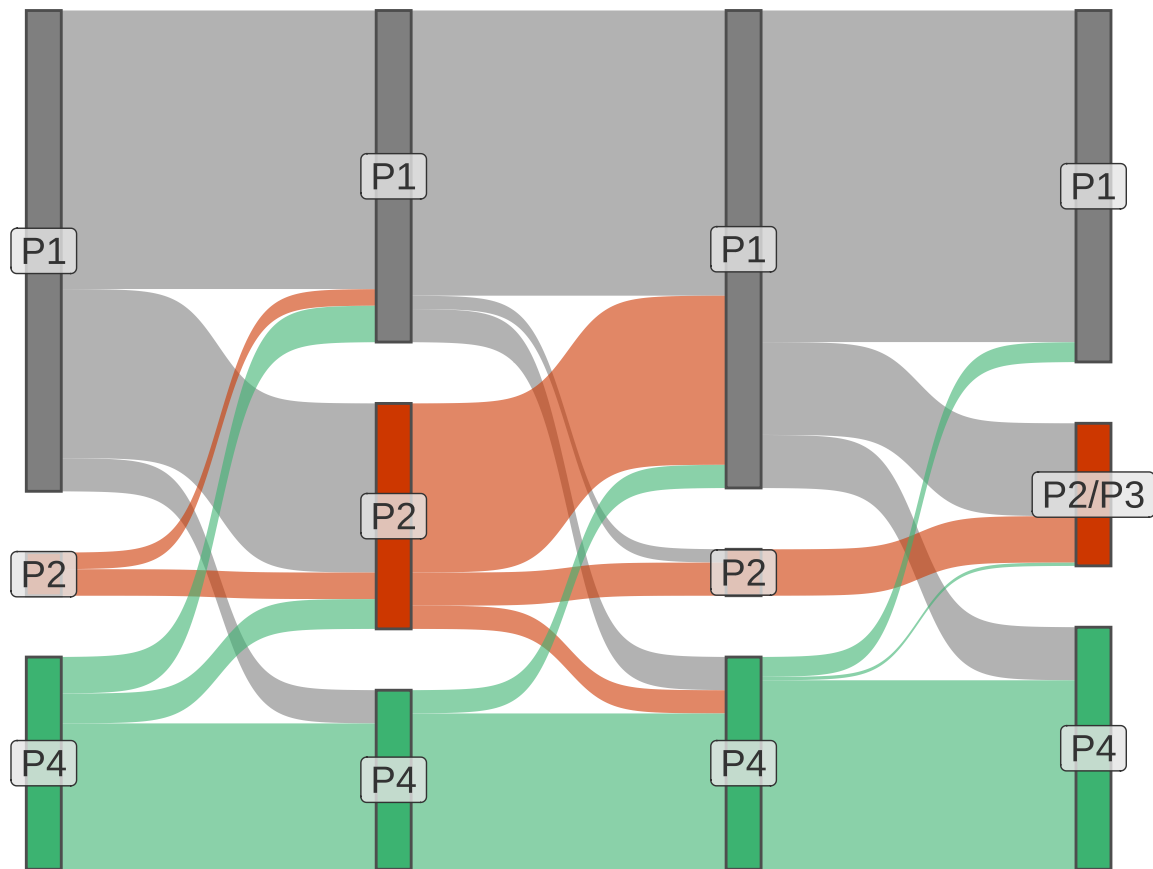

Supplement: Supplementary file 8 [file Data_Sheet_1.ZIP › sankeyPGrpSubsIRRIP3.pdf]

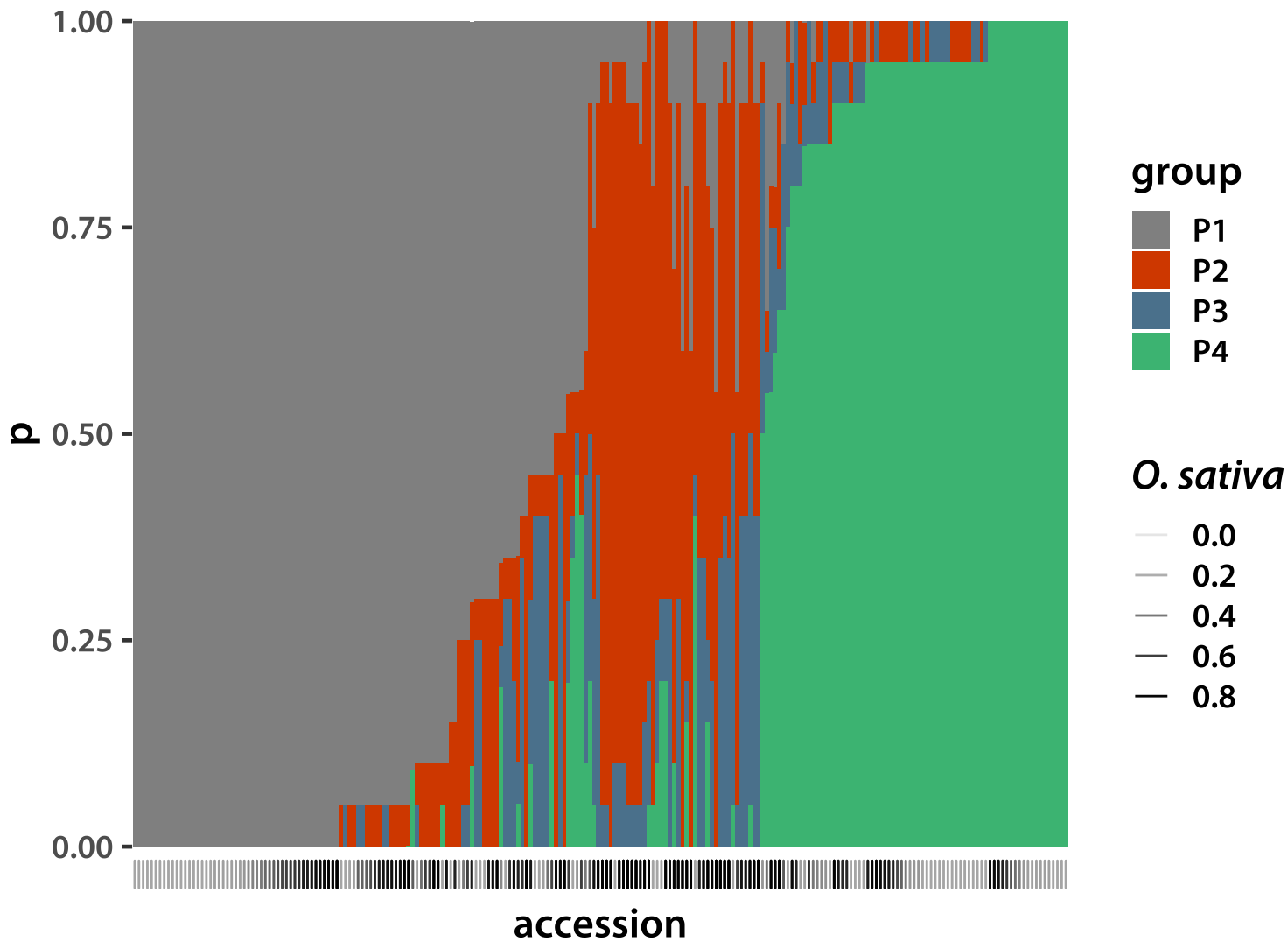

Supplement: Supplementary file 8 [file Data_Sheet_1.ZIP › structIRRIP4.pdf]

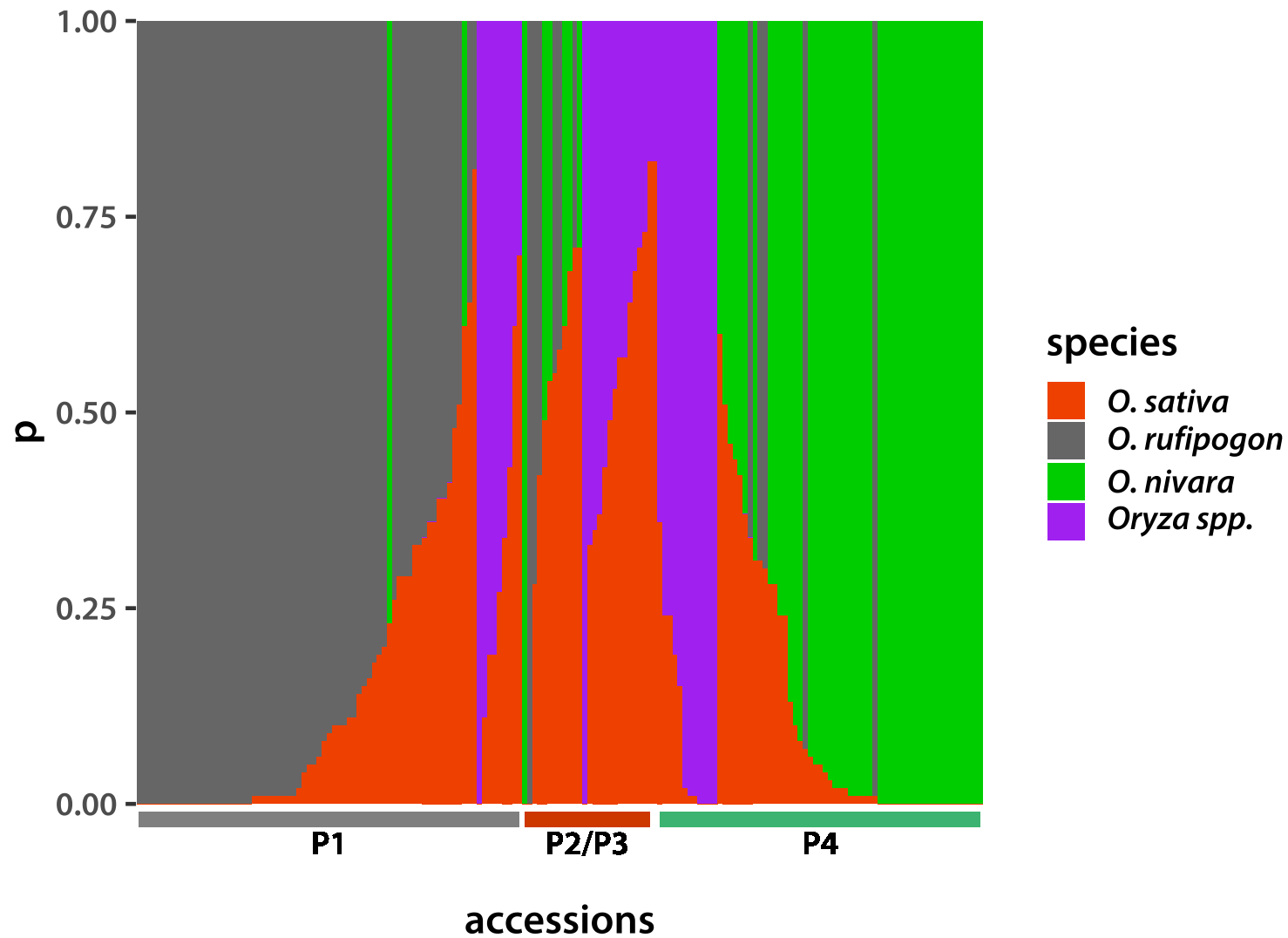

Supplement: Supplementary file 8 [file Data_Sheet_1.ZIP › structSppOsat.pdf]

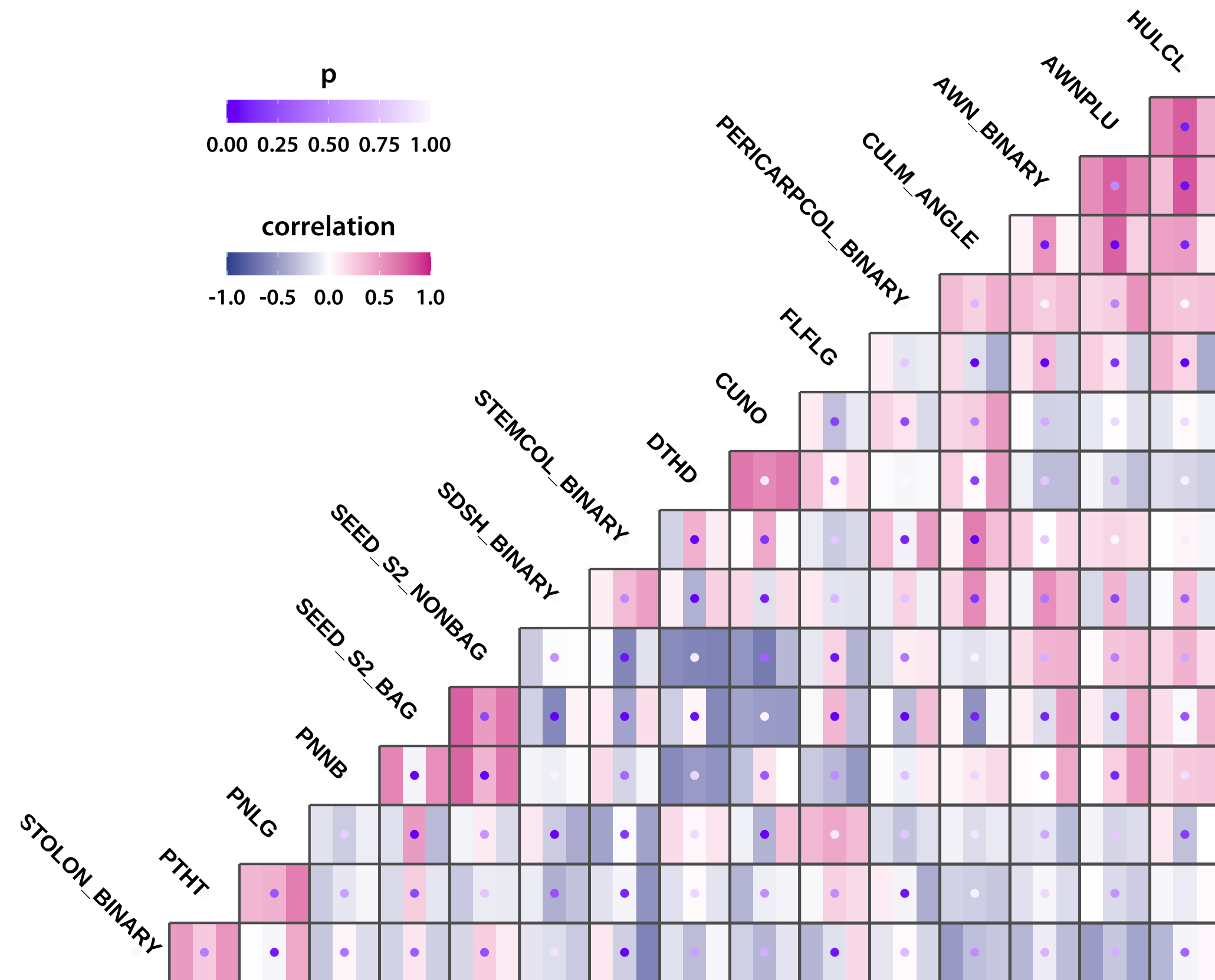

Supplement: Supplementary file 8 [file Data_Sheet_1.ZIP › traitCorCUp4.pdf]

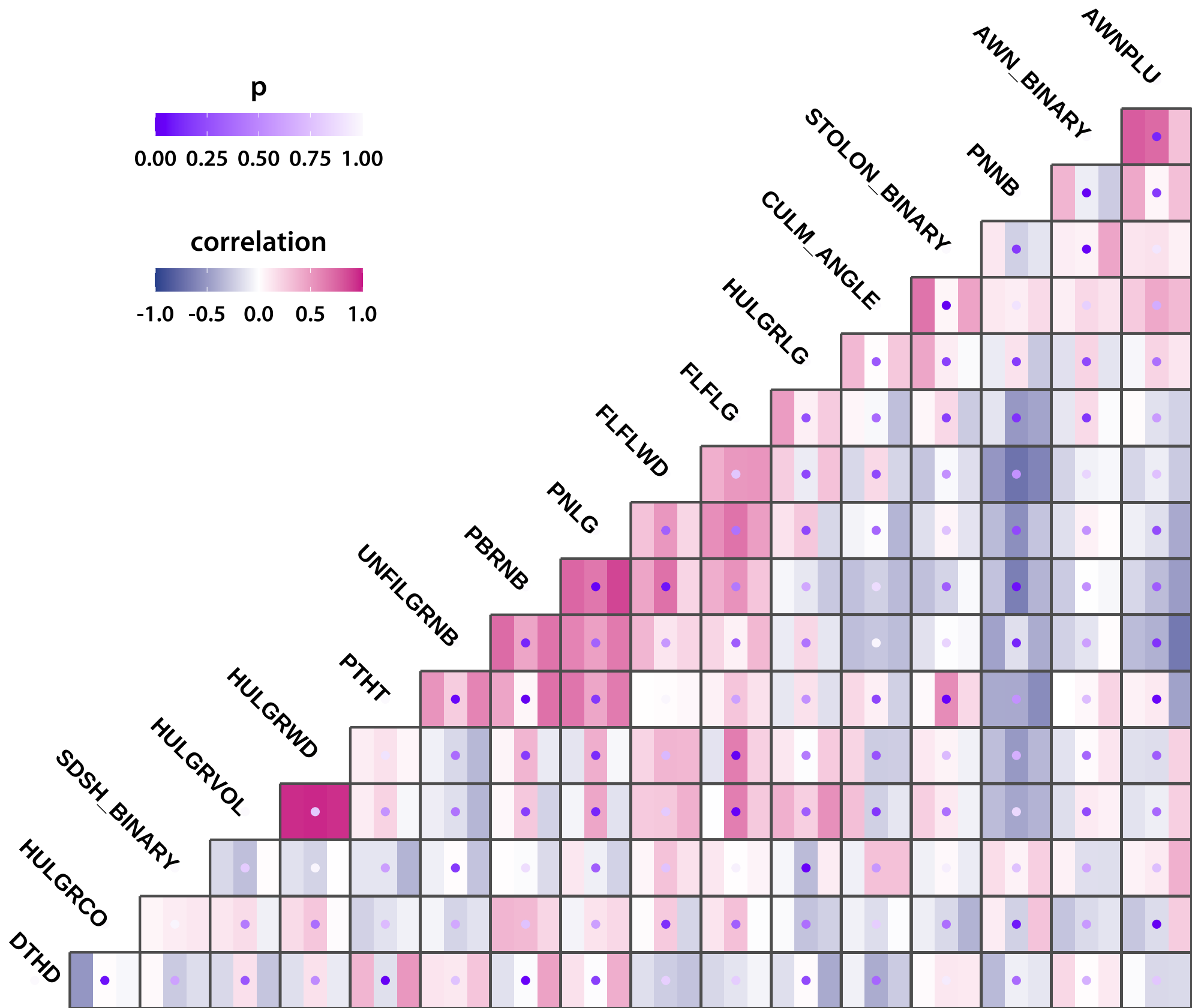

Supplement: Supplementary file 8 [file Data_Sheet_1.ZIP › traitCorDBp4.pdf]

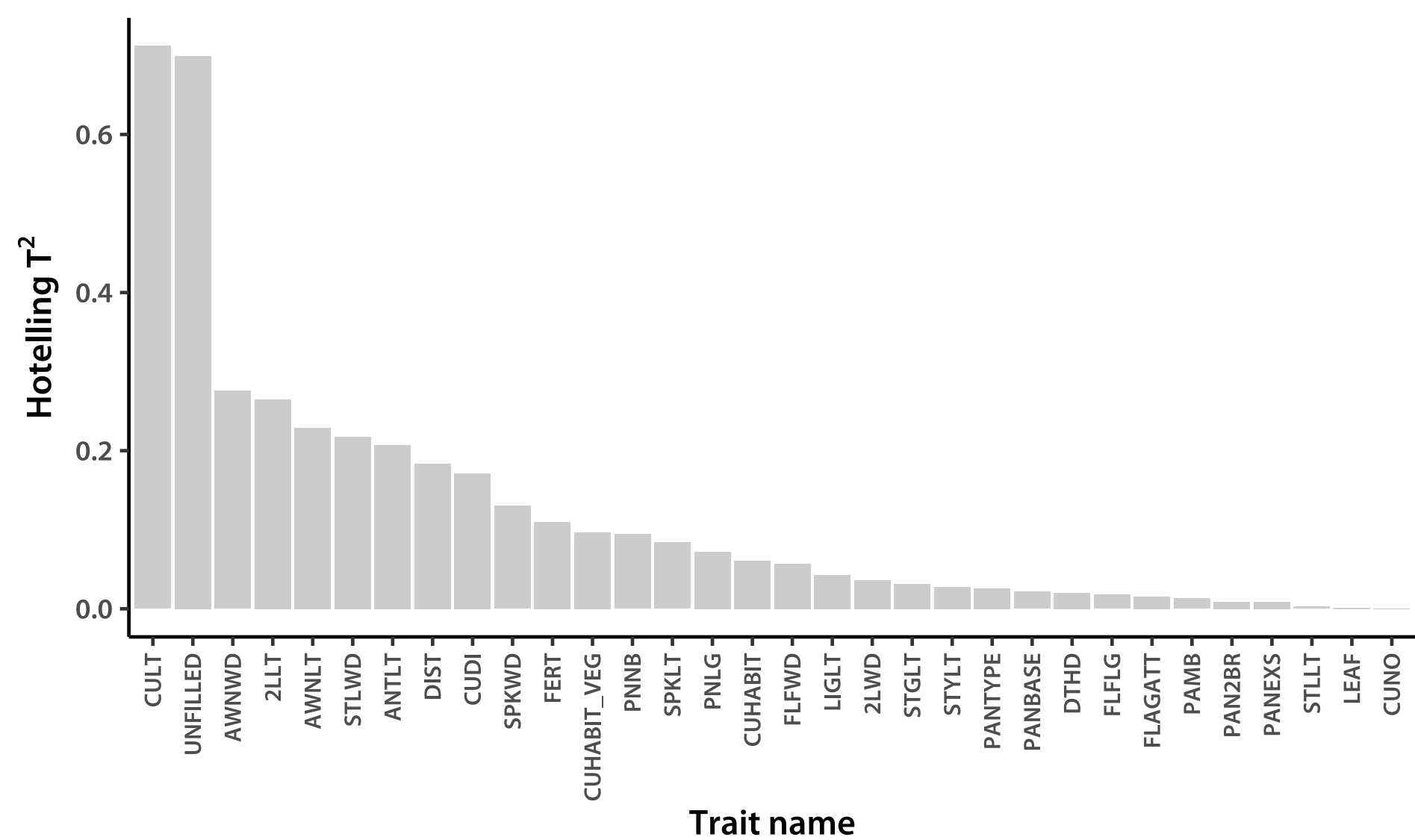

Supplement: Supplementary file 8 [file Data_Sheet_1.ZIP › traitMhlIRRI.pdf]

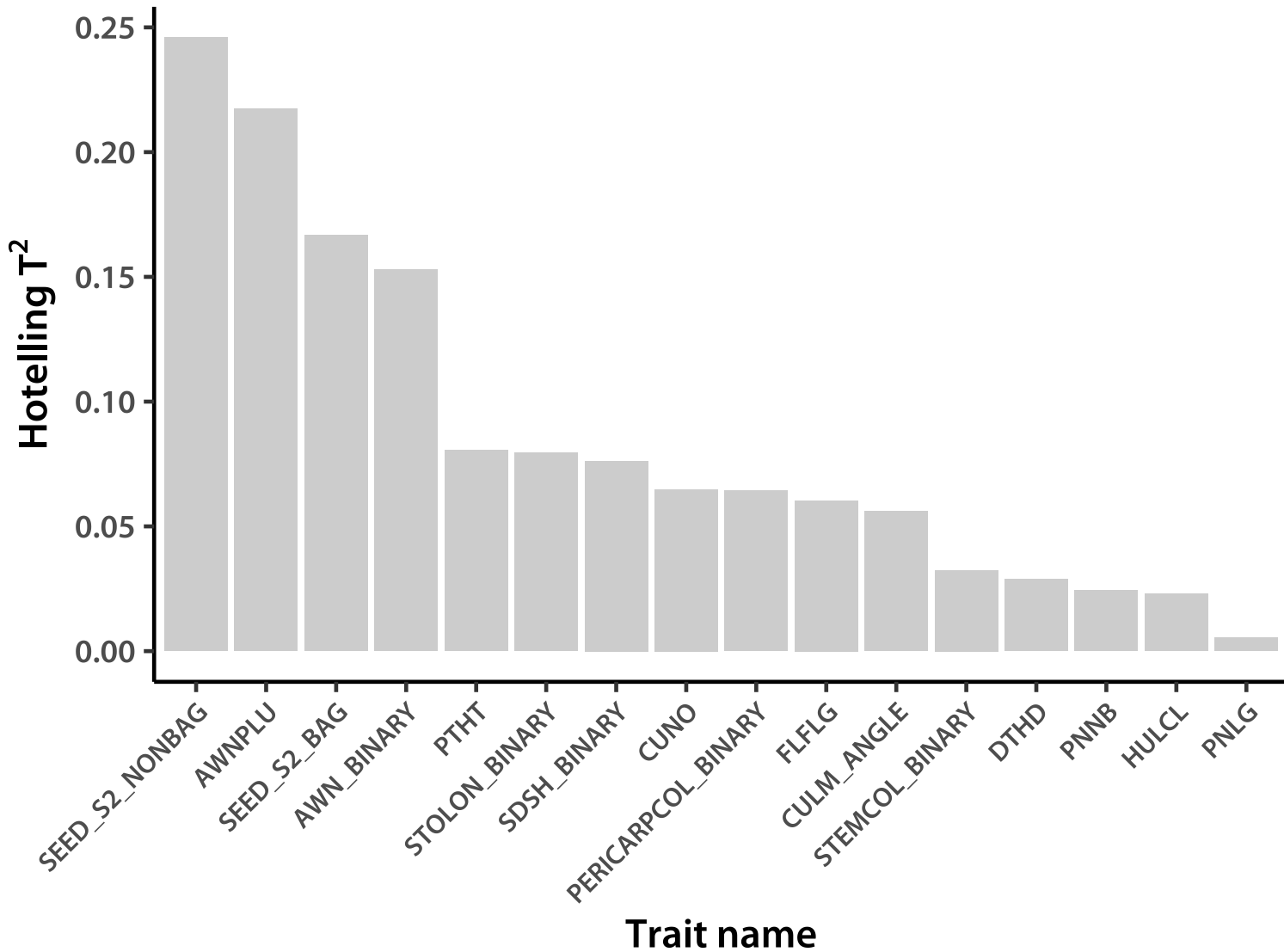

Supplement: Supplementary file 8 [file Data_Sheet_1.ZIP › traitMhlP4CU.pdf]

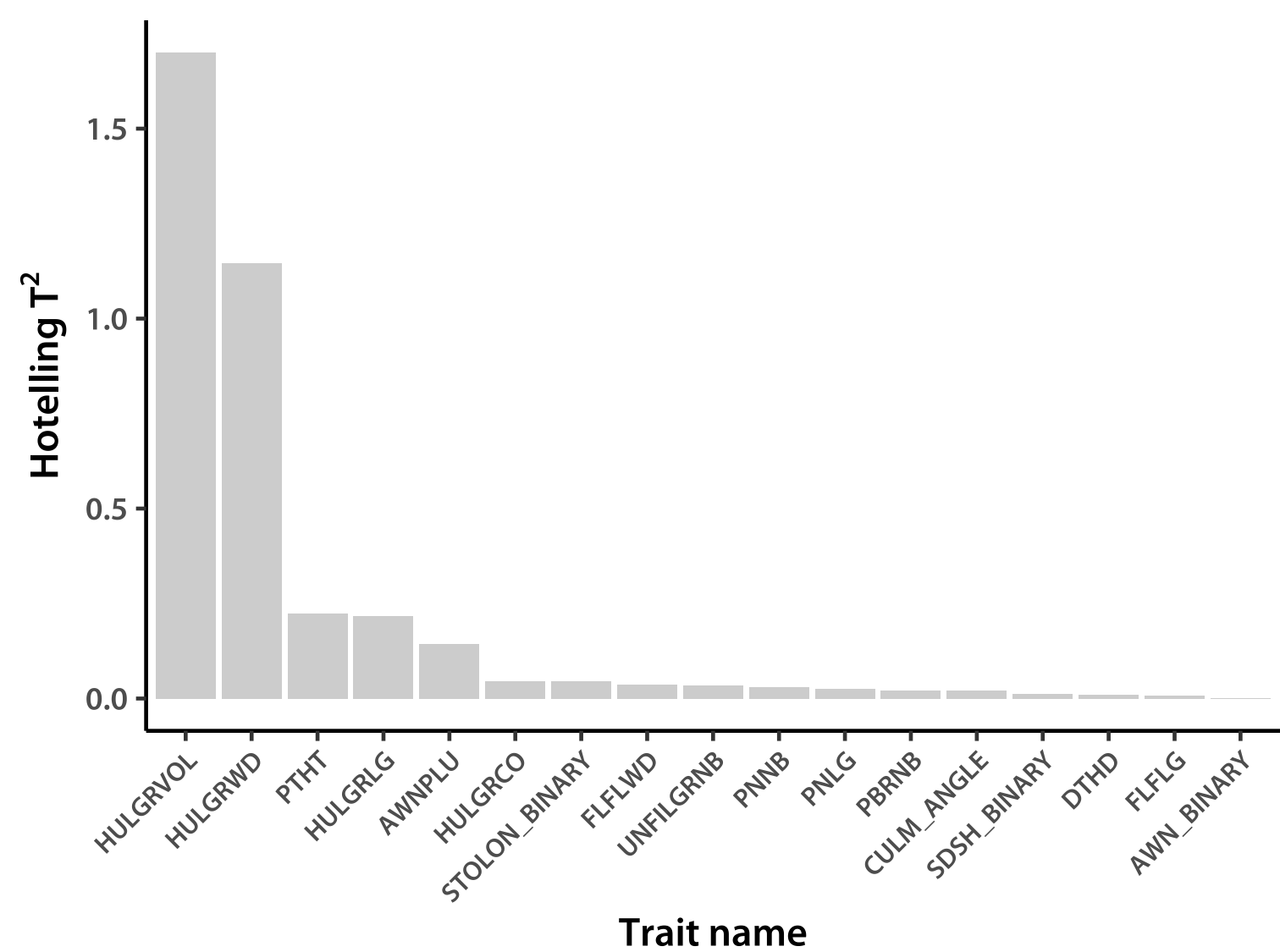

Supplement: Supplementary file 8 [file Data_Sheet_1.ZIP › traitMhlP4DB.pdf]

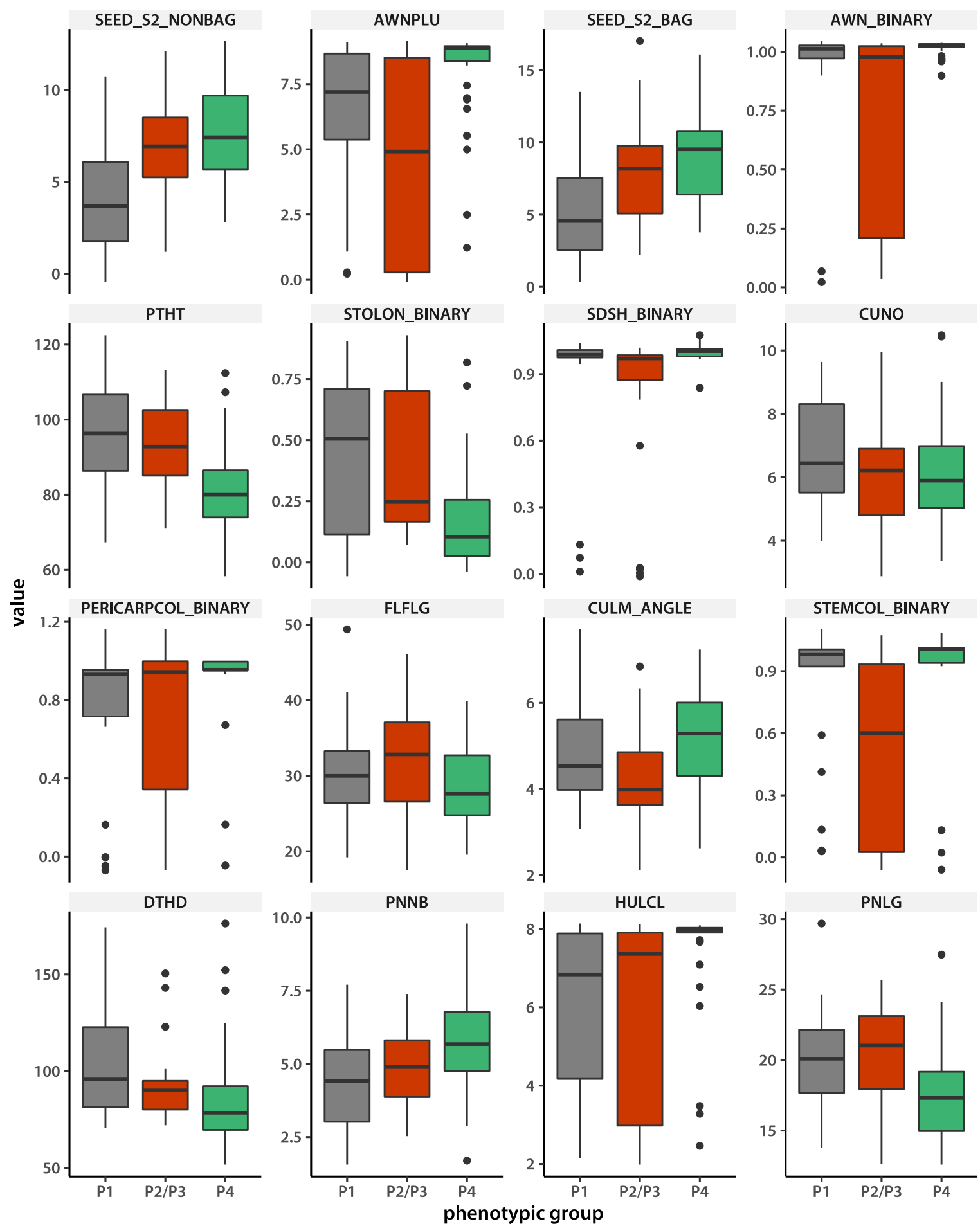

Supplement: Supplementary file 8 [file Data_Sheet_1.ZIP › traitsCUbxpP4.pdf]

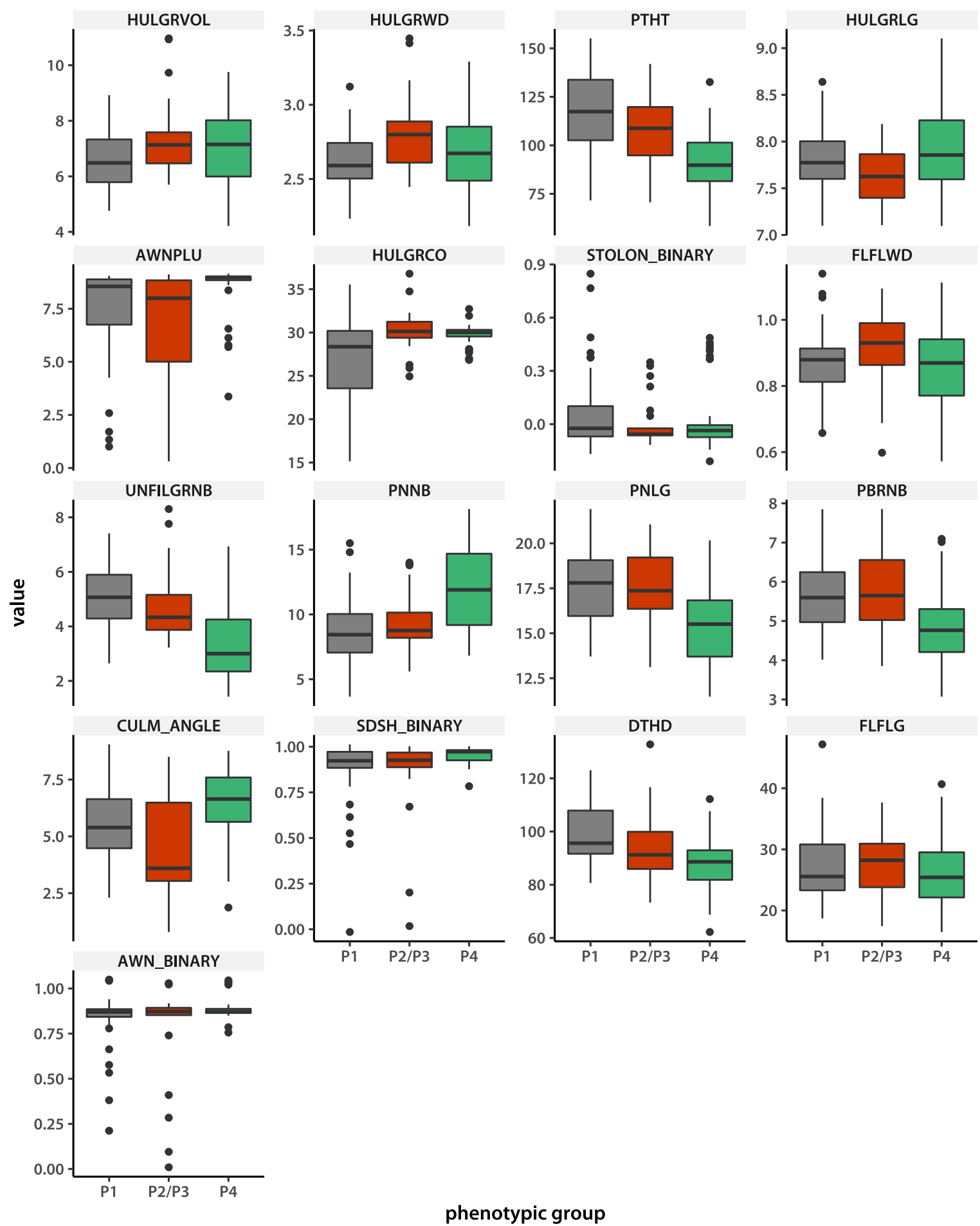

Supplement: Supplementary file 8 [file Data_Sheet_1.ZIP › traitsDBbxpP4.pdf]

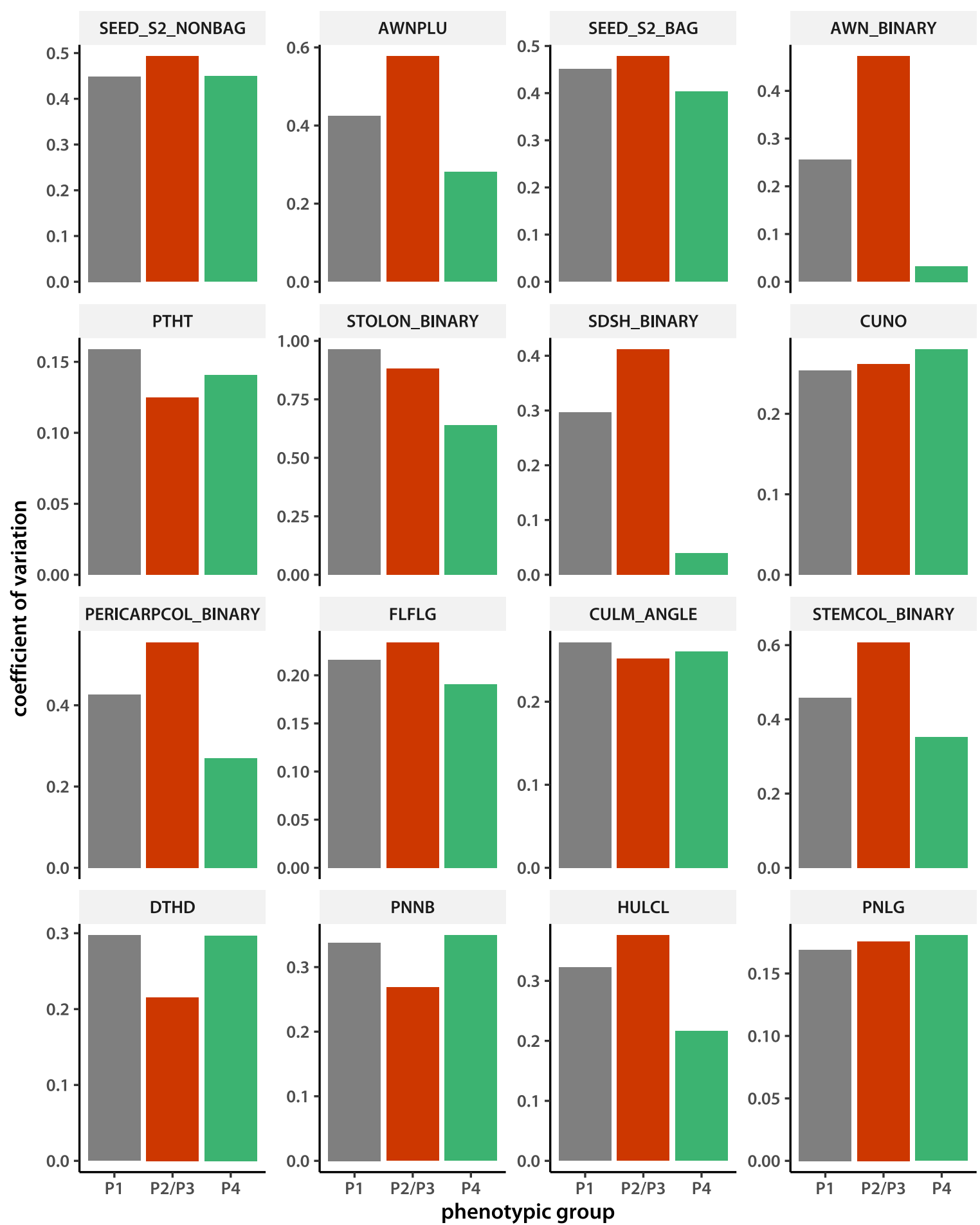

Supplement: Supplementary file 8 [file Data_Sheet_1.ZIP › traitSdCUbpP4.pdf]

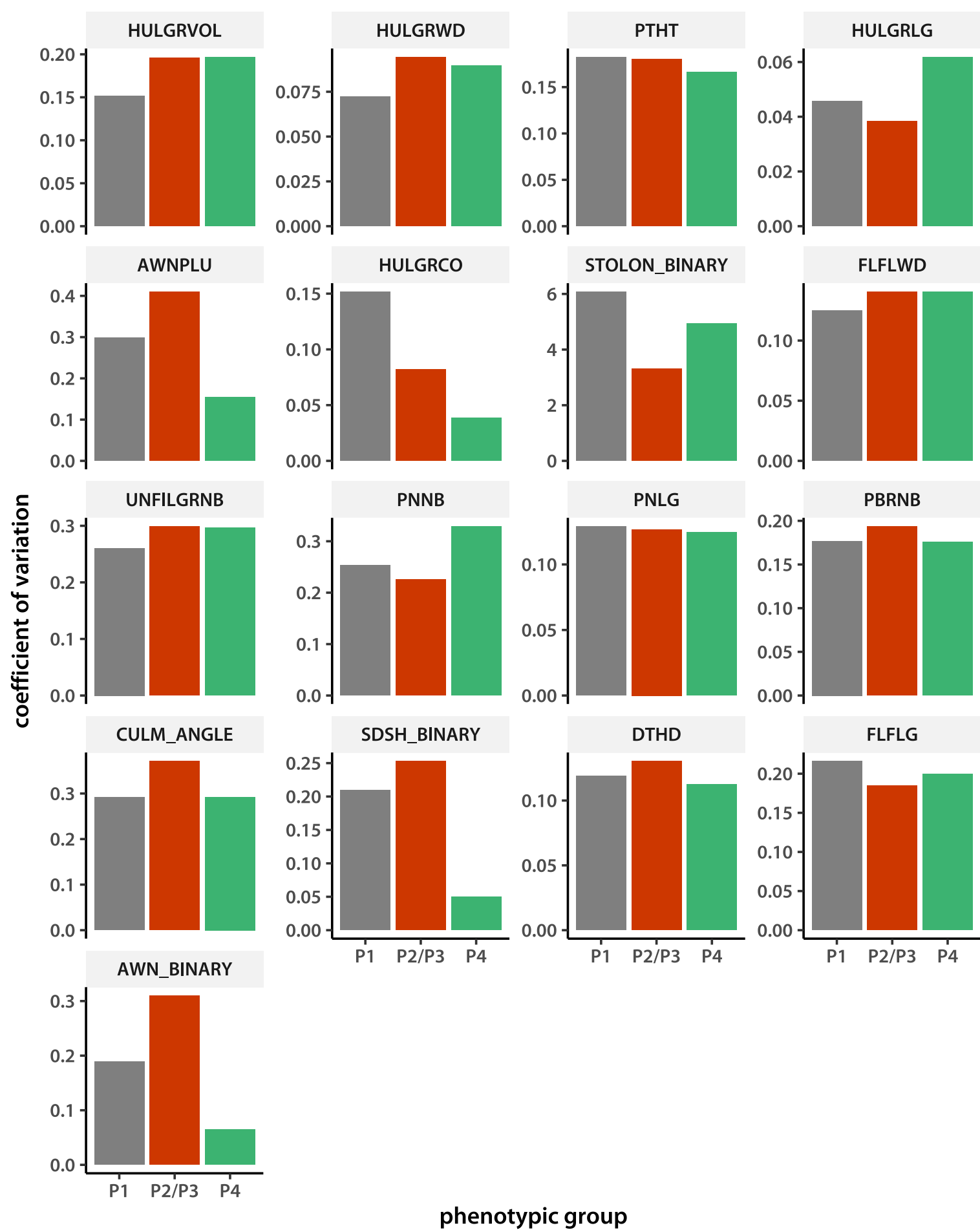

Supplement: Supplementary file 8 [file Data_Sheet_1.ZIP › traitSdDBbpP4.pdf]

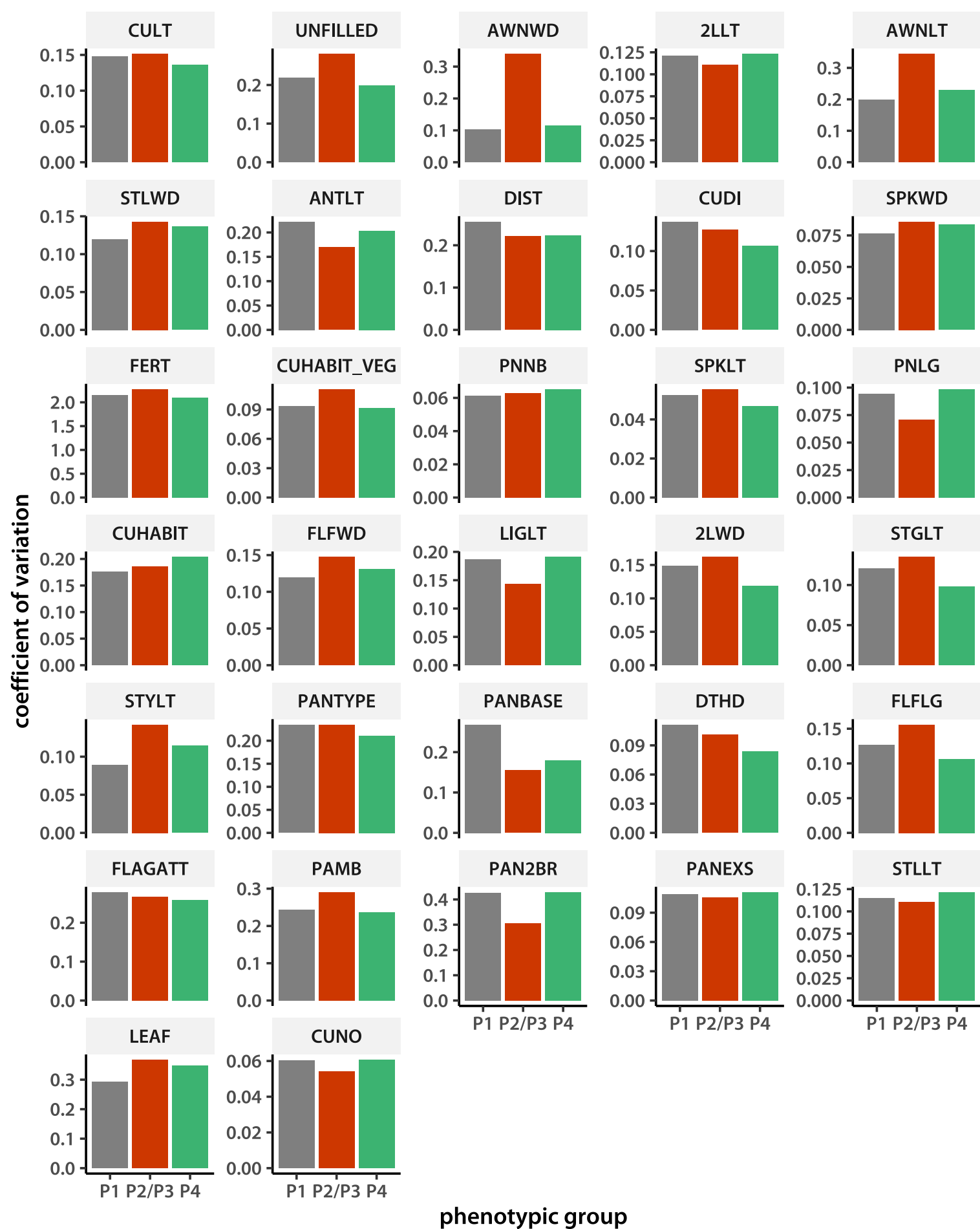

Supplement: Supplementary file 8 [file Data_Sheet_1.ZIP › traitSdIRRIbpP4.pdf]

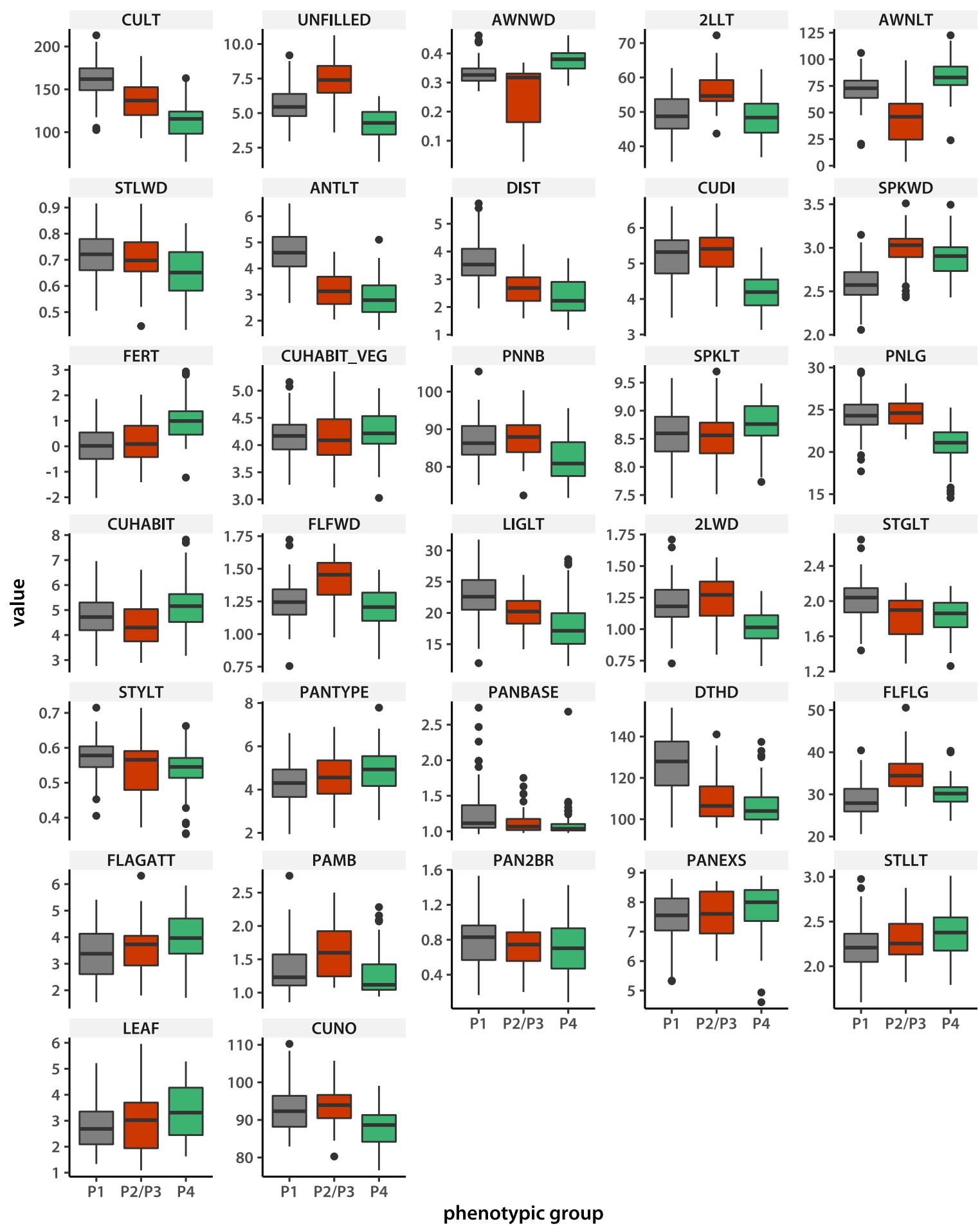

Supplement: Supplementary file 8 [file Data_Sheet_1.ZIP › traitsIRRIbxpP4.pdf]

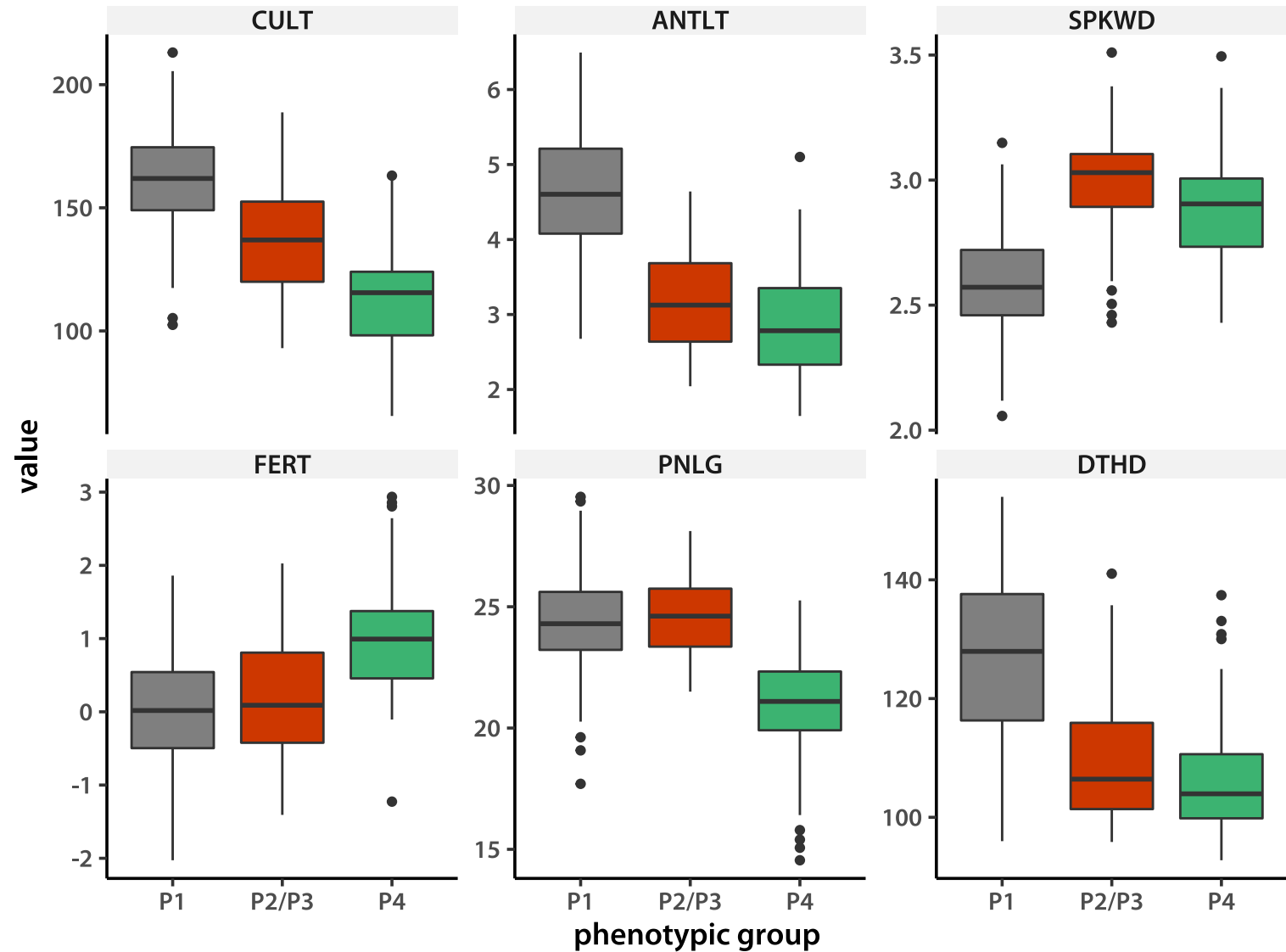

Supplement: Supplementary file 8 [file Data_Sheet_1.ZIP › traitsIRRIbxpP4sixTr.pdf]
