# Supplementary material for: Phenotypic Variation and the Impact of Admixture in the Oryza rufipogon Species Complex (ORSC)
Source: Front Plant Sci. 2022 Jun 13;13:787703. doi: 10.3389/fpls.2022.787703 (PMC9235872; doi:10.3389/fpls.2022.787703)
Supplement: Supplementary file 8 [file traitCorIRRIp4.pdf › traitCorIRRIp4.pdf]

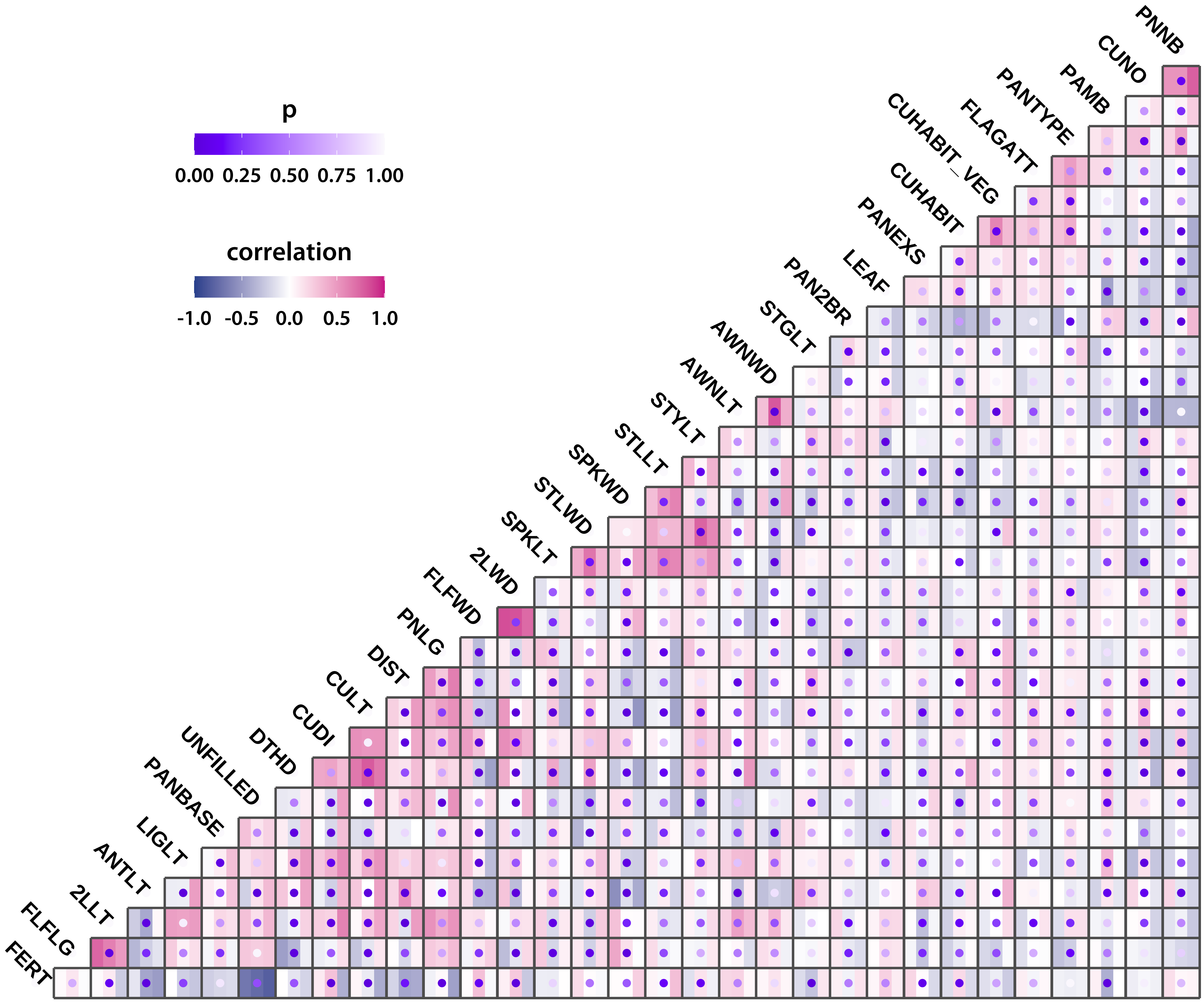
p
0.00 0.25 0.50 0.75 1.00
correlation
-1.0 -0.5 0.0 0.5 1.0
PNNB
CUNO
PAMB
PANTYPE
FLAGATT
CUHABIT_VEG
CUHABIT
PANEXS
LEAF
PAN2BR
STGLT
AWNWD
AWNLT
STYLT
STLLT
SPKWD
STLWD
SPKLT
2LWD
FLFWD
PNLG
DIST
CULT
CUDI
DTHD
UNFILLED
PANBASE
LIGLT
ANTLT
2LLT
FLFLG
FERT
