# Supplementary material for: Phenotypic Variation and the Impact of Admixture in the Oryza rufipogon Species Complex (ORSC)
Source: Front Plant Sci. 2022 Jun 13;13:787703. doi: 10.3389/fpls.2022.787703 (PMC9235872; doi:10.3389/fpls.2022.787703)
Supplement: Supplementary file 9 [file Data_Sheet_2.pdf]

Supplementary Figures 1 to 16 for:

Journal: Frontiers in Plant Science

Research Topic: Reproductive Barriers and Gene Introgression in Rice Species - Volume II

Title: Phenotypic variation and the impact of admixture in the *Oryza rufipogon Species Complex* (*ORSC*)

Authors:
Georgia C. Eizenga[1]*, HyunJung Kim[2], Janelle K. H. Jung[2], Anthony J. Greenberg[3], Jeremy D. Edwards[1], Maria Elizabeth B. Naredo[4], Maria Celeste N. Banaticla-Hilario[4], Sandra E. Harrington[2], Yuxin Shi[2], Jennifer A. Kimball[2], Lisa A. Harper[2], Kenneth L. McNally[4], Susan R. McCouch[2]*

[1] Dale Bumpers National Rice Research Center, USDA-ARS, Stuttgart, AR, USA
[2] Plant Breeding and Genetics Section, School of Integrative Plant Science, Cornell University, Ithaca, NY, USA
[3] Bayesic Research, Ithaca, NY, USA
[4] International Rice Research Institute, Los Baños, Laguna, Philippines

Citation:
Eizenga GC, Kim HJ, Jung JKH, Edwards JD, Naredo MEB, Banaticla-Hilario MCN, Harrington SE, Shi Y, Kimball JA, Harper LA, McNally KL and McCouch SR (2022) Phenotypic Variation and the Impact of Admixture in the *Oryza rufipogon* Species Complex (*ORSC*). Front. Plant Sci. 13:787703. doi: 10.3389/fpls.2022.787703

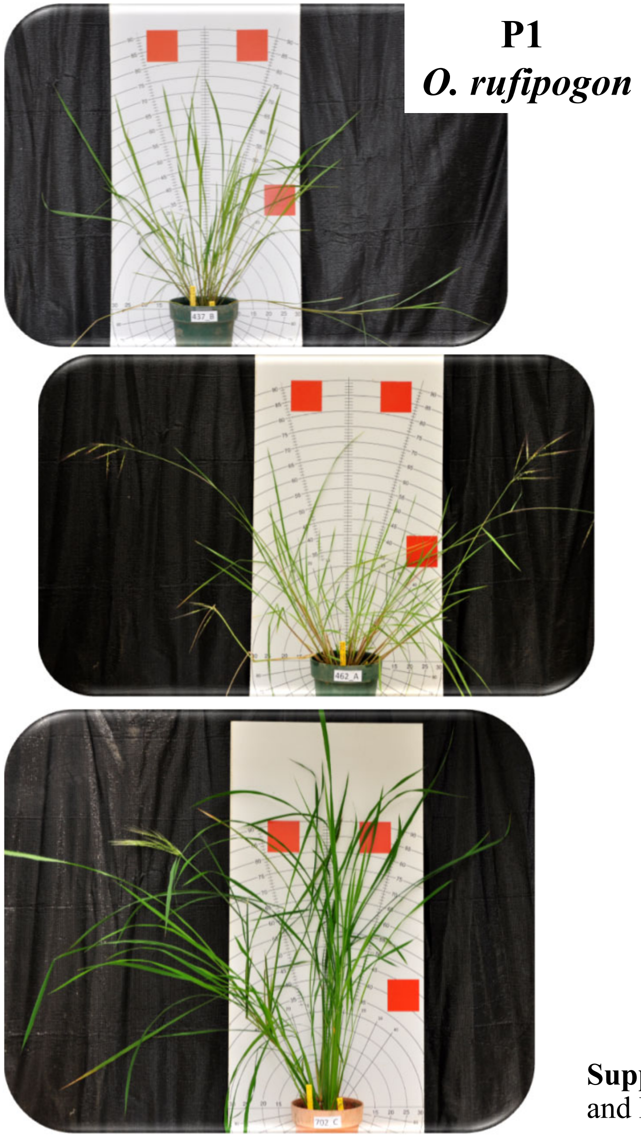

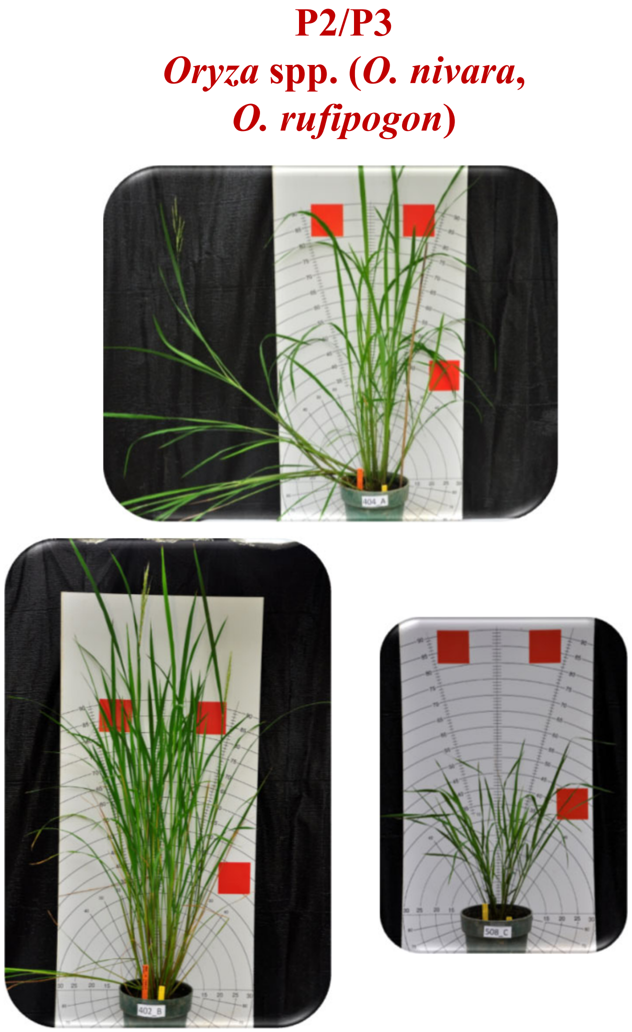

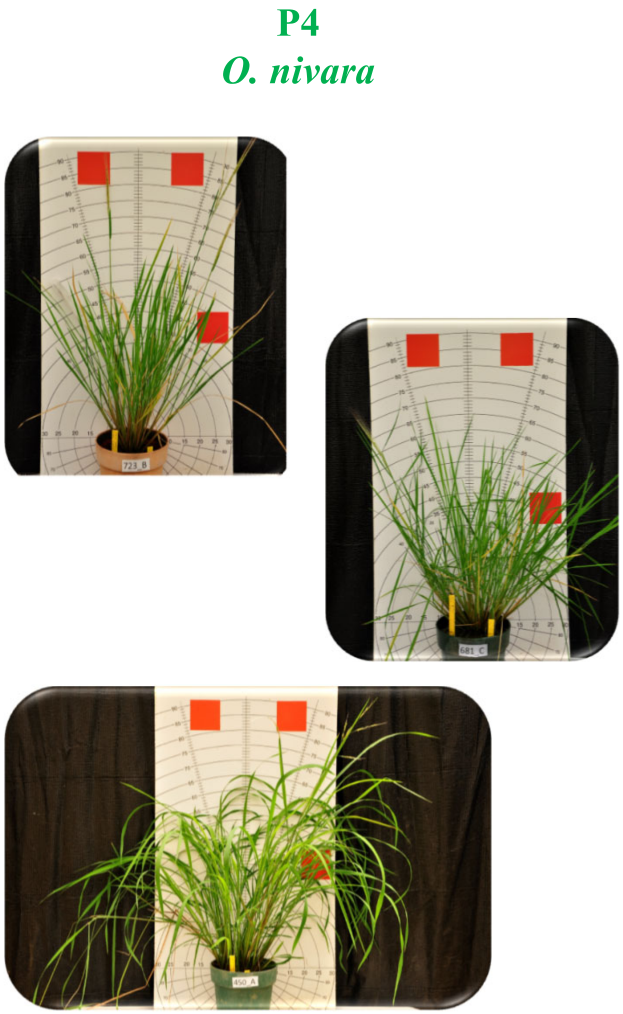

Supplementary Figure 1: Selected images of *ORSC* accessions in the three phenotypic groups, P1, P2/P3 and P4, reported in this study.

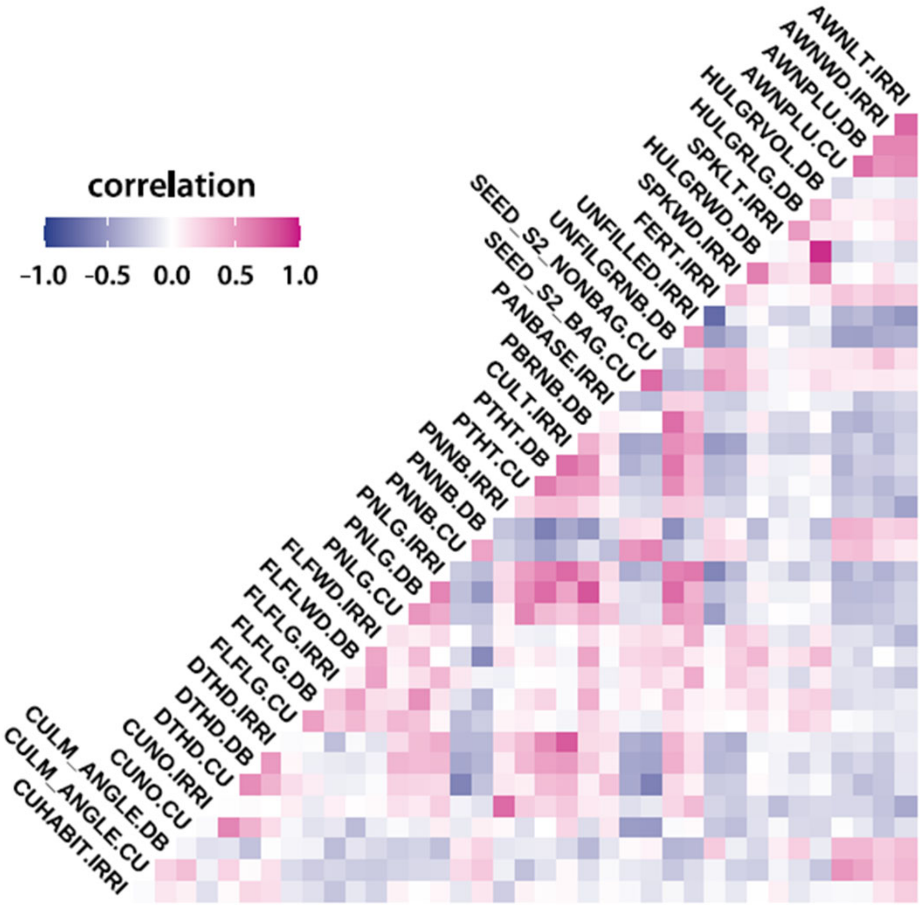

Supplementary Figure 2: Correlations among the 14 phenotypic traits measured at the three locations, the International Rice Research Institute (IRRI), Dale Bumpers National Rice Research Center (DB) and Cornell University (CU) that were the same or similar across two or three datasets. (Trait acronyms and actual values are listed in Supplementary Table 6.)

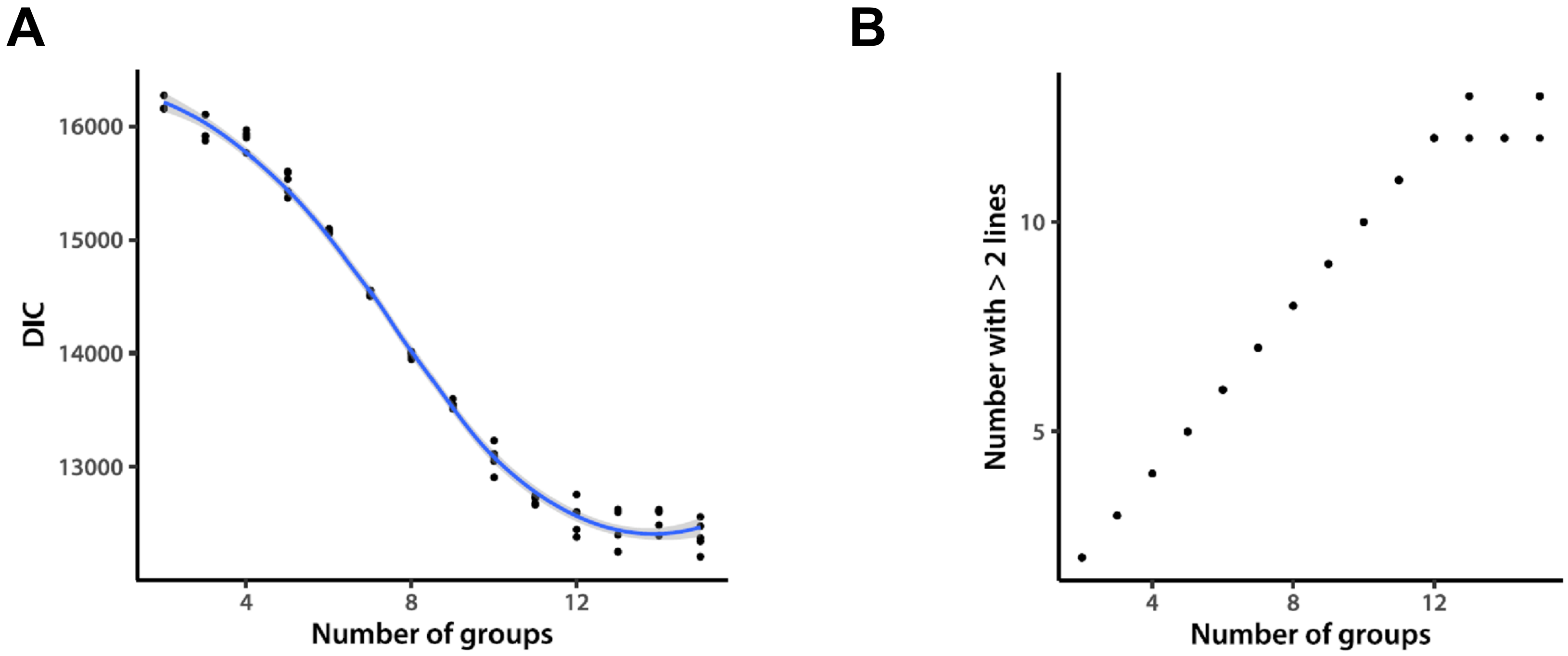

Supplementary Figure 3: Model fit for non-binary IRRI phenotypic data. **(A)** Deviance information criterion (DIC) as a function of the *a priori* set number of groups (N_G). Lower DIC values indicate a better fit. **(B)** The number of inferred groups comprising more than two accessions plotted against the number *of a priori* groups.

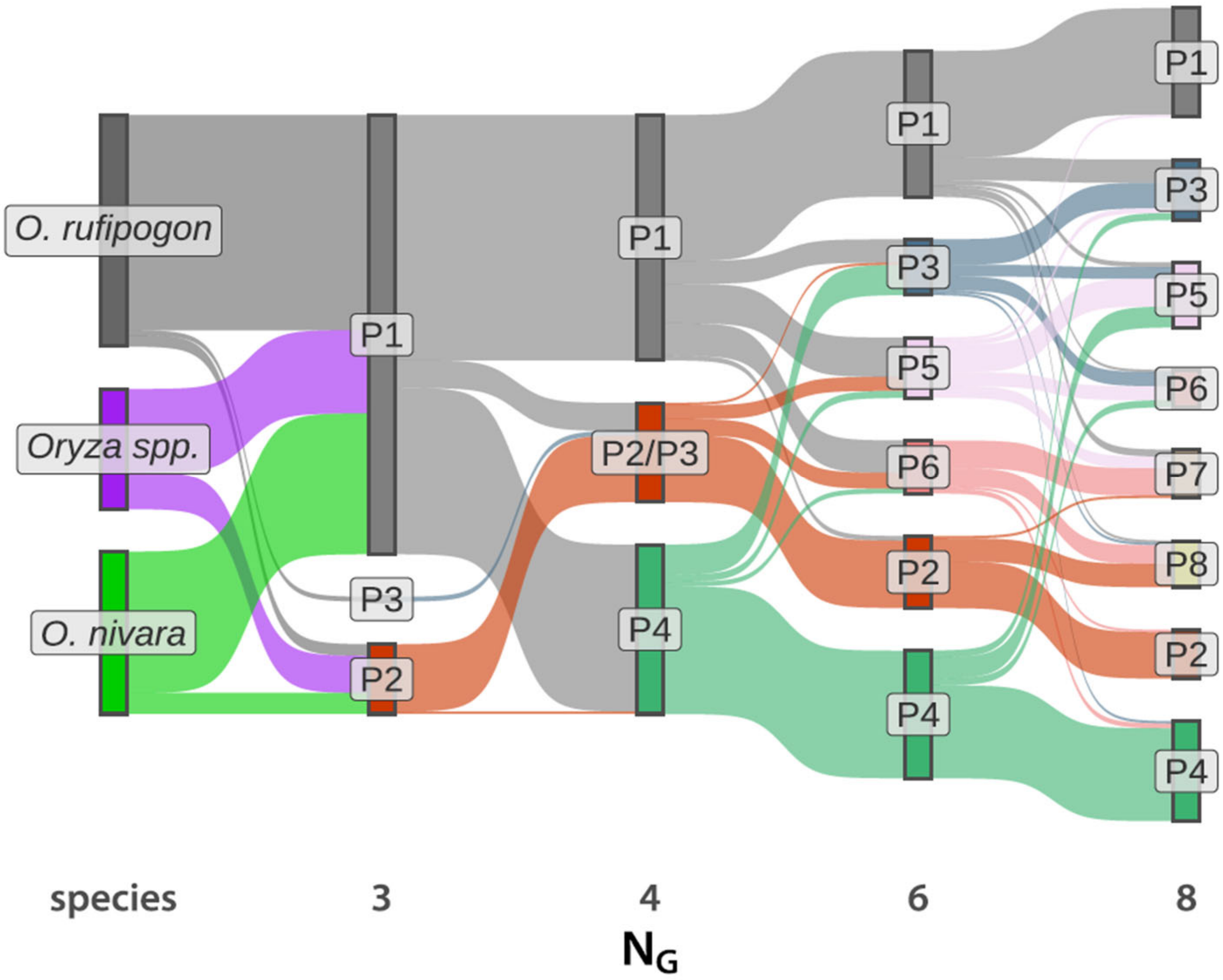

Supplementary Figure 4: Sankey plot tracking the individual accessions as lines or “ribbons” across the groups inferred assuming several N_G values *a priori*, with species designations for comparison. Each accession is an element in the ribbons, colored according to the grouping to the left.

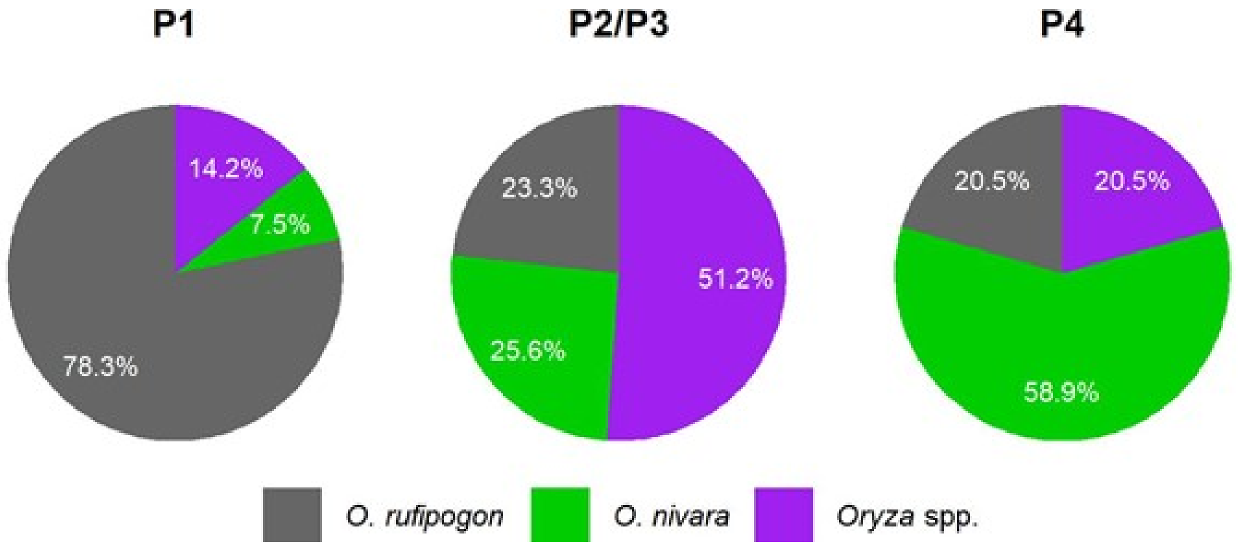

Supplementary Figure 5: Distribution of *O. rufipogon*, *O. nivara* and *Oryza* spp. accessions among the three phenotypic groups.

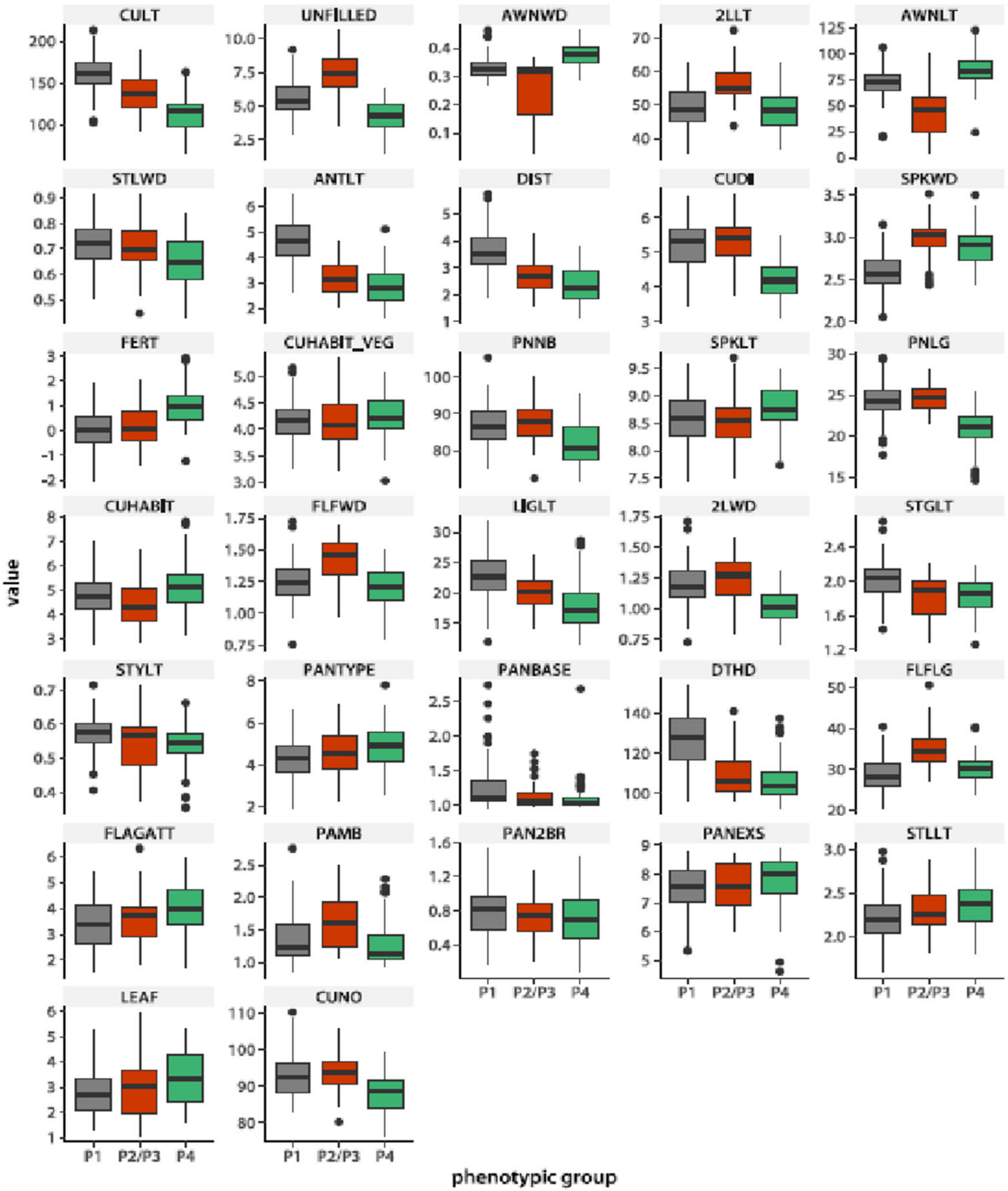

Supplementary Figure 6: Box plots for the distribution of the 32 non-binary phenotypic traits measured at IRRI by the three phenotypic groups. Group medians are depicted by black bars. The traits are ordered by the Hotelling T^2 values. (Trait acronyms are listed in Supplementary Table 3.)

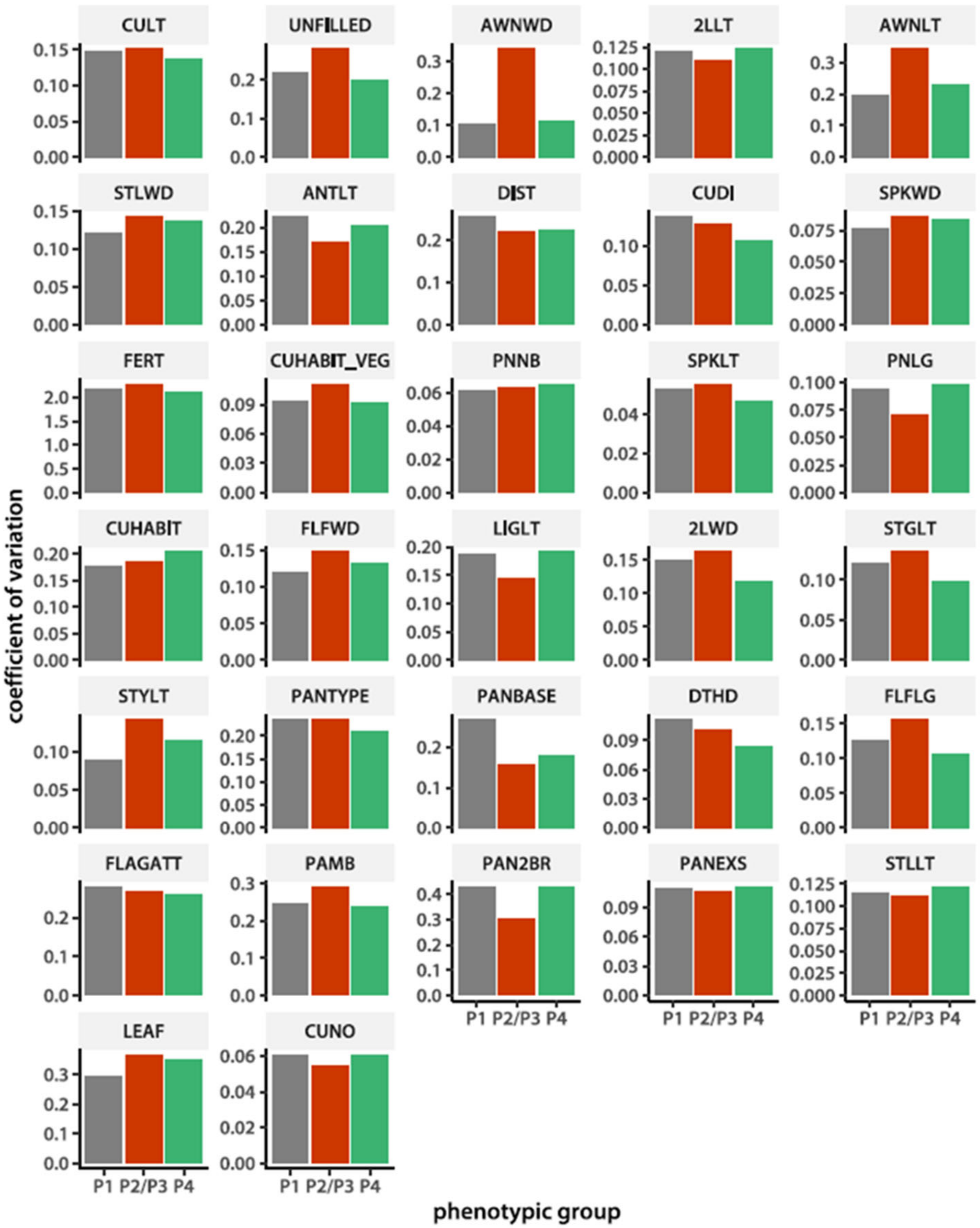

Supplementary Figure 7: Within-group standard deviations of trait values for the 32 non-binary phenotypic traits measured at IRRI by the three phenotypic groups. Bar heights reflect coefficients of variation of trait values within each phenotypic group. Traits are ordered by their Hotelling T^2 values. (Trait acronyms are listed in Supplementary Table 3.)

A

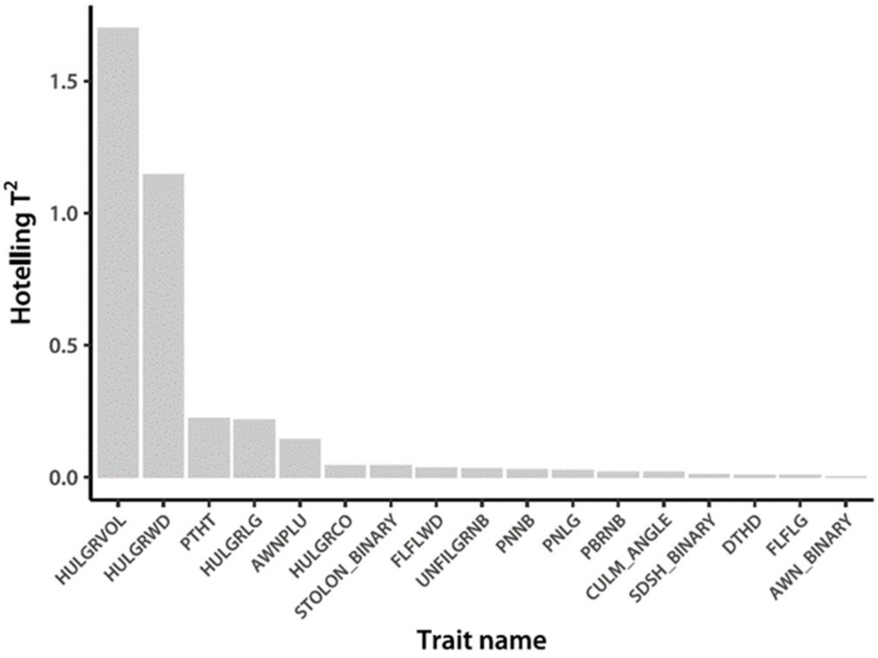

B

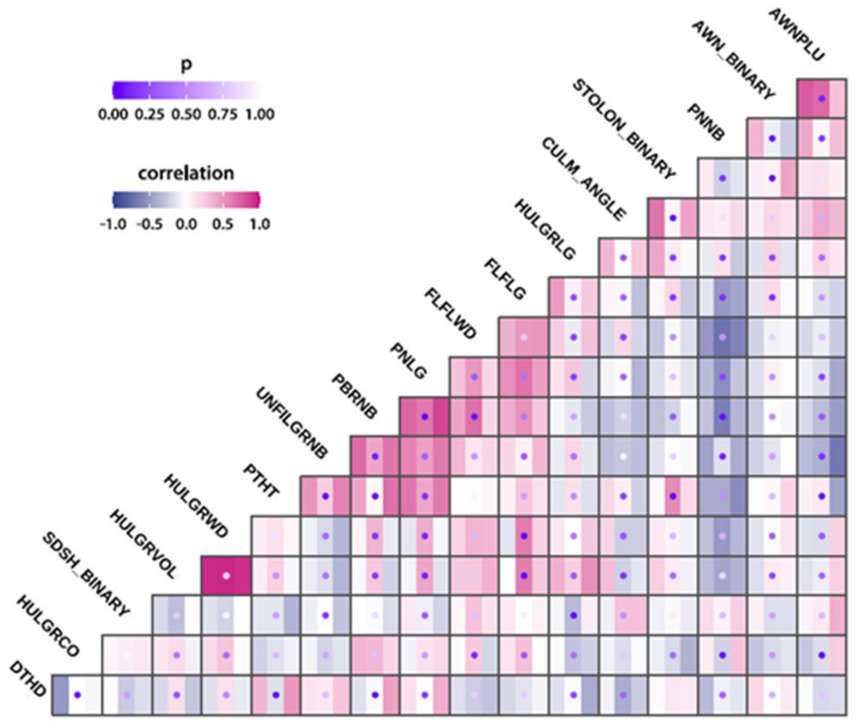

Supplementary Figure 8: Phenotypic identifiers of group divergence based on the Dale Bumpers (DB) data. (**A**) Traits sorted by the strength of the association of their values to $N_G = 3$ group assignments based on Hotelling T^2 values. (**B**) Among-trait correlation estimates within each phenotypic group. Squares mark trait pairs, with the three bars within each square colored according to correlation magnitude in the three phenotypic groups. Dots represent probabilities of observing the correlation differences by chance (lower probabilities reflect high confidence of correlation difference). (Actual values and trait acronyms are listed in Supplementary Table 7.)

A

B

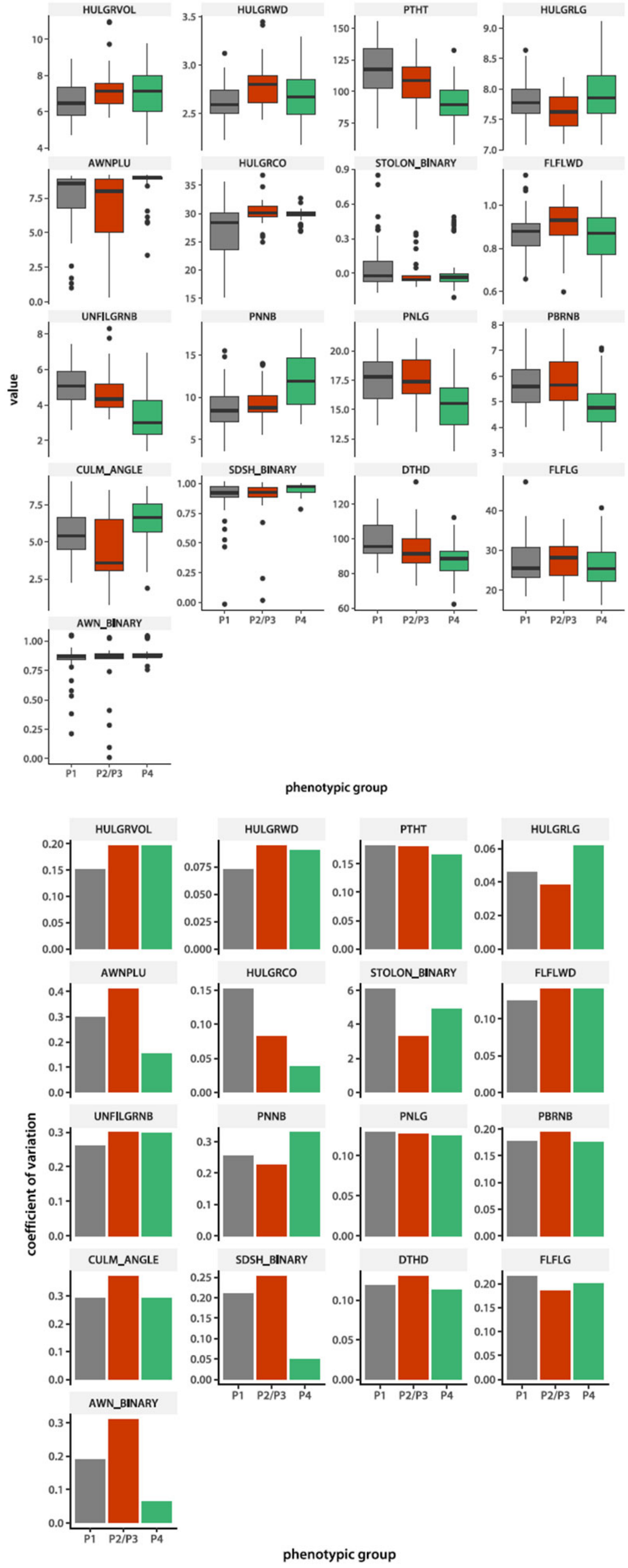

Supplementary Figure 9: Distribution of the Dale Bumpers (DB) phenotypic data. **(A)** Box plots of the distributions for 17 traits measured across three phenotypic groups using the data from the 111 accessions and **(B)** Within-group trait coefficients of variation. Traits are ordered by their Hotelling T^2 values. (Trait acronyms are listed in Supplementary Table 3.)

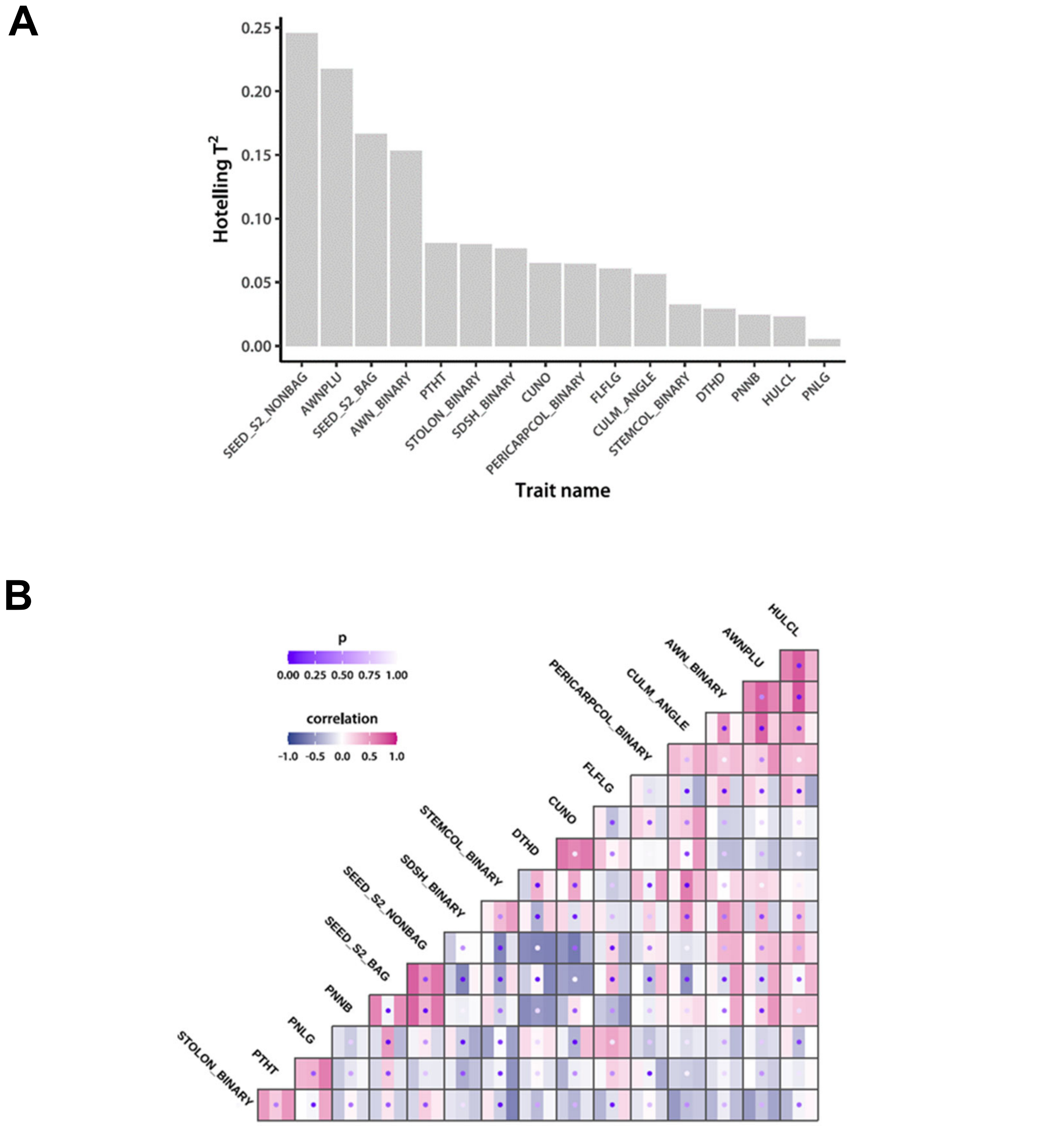

Supplementary Figure 10: Phenotypic identifiers of group divergence based on the Cornell (CU) data. (**A**) Traits sorted by the strength of the association of their values to $N_G = 3$ group assignments based on Hotelling T^2 values. (**B**) Among-trait correlation estimates within each phenotypic group. Squares mark trait pairs, with the three bars within each square colored according to correlation magnitude in the three phenotypic groups. Dots represent probabilities of observing the correlation differences by chance (lower probabilities reflect high confidence of correlation difference). (Actual values and trait acronyms are listed in Supplementary Table 7.)

A

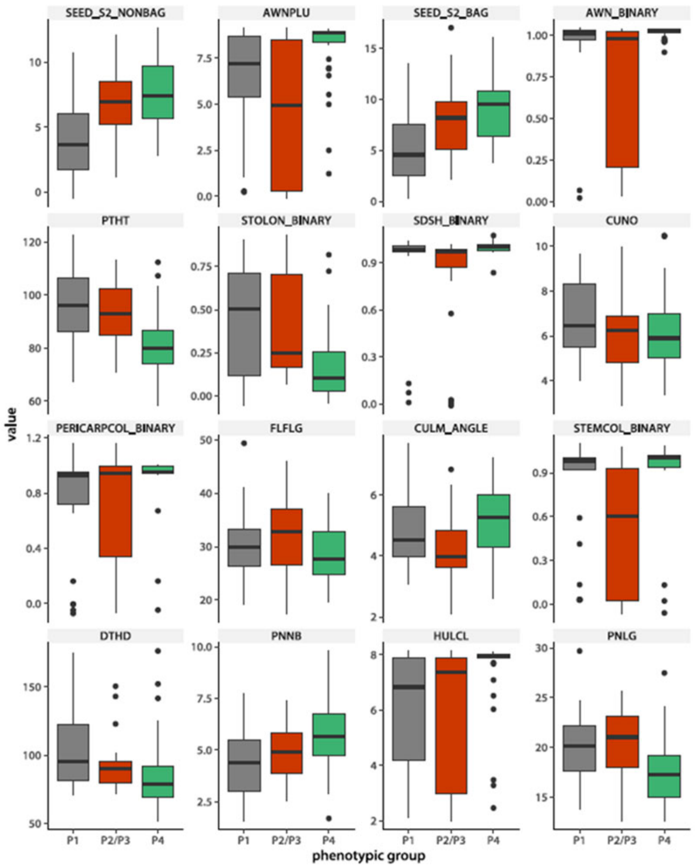

B

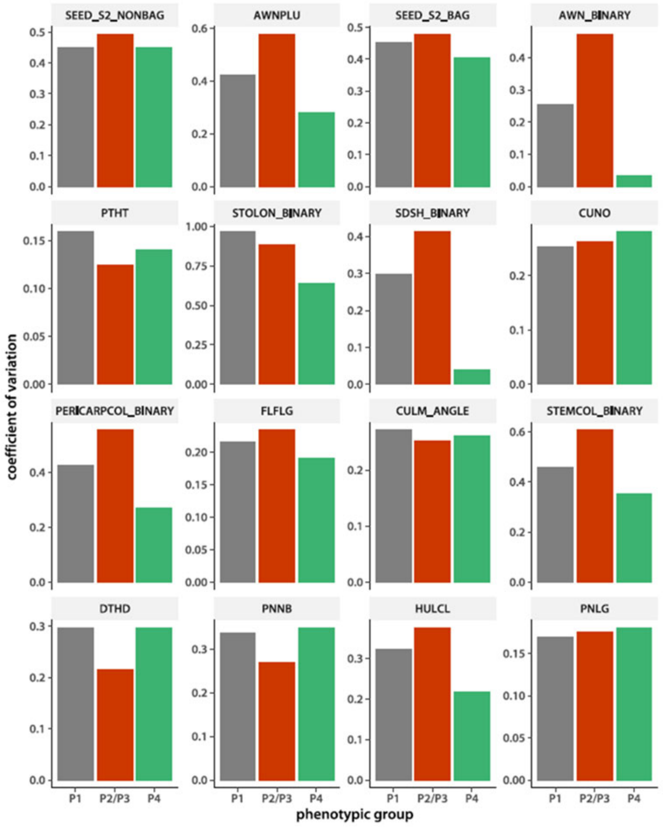

Supplementary Figure 11: Distribution of the Cornell University (CU) phenotypic data. **(A)** Box plots of the distributions for 16 traits measured across three phenotypic groups using the data from the 92 accessions and **(B)** Within-group trait coefficients of variation. Traits are ordered by their Hotelling T^2 values. (Trait acronyms are listed in Supplementary Table 3.)

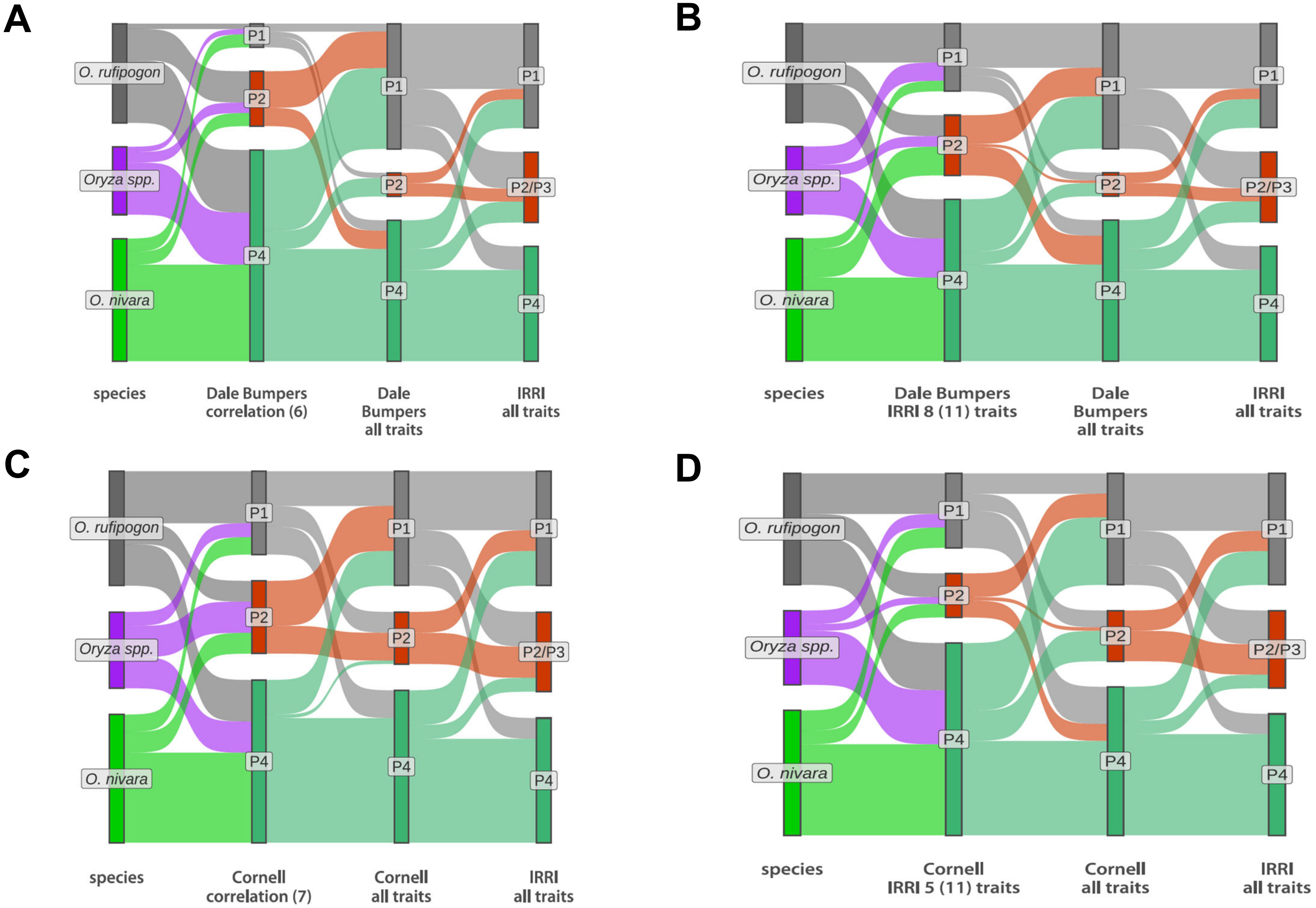

Supplementary Figure 12: Sankey plots comparing accession membership using the Dale Bumpers and Cornell data using $N_G = 3$ to the species taxonomy and the IRRI phenotypic group assignments using all 32 traits ($N_G = 4$). Sankey plots comparing **(A)** the top six traits in the Dale Bumpers correlation and **(B)** the eight traits that correspond to 11 top traits from the IRRI analysis (Table 1) to all 18 traits measured at Dale Bumpers. Sankey plots comparing **(C)** the top seven traits in the Cornell correlation and **(D)** the five traits that correspond to the top 11 traits from the IRRI analysis (Table 1) to all 16 traits measured at Cornell.

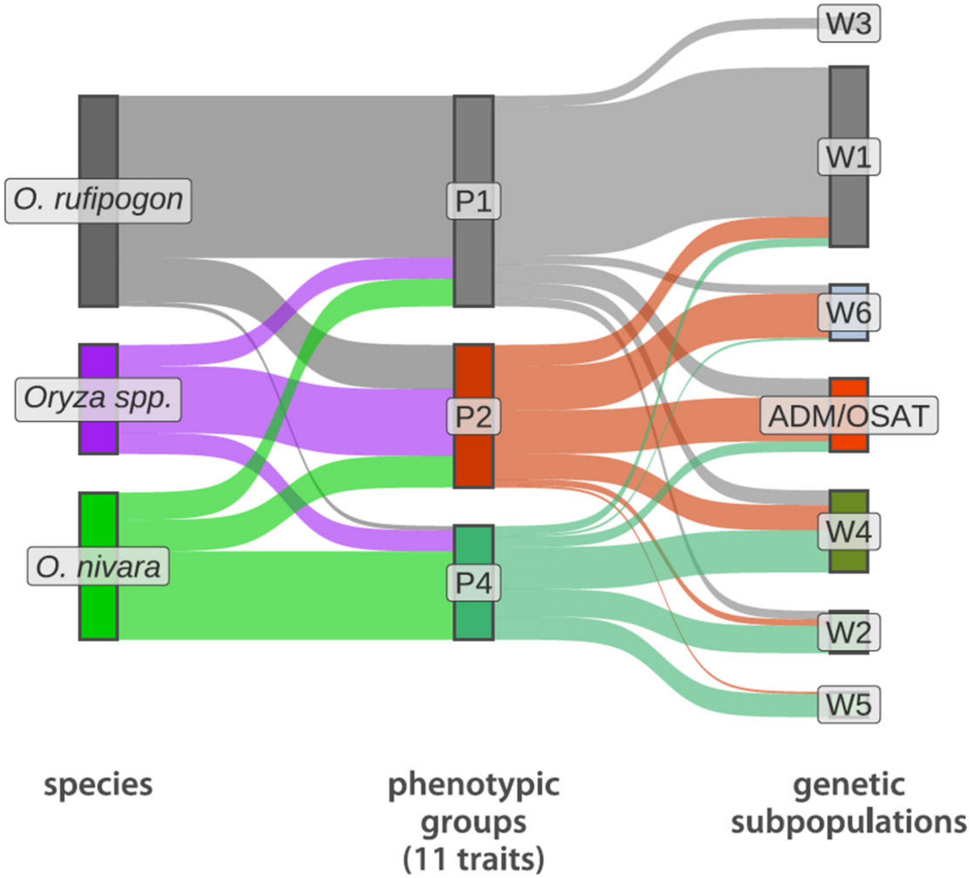

Supplementary Figure 13: Sankey plot depicting correspondence between traditional species designations, three phenotypic groups determined from the 11 top phenotypic traits by the correlation and value of the phenotypic trait data collected at IRRI, and the Kim et al. (2016) genetic subpopulations. (ADM/OSAT are accessions with a high proportion of admixture with *O. sativa*.)

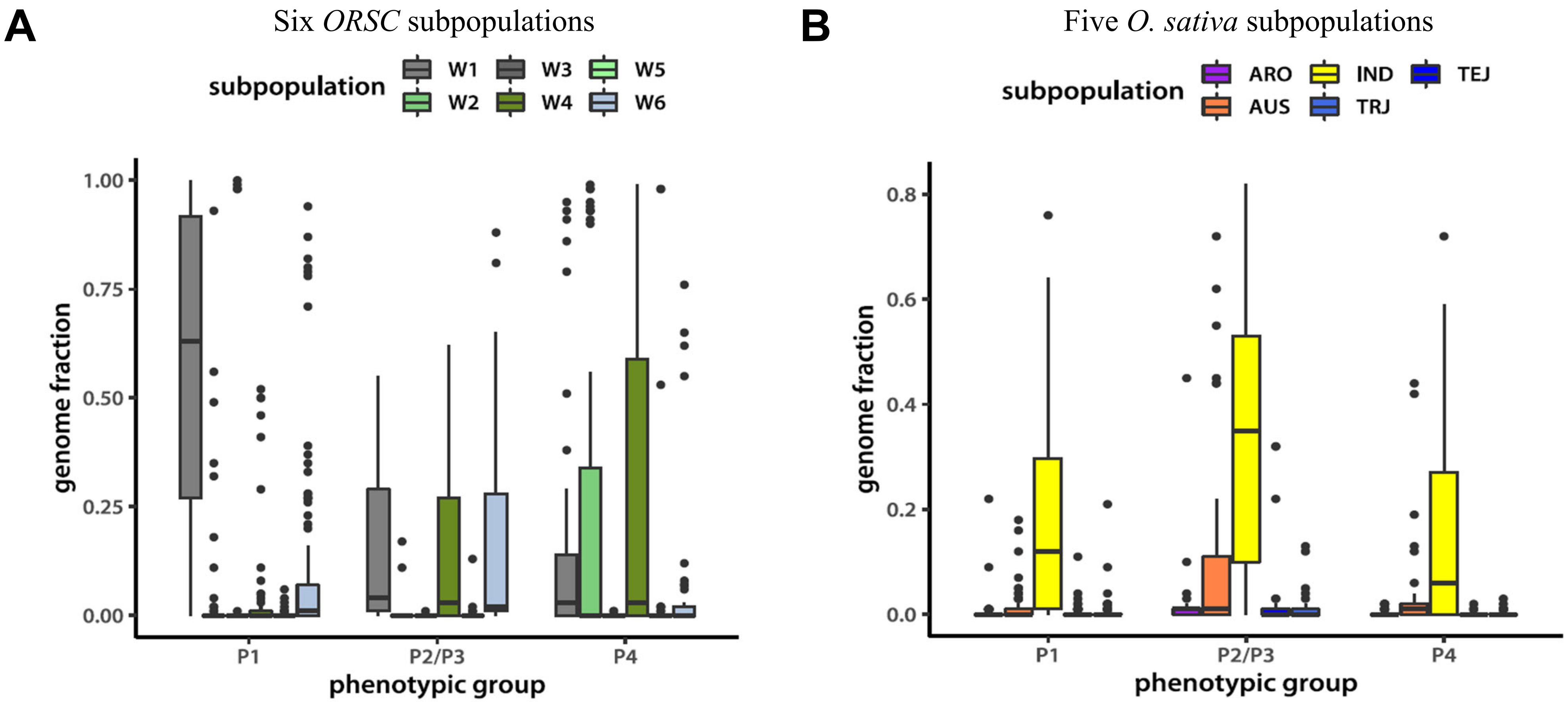

Supplementary Figure 14: Genotypic composition of the three phenotypic groups. Distributions of genome fractions of the (**A**) six *ORSC* subpopulations (W1-W6) and (**B**) five *O. sativa* subpopulations (IND, AUS, TRJ, TEJ, and ARO) in each of the three phenotypic groups.

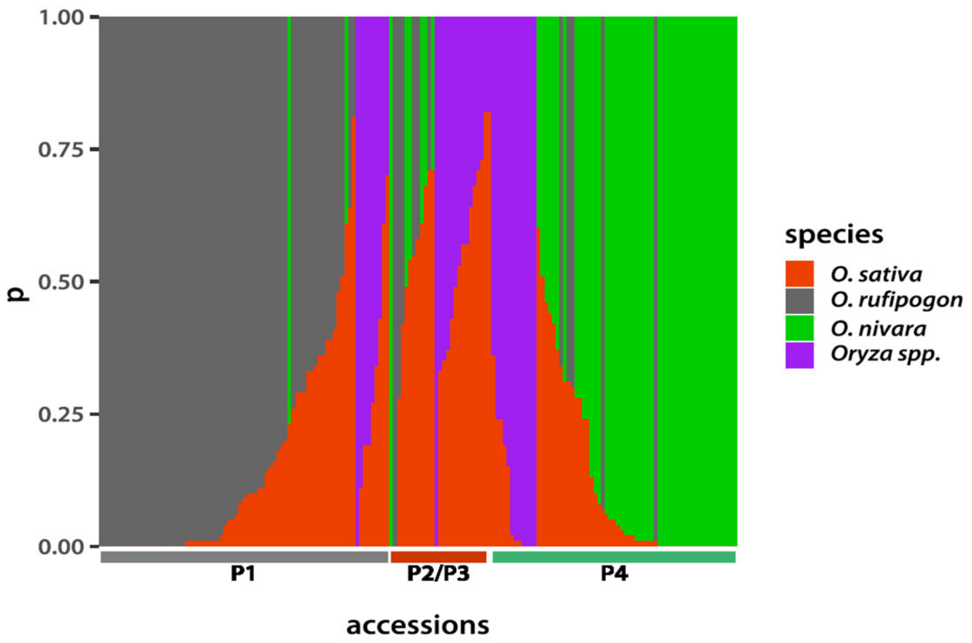

Supplementary Figure 15: Genome fractions of accessions originating from *O. sativa*, regardless of *O. sativa* subpopulation, based on the RFMix analysis. Genome portions not coming from *O. sativa* are marked with the subspecies names listed in IRRI-GRIN-Global. Each bar represents a single accession and the phenotypic group the accession belongs to is on the *x*-axis. Genomic fractions are indicated on the *y*-axis.

A

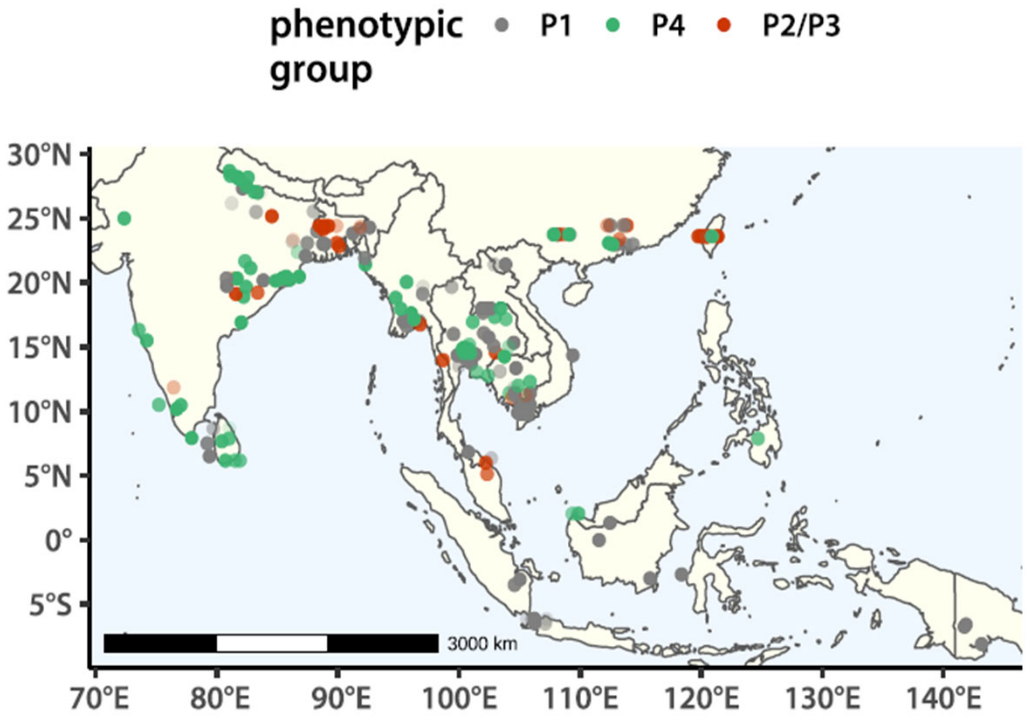

B

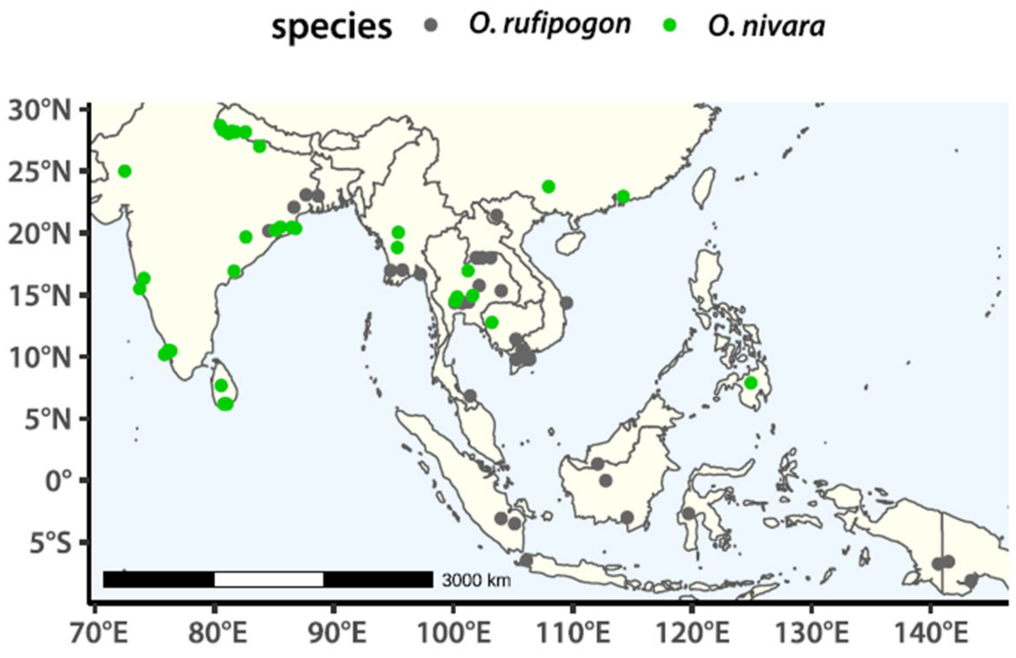

Supplementary Figure 16: Geographical distribution of *ORSC* accessions with the intensity of the dots indicating *ORSC* genome fractions. **(A)** Accession distributions according to the three phenotypic groups and **(B)** distribution of the "core" *O. rufipogon* and *O. nivara* accessions whose group is consistently inferred using the species taxonomy, top by value and top correlation trait sets (Table 1).
